# Supplementary material for: Primary care doctor and nurse consultations among people who live in slums: a retrospective, cross-sectional survey in four countries
Source: BMJ Open. 2022 Jan 7;12(1):e054142. doi: 10.1136/bmjopen-2021-054142 (PMC8744106; doi:10.1136/bmjopen-2021-054142)
Supplement: Supplementary data [file bmjopen-2021-054142supp003.pdf]

SUPPLEMENTARY INFORMATION

A Health system indicators

Table A1 reports major health system and population health indicators for the participating countries.

Table A1. Key indicators of health care systems in participating countries (Source: World Bank and WHO Global Health Observatory[1])

Key indicators	Nigeria	Kenya	Pakistan	Bangladesh
Life expectancy	54	66	67	72
GDP per capita (PPP; Int$)	5,991	3,468	5.572	4,372
Under five mortality (per 1,000 births)	120	41	69	30
Out of pocket expenditure as % of all health expenditures	75%	28%	65%	72%
Government health expenditure per capita (Int$)	27	52	40	16
Proportion with expenditures on healthcare greater than 10% of total household expenditure	15%	5%	13%	25%
Antenatal care coverage – at least four visits	49%	105%	51%	37%
ARV coverage among those with HIV	53%	68%	10%	22%
Hib (Hib3) coverage among one-year-olds	57%	92%	75%	98%
Tetanus coverage among neonates	60%	88%	85%	98%
Tuberculosis effective treatment coverage	20%	39%	64%	58%
Average of 13 International Heath Regulation core capacity scores	51	58	51	78

GDP: gross domestic product; Hib: Haemophilus influenzae type b vaccine; Int$: international dollar; PPP: purchasing power parity

B Supplementary information on methods

B1 Definitions of health care facilities

Table B1 Definitions of healthcare facilities used in the mapping and surveying of healthcare facilities at each site

Facility type	Definition
Clinic/centre	A facility used for the diagnosis and treatment of outpatients. Allopathic only. Subcategories include specialist clinics: vaccination; family planning; eye and vision; ear, nose, and throat; maternity and antenatal care; palliative care; and general primary or community care.
Dental clinic	A facility providing dentistry/dental care.
Hospital/secondary care facility	A health care organization that has a governing body, an organized medical staff and professional staff and inpatient facilities and provides medical nursing and related services for ill and injured patients 24 hrs per day, seven days per week
Residential or nursing care facility	Facilities that provide inpatient care specializing in nursing or long-term residential care but which are not hospital
Laboratory or sonologist clinic	A room or building equipped for testing, researching, or studying fluids, materials, or tissues from patients.
Pharmacy or medical store	A facility used by pharmacists for the compounding and dispensing of medicinal preparations and other associated professional and administrative services
Transportation services	Provider who moves a patient, specimen, or equipment from one location or another
General shop or kiosk selling healthcare products	Non-specialised vendor without healthcare professional selling health care products such as pain killers, first aid, or spectacles
Traditional practitioner	A practitioner of non-allopathic, traditional, alternative, or faith-based health services. Sub-categories include: traditional medicine, faith-based services, homeopathy, acupuncture, bone healing, and cupping.
Traditional products vendor	Vendor specialising in traditional (non-allopathic) medical products such as herbs.

B2 Supplementary Survey in Site PK1

An error with the electronic survey forms was identified following data collection in site PK1. The household survey form was completed successfully, including the household roster, however due to an error in the code to generate a randomly sampled adult the first recorded adult was instead sampled. This resulted in an almost exclusively male sample, which was not identified in the field at the time. After identification of this error we aimed to complete a set of individual surveys with randomly sampled women from the household rosters. However, the Covid-19 pandemic had started, so a telephone survey was deemed to be safer for the field workers. We sampled 400

households with at least one adult women and who had provided consent and contact details for follow-up from the original sample of households. Field workers then followed the same procedures as the main survey. Survey weights were modified for the sample from PK1 to reweight gender back to the population proportion.

B3 Statistical methods

Choice model

Choices

We define the choice set facing individual as: private clinic, public clinic, private hospital, and public hospital. In sites BD1 and PK1 we also include “private office” as a fifth option as a number of visits were recorded to these locations, whereas there were little to no visits to this type of provider in the other sites.

Choice-varying covariates

We consider two covariates that vary for each individual and choice: the price of the visit and the time to travel to the provider. The price includes consultation fees and transport cost. The price is reported in local currency (Naira, Kenyan Shillings, Pakistani Rupees, Taka). The time to provider is the travel time reported for the visit in minutes. We standardize both cost and time (dividing by the standard deviation) to ensure all variables are on the same scale to facilitate model fitting.

Individual-level covariates

From the survey data we extract the following individual level variables: age, sex, secondary education or not, if the visit was for an acute or communicable condition (excluding HIV), if the visit was for generalised or chronic pain, and if the monthly consumption expenditure was above Int$100 per person per month. For use in the models we standardize the age variable to facilitate model fitting.

Statistical Model

We take a Bayesian approach to model specification and fitting given the complex hierarchical structure of the model.

Each individual $i \in 1{:}N$ chooses from $k \in 1{:}K$ possible options (K is either 4 or 5). The healthcare provider options are described by a vector of choice attributes for each individual $X_{ik} = \left[C_{ik}, C_{ik}^2, T_{ik}\right]'$. This vector includes the cost, cost squared, and time. We include cost squared to allow for more flexibility between demand and price. Each individual has "part worth" preferences over the choice attributes β_i so that the utility of each choice for each individual is:

$$u_{ik} = \theta_k + X_{ik}\beta_i + u_{ik}$$

where u_{ik} is IID Gumbel distributed and θ_k are choice constant terms. Given the assumed distribution of the random error, the probability an individual chooses choice k is

$$\Pr(y_{ik} = k | X_{ik}, \beta_i, \theta_k) = \frac{\exp(\theta_k + X_{ik}\beta_i)}{\sum_{j=1}^{K} \exp\left(\theta_j + X_{ij}\beta_i\right)}$$

For more information see[2].

Hierarchical prior on model parameters

The model parameters β_i are allowed to vary by individual – they are so-called "random parameters". To model these parameters we use a hierarchical prior that "partially pools" information across individuals. We also allow these parameters to vary according to a vector of individual-level covariates W_i and further, we allow for correlation between parameters so that, for example, individuals more sensitive to price might be less sensitive to distance. In particular,

$$\beta_i \sim MVN(\beta + \Gamma W_i, \Sigma)$$

where β are mean values, Γ is a matrix that "loads" the individual covariates on to the parameters, and Σ is a covariance matrix. We can decompose the covariance matrix as $\Sigma = \mathrm{diag}(\tau)\Omega\,\mathrm{diag}(\tau)$, where τ is a scale vector and Ω is the correlation matrix of the variation across individuals. The correlation matrix can be further decomposed as $\Omega = L_\Omega L_\Omega'$ where L_Ω is the lower-triangular Cholesky decomposition. Therefore,

$$\beta_i = \beta + \Gamma W_i + \mathrm{diag}(\tau) L_\Omega z_i$$

$$z_i \sim N(0,1)$$

Missing data

We use an imputation-type procedure for the costs and times that we do not observe, i.e. for the choices not taken. The model below is part of the overall model and is estimated at the same time

so that the uncertainty in the imputed costs and times is carried through to the overall results. We explain the procedure for costs but use an identical model for times. Given that both variables are (often heavily) right-skewed we use log costs and time for the missing data models. We observe N values for log cost, one for each individual: $c_{ik}^{(obs)}$. We specify:

$$c_{ik}^{(obs)} = \mu_k + Z_i\gamma + u_i$$

where μ_k is a choice-specific intercept, Z_i is a vector of individual level covariates that may predict the cost of the visit (age, age squared, sex, reason for visit), γ are model parameters, and $u_i \sim N(0, \sigma_u^2)$ are IID error terms. We fit the above model using the observed data and then for the unobserved costs we sample from:

$$c_{ik}^{(mis)} \sim N(\mu_k + Z_i\gamma, \sigma_u^2)$$

so that the cost covariate is

$$C_{ik} = \begin{cases} \exp\left(c_{ik}^{(obs)}\right) & if\ y_{ik} = 1 \\ \exp\left(c_{ik}^{(mis)}\right) & if\ y_{ik} = 0 \end{cases}$$

Priors

We specify weakly informative priors on the model parameters (all N(0,1) except for the correlation matrix for which we use the lkj prior).

Estimation

We use Stan 2.19 to fit the model[3].

Elasticity of demand

To quantify the response to price or travel time we calculate the price and travel time elasticity of demand. We do this by calculating the arc elasticity:

$$\frac{(\mathrm{Pr1}\ - \mathrm{Pr0})/((\mathrm{Pr1} + \mathrm{Pr0})/2)}{(p_1 - p_0)/((p_1 + p_0)/2)}$$

where p_1 and p_0 are different prices or times and $\mathrm{Pr1}$ and $\mathrm{Pr0}$ are the probabilities of visiting a provider under each of the two prices or times, respectively.

C Additional Results

Table C1. Respondent care needs and outpatient primary care use, N(%)

Outcome	NG1	NG2	NG3	KE1	KE2	PK1	BD1
Adults							
Total	1,278 (100%)	840 (100%)	802 (100%)	1,008 (100%)	1,085 (100%)	1,112 (100%)	990 (100%)
Needed healthcare in the previous 12 months	772 (60%)	490 (58%)	418 (52%)	710 (70%)	906 (84%)	623 (56%)	959 (97%)
Received care when last needed	762 (60%)	481 (57%)	404 (50%)	674 (67%)	884 (81%)	596 (54%)	918 (93%)
Outpatient visit in last 12 months	626 (49%)	367 (44%)	309 (39%)	665 (66%)	867 (80%)	531 (48%)	900 (91%)
Outpatient visit to doctor or nurse	391 (31%)	215 (26%)	201 (25%)	405 (40%)	377 (35%)	498 (45%)	257 (26%)
Outpatient visit to doctor or nurse for new condition or routine visit	293 (23%)	132 (16%)	151 (19%)	310 (31%)	315 (29%)	291 (26%)	174 (18%)
Children (under 12)							
Total	128 (100%)	69 (100%)	79 (100%)	537 (100%)	421 (100%)	528 (100%)	658 (100%)
Needed healthcare in the previous 12 months	68 (53%)	29 (42%)	41 (52%)	482 (90%)	368 (87%)	409 (77%)	635 (97%)
Received care when last needed	68 (53%)	28 (41%)	41 (52%)	466 (87%)	363 (86%)	397 (75%)	627 (95%)
Outpatient visit in last 12 months	53 (41%)	24 (35%)	39 (49%)	462 (86%)	358 (85%)	374 (71%)	608 (92%)
Outpatient visit to doctor or nurse	36 (28%)	13 (19%)	24 (30%)	320 (60%)	205 (49%)	367 (70%)	136 (21%)
Outpatient visit to doctor or nurse for new condition	27 (21%)	11 (16%)	19 (24%)	283 (53%)	190 (45%)	329 (62%)	120 (18%)

Table C2. Summary statistics of study populations and national comparisons (DHS surveys – national results and urban results in parentheses where relevant)

Variable		Nigeria				Kenya			Pakistan		Bangladesh	
		**NG1**	**NG2**	**NG3**	**DHS 2018**	**KE1**	**KE2**	**DHS 2014**	**PK1**	**DHS 2017/8**	**BD1**	**DHS 2014**
Households												
N (completed)		1,286	845	812	-	1,018	1,089	-	988	-	1,035	-
Response rate (%)		83	69	68	99.3	69	57	99	73	96	94	99
Household size		3.7 (1.9)	3.4 (1.8)	4.2 (2.0)	4.3	3.2 (2.2)	3.4 (1.7)	3.9	5.7 (3.0)	6.6	3.8 (1.8)	4.5
Wealth quintile (%)	Bottom	0	0	0	20 (4.2)	0	0	20 (6)	0	20 (3)	0	20 (7)
	Lower	2	1	1	20 (8.1)	14	0	20 (8)	0	20 (7)	0	20 (6)
	Middle	50	59	23	20 (18.8)	59	53	20 (11)	84	20 (17)	21	20 (12)
	Upper	48	40	76	20 (30.6)	27	46	20 (26)	14	20 (31)	78	20 (26)
	Top	0	0	0	20 (38.4)	7	1	20 (49)	2	20 (42)	1	20 (49)
Monthly expenditure (Int$), median [IQR]	Total	312 [197, 487]	323 [210, 496]	502 [319, 815]	-	193 [131, 305]	187 [133, 295]	-	1,010 [672, 1,471]	--	490 [321, 770]	-
	Per person	97 [59, 162]	106 [69, 172]	144 [94, 231]	-	74 [45, 125]	96 [61, 156]	-	196 [146, 294]	-	110 [80, 162]	-
Individuals												
Age (%)	Under 5	11	9	10	15.4	13	12	14	11	13	11	10
	5 – 19	34	31	33	36.4	37	26	38	30	36	32	34
	20 – 44	36	35	39	32.0	37	51	33	41	34	45	36
	45 – 64	14	17	15	11.8	11	10	11	15	13	10	15
	65 and over	5	8	3	4.1	3	1	4	3	4	2	6
Male (%)		49	47	50	-	50	55	-	50	-	53	-
Education over 18s (%)	Completed primary	13	16	17	24.7	25	23	16	13	9	31	9
	Completed secondary	24	31	34	31.7	13	30	11	9	9	4	4
	Completed tertiary	11	6	20	12.1	5	6	7	0	12	4	10
Currently working over 18 (%)		72	76	74	75.5	65	77	81	57	96	75	98

Table C3. Outpatient primary care consultation rates and outpatient doctor and nurse consultation rates (visits per person-year (95% CI)).

Site	Outpatient consultation (new conditions)			Outpatient consultation (all conditions)		
	**Crude**	**WHO age adjusted**	**INDEPTH age adjusted**	**Crude**	**WHO age adjusted**	**INDEPTH age adjusted**
Adults						
NG1	0.31 (0.29, 0.33)	0.30 (0.28, 0.32)	0.30 (0.28, 0.32)	0.42 (0.40, 0.44)	0.42 (0.40, 0.44)	0.40 (0.38, 0.42)
NG2	0.25 (0.23, 0.27)	0.21 (0.19, 0.23)	0.20 (0.18, 0.22)	0.39 (0.35, 0.43)	0.32 (0.30, 0.34)	0.29 (0.27, 0.31)
NG3	0.21 (0.19, 0.23)	0.23 (0.21, 0.25)	0.22 (0.20, 0.24)	0.28 (0.26, 0.30)	0.31 (0.29, 0.33)	0.29 (0.27, 0.31)
KE1	0.77 (0.73, 0.81)	0.77 (0.73, 0.81)	0.72 (0.68, 0.76)	1.07 (1.03, 1.11)	1.17 (1.13, 1.21)	1.04 (1.00, 1.08)
KE2	0.76 (0.72, 0.80)	0.73 (0.69, 0.77)	0.70 (0.66, 0.74)	0.93 (0.89, 0.97)	1.06 (1.02, 1.10)	0.95 (0.91, 0.99)
PK1	0.60 (0.56, 0.64)	0.58 (0.54, 0.62)	0.56 (0.52, 0.60)	0.79 (0.75, 0.83)	0.85 (0.81, 0.89)	0.77 (0.73, 0.81)
BD1	1.08 (1.04, 1.12)	1.21 (1.15, 1.26)	1.16 (1.12, 1.20)	1.52 (1.46, 1.58)	1.73 (1.67, 1.79)	1.59 (1.53, 1.65)
Children (under 12)						
NG1	0.23 (0.15, 0.31)	-	-	0.29 (0.19, 0.39)	-	-
NG2	0.12 (0.04, 0.20)	-	-	0.15 (0.05, 0.25)	-	-
NG3	0.26 (0.14, 0.38)	-	-	0.34 (0.20, 0.48)	-	-
KE1	1.51 (1.41, 1.61)	-	-	1.74 (1.62, 1.86)	-	-
KE2	1.21 (1.11, 1.31)	-	-	1.30 (1.18, 1.42)	-	-
PK1	1.68 (1.56, 1.80)	-	-	1.85 (1.73, 1.97)	-	-
BD1	0.91 (0.83, 0.99)	-	-	1.04 (0.96, 1.12)	-	-
Children (under 5)						
NG1	0.24 (0.10, 0.38)	-	-	0.30 (0.14, 0.46)	-	-
NG2	0.13 (0.00, 0.27)	-	-	0.21 (0.03, 0.39)	-	-
NG3	0.38 (0.14, 0.62)	-	-	0.40 (0.16, 0.64)	-	-
KE1	2.17 (1.97, 2.37)	-	-	2.57 (2.35, 2.79)	-	-
KE2	1.62 (1.44, 1.80)	-	-	1.68 (1.50, 1.86)	-	-
PK1	2.33 (2.11, 2.55)	-	-	2.46 (2.24, 2.68)	-	-
BD1	1.35 (1.21, 1.49)	-	-	1.50 (1.36, 1.63)	-	-

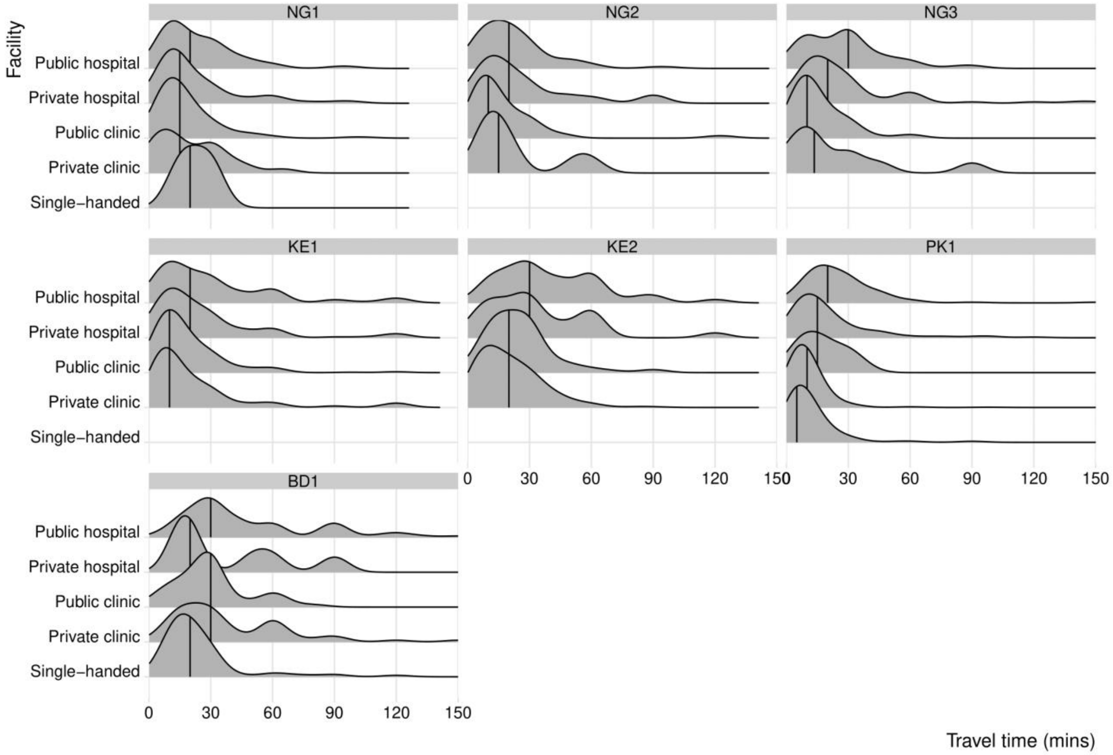

Figure C1 Distribution of reported travel times to different providers by study site with vertical lines indicating the median of the distribution.

Figure C2 Reported "primary reason" for visiting the doctor or nurse.

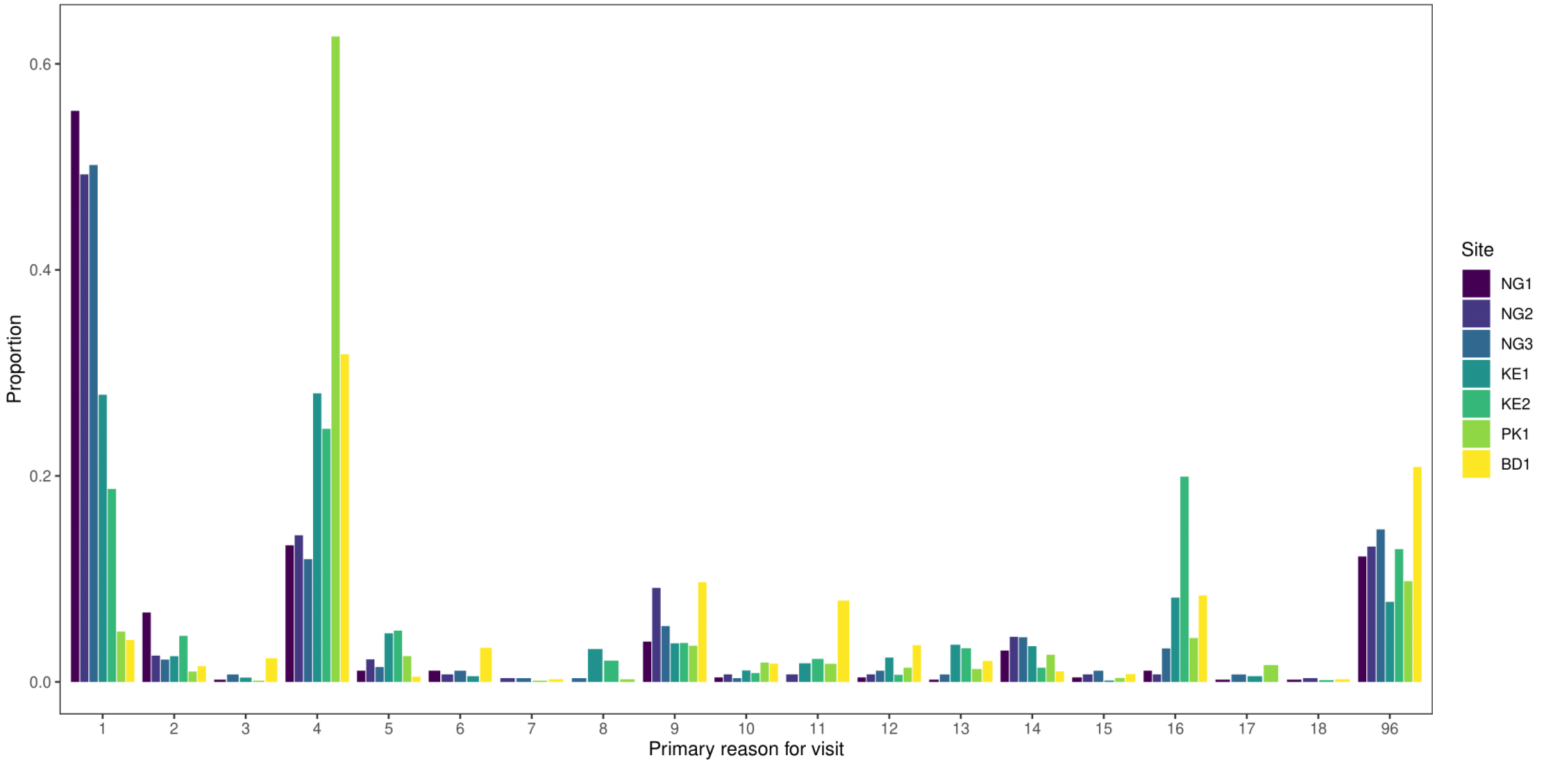

The reasons for visiting were: 1 Communicable disease (infections, malaria, tuberculosis, HIV), 2 Maternal and perinatal conditions (pregnancy), 3 Nutritional deficiencies, 4 Acute conditions (diarrhoea, fever, flu, headaches, cough, other), 5 Injury (not work related, see 8 below), 6 Surgery, 7 Sleep problems, 8 Occupation/work related condition/injury, 9 Chronic pain in your joints / arthritis (joints, back, neck), 10 Diabetes or related complications, 11 Problems with your heart, including unexplained pain in chest, 12 Problems with your mouth, teeth or swallowing, 13 Problems with your breathing, 14 High blood pressure / hypertension, 15 Stroke / sudden paralysis of one side of body, 16 Generalised pain (stomach, muscle or other non-specific pain), 17 Depression or anxiety, 18 Cancer, 96 Other (Specify)

Table C4 Percentage of respondents reporting reasons for choosing a healthcare providers by provider type

	Nigeria			Kenya		Pakistan	Bangladesh
	**NG1**	**NG2**	**NG3**	**KE1**	**KE2**	**PK1**	**BD1**
Nearness of the facility	63	69	57	78	72	61	54
Service providers are cordial	34	24	32	21	22	29	65
Good services available	38	32	44	31	37	37	95
Short waiting times	17	11	16	7	3	9	7
Qualified doctors are available	24	29	35	22	17	51	26
Low fees/treatment costs	28	33	43	36	24	36	39
Good waiting arrangement	12	9	15	3	2	7	5
Confidentiality is maintained	13	10	21	1	1	18	0
Don’t know where else to go	7	15	7	12	6	5	3
Medicines are available	21	17	25	35	22	9	4
Diagnostics are available	11	12	11	13	4	1	11
Recommendation	11	6	11	16	8	9	20

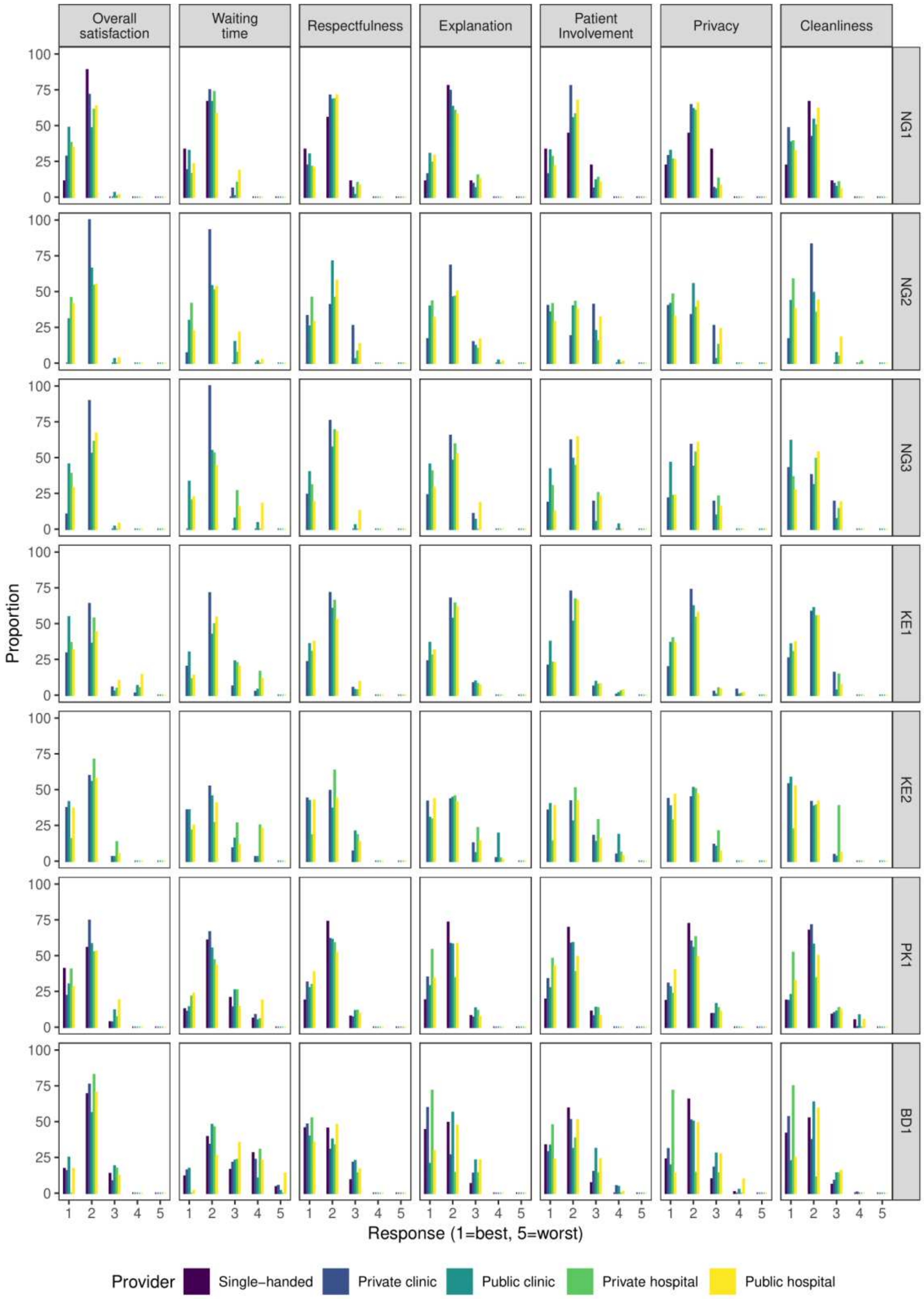

Figure C3. Satisfaction with outpatient consultations by type of facility and by site. Responses were on five-point Likert scales, either Very satisfied to very dissatisfied (Overall satisfaction) or Very good to very bad (Other questions).

REFERENCES

1 World Health Organization. Global Health Observatory. 2021.https://www.who.int/data/gho (accessed 6 Jun 2021).

2 J. Borah B. A mixed logit model of health care provider choice: analysis of NSS data for rural India. *Health Econ* 2006;**15**:915–32. doi:10.1002/hec.1166

3 Carpenter B, Gelman A, Hoffman M, *et al.* Stan: A Probabilistic Programming Language. *J Stat Softw* 2016;**VV**.
