# Supplementary material for: Primary care doctor and nurse consultations among people who live in slums: a retrospective, cross-sectional survey in four countries
Source: BMJ Open. 2022 Jan 7;12(1):e054142. doi: 10.1136/bmjopen-2021-054142 (PMC8744106; doi:10.1136/bmjopen-2021-054142)
Supplement: Supplementary data [file bmjopen-2021-054142supp001.pdf]

[Name of Country]
[Name of Slum]

Individual Questionnaire

[To be completed by the randomly selected adult (18+) from within the household. Note: first complete introduction and consent]

The next set of questions will ask you about the last time that you needed health care, whether or not you received that care. Please think back to the last time.

Module 4: Health Service Use

Q401	Have you needed health care in the last 12 months (whether or not you received health care)?<br><br>[INFORM THE RESPONDANT THAT THIS CAN BE INPATIENT CARE, OUTPATIENT CARE, MEDICATION, TRADITIONAL CARE, OR ANY OTHER INTERVENTION FOR THEIR HEALTH..]	1. Yes<br>2. No	Q402<br>Q443
Q402	The last time you needed health care, did you get health care?	1. Yes…………………………………………..-><br>2. No…………………………………………..->	Q403<br>Q402
Q402b<br><br>[ONLY IF 402 = “NO”]	Which reason(s) best explains why you did not get health care?<br><br>[CHOOSE ALL THAT THE RESPONDENT INDICATES]	1. Could not afford the cost of the visit<br>2. No transport available<br>3. Could not afford the cost of transport<br>4. You were previously badly treated<br>5. Could not take the time off work or had other commitments<br>6. The health care provider’s drugs or equipment were inadequate<br>7. The health care provider’s skills were inadequate<br>8. You did not know where to go<br>9. You tried but were denied health care<br>10. You thought you were not sick enough<br>97. Other, please specify	

Inpatient Hospital Care

The next two questions ask about any overnight stay in a hospital or other health care facility you had in the last year.

Q403	In the last 12 months, have you ever stayed overnight in a hospital or long-term health care facility?<br>[Select all that apply]	1. Yes, a hospital<br>2. Yes, long-term care facility<br>3. Yes, other provider (Please specify)<br><br>4. No ………………………………………………. →	 <br><br><br>Q423

. BMJ Open 2022; 12:e054142. doi: 10.1136/bmjopen-2021-054142

[Name of Country]
[Name of Slum]

Q404	Over the last 12 months, how many different times were you a patient in a hospital / long-term care facility for at least one night?	☐☐ times<br>98. Don't know	
		IF "00" (NO OVERNIGHT STAYS) .... →	Q423

I would like you to ask about your last inpatient stay only.

Q405	What type of hospital or facility was it? Remember we are asking now about your last (most recent) overnight stay.<br><br>[ONE ANSWER ONLY]	1. Public hospital<br>2. Private hospital<br>3. Charity or church-run hospital<br>4. Old person's home or long-term care facility<br>97. Other, please specify<br><br>[BANGLADESH: remove option 3, replace with "NGO clinical or health care facility and community health clinic"]	
Q405a	Please name the facility	Free text	
Q405b	Which reason(s) best describes why you chose this health care provider?<br>[CHOOSE ALL THAT THE RESPONDENT INDICATES]	1. Nearness of the facility<br>2. Service providers are cordial<br>3. Good service available<br>4. Short waiting time<br>5. Qualified doctors available<br>6. Low fees/low treatment cost<br>7. Good waiting arrangement<br>8. Confidentiality is maintained<br>9. Do not know where else to go<br>10. Medicine is also available<br>11. Availability of diagnostic service<br>12. Recommendation from someone<br>97. Other (please specify)	
Q405c	Which reason best describes why you were last hospitalised?<br><br>[RESPONDENT CAN SELECT ONLY ONE MAIN REASON FOR VISIT.]<br>1. Communicable disease (infections, malaria, tuberculosis, HIV)<br>2. Maternal and perinatal conditions (pregnancy)<br>3. Nutritional deficiencies<br>4. Acute conditions (diarrhoea, fever, flu, headaches, cough, other)<br>5. Injury (not work related, see 8 below)<br>6. Surgery<br>7. Sleep problems<br>8. Occupation/work related condition/injury<br>9. Chronic pain in your joints / arthritis (joints, back, neck)<br>10. Diabetes or related complications	11. Problems with your heart, including unexplained pain in chest<br>12. Problems with your mouth, teeth or swallowing<br>13. Problems with your breathing<br>14. High blood pressure / hypertension<br>15. Stroke / sudden paralysis of one side of body<br>16. Generalised pain (stomach, muscle or other non-specific pain)<br>17. Depression or anxiety<br>18. Cancer<br>97. Other, please specify	

2

[Name of Country]
[Name of Slum]

Q406	How did you get there?<br><br>[SELECT ALL THAT THE RESPONDENT MENTIONS]	1. Private vehicle<br>2. Public transportation<br>3. Taxicab<br>4. Ambulance or emergency vehicle<br>5. Bicycle<br>6. Walked<br>7. Boda boda, rickshaw, other private transport other than a vehicle<br>8. Don't know<br><br>[BANGLADESH: add option Rickshaw]	
Q406a	About how long did it take you to get there?	☐☐:☐☐ hours: minutes<br><br>98. Don't know	
Q406b	About how long did you wait between arrival and first consultation with a health professional?	☐☐:☐☐ hours: minutes<br><br>98. Don't know	
Q407	Who paid for this inpatient stay?<br><br>Anyone else?<br><br>[SELECT ALL RESPONSES.<br>PROBE TO SEE IF ANYONE ELSE PAID OR CONTRIBUTED TO PAYING FOR THE CARE?]	1. Respondent<br>2. Spouse / Partner<br>3. Son / Daughter<br>4. Other family member<br>5. Non-family member<br>6. Mandatory insurance scheme<br>7. Voluntary insurance scheme	
		8. Hospitalisation was free .... →	Q413
Q408	Thinking about you last inpatient stay, how much did you or your family / household members <u>pay out-of-pocket</u> for:<br><br>[WRITE "0" IF THE SERVICE WAS FREE – IF A PERSON DID NOT HAVE MEDICINES OR TESTS, ENTER 99990 FOR "NOT APPLICABLE, DID NOT HAVE."]<br><br>[USE LOCAL CURRENCY]	a. [Health care provider's] fees:<br>☐☐☐☐☐☐<br><br>b. Medicines:<br>☐☐☐☐☐☐<br><br>c. Tests:<br>☐☐☐☐☐☐<br><br>d. Transport:<br>☐☐☐☐☐☐<br><br>e. Other,<br>☐☐☐☐☐☐<br><br>please specify:	
Q409	About <u>how much in total</u> was paid <u>out-of-pocket</u> for this inpatient visit?<br><br>[USE LOCAL CURRENCY]	☐☐☐☐☐☐☐☐☐	

[Name of Country]
[Name of Slum]

Q410	Overall, how satisfied were you with the care you received during your last overnight stay?	1. Very satisfied<br>2. Satisfied<br>3. Neither satisfied nor dissatisfied<br>4. Dissatisfied<br>5. Very dissatisfied				
For your last overnight visit to a hospital or long-term care facility, how would you rate the following:		Very good	Good	Moderate	Bad	Very bad
Q411	… the amount of time you waited before being attended to?	1	2	3	4	5
Q412	… your experience of being treated respectfully?	1	2	3	4	5
Q413	… how clearly health care providers explained things to you?	1	2	3	4	5
Q414	… your experience of being involved in making decisions for your treatment?	1	2	3	4	5
Q415	… the way the health services ensured that you could talk privately to providers?	1	2	3	4	5
Q416	… the ease with which you could see a health care provider you were happy with?	1	2	3	4	5
Q417	… the cleanliness in the health facility?	1	2	3	4	5

Outpatient Care

Now I will shift away from questions about overnight stays – to questions about health care you received that did not include an overnight hospital stay. The following questions are about care you received at a hospital, health centre, clinic, pharmacy, or private office including traditional health care but where you did not stay overnight.

Q418	Over the last 12 months did you receive any health care NOT including an overnight stay?	1. Yes	
		2. No …………………………………………… →	Q443
Q419	In total, how many times did you receive health care or consultation in the last 12 months?	☐☐ times	

Q420	What was the last (most recent) health care facility you visited in the last 12 months?<br><br>[READ OUT RESPONSES, SELECT ONE OPTION ONLY.]	1. Private doctor's office<br>2. Private clinic or health care facility<br>3. Private hospital<br>4. Public clinic or health care facility<br>5. Public hospital<br>6. Charity or church run clinic<br>7. Charity or church run hospital<br>8. Traditional Healer [USE LOCAL TERMS]<br>9. Pharmacy<br>97. Other, please specify:<br><br>[BANGLADESH: remove options 6 and 7, replace with "NGO clinic or health care facility and community health clinic"]	
Q420a	Please name the provider	Free text	

4

[Name of Country]
[Name of Slum]

Q420b	Which reason(s) best describes why you chose this health care provider?<br>[CHOOSE ALL THAT THE RESPONDENT INDICATES]	1. Nearness of the facility<br>2. Service providers are cordial<br>3. Good service available<br>4. Short waiting time<br>5. Qualified doctors available<br>6. Low fees/low treatment cost<br>7. Good waiting arrangement<br>8. Confidentiality is maintained<br>9. Do not know where else to go<br>10. Medicine is also available<br>11. Availability of diagnostic service<br>12. Recommendation from someone<br>97. Other (please vspecify)	
Q421	Which was the last (most recent) health care provider you visited?<br><br>[THE PERSON WHO PROVIDED THE CARE FOR THE MAIN REASON FOR THE VISIT]<br><br>[AFTER Q426 SUBSTITUTE THE TYPE OF HEALTH CARE PROVIDER SELECTED BY THE PATIENT WHEN YOU SEE [HEALTH CARE PROVIDER] IN BRACKETS.]	1. Medical doctor (including surgeon, gynecologist, psychiatrist, ophthalmologist…)<br>2. Nurse<br>3. Midwife<br>4. Dentist<br>5. Physiotherapist or chiropractor<br>6. Traditional medicine practitioner [USE LOCAL NAME]<br>7. Pharmacist, druggist<br>8. Don't know	
Q421a	What was the sex of the [HEALTH CARE PROVIDER]?	1. Male<br>2. Female	
Q421b	Was this visit to [HEALTH CARE PROVIDER] for a chronic (ongoing) condition, new condition, both, or routine check-up?	1. Chronic<br>2. New<br>3. Both<br>4. Routine check-up	
Q421c	Which reason best describes why you needed this visit?<br><br>[RESPONDENT CAN SELECT ONLY ONE MAIN REASON FOR VISIT.		
	1. Communicable disease (infections, malaria, tuberculosis, HIV)<br>2. Maternal and perinatal conditions (pregnancy)<br>3. Nutritional deficiencies<br>4. Acute conditions (diarrhoea, fever, flu, headaches, cough, other)<br>5. Injury (not work related, see 8 below)<br>6. Surgery<br>7. Sleep problems<br>8. Occupation/work related condition/injury<br>9. Chronic pain in your joints / arthritis (joints, back, neck)<br>10. Diabetes or related complications	11. Problems with your heart, including unexplained pain in chest<br>12. Problems with your mouth, teeth or swallowing<br>13. Problems with your breathing<br>14. High blood pressure / hypertension<br>15. Stroke / sudden paralysis of one side of body<br>16. Generalised pain (stomach, muscle or other non-specific pain)<br>17. Depression or anxiety<br>18. Cancer<br>97. Other, please specify	

[Name of Country]
[Name of Slum]

Q422	Thinking about your last visit, how did you get there?<br><br>[CIRCLE ALL THAT THE RESPONDENT MENTIONS.]	1. Private vehicle<br>2. Public transportation<br>3. Taxicab<br>4. Ambulance or emergency vehicle<br>5. Bicycle<br>6. Walked<br>8. Don't know<br><br>[BANGLADESH: add option Rickshaw]	
Q423	About how long did it take you to get there?	☐☐:☐☐ hours: minutes<br><br>98. Don't know	
Q423a	About how long did you wait between arrival and first consultation with a health professional?	☐☐:☐☐ hours: minutes<br><br>98. Don't know	
Q424	Who paid for this most recent visit?<br><br>Anyone else?<br><br>[SELECT ALL RESPONSES.<br>PROBE TO SEE IF ANYONE ELSE PAID OR CONTRIBUTED TO PAYING FOR THE CARE?]	1. Respondent<br>2. Spouse / Partner<br>3. Son / Daughter<br>4. Other family member<br>5. Non-family member<br>6. Mandatory insurance scheme<br>7. Voluntary insurance scheme	
		8. It was free .... →	Q431
Q425	Thinking about your last visit, how much did you or your family / household members pay for:<br><br>[WRITE "0" IF THE SERVICE WAS FREE – IF A PERSON DID NOT HAVE MEDICINES OR TESTS, ENTER 99998 FOR "NOT APPLICABLE, DID NOT HAVE."]<br><br>[USE LOCAL CURRENCY]	a. [HEALTH CARE PROVIDER'S] fees:<br>☐☐☐☐☐☐<br><br>b. Medicines:<br>☐☐☐☐☐☐<br><br>c. Tests:<br>☐☐☐☐☐☐<br><br>d. Transport:<br>☐☐☐☐☐☐<br><br>e. Other,<br>☐☐☐☐☐☐<br><br>please specify:	
		f. Total costs:<br>☐☐☐☐☐☐☐☐☐	

[Name of Country]
[Name of Slum]

Q426	Overall, how <u>satisfied</u> were you with the care you received during your last visit?	1. Very satisfied<br>2. Satisfied<br>3. Neither satisfied nor dissatisfied<br>4. Dissatisfied<br>5. Very dissatisfied	

For your <u>last visit</u> to a <u>health care provider</u>, how would you rate the following:		Very good	Good	Moderate	Bad	Very bad
Q427	… the amount of time you <u>waited</u> before being attended to?	1	2	3	4	5
Q428	… your experience of <u>being treated respectfully</u>?	1	2	3	4	5

For your <u>last visit</u> to a <u>health care provider</u>, how would you rate the following:		*Very good*	*Good*	*Moderate*	*Bad*	*Very bad*
Q429	… how <u>clearly</u> health care providers explained things to you?	1	2	3	4	5
Q430	… your experience of being <u>involved in making decisions</u> for your treatment?	1	2	3	4	5
Q431	… the way the health services ensured that you could <u>talk privately</u> to providers?	1	2	3	4	5
Q432	… the <u>ease</u> with which you could see a health care provider you were happy with?	1	2	3	4	5
Q433	… the <u>cleanliness</u> in the health facility?	1	2	3	4	5

Care at home

Now I would like to ask you to think about care you received from a health care worker or provider while at home, place of work or at an education centre. For the following questions please think about health care accessed in the past year only.

Q434	Over the last 12 months, have you received a visit in your home or place of work or education from any health care worker or provider?	1. Yes<br>2. No…………………………………… →<br>8. Don't know……………………….→	 Q444<br>Q444
Q434a	What was discussed with the health care worker?<br><br>[SELECT ALL THAT APPLY]	1. Family planning<br>2. Healthy life-style<br>3. Advice for common illness<br>4. Vaccines for pregnant women and children<br>5. Diet and nutrition<br>6. Other (specify _____________)<br>8. Don't know	
Q434b	What, if any, materials were distributed by the health care worker?	1. Family planning materials<br>2. De-worming tablets<br>3. Medicine for common cold or fever<br>4. Vitamin(s)<br>5. Calcium<br>6. Iron tablet(s)<br>7. Other (specify _____________)	

. *BMJ Open* 2022; 12:e054142. doi: 10.1136/bmjopen-2021-054142

[Name of Country]
[Name of Slum]

		8. Don't know	

M-health

Q435	In the last 12 months have you used or attempted to use your mobile phone or other digital communication device (e.g.: laptop, tablet) to access health information, advice or care for yourself, where information about your health was received or given?	1. Yes<br>2. No……………………………………… →	Q472
Q436	Which ways of accessing health advice or information have you used?<br><br>[SELECT ALL THAT APPLY]	1. Contacted someone (via text message, chat website, e-mail, video call etc.)……………………-><br>2. Researched health information without speaking to someone (via Google, health website etc.)……………………………….->	
Q437	Have you had any problems when using your mobile phone or other device for this purpose?	1. Yes<br>2. No ……………………………………… →	Q448
Q437a	What were the problem(s):<br><br>[SELECT ALL THAT THE RESPONDENT INDICATES]	1. No airtime/data/wifi<br>2. Poor connectivity/signal<br>3. Device not sufficiently charged<br>4. No response from the person/organisation contacted<br>5. Unable to read the textual message received<br>6. Unable to find the information needed on web/social media<br>7. Unable to read the relevant text found<br>97. Other	

Now I would like to ask you to think about the last time you used or attempted to use your mobile phone or other digital communication device to access health information, advice or care for yourself, where information about your health was received or given. For the following questions please think about the most recent time only.

Q438	Which ways of accessing health or health care information did you use?<br><br>[SELECT ALL THAT APPLY.]	1. Contacted someone (via text message, chat website, e-mail, video call etc.)……………………-><br>2. Researched health information without speaking to someone (via Google, health website etc.)……………………………….->	Q450<br><br>Q451
Q439	Who did you receive this information or what service did you use to get this information?<br><br>[REMEMBER WE ARE ASKING NOW ABOUT YOUR LAST (MOST RECENT) USE OF A DIGITAL CHANNEL FOR SEEKING HEALTH INFORMATION, ADVICE OR CARE.]	1. Medical Doctor<br>2. Nurse<br>3. Midwife<br>4. Dentist<br>5. Physiotherapist or chiropractor<br>6. Traditional medicine practitioner<br>7. Pharmacist, druggist<br>8. Users and/or provider of health related app.	
Q440	Please provide name of person/organisation/website/social media site	[Free text]	

[Name of Country]
[Name of Slum]

	and phone number or web address/e-mail address		
Q441	Why did you choose to get health information this way?<br><br>[QUESTION REFERS TO WHY THE RESPONDENT CHOSE TO USE M-HEALTH RATHER THAN SEEING A PROVIDER IN PERSON]<br><br>[CHOOSE ALL THAT THE RESPONDENT INDICATES]	1. I could seek the health information, advice or care at a time I chose<br>2. I could seek the health information, advice or care in a place I chose<br>3. The problem was urgent<br>4. The problem was not urgent<br>5. Service providers are cordial<br>6. I would not have to wait at a facility<br>7. I trust the provider<br>8. I needed more information than I usually receive from a health worker<br>9. I had no money to get to see a health worker<br>10. It was cheaper than going to see a health worker<br>11. I had more privacy during the communication than when seeing a health worker<br>12. I did not want other people to know I was seeking health, information advice or care<br>13. This was the only option that was available at that time	
Q442	Which reason best describes why you needed this contact?<br><br>[RESPONDENT CAN SELECT ONLY ONE MAIN REASON FOR VISIT.		
	1. Communicable disease (infections, malaria, tuberculosis, HIV)<br>2. Maternal and perinatal conditions (pregnancy)<br>3. Nutritional deficiencies<br>4. Acute conditions (diarrhoea, fever, flu, headaches, cough, other)<br>5. Injury (not work related, see 8 below)<br>6. Surgery<br>7. Sleep problems<br>8. Occupation/work related condition/injury<br>9. Chronic pain in your joints / arthritis (joints, back, neck)<br>10. Diabetes or related complications	11. Problems with your heart, including unexplained pain in chest<br>12. Problems with your mouth, teeth or swallowing<br>13. Problems with your breathing<br>14. High blood pressure / hypertension<br>15. Stroke / sudden paralysis of one side of body<br>16. Generalised pain (stomach, muscle or other non-specific pain)<br>17. Depression or anxiety<br>18. Cancer<br>97. Other, please specify	
Q443	Was there a cost associated with accessing this health advice or information?	1. Yes<br>2. No…………………………………. →	Q458
Q444	Thinking about the last time you last accessed health advice or information, how much did you or your family / household members pay (Do not include cost of airtime/data):	[ ][ ][ ][ ]<br><br>8. Don't know	
Q445	Overall, how <u>satisfied</u> were you with the information, advice or care you received?	1. Very satisfied<br>2. Satisfied<br>3. Neither satisfied nor dissatisfied<br>4. Dissatisfied<br>5. Very dissatisfied	

9

[Name of Country]
[Name of Slum]

Q446	Did you seek information, advice or care about the same problem from a health worker face-to-face after this digital contact?	1. Yes<br>2. No	

Now I would like you to think about your most recent contact again. I want to know your impression of your most recent contact for health information, advice or care. I would like you to rate your experiences using the following questions:

For the last time you accessed health advice or information with a phone, computer, or other electronic device, , how would you rate the following:		Very good	Good	Moderate	Bad	Very bad
FOR RESPONDENTS WHO HAVE REPORTED A CONTACTING SOMEONE						
Q447	… your experience of <u>being treated respectfully</u>?	1	2	3	4	5
Q448	… how <u>clearly</u> health care providers explained things to you?	1	2	3	4	5
Q449	… the <u>ease</u> with which you could make contact with a health care provider you were happy with?	1	2	3	4	5
FOR RESPONDENTS WHO HAVE REPORTED RESEARCHING HEALTH INFO						
Q450	… how understandable was the information or advice?	1	2	3	4	5

Unpaid care

Now I would like to ask you to think about the last time you provided/receive care from someone for a long-term physical or mental illness or disability or for getting old and weak. For the following questions please think about care provided or received in the past year only.

Q451	During the past year, did you provide help to a relative or friend (adult or child), because this person has a long-term physical or mental illness or disability, or is getting old and weak?	1. Yes, for a person living in the same household<br>2. Yes, for a person living in a separate household<br>3. No…………………………………………. →	Q473
Q451a	Please tell me the kind of care provided:<br><br>[PLEASE SELECT ALL THAT APPLY]	1. You helped with personal care, such as going to the toilet, washing, getting dressed, or eating<br>2. You helped with medical care, like changing bandages, and giving medicines<br>3. You helped with household activities, such as meal preparation, shopping, cleaning, laundry<br>4. You watched over them since their behaviour can be upsetting or dangerous to themselves or others	
Q451b	Is/was this one-off or regular care?	1. One-off<br>2. Regular	
Q451c	Approximately how many hours a week do/did you spend providing this help?	☐☐☐☐ hours per week	

. BMJ Open 2022; 12:e054142. doi: 10.1136/bmjopen-2021-054142

[Name of Country]
[Name of Slum]

Q452	During the past year, did you receive help from a relative or friend (adult or child) because you have a long-term physical or mental illness or disability or are getting old and weak?	1. Yes, from a person living in the same household<br>2. Yes, from a person living in a separate household<br>3. No………………………………………. →	Q474
Q452a	Please tell me the kind of care provided:<br><br>[PLEASE SELECT ALL THAT APPLY]	1. You were helped with personal care, such as going to the toilet, washing, getting dressed, or eating<br>2. You were helped with medical care, like changing bandages, and giving medicines<br>3. You were helped with household activities, such as meal preparation, shopping, cleaning, laundry<br>4. You were watched over since your behaviour can be upsetting or dangerous to yourself or others	
Q452b	Is/was this one-off or regular care?	1. One-off<br>2. Regular	
Q452c	Approximately how many hours a week do you receive this help?	☐☐☐☐ hours per week	

[Name of Country]
[Name of Slum]

Module 5: Health Status and Wellbeing

We will now as you a series of questions about your health and well-being. Please try to answer as honestly and accurately as possible. All responses will be kept in the strictest confidence.

Q501	In general would you say your health is:	1. Excellent<br>2. Very good<br>3. Good<br>4. Fair<br>5. Poor
Q502	Compared to one year ago, how would you rate your health in general now?	1. Much better than one year ago<br>2. Somewhat better now than one year ago<br>3. About the same<br>4. Somewhat worse now that one year ago<br>5. Much worse now than one year ago

Please select the response that best describes you

Q503	Physical functioning	1. Your health does not limit you in vigorous activities (such as running, lifting heavy objects)<br>2. Your health limits you a little in vigorous activities (such as running, lifting heavy objects)<br>3. Your health limits you a little in moderate activities (such as moving a table, playing with children)<br>4. Your health limits you a lot in moderate activities (such as moving a table, playing with children)<br>5. Your health limits you a little in bathing and dressing<br>6. Your health limits you a lot in bathing and dressing	
Q504	Role limitations	1. You have no problems with your work or other regular daily activities as a result of your physical health or emotional problems<br>2. You are limited in the kind of work or other activities as a result of your physical health<br>3. You accomplish less than you would like as a result of emotional problems<br>4. You are limited in the kind of work or other activities as a result of you physical health and accomplish less than you would like as a result of emotional problems	
Q505	Social functioning	Your health limits your social activities (like visiting friends, relatives, etc.):<br>1. none of the time<br>2. a little of the time<br>3. some of the time<br>4. most of the time<br>5. all of the time	
Q506	Pain	1. You have no pain<br>2. You have pain but it does not interfere with your normal work (both outside the home and housework)<br>3. You have pain that interferes with your normal work (both outside the home and housework) a little bit<br>4. You have pain that interferes with your normal work (both outside the home and housework) moderately<br>5. You have pain that interferes with your normal work (both outside the home and housework) quite a bit<br>6. You have pain that interferes with your normal work (both outside the home and housework) extremely	

12

[Name of Country]
[Name of Slum]

Q507	Mental health<br>You feel tense or downhearted and low:	1. None of the time<br>2. A little of the time<br>3. Some of the time<br>4. Most of the time<br>5. All of the time	
Q508	Vitality<br>You have a lot of energy:	1. None of the time<br>2. A little of the time<br>3. Some of the time<br>4. Most of the time<br>5. All of the time	

The following question asks how satisfied you feel, on a scale from 0 to 10. Zero means you feel 'not at all satisfied', and 10 means you feel 'completely satisfied'.

Q509	Overall, how satisfied are you with life as a whole these days? [0-10]	☐☐

The following question asks how worthwhile you feel the things you do in your life are, on a scale from 0 to 10. Zero means you feel the things you do in your life are 'not at all worthwhile', and 10 means 'completely worthwhile'.

Q510	Overall, to what extent do you feel the things you do in your life are worthwhile? [0-10]	☐☐

The following questions ask about how you felt yesterday on a scale from 0 to 10. Zero means you did not experience the feeling 'at all' yesterday, while 10 means you experienced the feeling 'all of the time' yesterday.

I will now read out a list of ways you might have felt yesterday.

Q511	How about happy? [0-10]	☐☐
Q512	How about worried? [0-10]	☐☐
Q513	How about depressed? [0-10]	☐☐
