# Supplementary material for: Primary care doctor and nurse consultations among people who live in slums: a retrospective, cross-sectional survey in four countries
Source: BMJ Open. 2022 Jan 7;12(1):e054142. doi: 10.1136/bmjopen-2021-054142 (PMC8744106; doi:10.1136/bmjopen-2021-054142)
Supplement: Supplementary data [file bmjopen-2021-054142supp002.pdf]

[Name of Country]
[Name of Slum]

Individual (child) Questionnaire

[THE PERSON COMPLETING THE QUESTIONNAIRE SHOULD BE THE RESPONSIBLE ADULT FOR THE RANDOMLY SELECTED CHILD]

[The child's name is referred to as {NAME}]

The next set of questions will ask you about the last time that you needed health care, whether or not you received that care. Please think back to the last time.

Module 4: Health Service Use

Q401	Has {NAME} needed health care in the last 12 months (whether or not {NAME} received health care)?<br><br>[INFORM THE RESPONDANT THAT THIS CAN BE INPATIENT CARE, OUTPATIENT CARE, MEDICATION, TRADITIONAL CARE, OR ANY OTHER INTERVENTION FOR THEIR HEALTH	1. Yes<br>2. No	Q402<br>Q443
Q402	The last time {NAME} needed health care, did {NAME} get health care?	1. Yes<br>2. No	
Q402b<br><br>[ONLY IF 402 = "NO"]	Which reason(s) best explains why {NAME} did not get health care?<br><br>[CHOOSE ALL THAT THE RESPONDENT INDICATES]	1. Could not afford the cost of the visit<br>2. No transport available<br>3. Could not afford the cost of transport<br>4. You or your child were previously badly treated<br>5. Could not take the time off work or had other commitments<br>6. The health care provider's drugs or equipment were inadequate<br>7. The health care provider's skills were inadequate<br>8. You did not know where to go<br>9. You tried but were denied health care<br>10. You thought you or your child were not sick enough<br>97. Other, please specify	

Inpatient Hospital Care

The next two questions ask about any overnight stay in a hospital or other health care facility {NAME} had in the last year.

. BMJ Open 2022; 12:e054142. doi: 10.1136/bmjopen-2021-054142

[Name of Country]
[Name of Slum]

Q403	In the last 12 months, has {NAME} ever stayed overnight in a hospital, long-term care facility, or other provider?<br>[Select all that apply]	1. Yes, a hospital<br>2. Yes, long-term care facility<br>3. Yes, other provider (Please specify)	
		4. No ……………………………………………… →	Q423

Q404	Over the last 12 months, how many different times was {NAME} a patient in a hospital / long-term care facility for at least one night?	☐☐ times<br>98. Don't know	
		IF "00" (NO OVERNIGHT STAYS) ……………… →	Q423

I would like you to ask about {NAME}'s last inpatient stay only.

Q405	What type of hospital or facility was it? Remember we are asking now about {NAME}'s last (most recent) overnight stay.<br><br>[ONE ANSWER ONLY]	1. Public hospital<br>2. Private hospital<br>3. Charity or church-run hospital<br>4. Old person's home or long-term care facility<br>97. Other, please specify<br><br>[BANGLADESH: remove option 3, replace with "NGO clinical or health care facility and community health clinic"]	
Q405a	Please name the facility	Free text	
Q405b	Which reason(s) best describes why you chose this health care provider?<br>[CHOOSE ALL THAT THE RESPONDENT INDICATES]	1. Nearness of the facility<br>2. Service providers are cordial<br>3. Good service available<br>4. Short waiting time<br>5. Qualified doctors available<br>6. Low fees/low treatment cost<br>7. Good waiting arrangement<br>8. Confidentiality is maintained<br>9. Do not know where else to go<br>10. Medicine is also available<br>11. Availability of diagnostic service<br>12. Recommendation from someone<br>97. Other (please specify)	
Q405c	Which reason best describes why {NAME} was last hospitalised?<br><br>[RESPONDENT CAN SELECT ONLY ONE MAIN REASON FOR VISIT. USE SHOWCARD IN APPENDIX.]		

[Name of Country]
[Name of Slum]

	1. Communicable disease (infections, malaria, tuberculosis, HIV)<br>2. Maternal and perinatal conditions (pregnancy)<br>3. Nutritional deficiencies<br>4. Acute conditions (diarrhoea, fever, flu, headaches, cough, other)<br>5. Injury (not work related, see 8 below)<br>6. Surgery<br>7. Sleep problems<br>8. Occupation/work related condition/injury<br>9. Chronic pain in your joints / arthritis (joints, back, neck)<br>10. Diabetes or related complications	11. Problems with your heart, including unexplained pain in chest<br>12. Problems with your mouth, teeth or swallowing<br>13. Problems with your breathing<br>14. High blood pressure / hypertension<br>15. Stroke / sudden paralysis of one side of body<br>16. Generalised pain (stomach, muscle or other non-specific pain)<br>17. Depression or anxiety<br>18. Cancer<br>97. Other, please specify	
Q406	How did you get there?<br><br>[CIRCLE ALL THAT THE RESPONDENT MENTIONS]	1. Private vehicle<br>2. Public transportation<br>3. Taxicab<br>4. Ambulance or emergency vehicle<br>5. Bicycle<br>6. Walked<br>7. Boda boda, rickshaw, other private transport other than a vehicle<br>8. Don't know<br><br>[BANGLADESH: add option Rickshaw]	
Q406a	About how long did it take you to get there?	☐☐:☐☐ hours: minutes<br><br>98. Don't know	
Q406b	About how long did you wait between arrival and first consultation with a health professional?	☐☐:☐☐ hours: minutes<br><br>98. Don't know	
Q407	Who paid for this inpatient stay?<br><br>Anyone else?<br><br>[CIRCLE ALL RESPONSES.<br>PROBE TO SEE IF ANYONE ELSE PAID OR CONTRIBUTED TO PAYING FOR THE CARE?]	1. Respondent<br>2. Spouse / Partner<br>3. Son / Daughter<br>4. Other family member<br>5. Non-family member<br>6. Mandatory insurance scheme<br>7. Voluntary insurance scheme<br>8. Hospitalisation was free .... →	 <br><br><br><br><br><br>Q413
Q408	Thinking about {NAME}'s last inpatient stay, how much did you or your family / household members pay out-of-pocket for:<br><br>[WRITE "0" IF THE SERVICE WAS FREE – IF A PERSON DID NOT HAVE MEDICINES OR TESTS, ENTER 99990 FOR "NOT APPLICABLE, DID NOT HAVE."]<br><br>[USE LOCAL CURRENCY]	a. [Health care provider's] fees:<br>☐☐☐☐☐☐<br><br>b. Medicines:<br>☐☐☐☐☐☐<br><br>c. Tests:<br>☐☐☐☐☐☐	

3

[Name of Country]
[Name of Slum]

		d. Transport: ☐☐☐☐☐☐<br><br>e. Other, ☐☐☐☐☐☐<br><br>please specify:	
Q409	About how much in total was paid out-of-pocket for this inpatient visit?<br><br>[USE LOCAL CURRENCY]	☐☐☐☐☐☐☐☐☐	
Q410	Overall, how satisfied were you with the care {NAME} received during his/her last [hospital] stay?	1. Very satisfied<br>2. Satisfied<br>3. Neither satisfied nor dissatisfied<br>4. Dissatisfied<br>5. Very dissatisfied	

For {NAME}'s last overnight visit to a hospital or long-term care facility, how would you rate the following:		Very good	Good	Moderate	Bad	Very bad
Q411	… the amount of time you waited before being attended to?	1	2	3	4	5
Q412	… your experience of being treated respectfully?	1	2	3	4	5
Q413	… how clearly health care providers explained things to you?	1	2	3	4	5
Q414	… your experience of being involved in making decisions for {NAME}'s treatment?	1	2	3	4	5
Q415	… the way the health services ensured that you could talk privately to providers?	1	2	3	4	5
Q416	… the ease with which you could see a health care provider you were happy with?	1	2	3	4	5
Q417	… the cleanliness in the health facility?	1	2	3	4	5

Outpatient Care

Now I will shift away from questions about overnight stays – to questions about health care {NAME} received that did not include an overnight hospital stay. The following questions are about care {NAME} received at a hospital, health centre, clinic, pharmacy, or private office including traditional health care but where {NAME} did not stay overnight.

Q418	Over the last 12 months did {NAME} receive any health care NOT including an overnight stay?	1. Yes<br>2. No ……………………………………………… →	 Q443
Q419	In total, how many times did {NAME} receive health care or consultation in the last 12 months?	☐☐ times	

Now I would like you to think about the most recent visit – and ask you specifically about {NAME}'s last or most recent visit:

[Name of Country]
[Name of Slum]

Q420	What was the last (most recent) health care facility {NAME} visited in the last 12 months?<br><br>[READ OUT RESPONSES, CHOOSE ONE OPTION ONLY.]	1. Private doctor's office<br>2. Private clinic or health care facility<br>3. Private hospital<br>4. Public clinic or health care facility<br>5. Public hospital<br>6. Charity or church run clinic<br>7. Charity or church run hospital<br>8. Traditional Healer [USE LOCAL TERMS]<br>9. Pharmacy<br>97. Other, please specify:<br><br>[BANGLADESH: remove options 6 and 7, replace with "NGO clinic or health care facility and community health clinic"]	
Q420a	Please name the provider	Free text	
Q420b	Which reason(s) best describes why you chose this health care provider?<br>[CHOOSE ALL THAT THE RESPONDENT INDICATES]	1. Nearness of the facility<br>2. Service providers are cordial<br>3. Good service available<br>4. Short waiting time<br>5. Qualified doctors available<br>6. Low fees/low treatment cost<br>7. Good waiting arrangement<br>8. Confidentiality is maintained<br>9. Do not know where else to go<br>10. Medicine is also available<br>11. Availability of diagnostic service<br>12. Recommendation from someone<br>97. Other (please specify)	
Q421	Which was the last (most recent) health care provider {NAME} visited?<br><br>[THE PERSON WHO PROVIDED THE CARE FOR THE MAIN REASON FOR THE VISIT]<br><br>[AFTER Q426 SUBSTITUTE THE TYPE OF HEALTH CARE PROVIDER SELECTED BY THE PATIENT WHEN YOU SEE [HEALTH CARE PROVIDER] IN BRACKETS.]	1. Medical doctor (including surgeon, gynecologist, psychiatrist, ophthalmologist…)<br>2. Nurse / midwife<br>3. Dentist<br>4. Physiotherapist or chiropractor<br>5. Traditional medicine practitioner [USE LOCAL NAME]<br>6. Pharmacist, druggist<br>8. Don't know	
Q421a	What was the sex of the [HEALTH CARE PROVIDER]?	1. Male<br>2. Female	
Q421b	Was this visit to [HEALTH CARE PROVIDER] for a chronic (ongoing) condition, new condition, both, or routine check-up?	1. Chronic<br>2. New<br>3. Both<br>4. Routine check-up	
Q421c	Which reason best describes why {NAME} needed this visit?<br><br>[RESPONDENT CAN SELECT ONLY ONE MAIN REASON FOR VISIT.		

[Name of Country]
[Name of Slum]

	1. Communicable disease (infections, malaria, tuberculosis, HIV)<br>2. Maternal and perinatal conditions (pregnancy)<br>3. Nutritional deficiencies<br>4. Acute conditions (diarrhoea, fever, flu, headaches, cough, other)<br>5. Injury (not work related, see 8 below)<br>6. Surgery<br>7. Sleep problems<br>8. Occupation/work related condition/injury<br>9. Chronic pain in your joints / arthritis (joints, back, neck)<br>10. Diabetes or related complications	11. Problems with your heart, including unexplained pain in chest<br>12. Problems with your mouth, teeth or swallowing<br>13. Problems with your breathing<br>14. High blood pressure / hypertension<br>15. Stroke / sudden paralysis of one side of body<br>16. Generalised pain (stomach, muscle or other non-specific pain)<br>17. Depression or anxiety<br>18. Cancer<br>97. Other, please specify	
Q422	Thinking about {NAME}'s last visit, how did you get there?<br><br>[CIRCLE ALL THAT THE RESPONDENT MENTIONS.]	1. Private vehicle<br>2. Public transportation<br>3. Taxicab<br>4. Ambulance or emergency vehicle<br>5. Bicycle<br>6. Walked<br>8. Don't know<br><br>[BANGLADESH: add option Rickshaw]	
Q423	About how long did it take you to get there?	☐☐:☐☐ hours: minutes<br><br>98. Don't know	
Q423a	About how long did you wait between arrival and first consultation with a health professional?	☐☐:☐☐ hours: minutes<br><br>98. Don't know	
Q424	Who paid for this most recent visit?<br><br>Anyone else?<br><br>[SELECT ALL RESPONSES.<br>PROBE TO SEE IF ANYONE ELSE PAID OR CONTRIBUTED TO PAYING FOR THE CARE?]	1. Respondent<br>2. Spouse / Partner<br>3. Son / Daughter<br>4. Other family member<br>5. Non-family member<br>6. Mandatory insurance scheme<br>7. Voluntary insurance scheme<br>8. It was free .... →	 <br><br><br><br><br><br>Q431

[Name of Country]
[Name of Slum]

Q425	Thinking about {NAME}'s last visit, how much did you or your family / household members pay for:<br><br>[WRITE "0" IF THE SERVICE WAS FREE – IF A PERSON DID NOT HAVE MEDICINES OR TESTS, ENTER 99998 FOR "NOT APPLICABLE, DID NOT HAVE."]<br><br>[USE LOCAL CURRENCY]	a. [HEALTH CARE PROVIDER'S] fees: ☐☐☐☐☐☐<br>b. Medicines: ☐☐☐☐☐☐<br>c. Tests: ☐☐☐☐☐☐<br>d. Transport: ☐☐☐☐☐☐<br>e. Other, ☐☐☐☐☐☐<br>please specify:	
		f. Total costs: ☐☐☐☐☐☐☐☐☐	
Q426	Overall, how satisfied were you with the care {NAME} received during your last visit?	1. Very satisfied<br>2. Satisfied<br>3. Neither satisfied nor dissatisfied<br>4. Dissatisfied<br>5. Very dissatisfied	

I want to know your impression of your most recent visit for health care. I would like you to rate your experiences using the following questions:

For {NAME}'s last visit to a health care provider, how would you rate the following:		Very good	Good	Moderate	Bad	Very bad
Q427	… the amount of time you waited before being attended to?	1	2	3	4	5
Q428	… your experience of being treated respectfully?	1	2	3	4	5

For {NAME}'s last visit to a health care provider, how would you rate the following:		*Very good*	*Good*	*Moderate*	*Bad*	*Very bad*
Q429	… how clearly health care providers explained things to you?	1	2	3	4	5
Q430	… your experience of being involved in making decisions for {NAME}'s treatment?	1	2	3	4	5
Q431	… the way the health services ensured that you could talk privately to providers?	1	2	3	4	5
Q432	… the ease with which you could see a health care provider you were happy with?	1	2	3	4	5
Q433	… the cleanliness in the health facility?	1	2	3	4	5

[Name of Country]
[Name of Slum]

Q501	In general would you say {NAME}'s health is:	1. Excellent<br>2. Very good<br>3. Good<br>4. Fair<br>5. Poor
Q502	Compared to one year ago, how would you rate {NAME}'s health in general now?	1. Much better than one year ago<br>2. Somewhat better now than one year ago<br>3. About the same<br>4. Somewhat worse now that one year ago<br>5. Much worse now than one year ago

. *BMJ Open* 2022; 12:e054142. doi: 10.1136/bmjopen-2021-054142
