# Supplementary material for: Primary care doctor and nurse consultations among people who live in slums: a retrospective, cross-sectional survey in four countries
Source: BMJ Open. 2022 Jan 7;12(1):e054142. doi: 10.1136/bmjopen-2021-054142 (PMC8744106; doi:10.1136/bmjopen-2021-054142)
Supplement: Supplementary data [file bmjopen-2021-054142supp004.pdf]

[Name of Country]
[Name of Slum]

Household Questionnaire

[THE PERSON COMPLETING THE HOUSEHOLD QUESTIONNAIRE SHOULD BE THE HEAD OF HOUSEHOLD IF AVAILABLE. WHERE UNAVAILABLE, ANY SPOUSE OF THE HEAD OF HOUSEHOLD SHOULD BE SELECTED TO COMPLETE THE HOUSEHOLD QUESTIONNAIRE. IN THE CASE THAT NEITHER THE HEAD OF HOUSEHOLD NOR ANY SPOUSE IS AVAILABLE, PLEASE COMPLETE THE HOUSEHOLD QUESTIONNAIRE WITH ANY OTHER AVAILABLE ADULT MEMBER OF THE HOUSEHOLD, AGED 18+]

Module 1: Household Schedule

Line no.	Usual residents and visitors	Respondent	Relationship to head of household	Sex	Residence		Age	Marital status
1	Q101A	Q101B	Q101C	Q101D	Q102A	Q102B	Q103	Q104
	Please give me the names of the persons who usually live in your household and guests of the household who stayed here last night, starting with the respondent.<br><br>[WHEN THE RESPONDENT RESPONDS FOR THEMSELF, REFER TO 'YOU' OR 'YOUR']<br><br>[AFTER LISTING THE NAMES AND RECORDING THE RELATIONSHIP AND SEX FOR EACH PERSON (Q101A-C), ASK QUESTIONS **102A-C** TO BE SURE THAT THE LISTING IS COMPLETE. THEN ASK APPROPRIATE QUESTIONS 103-111 FOR EACH PERSON.]	Is this the respondent?<br>1 Yes<br>2 No	What is the relationship of [NAME] to the head of the household?<br><br>01 head; 02 spouse; 03 son or daughter; 04 son-in-law or daughter-in-law; 05 grandchild; 06 parent; 07 parent-in-law; 08 sibling; 09 other relative; 10 adopted/ foster/ stepchild; 11 not related; 98 don't know	Is [NAME] male or female?<br><br>01 Male; 02 Female	Does [NAME] usually live here?<br><br>01 Yes; 02 No	Did [NAME] sleep here last night?<br><br>01 Yes; 02 No	How old is [NAME]?<br><br>[IN YEARS; ASK TO SEE ID IF RESPONDENT UNSURE OF OWN AGE]<br><br>999 = Don't know	[IF AGED 16 OR OLDER]<br><br>What is [NAME]'s current marital status:<br>01 married or living together; 02 divorced / separated; 03 widowed; 04 Never-married and never lived together
01								
02								
03								
04								
05								
06								
07								
08								
09								
10								

1

[Name of Country]
[Name of Slum]

Line no.	Ever attended school		Current work status	Migration status				Health status
1	Q105A	Q105B	Q106	Q107A	Q107B	Q107C	Q107D	Q108
	[IF AGED 6 OR OLDER]<br><br>Has [NAME] ever attended school?<br><br>01 Yes;<br>02 No<br>08 Don't know [IF "NO" OR "DON'T KNOW" GO TO Q107]	[IF AGED 6 OR OLDER]<br><br>What is the highest level of school [NAME] has attended?<br><br>What is the highest grade [NAME] completed at that level?	Is [NAME] currently working?<br><br>01 Yes; 02 No<br><br>[DEFINE 'CURRENTLY WORKING' AS HAVING WORKED IN THE LAST WEEK IF UNSURE]	Has [NAME] always lived in this area/neighbourhood?<br><br>01 Yes; 02 No; 08 Don't know<br><br>[DEFINE AS HAVING BEEN BORN HERE IF UNSURE]<br><br>[IF "YES" GO TO Q110, IF "NO" CONTINUE WITH Q109B-D]	How long has [NAME] been living (continuously) in this area?<br><br>[☐☐ YEARS ☐☐ MONTHS]	Where was [NAME] living before?<br><br>01 in the same area/ neighbourhood; 02 In another "slum" area in this country; 03 In "non-slum" urban area in this country; 04 In a rural area in this country; 05 Outside the country.<br><br>SENSITIVE TRANSLATION REQUIRED	Where has [NAME] lived for most of their life<br><br>01 in the same area/ neighbourhood; 02 In another "slum" area in this country; 03 In "non-slum" urban area in this country; 04 In a rural area in this country; 05 Outside the country.<br><br>SENSITIVE TRANSLATION REQUIRED	Does [NAME] have any physical or mental health conditions or illness lasting or expected to last for 12 months or more?<br><br>01 Yes;<br>02 No
01					☐☐ ☐☐			
02					☐☐ ☐☐			
03					☐☐ ☐☐			
04					☐☐ ☐☐			
05					☐☐ ☐☐			
06					☐☐ ☐☐			
07					☐☐ ☐☐			
08					☐☐ ☐☐			
09					☐☐ ☐☐			
10					☐☐ ☐☐			

[Name of Country]
[Name of Slum]

Health insurance status					Digital communication			
Q109A	Q109B	Q109C	Q109D	Q109E	Q110A	Q110B	Q110C	Q110D
Is [NAME] covered by any kind of insurance plan?<br><br>[IF "NO" Q111A, IF "YES" continue with Q110B-E]	What type of health insurance is (NAME) covered by?<br><br>[SELECT ALL THAT RESPONDENT INDICATES]<br><br>Mutual health organisation/ community-based health insurance;<br>Health insurance through employer;<br>Social security;<br>Other privately purchased health insurance;<br>Other, please specify	How much does your household pay for [NAME]'s health insurance each year?	Is [NAME] covered by insurance only because of his/her relationship to someone else who has health insurance?	Who is enrolled in the insurance plan that gives [NAME] health insurance?	Does [NAME] carry a mobile phone day-to-day?<br><br>01 Yes; 02 No; 08 Don't know	Is [NAME] able to use a computer, tablet or other form of digital communication other than a mobile phone, day-to-day?<br><br>01 Yes; 02 No; 08 Don't know	How do they usually access use of a mobile phone?<br><br>1. They have no access<br>2. They ask another household member to communicate for them<br>3. They borrow a phone from another member of the household when they need to use one	How do they usually access the internet?<br><br>1. They have no access<br>2. They ask another member of the household to communicate for them<br>3. They borrow a device from another member of the household when they need to use one

Q112A. Just to make sure that I have a complete listing: are there any other people such as small children or infants that we have not listed?

Q112B. Are there any other people who may not be members of your family, such as domestic servants, lodgers or friends who usually live here?

Q112C. Are there any guests or temporary visitors staying here, or anyone else who stayed here last night, who have not been listed?

3

[Name of Country]
[Name of Slum]

Module 2: Household characteristics

IN-COUNTRY TEAMS TO DELETE AS APPROPRIATE

I would like to ask you some questions about your dwelling or home. [BANGLADESH VERSION]

Q201	What is the main source of drinking water for members of your household?	1. Piped into dwelling……→<br>2. Piped to yard/plot……→<br>3. Public tap/standpipe<br>4. Tube well or Borehole<br>5. Dugwell: Protected well<br>6. Dugwell: Unprotected well<br>7. Protected spring<br>8. Unprotected spring<br>9. Rainwater……→<br>10. Tanker truck<br>11. Cart with small tank<br>12. Surface water (river/dam/lake/pond/stream/canal/irrigation channel)<br>13. Bottled water<br>97. Other	Q206<br>Q206<br><br><br><br><br><br><br>Q206
Q202	Where is that water source located?	1. In own dwelling……→<br>2. In own yard/plot……→<br>3. Elsewhere	Q206<br>Q206
Q203	How long does it take to go there, get water, and come back?	☐☐☐ Minutes<br>998. Don't know	
Q203A	Who usually goes to this source to fetch the water for your household	1. Adult woman<br>2. Adult man<br>3. Female child<br>4. Male child<br>5. No one person usually fetches the water	
Q204	Do you share this source with other households?	1. Yes<br>2. No……→	 Q206
Q205	How many households use this source of water?	No. of households if less than 10: [0][ ]<br><br>95. 10 of more households<br>98. Don't know	
Q206	Do you do anything to the water to make it safer to drink?	1. Yes<br>2. No……→<br>8. Don't know……→	 Q207<br>Q207
Q206A	What do you usually do to make the water safer to drink?<br><br>Anything else?<br><br>[RECORD ALL MENTIONED]	1. Boil<br>2. Add bleach/chlorine<br>3. Strain through a cloth<br>4. Use water filter (ceramic/sand/composite/etc.)<br>5. Solar disinfection<br>6. Let it stand and Settle<br>97. Other	

. BMJ Open 2022; 12:e054142. doi: 10.1136/bmjopen-2021-054142

[Name of Country]
[Name of Slum]

Q207	What kind of toilet facility do members of your household usually use?<br><br>[IF RESPONDENT INDICATES 'FLUSH' OR 'POUR FLUSH', THEN PROBE WITH:<br><br>WHERE DOES IT FLUSH TO?]	1. Flush/pour flush to piped sewer system<br>2. Flush/pour flush to septic tank<br>3. Flush/pour flush to pit latrine<br>4. Flush/pour flush to somewhere else<br>5. Flush/pour flush don't know where<br>6. Ventilated improved pit latrine<br>7. Pit latrine with slab<br>8. Pit latrine without slab/Open pit<br>9. Composting toilet<br>10. Bucket toilet<br>11. Hanging toilet/hanging latrine<br>12. No facility/bush/field…………………..……→<br>97. Other	Q210
Q208	Do you share this toilet facility with other households?	1. Yes<br>2. No……………………………………………………→	Q210
Q209	How many households use this toilet facility	No of household if less than 10 [0][ ]<br>95. 10 or more households<br>98. Don't know	
Q210	Does your household have:<br>[RECORD ALL THAT APPLY]	1. Electricity<br>2. Solar Electricity<br>3. Radio<br>4. Television<br>5. Mobile telephone<br>6. Non-mobile telephone<br>7. Refrigerator<br>8. Almirah/wardrobe<br>9. Electric Fan<br>10. Blu-ray/DVD/VCD Player<br>11. Water pump<br>12. IPS/Generator<br>13. Air conditioner<br>14. Computer Laptop	
Q210A	Does this household receive a cash transfer or any social assistance from the government, NGO's, CBO's, Churches or Other organizations?<br><br>[DEFINE AS HAVE RECEIVED IN THE LAST MONTH IF UNSURE]	1. Yes<br>1. No…………………………………………………→	Q211
Q210B	For what reason does the household receive a cash transfer or social assistance?<br><br>Any other reason?<br><br>[RECORD ALL MENTIONED]	1. Orphaned children 18 years or younger<br>2. Elderly person<br>3. Person with severe disability<br>4. Urban food subsidy<br>5. Food aid for person in arid and semi-arid lands<br>6. Health voucher<br>7. Food/cash for work<br>8. School feeding<br>9. Hunger safety net programme<br>2. Other	

[Name of Country]
[Name of Slum]

Q211	What type of fuel does your household mainly use for cooking?	3. Electricity<br>4. LPG<br>5. Natural Gas<br>6. Biogas<br>7. Kerosene<br>8. Coal, lignite<br>9. Charcoal<br>10. Wood<br>11. Straw/shrubs/grass<br>12. Agricultural crop<br>13. Animal dung<br>14. No food cooked in household………………→<br>97. Other	Q215
Q212	Where is cooking usually done?	1. In a room used for living or sleeping<br>2. In a separate room used as kitchen<br>3. In a separate building used as kitchen<br>4. Outdoor<br>7. Other, please specify:	
Q213	[MAIN MATERIAL OF THE FLOOR- RECORD OBSERVATION]	1. Natural floor (earth/sand)<br>2. Rudimentary floor: wood planks<br>3. Rudimentary floor: palm/bamboo<br>4. Finished floor: Parquet or polished wood<br>5. Finished floor: ceramic tiles<br>6. Finished floor: Cement<br>7. Finished floor: Carpet<br>97. Other	
Q214	[MAIN MATERIAL OF THE ROOF- RECORD OBSERVATION]	1. Natural roof: no roof<br>2. Natural roof: thatch/palm leaf<br>3. Rudimentary roof: palm/bamboo<br>4. Rudimentary roof: wood planks<br>5. Rudimentary roof: cardboard<br>6. Finished roof: tin<br>7. Finished roof: wood<br>8. Finished roof: ceramic tiles<br>9. Finished roof: cement<br>10. Finished roof: roofing shingles<br>97. Other	
Q215	[MAIN MATERIAL OF THE EXTERIOR WALLS- RECORD OBSERVATION]	1. Natural walls: no walls<br>2. Natural walls: cane/palm/trunks<br>3. Natural walls: dirt<br>4. Rudimentary walls: bamboo with mud<br>5. Rudimentary walls: stone with mud<br>6. Rudimentary walls: plywood<br>7. Rudimentary walls: cardboard<br>8. Finished walls: tin<br>9. Finished walls: cement<br>10. Finished walls: stone with lime/cement<br>11. Finished walls: brick<br>12. Finished walls: woodplanks/shingles<br>97. Other	
Q216	How many rooms in this household are used for sleeping?	☐☐ Rooms	

[Name of Country]
[Name of Slum]

Q217	Does any member of this household own:	1. A car/truck/microbus<br>2. An autobike/tempo/CNG<br>3. A rickshaw/van<br>4. A bicycle<br>5. A motorcycle or motor scooter	
Q217A	Does your household own this structure (house, flat, shack)?	1. Yes<br>2. No	Q217B<br>Q217C
Q217B	Does your household own the land on which the structure (house, flat, shack) sits?	1. Yes<br>2. No	
Q217C	Do you pay rent?	1. Pays rent/lease<br>2. No rent with consent of owner<br>3. No rent: squatting	
Q218	Does your household own any homestead?	4. Yes<br>5. No	
Q218AA	Does your household own any land (other than the homestead land)?	1. Yes<br>2. No…………………………………………… →	Q222
Q219	Does this household own any livestock, herds, other farm animals or poultry?	1. Yes<br>2. No…………………………………………… →	Q224
Q220	How many of the following animals does this household own?<br><br>Buffaloes?<br>Cows?<br>Goats or sheep?<br>Chickens or ducks?<br>Other farm animals?		
Q221	Does any member of this household have a bank account?	1. Yes<br>2. No	
Q222	In the past 7 days were there days when your household did not have enough food or money to buy food?	1. Yes<br>2. No	
Q223	How many days in the month do you have air time (for calls and SMS) for at least one mobile phone in the household	1. Everyday<br>2. Almost every day (over 21 days a month but not every day)<br>3. More than half the days (over 14 but not as many as 21)<br>4. More than 7 days but less than 14<br>5. Less than 7 days<br>6. Never	
Q224	How many days in the month does someone in the household have data or access to wifi (for accessing internet for searching the web, using social media or using Email) for at least one of your digital communication devices in the household (smart phone, laptop, tablet)?	1. Everyday<br>2. Almost every day (over 21 days a month but not every day)<br>3. More than half the days (over 14 but not as many as 21)<br>4. More than 7 days but less than 14<br>Less than 7 days<br>5. Never	

7

[Name of Country]
[Name of Slum]

I would like to ask you some questions about your dwelling or home. KENYA VERSION

Q201	What is the main source of drinking water for members of your household?	1. Piped into dwelling……………………→<br>2. Piped to yard/plot……………………→<br>3. Public tap/standpipe<br>4. Tube well or Borehole<br>5. Dugwell: Protected well<br>6. Dugwell: Unprotected well<br>7. Protected spring<br>8. Unprotected spring<br>9. Rainwater…………………………….→<br>10. Tanker truck<br>11. Cart with small tank<br>12. Surface water (river/dam/lake/pond/stream/ canal/irrigation channel)<br>13. Bottled water<br>97. Other	Q206<br>Q206<br><br><br><br><br><br><br>Q206
Q202	Where is that water source located?	1. In own dwelling…………………………….→<br>2. In own yard/plot…………………………..→<br>3. Elsewhere	Q206<br>Q206
Q203	How long does it take to go there, get water, and come back?	☐☐☐ Minutes<br>998. Don't know	
Q203A	Who usually goes to this source to fetch the water for your household	1. Adult woman<br>2. Adult man<br>3. Female child<br>4. Male child<br>5. No one person usually fetches the water	
Q204	Do you share this source with other households?	1. Yes<br>2. No…………………………………………→	 Q206
Q205	How many households use this source of water?	No. of households if less than 10: 0 ☐<br><br>95. 10 of more households<br>98. Don't know	
Q206	Do you do anything to the water to make it safer to drink?	1. Yes<br>2. No……………………………………………………→<br>8. Don't know…………………………………………→	 Q207<br>Q207
Q206A	What do you usually do to make the water safer to drink?<br><br>Anything else?<br><br>[RECORD ALL MENTIONED]	1. Boil<br>2. Add bleach/chlorine<br>3. Strain through a cloth<br>4. Use water filter (ceramic/sand/composite/etc.)<br>5. Solar disinfection<br>6. Let it stand and Settle<br>7. Cover the water container<br>97. Other	
Q207	What kind of toilet facility do members of your household usually use?	1. Flush/pour flush to piped sewer system<br>2. Flush/pour flush to septic tank<br>3. Flush/pour flush to pit latrine	

[Name of Country]
[Name of Slum]

	[IF RESPONDENT INDICATES 'FLUSH' OR 'POUR FLUSH', THEN PROBE WITH: WHERE DOES IT FLUSH TO?]	4. Flush/pour flush to somewhere else<br>5. Flush/pour flush don't know where<br>6. Ventilated improved pit latrine<br>7. Pit latrine with slab<br>8. Pit latrine without slab/Open pit<br>9. Composting toilet<br>10. Bucket toilet<br>11. Hanging toilet/hanging latrine<br>12. No facility/bush/field……………….……→<br>97. Other	 <br><br><br><br><br><br><br>Q210
Q208	Do you share this toilet facility with other households?	1. Yes<br>2. No………………………………………………→	 Q210
Q209	How many households use this toilet facility	No of household if less than 10 [0][ ]<br>95. 10 or more households<br>98. Don't know	
Q210	Does your household have:<br>[RECORD ALL THAT APPLY]	1. Electricity<br>2. Radio<br>3. Television<br>4. Mobile telephone<br>5. Non-mobile telephone<br>6. Refrigerator<br>7. Solar panel<br>8. Table<br>9. Chair<br>10. Sofa<br>11. Bed<br>12. Cupboard<br>13. Clock<br>14. Microwave oven<br>15. Blu-ray/VCD/DVD Player<br>16. Cassette/CD Player	
Q210A	Does this household receive a cash transfer or any social assistance from the government, NGOs, CBOs, Churches or other organisations?<br><br>[DEFINE AS HAVE RECEIVED IN THE LAST MONTH IF UNSURE]	1. Yes<br>2. No………………………………………………→	 Q211
Q210B	For what reason does the household receive a cash transfer or social assistance?<br><br>Any other reason?<br><br>[RECORD ALL MENTIONED]	1. Orphaned children 18 years or younger<br>2. Elderly person<br>3. Person with severe disability<br>4. Urban food subsidy<br>5. Food aid for person in arid and semi-arid lands<br>6. Health voucher<br>7. Food/cash for work<br>8. School feeding<br>9. Hunger safety net programme<br>97. Other	

[Name of Country]
[Name of Slum]

Q211	What type of fuel does your household mainly use for cooking?	1. Electricity<br>2. LPG/Natural gas<br>3. Biogas<br>4. Parafin/Kerosene<br>5. Coal, lignite<br>6. Charcoal<br>7. Wood<br>8. Straw/shrubs/grass<br>9. Agricultural crop<br>10. Animal dung<br>11. No food cooked in household…………….→<br>97. Other	Q215
Q212	Where is cooking usually done?	1. In a room used for living or sleeping<br>2. In a separate room used as kitchen<br>3. In a separate building used as kitchen<br>4. Outdoor<br>97. Other, please specify:	
Q213	[MAIN MATERIAL OF THE FLOOR- RECORD OBSERVATION]	1. Natural floor: earth/sand<br>2. Natural floor: dung<br>3. Rudimentary floor: wood planks<br>4. Rudimentary floor: palm/bamboo<br>5. Finished floor: Parquet or polished wood<br>6. Finished floor: vinyl or asphalt strips<br>7. Finished floor: ceramic tiles<br>8. Finished floor: Cement<br>9. Finished floor: Carpet<br>97. Other	
Q214	[MAIN MATERIAL OF THE ROOF- RECORD OBSERVATION]	1. Natural roof: no roof<br>2. Natural roof: thatch/grass/makuti<br>3. Natural roof: dung/mud/sod<br>4. Rudimentary roof: iron sheets<br>5. Rudimentary roof: tin cans<br>6. Finished roof: asbestos sheet<br>7. Finished roof: concrete<br>8. Finished roof: tiles<br>97. Other	
Q215	[MAIN MATERIAL OF THE EXTERIOR WALLS- RECORD OBSERVATION]	1. Natural walls: no walls<br>2. Natural walls: cane/palm/trunks<br>3. Natural walls: dung/mud/sod<br>4. Rudimentary walls: bamboo with mud<br>5. Rudimentary walls: stone with mud<br>6. Rudimentary walls: uncovered adobe<br>7. Rudimentary walls: plywood<br>8. Rudimentary walls: cardboard<br>9. Rudimentary walls: reused wood<br>10. Rudimentary walls: iron sheets<br>11. Finished walls: cement<br>12. Finished walls: stone with lime/cement<br>13. Finished walls: brick<br>14. Finished walls: cement blocks<br>15. Finished walls: covered adobe<br>16. Finished walls: wood planks/shingles	

[Name of Country]
[Name of Slum]

		97. Other	
Q216	How many rooms in this household are used for sleeping?	☐☐ Rooms	
Q217	Does any member of this household own:	1. A watch<br>2. A bicycle<br>3. A motorcycle/scooter<br>4. An animal-drawn cart<br>5. A car/truck<br>6. A boat with a motor	
Q217A	Does your household own this structure (house, flat, shack)?	1. Yes<br>2. No	Q217B<br>Q217C
Q217B	Does your household own the land on which the structure (house, flat, shack) sits?	1. Yes<br>2. No	Q218<br>Q218
Q217C	Does your household pay rent?	6. Pays rent/lease<br>7. No rent with consent of owner<br>1. No rent: squatting	
Q218	Does any member of this household own any agricultural land?	2. Yes<br>3. No…………………………………… →	Q222
Q219	Does this household own any livestock, herds, other farm animals or poultry?	1. Yes<br>2. No…………………………………… →	Q224
Q220	How many of the following animals does this household own?<br><br>Local cattle (indigenous)<br>Exotic/grade cattle<br>Horses, donkeys, or camels?<br>Goats or sheep?<br>Pigs?<br>Chickens?<br>Other?	☐☐<br>☐☐<br>☐☐<br>☐☐<br>☐☐<br>☐☐	
Q221	Does any member of this household have a bank account?	1. Yes<br>2. No	
Q222	In the past 7 days were there days when your household did not have enough food or money to buy food?	1. Yes<br>2. No	
Q223	How many days in the month do you have air time (for calls and SMS) for at least one mobile phone in the household	1. Everyday<br>2. Almost every day (over 21 days a month but not every day)<br>3. More than half the days (over 14 but not as many as 21)<br>4. More than 7 days but less than 14<br>5. Less than 7 days<br>6. Never	
Q224	How many days in the month does someone in the household have data or access to wifi (for accessing internet for searching the web, using social media or using Email) for at least one of your digital communication devices I the household (smart phone, laptop, tablet)?	1. Everyday<br>2. Almost every day (over 21 days a month but not every day)<br>3. More than half the days (over 14 but not as many as 21)<br>4. More than 7 days but less than 14	

[Name of Country]
[Name of Slum]

		5. Less than 7 days<br>6. Never	

I would like to ask you some questions about your dwelling or home. NIGERIA VERSION

Q201	What is the main source of drinking water for members of your household?	1. Piped into dwelling……………………→<br>2. Piped to yard/plot……………………→<br>3. Public tap/standpipe<br>4. Tube well or Borehole<br>5. Dugwell: Protected well<br>6. Dugwell: Unprotected well<br>7. Protected spring<br>8. Unprotected spring<br>9. Rainwater……………………………→<br>10. Tanker truck<br>11. Cart with small tank<br>12. Surface water (river/dam/lake/pond/stream/ canal/irrigation channel)<br>13. Bottled water<br>97. Other	Q206<br>Q206<br><br><br><br><br><br><br>Q206
Q202	Where is that water source located?	1. In own dwelling……………………………→<br>2. In own yard/plot…………………………→<br>3. Elsewhere	Q206<br>Q206
Q203	How long does it take to go there, get water, and come back?	[ ][ ][ ] Minutes<br>998. Don't know	
Q203A	Who usually goes to this source to fetch the water for your household	1. Adult woman<br>2. Adult man<br>3. Female child<br>4. Male child<br>5. No one person usually fetches the water	
Q204	Do you share this source with other households?	1. Yes<br>2. No……………………………………→	 Q206
Q205	How many households use this source of water?	No. of households if less than 10: [0][ ]<br><br>95. 10 of more households<br>98. Don't know	
Q206	Do you do anything to the water to make it safer to drink?	1. Yes<br>2. No…………………………………………………→<br>8. Don't know………………………………………→	 Q207<br>Q207
Q206A	What do you usually do to make the water safer to drink?<br><br>Anything else?<br><br>[RECORD ALL MENTIONED]	1. Boil<br>2. Add bleach/chlorine<br>3. Strain through a cloth<br>4. Use water filter (ceramic/sand/composite/etc.)<br>5. Solar disinfection<br>6. Let it stand and Settle<br>7. Alum<br>97. Other	

[Name of Country]
[Name of Slum]

Q207	What kind of toilet facility do members of your household usually use?<br><br>[IF RESPONDENT INDICATES 'FLUSH' OR 'POUR FLUSH', THEN PROBE WITH:<br><br>WHERE DOES IT FLUSH TO?]	1. Flush/pour flush to piped sewer system<br>2. Flush/pour flush to septic tank<br>3. Flush/pour flush to pit latrine<br>4. Flush/pour flush to somewhere else<br>5. Flush/pour flush don't know where<br>6. Ventilated improved pit latrine<br>7. Pit latrine with slab<br>8. Pit latrine without slab/Open pit<br>9. Composting toilet<br>10. Bucket toilet<br>11. Hanging toilet/hanging latrine<br>12. No facility/bush/field…………………..…….→<br>97. Other	Q210
Q208	Do you share this toilet facility with other households?	1. Yes<br>2. No……………………………………………→	Q210
Q209	How many households use this toilet facility	No of household if less than 10 [0][ ]<br>95. 10 or more households<br>98. Don't know	
Q210	Does your household have:<br>[RECORD ALL THAT APPLY]	1. Electricity<br>2. Radio<br>3. Television<br>4. Mobile telephone<br>5. Non-mobile telephone<br>6. Refrigerator<br>7. Cable TV<br>8. Generating Set<br>9. Air conditioner<br>10. Computer<br>11. Electric Iron<br>12. Fan	
Q210A	Does this household receive a cash transfer or any social assistance from the government, NGOs, CBOs, churches or other organizations?<br><br>[DEFINE AS HAVING RECEIVED IN THE LAST MONTH IF UNSURE]	1. Yes<br>2. No……………………………………………….→	Q211
Q210B	For what reason does the household receive a cash transfer or social assistance?<br><br>Any other reason?<br><br>[RECORD ALL MENTIONED]	1. Orphaned children 18 years or younger<br>2. Elderly person<br>3. Person with severe disability<br>4. Urban food subsidy<br>5. Food aid for person in arid and semi-arid lands<br>6. Health voucher<br>7. Food/cash for work<br>8. School feeding<br>9. Hunger safety net programme<br>97. Other	
Q211	What type of fuel does your household mainly use for cooking?	1. Electricity<br>2. LPG<br>3. Natural gas<br>4. Biogas	

13

[Name of Country]
[Name of Slum]

		5. Kerosene<br>6. Coal, lignite<br>7. Charcoal<br>8. Wood<br>9. Straw/shrubs/grass<br>10. Agricultural crop<br>11. Animal dung<br>12. No food cooked in household……….→<br>97. Other	Q215
Q212	Where is cooking usually done?	1. In a room used for living or sleeping<br>2. In a separate room used as kitchen<br>3. In a separate building used as kitchen<br>4. Outdoors<br>97. Other, please specify:	
Q213	[MAIN MATERIAL OF THE FLOOR- RECORD OBSERVATION]	1. Natural floor: earth/sand<br>2. Natural floor: dung<br>3. Rudimentary floor: wood planks<br>4. Rudimentary floor: palm/bamboo<br>5. Finished floor: parquet or polished wood<br>6. Finished floor: vinyl or asphalt strips<br>7. Finished floor: ceramic tiles<br>8. Finished floor: cement<br>9. Finished floor: carpet/rug<br>97. Other	
Q214	[MAIN MATERIAL OF THE ROOF- RECORD OBSERVATION]	1. Natural roof: no roof<br>2. Natural roof: thatch/palm leaf<br>3. Rudimentary roof: rustic mat<br>4. Rudimentary roof: palm/bamboo<br>5. Rudimentary roof: wood planks<br>6. Rudimentary roof: cardboard<br>7. Finished roof: metal/zinc<br>8. Finished roof: wood<br>9. Finished roof: ceramic tiles<br>10. Finished roof: cement<br>11. Finished roof: roofing shingles<br>97. Other	
Q215	[MAIN MATERIAL OF THE EXTERIOR WALLS- RECORD OBSERVATION]	1. Natural walls: no walls<br>2. Natural walls: cane/palm/trunks<br>3. Natural walls: dirt (mud)<br>4. Rudimentary walls: bamboo with mud<br>5. Rudimentary walls: stone with mud<br>6. Rudimentary walls: plywood<br>7. Rudimentary walls: cardboard<br>8. Rudimentary walls: reused wood<br>9. Finished walls: cement<br>10. Finished walls: stone with lime/cement<br>11. Finished walls: bricks<br>12. Finished walls: cement blocks<br>13. Finished walls: wood planks/shingles<br>97. Other	
Q216	How many rooms in this household are used for sleeping?	☐☐ Rooms (Sleeping)	

[Name of Country]
[Name of Slum]

Q216A	How many rooms in total are in your household, including rooms for sleeping and all other rooms?	☐☐ Rooms (Total)	
Q217	Does any member of this household own:<br><br>[RECORD ALL THAT APPLY]	1. A watch<br>2. A bicycle<br>3. A motorcycle or motor scooter<br>4. An animal-drawn cart<br>5. A car or truck<br>6. A boat with a motor<br>7. A canoe	
Q217A	Does your household own this structure (house, flat, shack)?	1. Yes<br>2. No	Q217B<br>Q217C
Q217B	Does your household own the land on which the structure (house, flat, shack) sits?	1. Yes<br>2. No	Q218<br>Q218
Q217C	Do you pay rent?	8. Pays rent/lease<br>9. No rent with consent of owner<br>1. No rent: squatting	
Q218	Does any member of this household own any agricultural land?	2. Yes<br>3. No…………………………………… →	Q222
Q219	Does this household own any livestock, herds, other farm animals or poultry?	1. Yes<br>2. No…………………………………… →	Q224
Q220	How many of the following animals does this household own?<br><br>Milk cows or bulls?<br>Horses, donkeys, or mules?<br>Goats?<br>Sheep?<br>Chickens/ducks?<br>Pigs?<br>Other farm animals?	☐☐<br>☐☐<br>☐☐<br>☐☐<br>☐☐<br>☐☐<br>☐☐	
Q221	Does any member of this household have a bank account?	1. Yes<br>2. No	
Q222	In the past 7 days were there days when your household did not have enough food or money to buy food?	1. Yes<br>2. No	
Q223	How many days in the month do you have air time (for calls and SMS) for at least one mobile phone in the household	1. Everyday<br>2. Almost every day (over 21 days a month but not every day)<br>3. More than half the days (over 14 but not as many as 21)<br>4. More than 7 days but less than 14<br>5. Less than 7 days<br>6. Never	
Q224	How many days in the month does someone in the household have data or access to wifi (for accessing internet for searching the web, using social media or using Email) for at least	1. Everyday<br>2. Almost every day (over 21 days a month but not every day)	

15

[Name of Country]
[Name of Slum]

	one of your digital communication devices I the household (smart phone, laptop, tablet)?	3. More than half the days (over 14 but not as many as 21)<br>4. More than 7 days but less than 14<br>5. Less than 7 days<br>6. Never	

I would like to ask you some questions about your dwelling or home. PAKISTAN VERSION

Q201	What is the main source of drinking water for members of your household?	1. Piped into dwelling…………………….→<br>2. Piped to yard/plot…………………….→<br>3. Public tap/standpipe<br>4. Tube well or Borehole<br>5. Handpump<br>6. Dugwell: Protected well<br>7. Dugwell: Unprotected well<br>8. Protected spring<br>9. Unprotected spring<br>10. Rainwater……………………………….→<br>11. Tanker truck<br>12. Filtration plant<br>13. Cart with small tank<br>14. Surface water (river/dam/lake/pond/stream/ canal/irrigation channel)<br>15. Bottled water<br>97. Other	Q206<br>Q206<br><br><br><br><br><br><br><br>Q206
Q202	Where is that water source located?	1. In own dwelling……………………………..→<br>2. In own yard/plot…………………………..→<br>3. Elsewhere	Q206<br>Q206
Q203	How long does it take to go there, get water, and come back?	☐☐☐ Minutes<br>998. Don't know	
Q203A	Who usually goes to this source to fetch the water for your household	1. Adult woman<br>2. Adult man<br>3. Female child<br>4. Male child<br>5. No one person usually fetches the water	
Q204	Do you share this source with other households?	1. Yes<br>2. No……………………………………..→	 Q206
Q205	How many households use this source of water?	No. of households if less than 10: [0][ ]<br><br>95. 10 of more households<br>98. Don't know	
Q206	Do you do anything to the water to make it safer to drink?	1. Yes<br>2. No…………………………………………….→<br>8. Don't know……………………………………→	 Q207<br>Q207
Q206A	What do you usually do to make the water safer to drink?<br><br>Anything else?	1. Boil<br>2. Add bleach/chlorine<br>3. Strain through a cloth	

16

[Name of Country]
[Name of Slum]

	[RECORD ALL MENTIONED]	4. Use water filter (ceramic/sand/composite/etc.)<br>5. Solar disinfection<br>6. Let it stand and Settle<br>97. Other	
Q207	What kind of toilet facility do members of your household usually use?<br><br>[IF RESPONDENT INDICATES ‘FLUSH’ OR ‘POUR FLUSH’, THEN PROBE WITH:<br><br>WHERE DOES IT FLUSH TO?]	1. Flush/pour flush to piped sewer system<br>2. Flush/pour flush to septic tank<br>3. Flush/pour flush to pit latrine<br>4. Flush/pour flush to somewhere else<br>5. Flush/pour flush don’t know where<br>6. Ventilated improved pit latrine<br>7. Pit latrine with slab<br>8. Pit latrine without slab/Open pit<br>9. Bucket toilet<br>10. Hanging toilet/hanging latrine<br>11. No facility/bush/field……………….…….→<br>97. Other	Q210
Q208	Do you share this toilet facility with other households?	1. Yes<br>2. No…………………………………………→	Q210
Q209	How many households use this toilet facility	No of household if less than 10 [0][ ]<br>95. 10 or more households<br>98. Don’t know	
Q210	Does your household have:<br>[RECORD ALL THAT APPLY]	1. Electricity<br>2. Radio<br>3. Television<br>4. Landline telephone<br>5. Refrigerator<br>6. Almirah/cabinet<br>7. Chair<br>8. Room cooler<br>9. Air conditioner<br>10. Washing machine<br>11. Water pump<br>12. Bed<br>13. Clock<br>14. Sofa<br>15. Camera<br>16. Sewing machine<br>17. Computer<br>18. Internet connection	
Q210A	Does this household receive a cash transfer or any social assistance from the government, NGOs, CBOs, churches or other organizations?<br><br>[DEFINE AS HAVING RECEIVED IN THE LAST MONTH IF UNSURE]	1. Yes<br>2. No…………………………………………….→	Q211
Q210B	For what reason does the household receive a cash transfer or social assistance?<br><br>Any other reason?<br><br>[RECORD ALL MENTIONED]	98. Orphaned children 18 years or younger<br>99. Elderly person<br>100. Person with severe disability<br>101. Urban food subsidy	

17

[Name of Country]
[Name of Slum]

		102. Food aid for person in arid and semi-arid lands<br>103. Health voucher<br>104. Food/cash for work<br>105. School feeding<br>106. Hunger safety net programme<br>3. Other	
Q211	What type of fuel does your household mainly use for cooking?	1. Electricity<br>2. LPG<br>3. Natural gas<br>4. Biogas<br>5. Kerosene<br>6. Coal, lignite<br>7. Charcoal<br>8. Wood<br>9. Straw/shrubs/grass<br>10. Animal dung<br>11. No food cooked in household………→<br>97. Other	 <br><br><br><br><br><br><br><br><br>Q215
Q212	Where is cooking usually done?	1. In a room used for living or sleeping<br>2. In a separate room used as kitchen<br>3. In a separate building used as kitchen<br>4. Outdoors<br>97. Other, please specify:	
Q213	[MAIN MATERIAL OF THE FLOOR- RECORD OBSERVATION]	1. Natural floor: earth/sand/mud<br>2. Natural floor: dung<br>3. Rudimentary floor: wood planks<br>4. Rudimentary floor: palm/bamboo<br>5. Finished floor: parquet or polished wood<br>6. Finished floor: vinyl or asphalt strips<br>7. Finished floor: ceramic tiles<br>8. Finished floor: cement<br>9. Finished floor: carpet<br>10. Finished floor: chips/terrazzo<br>11. Finished floor: bricks<br>12. Finished floor: mats<br>13. Finished floor: marble<br>97. Other	
Q214	[MAIN MATERIAL OF THE ROOF- RECORD OBSERVATION]	1. Natural roof: no roof<br>2. Natural roof: thatch/palm leaf<br>3. Natural roof: sod/grass<br>4. Rudimentary roof: rustic mat<br>5. Rudimentary roof: palm/bamboo<br>6. Rudimentary roof: wood planks<br>7. Rudimentary roof: cardboard<br>8. Finished roof: iron sheets/asbestos<br>9. Finished roof: reinforced brick cement/rcc metal<br>10. Finished roof: wood/t iron/mud	

[Name of Country]
[Name of Slum]

		11. Finished roof: calamine/cement fiber<br>12. Finished roof: ceramic tiles<br>13. Finished roof: cement/rcc<br>14. Finished roof: roofing shingles<br>97. Other	
Q215	[MAIN MATERIAL OF THE EXTERIOR WALLS-RECORD OBSERVATION]	1. Natural walls: no walls<br>2. Natural walls: cane/palm/trunks<br>3. Natural walls: dirt<br>4. Natural walls: mud/stones<br>5. Natural walls: bamboo/sticks/mud<br>6. Rudimentary walls: unbaked bricks/mud<br>7. Rudimentary walls: carton/plastic<br>8. Rudimentary walls: bamboo with mud<br>9. Rudimentary walls: stone with mud<br>10. Rudimentary walls: uncovered adobe<br>11. Rudimentary walls: plywood<br>12. Rudimentary walls: cardboard<br>13. Rudimentary walls: reused wood<br>14. Finished walls: baked bricks<br>15. Finished walls: tent<br>16. Finished walls: cement<br>17. Finished walls: stone with lime/cement<br>18. Finished walls: bricks<br>19. Finished walls: cement blocks<br>20. Finished walls: covered adobe<br>21. Finished walls: wood planks/shingles<br>97. Other	
Q216	How many rooms in this household are used for sleeping?	☐☐ Rooms (Sleeping)	
Q217	Does any member of this household own:	1. Watch<br>2. Mobile telephone<br>3. Bicycle<br>4. Motorcycle or motor scooter<br>5. An animal-drawn cart<br>6. A car or truck or bus<br>7. A tractor<br>8. A boat with a motor<br>9. A boat without a motor	
Q217A	Does your household own this structure (house, flat, shack)?	1. Yes<br>2. No	
Q217B	Does your household own the land on which the structure (house, flat, shack) sits?	1. Yes<br>2. No	
Q217C	Do you pay rent?	10. Pays rent/lease<br>11. No rent with consent of owner<br>1. No rent: squatting	
Q218	Does any member of this household own any agricultural land?	2. Yes<br>3. No……………………………… →	Q222

[Name of Country]
[Name of Slum]

Q219	Does this household own any livestock, herds, other farm animals or poultry?	1. Yes<br>2. No……………………………… →	 Q224
Q220	How many of the following animals does this household own?<br><br>Milk cows or bulls?<br>Horses, donkeys or mules?<br>Goats?<br>Sheep?<br>Chickens?<br>Buffalo?<br>Camels?<br>Other?		
Q221	Does any member of this household have a bank account?	1. Yes<br>2. No	
Q222	In the past 7 days were there days when your household did not have enough food or money to buy food?	1. Yes<br>2. No	
Q223	How many days in the month do you have air time (for calls and SMS) for at least one mobile phone in the household	1. Everyday<br>2. Almost every day (over 21 days a month but not every day)<br>3. More than half the days (over 14 but not as many as 21)<br>4. More than 7 days but less than 14<br>5. Less than 7 days<br>6. Never	
Q224	How many days in the month does someone in the household have data or access to wifi (for accessing internet for searching the web, using social media or using Email) for at least one of your digital communication devices I the household (smart phone, laptop, tablet)?	1. Everyday<br>2. Almost every day (over 21 days a month but not every day)<br>3. More than half the days (over 14 but not as many as 21)<br>4. More than 7 days but less than 14<br>5. Less than 7 days<br>6. Never	

Household income and expenditure

In the last part of this section, I will ask you about the total income for the household in the last 30 days. I would like to know about all sources of income. I know it may be difficult to calculate that figure, but please do try to give as accurate an amount as possible. Remember that all information will be kept strictly confidential. This information is important to assess overall health and wellbeing of people in your household compared to other, similar households.

Q225	I would like you to tell me your best estimate of the TOTAL income that this household has had in the last 30 days.	1. Less than 1000 KSh<br>2. 1000 – 2499 KSh<br>3. 2500 – 4999 KSh<br>4. 5000 – 7499 KSh<br>5. 7500 – 9999 KSh<br>6. 10000 – 14999 KSh<br>7. 15000 – 20000 KSh<br>8. Above 20000 KSh	

[Name of Country]
[Name of Slum]

Q226	Did your household receive or earn any income from the following in the last 30 days?	1. Salaried/waged employment<br>2. Business<br>3. Savings<br>4. Agriculture<br>5. Financial gifts/ support from any source<br>6. Any other source of income	

How much in total did your household spend on the following items?<br><br>[IF RESPONDENT UNSURE, PLEASE PROMPT WITH "TO THE BEST OF YOUR RECOLLECTION" THEN "CAN YOU ESTIMATE APPROXIMATELY WHAT IT MIGHT HAVE BEEN?"]			
Q227a	FOOD in the last 7 days?	☐☐☐☐☐☐☐☐<br>8. Don't know	
Q227b	ENERGY (PARAFFIN, CHARCOAL) in the last 7 days?	☐☐☐☐☐☐☐☐<br>8. Don't know	
Q227c	WATER in the last 7 days?	☐☐☐☐☐☐☐☐<br>8. Don't know	
Q227d	TRANSPORT in the last 7 days?	☐☐☐☐☐☐☐☐<br>8. Don't know	
Q227e	FINANCIAL GIFT/SUPPORT TO OTHERS in the last 7 days?	☐☐☐☐☐☐☐☐<br>8. Don't know	
Q227f	ELECTRICITY in the last 30 days?	☐☐☐☐☐☐☐☐<br>8. Don't know	
Q227g	HEALTH CARE in the last 30 days?	☐☐☐☐☐☐☐☐<br>8. Don't know	
Q227h	RELIGIOUS OBLIGATIONS in the last 30 days?	☐☐☐☐☐☐☐☐<br>8. Don't know	
Q227i	RENT in the last month?	☐☐☐☐☐☐☐☐<br>8. Don't know	
Q227i	SCHOOL RELATED EXPENSES (school fees, scholastic materials) in the last 30 days?	☐☐☐☐☐☐☐☐<br>8. Don't know	
Q227k	OTHER in the last 7 days?	☐☐☐☐☐☐☐☐<br>8. Don't know	

[Name of Country]
[Name of Slum]

[Name of Country]
[Name of Slum]

Module 3: Household Health Care Spending

I would like to ask you more specific questions about how much your household and all its members spent in cash or in-kind on all health care and services that did not require an overnight stay. We want expenses in the last 3 months. If payment was in-kind, please estimate a monetary value. Please exclude costs to be reimbursed by insurance.

In the last 3 months, how much did your household spend on:		
Q301	Registration and consultation fees by doctors, nurses, or trained midwives that did not require an overnight stay?	☐☐☐☐☐☐☐☐ 8. Don't know
Q302	Health-care by traditional or alternative healers? [USE COUNTRY-SPECIFIC TERMS]	☐☐☐☐☐☐☐☐ 8. Don't know
Q303	Diagnostic and laboratory tests, such as x-rays or blood tests?	☐☐☐☐☐☐☐☐ 8. Don't know
Q304	Medications or drugs (prescription, non-prescription, traditional, homeopathic…)?	☐☐☐☐☐☐☐☐ 8. Don't know
Q305	Dentists or dental care?	☐☐☐☐☐☐☐☐ 8. Don't know
Q306	Ambulance or other transport?	☐☐☐☐☐☐☐☐ 8. Don't know
Q307	Costs associated with overnight stays in a hospital or health facility? Please exclude any reimbursements from insurance and transportation costs.	☐☐☐☐☐☐☐☐ 8. Don't know
Q308	Any other health care products or services that were not included above? Please specify: [PROMPT FOR FOOD COSTS IF PROVIDED TO HOUSEHOLD MEMBERS DURING HOSPITAL/CLINIC STAYS; PROMPT FOR HOME BASED CARE PROVIDED FOR HOUSEHOLD MEMBERS (ADULT OR CHILD) DUE TO LONG-TERM CONDITIONS OR GETTING OLD AND WEAK]	☐☐☐☐☐☐☐☐ 8. Don't know

[Name of Country]
[Name of Slum]
Finally, I want you to think of how you paid for your health care expenditures over the last 12 months. This includes costs for all fees, services and goods, including overnight stays.

Q309	In the last 12 months , have you borrowed from financial institutions, agencies (microfinance schemes, banks…), or individuals to pay for any health expenditures?	1. Yes<br>2. No<br>8. Don't know	
Q310	If you borrowed any money in the last 12 months to pay for health expenditures, are you expected to pay this back?	1. Yes…………………………………… →<br>2. No…………………………………… →	Q311<br>END
Q311	Have you started paying back the loan?	1. Yes…………………………………… →<br>2. No…………………………………… →	Q312<br>END
Q311A	Are you repaying or have you repayed the loan as a one-off payment or in installments?	1. Lump sum<br>2. Installments	Q312A<br>Q312B
Q312A	How much did you pay back?	________	Q401
Q312B	What is the monthly repayment on the loan including interest?<br><br>[FOR INFORMAL PAYMENT, PLEASE TEASE OUT THE AVERAGE MONTHLY REPAYMENT, IF ANY]	☐☐☐☐☐☐☐☐<br><br>8. Don't know	

END: Thank you for your time in completing the household questionnaire. I will now move on to complete an individual questionnaire with a member of your household.
