# Supplementary material for: Primary care doctor and nurse consultations among people who live in slums: a retrospective, cross-sectional survey in four countries
Source: BMJ Open. 2022 Jan 7;12(1):e054142. doi: 10.1136/bmjopen-2021-054142 (PMC8744106; doi:10.1136/bmjopen-2021-054142)
Supplement: Author's manuscript [file bmjopen-2021-054142.draft_revisions.pdf]

BMJ Open is committed to open peer review. As part of this commitment we make the peer review history of every article we publish publicly available.

When an article is published we post the peer reviewers' comments and the authors' responses online. We also post the versions of the paper that were used during peer review. These are the versions that the peer review comments apply to.

The versions of the paper that follow are the versions that were submitted during the peer review process. They are not the versions of record or the final published versions. They should not be cited or distributed as the published version of this manuscript.

BMJ Open is an open access journal and the full, final, typeset and author-corrected version of record of the manuscript is available on our site with no access controls, subscription charges or pay-per-view fees (http://bmjopen.bmj.com).

If you have any questions on BMJ Open's open peer review process please email info.bmjopen@bmj.com

BMJ Open

Primary-care doctor and nurse consultations among people who live in slums: A retrospective, cross-sectional survey in four countries

Journal:	*BMJ Open*
Manuscript ID	bmjopen-2021-054142
Article Type:	Original research
Date Submitted by the Author:	08-Jun-2021
Complete List of Authors:	Watson, Samuel; University of Birmingham
Keywords:	PRIMARY CARE, PUBLIC HEALTH, International health services < HEALTH SERVICES ADMINISTRATION & MANAGEMENT, Organisation of health services < HEALTH SERVICES ADMINISTRATION & MANAGEMENT

SCHOLARONE™
Manuscripts

I, the Submitting Author has the right to grant and does grant on behalf of all authors of the Work (as defined in the below author licence), an exclusive licence and/or a non-exclusive licence for contributions from authors who are: i) UK Crown employees; ii) where BMJ has agreed a CC-BY licence shall apply, and/or iii) in accordance with the terms applicable for US Federal Government officers or employees acting as part of their official duties; on a worldwide, perpetual, irrevocable, royalty-free basis to BMJ Publishing Group Ltd ("BMJ") its licensees and where the relevant Journal is co-owned by BMJ to the co-owners of the Journal, to publish the Work in this journal and any other BMJ products and to exploit all rights, as set out in our licence.

The Submitting Author accepts and understands that any supply made under these terms is made by BMJ to the Submitting Author unless you are acting as an employee on behalf of your employer or a postgraduate student of an affiliated institution which is paying any applicable article publishing charge ("APC") for Open Access articles. Where the Submitting Author wishes to make the Work available on an Open Access basis (and intends to pay the relevant APC), the terms of reuse of such Open Access shall be governed by a Creative Commons licence – details of these licences and which Creative Commons licence will apply to this Work are set out in our licence referred to above.

Other than as permitted in any relevant BMJ Author's Self Archiving Policies, I confirm this Work has not been accepted for publication elsewhere, is not being considered for publication elsewhere and does not duplicate material already published. I confirm all authors consent to publication of this Work and authorise the granting of this licence.

For peer review only

Primary-care doctor and nurse consultations among people who live in slums: A retrospective, cross-sectional survey in four countries

Improving Health in Slums Collaborative

Corresponding author

Sam Watson

Institute for Applied Health Research, University of Birmingham, Birmingham, United Kingdom

s.i.watson@bham.ac.uk

Word count

3,861 words

Keywords

Healthcare access, health, LMICs, slums, welfare

Data sharing statement

The data are available from the authors on request

Author Contributions

SW and RJL drafted the manuscript and conducted the analyses. Y-FC and J-EP conducted the literature searches and summary in the Research in Context section. All members of the collaborative were involved in the design of the project, data collection, data analysis, and approved the final manuscript.

For peer review only

Conflicts of Interest

None declared.

Acknowledgements

The Improving Health in Slums Collaborative is (in alphabetical order):

African Population and Health Research Centre (APHRC), Nairobi, Kenya
Pauline Bakibinga, Caroline Kabaria, Ziraba Kasiira, Peter Kibe, Catherine Kyobutungi, Nelson Mbaya, Blessing Mberu, Shukri Mohammed, Anne Njeri,

Aga Khan University, Karachi, Pakistan
Iqbal Azam, Romaina Iqbal, Ahsana Nazish, Narijis Rizvi,

Independent University, Bangladesh, Dhaka, Bangladesh
Syed A. K. Shifat Ahmed, Nazratun Choudhury, Ornob Alam, Afreen Zaman Khan, Omar Rahman, Rita Yusuf

Nigerian Academy of Sciences, Lagos, Nigeria
Doyin Odubanjo

University of Ibadan, Ibadan, Nigeria
Motunrayo Ayobola, Olufunke Fayehun, Akinyinka Omigbodun, Mary Osuh, Eme Owoaje, Olalekan Taiwo

University of Birmingham, Birmingham, UK
Richard J Lilford, Jo Sartori, Samuel I Watson

University of Lancaster, Lancaster, UK
Peter J Diggle

University of Warwick, Coventry, UK
Navneet Aujla, Yen-Fu Chen, Christopher Conlan, Paramjit Gill, Frances Griffiths, Bronwyn Harris, Jason Madan, Helen Muir, Oyinlola Oyebode, Vangelis Pitidis, João Porto de Albuquerque, Simon Smith, Celia Taylor, Grant Tregonning, Philip Ulbrich, Olalekan A Uthman, Ria Wilson, Godwin Yeboah, Ji-Eun Park

Patient and public involvement

Mapping of the study sites, identification of healthcare facilities, and enumeration of resident households was conducted using a participatory process involving local residents. Healthcare facility managers and owners were consulted about identification of their facilities. The public were not involved in the design of the survey questionnaires, however feedback was sought from residents in a pilot survey in all sites to assess the time burden of participating. Patient and public

For peer review only - http://bmjopen.bmj.com/site/about/guidelines.xhtml

focus groups were established to present the findings, receive feedback, and provide contextualizing interpretation of the results.

For peer review only

For peer review only - http://bmjopen.bmj.com/site/about/guidelines.xhtml

ABSTRACT

Introduction

Slums or informal settlements characterize most large cities in low and middle income countries (LMIC). Residents of these crowded settlements are thought to have high risk of ill health yet limited information exists on the availability and use of primary care services for this population.

Methods

We conducted household, individual, and healthcare provider surveys in seven slum sites in four countries (Nigeria, Kenya, Pakistan, and Bangladesh).

Results

We completed 7,692 household and 7,451 individual adult and 2,633 individual child surveys across seven sites. The majority of consultations were to doctors/nurses (in clinics or hospitals) and pharmacies rather than single handed providers or traditional healers. Consultation rates with a doctor or nurse varied from 0.2 to 1.5 visits per person-year, which was higher than visit rates to any other type of provider in all sites except Bangladesh, where pharmacies predominated. Approximately half the doctor/nurse visits were in hospital outpatient departments and most of the remainder were to clinics. Over 90% of visits across all sites were for acute symptoms rather than chronic disease. Median travel times were between 15 and 45 minutes and the median cost per visit was between 2 and 10% of a household's monthly total expenditure. Medicines comprised most of the cost. More respondents reported proximity (54-78%) and service quality (31-95%) being a reason for choosing a provider than fees (23-43%). Demand was relatively inelastic with respect to both price of consultation and travel time.

Conclusions

For peer review only

For peer review only - http://bmjopen.bmj.com/site/about/guidelines.xhtml

People in slums tend to live sufficiently close to formal doctor/nurse facilities for their health seeking behavior to be influenced by preference for provider type over distance and cost. However, costs, especially for medicines are high in relation to income and use rates remain significantly below those of high-income countries.

Funding

National Institute for Health Research (16/136/87).

For peer review only

Strength and limitations of this study

- This study provides the most comprehensive evidence across multiple nations to date on the facilities available to people who live in slums in general and on doctor and nurse consultations, in particular.
- We find that across the seven sites, patients most often consulted a doctor or nurse or a pharmacist/medicine seller. Moreover, around half of all doctor/nurse consultations without an overnight stay take place in a local hospital as opposed to a clinic or doctor's office and that traditional/faith healers are relatively infrequently consulted for health needs.
- The cost of outpatient care makes up more than 10% of income in all participating countries except Kenya. Drugs are the greatest cost for a visit, not consultation fees.
- Demand for doctor and nurse care is relatively inelastic with respect to consultation fees and travel time, so reducing travel distances in urban areas is unlikely to impact on service utilisation. Rather, service quality and patient education should be the main aim.

For peer review only - http://bmjopen.bmj.com/site/about/guidelines.xhtml

INTRODUCTION

Strengthening primary care is recognized as the most “inclusive, effective, and efficient approach” to improving population health and wellbeing and as being key to achieving Universal Health Care (UHC) globally as codified in recent international declarations.[1] However, recent high-profile analyses have shown that little systematic evidence has been collected on services that are available, how often they are used, how much they cost and what type of service patients will choose under various constraints.[2–5] It is widely believed though that people in LMIC have poor access to high quality primary care services.[2,3,6–8]

In this paper we focus on a large and vulnerable group of people; those who live in slums. A recent Lancet Series documented the many barriers that people is slums encounter in accessing services.[4,5] Following further review of the literature,[9] most evidence relates to specific programs, particularly those concerned with vaccination and childbirth (e.g.[10]). The little available evidence suggests that in many urban slums, pharmacies and drug sellers are frequently the first and only point of contact with a health system.[11,12] However, consultation with a doctor or nurse is an essential component of primary care providing diagnosis, treatment, advice and referral. Outpatient doctor and nurse consultations can be provided by public and private facilities that range from “single-handed”, sometimes informal, practitioners in solo practices to large hospital outpatient departments.

The purpose of our study is to start to fill the above gaps in the literature, examining outpatient care services in seven slum sites across four countries: Nigeria, Kenya, Pakistan, and Bangladesh. We compare visit rates to all providers that might offer primary care services including traditional/faith healers and pharmacies/medicine sellers. We then describe doctor and nurse services in more detail. Here we aim to find out use rates of different facilities that provide outpatient doctor/nurse consultations for people who live in slums, including solo providers, clinics, hospitals, and public verses private provision. We also aim to estimate how individuals

For peer review only - http://bmjopen.bmj.com/site/about/guidelines.xhtml

trade-off characteristics like cost and time when choosing between types of provider of outpatient care.

METHODS

Setting

Our aim was to examine the use of outpatient consultations with a doctor or nurse. The results reported in this article form part of a broader programme of work on health and healthcare in informal settlements described in detail elsewhere.[13]

We conducted a series of household, individual, and healthcare facility surveys across seven informal settlements in four countries: Nigeria, Kenya, Pakistan, and Bangladesh. We selected sites that (1) fulfilled the UN definition of a 'slum'[14] and (2) were named, geographically distinguishable, neighborhoods within city boundaries. This also provided a geographical basis on which to survey available health services.

Study design and population

Household and individual surveys

The design and methods of the survey components of the Improving Health in Slums project have been published elsewhere.[13] Briefly, we aimed to conduct a spatially-referenced, household-based, retrospective, cross-sectional survey in seven sites, which are described in Table 1. Key indicators for the health systems of the respective countries are reported in Supplementary Information Table 1.

All structures in each site were first mapped using satellite imagery. The resulting maps were then "ground-truthed" through participatory geospatial mapping and corrected as required. During this stage all households in each structure were identified, which formed the sampling frame for the household survey. We used an inhibitory sampling design with close pairs to generate a spatially-

For peer review only - http://bmjopen.bmj.com/site/about/guidelines.xhtml

regular and well-dispersed sample of 1,200 households for each study site,[15] assuming a response rate of 80%. Within each household, all residents were identified and an adult over 18 and a child under 12 were selected at random for the individual surveys. Adult women were oversampled compared to men at a ratio of 2:1 to achieve reasonable precision in both groups as we expected greater healthcare use among women. Up to three attempts were made to complete each survey should the participant not be available at the initial attempt. Due to an error with the sampling process, a follow-up telephone survey was conducted in PK1 to supplement the individual surveys with additional female respondents, which is described in Supplementary Information Section B2.

For peer review only

For peer review only - http://bmjopen.bmj.com/site/about/guidelines.xhtml

Table 1. Summary of study sites

Site	Location	Approx. pop. (000s)*	Approx. density (000s/km^2)	Description of population and area
NG1	Ibadan, Nigeria	5.8	5	Resettled, mixed Yoruba and Hausa community at the edge of the city including a large proportion of recent migrants from the North. Structures are mostly permanent and well-spaced. Variable access to energy with poor sanitation.
NG2	Ibadan, Nigeria	5.5	14	Mostly Yoruba population inhabiting a central, historical part of the city. Building mostly permanent but dilapidated with access to energy but little sanitation.
NG3	Lagos, Nigeria	8.1	11	Mixed Yoruba, Ilajes and other ethnic group population in low-paying or no employment. High crime area with little development and temporary structures. Little access to basic services.
KE1	Nairobi, Kenya	24.4	52	Mixed but segregated ethnicity community of generally long-term multi-generational residents. Structures are temporary with little to no access to basic services.
KE2	Nairobi, Kenya	44.9	83	Mixed community of predominantly economic migrants at the edge of an industrial area. Structures are temporary with little to no access to basic services.
PK1	Karachi, Pakistan	33.5	91	Mixed ethnicity and religion, mostly permanent, population working in blue collar jobs. Structures are permanent and multi-story with access to transient energy and sanitation services.
BD1	Dhaka, Bangladesh	60.0	171	Mostly Bengali, Muslim population working in manual services like rickshaw pulling and house work. Semi-permanent residential structures with variable access to water, sanitation, and other services.

*Estimated from data collected in this study.

The NIHR Global Health Research Unit on Improving Health in Slums was granted full ethical approval by the Ministry of Health, Lagos State Government (LSMH/2695/11/259) the Ministry of Health, Oyo State Government (ADB/479/657), Amref Health Africa (AMREF-ESRC P440/2018), the National Bioethics Committee Pakistan (4-87/NBC-298/18/RDC3530), the Bangladesh Medical Research Council and the University of Warwick Biomedical and Scientific Research Ethics Sub-Committee (REGO-2017-2043 AM01).

Procedures

Three instruments were used in the household surveys: (i) a household level survey of demographic and socioeconomic characteristics, including monthly household expenditures

For peer review only - http://bmjopen.bmj.com/site/about/guidelines.xhtml

across different categories; (ii) an individual adult survey enquiring about health care need, access, and use, and health and well-being; and (iii) a child (under 12 years of age) survey asking a caregiver about health care needs, access, and use. Questions were adapted from similar studies to facilitate comparability.[16] The adult and child surveys both asked the respondents to provide details of the last time they used healthcare (if they had done so in the previous 12 months), including facility type (public clinic, private hospital, etc.), who they saw (doctor, nurse, etc.), the reason for the visit, the cost and time taken for the visit, and questions regarding their satisfaction with the care provided. We included "doctor's office" or "chamber" among the list of responses to facility-type, which generally refers to a solo clinician working alone in a private office – we refer to this category as "single-handed". Survey instruments were translated using an iterative process involving forward and independent backward translations.

Patient and public involvement

Mapping of the study sites, identification of healthcare facilities, and enumeration of resident households was conducted using a participatory process involving local residents. Healthcare facility managers and owners were consulted about identification of their facilities. The public were not involved in the design of the survey questionnaires, however feedback was sought from residents in a pilot survey in all sites to assess the time burden of participating. Patient and public focus groups were established to present the findings, receive feedback, and provide contextualizing interpretation of the results.

Outcomes and Statistical analysis

Summary statistics

From the individual survey data we identified all reported outpatient consultations to any type of provider and calculated visit rates per patient-year for each type of provider (doctor/nurse, pharmacy, traditional/faith, other) for new and existing conditions and for adults and children

For peer review only - http://bmjopen.bmj.com/site/about/guidelines.xhtml

under 12 for comparison. We report crude rates as well as age standardized consultation rates calculated with respect to both the WHO reference population,[17] and INDEPTH population.[18] For doctor and nurse visits we further examined the proportion of visits by facility type (single-handed, public/private hospital, or public/private clinic) and reported provider type (doctor or nurse).

We estimated key characteristics of these visits including the median [Interquartile range (IQR)] time required for the visit, median [IQR] cost of the visit, proportion of households spending more than 10% of their monthly expenditure on a visit (as used by the WHO's Global Health Observatory), reason for choosing the provider, and satisfaction by site and facility type.

Choice model

To estimate the role of price and travel time in the choice over a provider for a doctor or nurse for an outpatient consultation, we estimated a 'choice model'. We specified a random parameters logit model,[19] which we describe in detail in the Supplementary Information. In the model, each option from a set of choices has an observed component (price and time), the utility of which is determined by observed (e.g. age, sex) and unobserved (e.g. taste, quality) characteristics. There are several examples of choice modelling for healthcare providers in LMICs (for example, Borah (2006)[20]). The "choice set" we examined was: private clinic, public clinic, private hospital, or public hospital. We also included "single-handed" doctor's office for Bangladesh and Pakistan, since this type of consultation was rare or non-existent in our African sites.

We studied each site separately. There were four estimates of interest from each model: the predicted proportion of visits to each provider type "holding fixed" price and travel time, the price elasticity of demand, the travel time elasticity of demand, and the average change in price (willingness to pay, WTP) to make an individual indifferent between two options, one of which is 15 minutes further travel away than the other. The elasticities and WTP were calculated separately for households whose monthly consumption expenditure was above and below Int$

For peer review only - http://bmjopen.bmj.com/site/about/guidelines.xhtml

100 per person per month. An elasticity is interpreted as the percentage change in demand you would expect for a 1% increase in price or travel time. We allowed preferences to vary by age, sex, secondary education, seeking care for an acute or communicable condition, or for chronic or generalized pain, and monthly consumption expenditure. The "price" of a visit included the consultation fee plus the travel cost, which would be known up front to the individual, and not drugs or tests, which would not be known in advance. The prices and times of travel for providers not visited were imputed based on the above listed individual covariates.

For peer review only

For peer review only - http://bmjopen.bmj.com/site/about/guidelines.xhtml

RESULTS

Household and Individual Sample

Overall, 7,692 households participated in the surveys with 7,451 individual adults and 2,633 individual child surveys completed. The median response rate was 69%, varying by site from 57% in Site KE2 to 94% in Site BD1. Table 2 reports demographic and socioeconomic statistics of the population-weighted sample of individual respondents by site, and for those reporting an outpatient consultation (population summaries are reported in Table C2, Supplementary Information).

Outpatient consultation rates across providers of all types

Figure 1 shows the visit rates per patient-year to different types of provider. Between 29% (BD1) and 61% (KE1) of visits were to doctors and nurses, while the majority of other visits were to a pharmacy. In only two sites (BD1 and KE2) were pharmacies visited more frequently than doctors or nurses. The proportion of outpatient consultations that were for new conditions ranged from 61% (NG2) to 84% (KE2). Proportionately very few healthcare visits were made to traditional or faith healers.

Outpatient consultation rates to a doctor or nurse

Table 3 reports the outpatient consultation rates by study site for both adults and children (under 12 and under 5) – equivalent rates for new conditions only are reported in Table C3 Supplementary Information. Nigerian sites had consistently the lowest outpatient consultation rates, which were comparable for adults and children: approximately 0.2 to 0.4 outpatient visits to a doctor or nurse for a new condition per patient-year. Rates were higher in other sites, ranging, for adults, from approximately 0.8 (PK1) to 1.5 (BD1) visits per patient-year. Apart from Nigeria, outpatient consultation rates were higher for children than adults.

For peer review only - http://bmjopen.bmj.com/site/about/guidelines.xhtml

TABLE 2 Summary statistics of the overall study sample (All) and those reporting a doctor or nurse (D/N) consultation for a new condition visit by site. Values are mean (sd) unless otherwise stated.

Variable		Nigeria						Kenya				Pakistan		Bangladesh	
		NG1		NG2		NG3		KE1		KE2		PK1		BD1	
		All	D/N	All	D/N	All	D/N	All	D/N	All	D/N	All	D/N	All	D/N
Adults															
N		1,278	391	840	215	802	201	1,008	405	1,085	377	1,112	498	990	257
Household size		3.6 (1.8)	3.8 (1.8)	4.7 (1.4)	4.9 (1.3)	4.6 (1.8)	4.7 (1.8)	3.5 (2.1)	3.1 (2.0)	2.5 (1.4)	2.4 (1.3)	5.5 (2.6)	5.5 (2.5)	4.3 (1.9)	4.4 (2.0)
Wealth quintile (%)	Bottom	0	0	0	0	0	0	0	0	0	0	0	0	0	0
	Lower	2	3	3	0	1	1	15	13	0	0	0	0	0	0
	Middle	49	48	51	47	24	24	59	61	55	54	83	83	21	19
	Upper	48	49	46	53	75	75	25	26	44	45	15	15	78	77
	Top	0	0	0	0	0	0	0	0	1	1	3	2	1	3
Monthly household expenditure (Int$)	Total	300 (248)	362 (250)	342 (274)	349 (268)	560 (364)	580 (363)	232 (136)	213 (127)	243 (127)	274 (152)	1,122 (631)	1,179 (667)	463 (234)	505 (257)
	Per person	99 (86)	108 (79)	95 (78)	101 (87)	136 (101)	142 (104)	84 (62)	87 (63)	118 (81)	130 (92)	223 (125)	230 (138)	116 (58)	126 (61)
Age		39.0 (16.7)	39.9 (17.4)	47.8 (16.6)	51.3 (17.7)	42.0 (15.0)	43.8 (15.2)	37.6 (13.6)	40.1 (14.7)	34.3 (11.1)	34.4 (11.0)	37.6 (12.6)	39.2 (13.1)	34.6 (12.3)	36.6 (12.7)
Sex (% male)		45	35	49	50	47	45	46	34	46	42	50	42	53	41
Education (%)	Primary/middle	22	19	34	36	16	19	60	55	39	38	7	10	61	58
	Secondary	46	52	55	52	54	49	34	38	51	49	79	76	31	30
	Tertiary	24	22	9	9	32	31	6	7	8	12	14	14	8	12
Children (under 12)															
N		128	36	69	13	79	24	537	320	421	205	528	367	658	136
Household size		4.9 (1.5)	4.9 (1.4)	4.7 (1.4)	4.9 (1.3)	4.9 (1.4)	5.1 (1.4)	5.2 (1.8)	5.2 (1.9)	4.4 (1.4)	4.3 (1.4)	7.5 (3.0)	7.4 (2.9)	4.9 (1.6)	5.4 (1.8)
Wealth quintile (%)	Lowest	0	0	0	0	0	0	0	0	0	0	0	0	0	0
	Lower	2	1	3	0	1	0	13	13	0	0	0	0	0	0
	Middle	48	62	51	47	25	37	60	56	55	55	84	85	25	20
	Upper	50	37	46	53	74	63	27	30	44	45	15	14	74	78
	Top	0	0	0	0	0	0	0	1	0	0	1	1	1	2
Monthly household expenditure (Int$)	Total	365 (315)	359 (227)	437 (310)	417 (254)	579 (336)	626 (386)	284 (29)	274 (152)	276 (132)	286 (143)	1,304 (733)	1,324 (740)	472 (235)	525 (252)
	Per person	74 (58)	75 (49)	93 (60)	92 (59)	123 (68)	130 (75)	56 (29)	130 (92)	69 (37)	70 (36)	184 (112)	194 (117)	100 (46)	104 (50)
Age		6.9 (3.6)	6.0 (3.4)	6.7 (3.7)	4.9 (3.3)	6.5 (3.9)	6.2 (3.8)	5.9 (3.7)	5.5 (3.8)	5.6 (3.6)	5.1 (3.7)	6.0 (3.7)	5.4 (3.4)	5.5 (3.7)	4.7 (3.7)
Sex (% male)		44	33	57	51	43	40	55	52	48	46	53	53	52	62

For peer review only - http://bmjopen.bmj.com/site/about/guidelines.xhtml

Doctor and nurse consultation rates by provider type

Figure 2 shows the proportion of outpatient consultations for a new condition by facility type. There were differences between the sites and countries. Single-handed facilities accounted for approximately 25% of adult visits in Bangladesh and Pakistan, and 50% and 20% of child visits in these countries, respectively. However, almost no visits to single-handed facilities were recorded in Nigeria and Kenya. Hospital outpatient departments and clinics accounted for comparable shares of outpatient consultations; in particular, for adults, hospital visit shares were 51% (NG1), 66% (NG2), 69% (NG3), 38% (KE1), 39% (KE2), 37% (PK1), and 21% (BD1). These figures were similar for children. Figure 3 shows the proportion of visits by provider (doctor or nurse). For the Pakistani and Bangladeshi communities, almost all outpatient consultations were with a doctor, whereas in Kenya and Nigeria a significant minority of consultations were with a nurse for both adults and children.

For peer review only - http://bmjopen.bmj.com/site/about/guidelines.xhtml

Table 3. Outpatient primary care consultation rates and outpatient doctor and nurse consultation rates (visits per person-year (95% CI)).

Site	Outpatient consultation (all conditions)		
	**Crude**	**WHO age adjusted**	**INDEPTH age adjusted**
	Adults		
NG1	0.42 (0.40, 0.44)	0.42 (0.40, 0.44)	0.40 (0.38, 0.42)
NG2	0.39 (0.35, 0.43)	0.32 (0.30, 0.34)	0.29 (0.27, 0.31)
NG3	0.28 (0.26, 0.30)	0.31 (0.29, 0.33)	0.29 (0.27, 0.31)
KE1	1.07 (1.03, 1.11)	1.17 (1.13, 1.21)	1.04 (1.00, 1.08)
KE2	0.93 (0.89, 0.97)	1.06 (1.02, 1.10)	0.95 (0.91, 0.99)
PK1	0.79 (0.75, 0.83)	0.85 (0.81, 0.89)	0.77 (0.73, 0.81)
BD1	1.52 (1.46, 1.58)	1.73 (1.67, 1.79)	1.59 (1.53, 1.65)
	Children (under 12)		
NG1	0.29 (0.19, 0.39)	-	-
NG2	0.15 (0.05, 0.25)	-	-
NG3	0.34 (0.20, 0.48)	-	-
KE1	1.74 (1.62, 1.86)	-	-
KE2	1.30 (1.18, 1.42)	-	-
PK1	1.85 (1.73, 1.97)	-	-
BD1	1.04 (0.96, 1.12)	-	-
	Children (under 5)		
NG1	0.30 (0.14, 0.46)	-	-
NG2	0.21 (0.03, 0.39)	-	-
NG3	0.40 (0.16, 0.64)	-	-
KE1	2.57 (2.35, 2.79)	-	-
KE2	1.68 (1.50, 1.86)	-	-
PK1	2.46 (2.24, 2.68)	-	-
BD1	1.50 (1.36, 1.63)	-	-

For peer review only

For peer review only - http://bmjopen.bmj.com/site/about/guidelines.xhtml

Choice of providers of doctor and nurse consultations

Table 4 reports travel times, waiting times, and travel, drug, tests and other costs for the different types of facility for doctor and nurse consultations. Within each site the travel time to reach each type of facility was broadly similar with median travel times generally ranging from 15 to 30 minutes for all types of facility (see also Figure C1, Supplementary Information).

Bangladesh and Nigeria were the most expensive locations to seek treatment, both in relative and absolute terms, with median spending ranging from Int$ 21 to 82 depending on facility type. Median spending in Kenyan facilities ranged from Int$ 6 to 15, and in Pakistan Int$ 16 to 42. Medication costs accounted the bulk of the cost of an outpatient consultation in all sites: the median proportion of the total cost of a visit accounted for by drugs was 67% to 100%. Consultation fees in the Nigerian and Kenyan sites were generally under Int$5 and often zero, whereas in Pakistan and Bangladesh they were higher (approximately Int$5 to 15). The median expenditure for a visit was less than 10% of total monthly household expenditure for almost all types of consultation across all seven sites. However, the costs were highly skewed so that in all countries except Kenya over a third of consultations would constitute more than a third of a person's total monthly household expenditure.

Table 5 reports the results from the choice model. All elasticities were below zero, showing people prefer less costly and nearer services. However, the mean estimated elasticities were almost all between zero and minus one, with the exception of in Kenya, suggesting demand was relatively inelastic with respect to both price and time. Figure 4 compares predicted share of visits to different providers, net of costs and travel times, to actual proportions of visits: there was little qualitative difference between the two. This expressed choice is similar to survey responses; only 23-43% of respondents reported "low fees" being a reason they chose a provider (Table C4, Supplementary Information). Demand was generally more elastic in poorer households (Table 5).

For peer review only - http://bmjopen.bmj.com/site/about/guidelines.xhtml

Table 4. Summary of time and costs of outpatient doctor and nurse consultations by study site for adults and children combined. All values are median [IQR] if the number of recorded visits N was five or more.

Variable		Facility type				
		Single-handed	Clinics		Hospitals	
			Private	Public/NGO	Private	Public/NGO
NG1						
Travel time (mins)		-	15 [5, 30]	15 [10, 20]	15 [10, 30]	15 [10, 30]
Waiting time (mins)		-	5 [2, 10]	10 [5, 40]	5 [2, 10]	30 [10, 60]
Costs (Int$)	Consultation	-	0 [0, 3]	0 [0, 2]	0 [0, 8]	0 [0, 3]
	Drugs	-	17 [4, 73]	15 [8, 23]	21 [10, 42]	17 [10, 25]
	Tests	-	0 [0, 7]	1 [0, 4]	2 [0, 8]	2 [0, 8]
	Travel	-	0 [0, 4]	0 [0, 5]	2 [0, 8]	2 [0, 10]
	Total	-	42 [5, 88]	21 [13, 33]	42 [21, 61]	23 [14, 42]
Total cost as % of monthly h/h expenditure		-	9 [2, 21]	7 [3, 15]	11 [6, 19]	9 [4, 16]
Households spending >10% of monthly expenditure on visit (%)		-	43	32	56	43
NG2						
Travel time (mins)		-	20 [12, 44]	10 [5, 20]	15 [10, 30]	20 [10, 30]
Waiting time (mins)		-	30 [12, 55]	15 [5, 30]	10 [5, 30]	30 [13, 60]
Costs (Int$)	Consultation	-	0 [0, 1]	0 [0, 1]	0 [0, 0]	0 [0, 4]
	Drugs	-	5 [1, 11]	15 [8, 29]	20 [8, 42]	17 [11, 29]
	Tests	-	0 [0, 0]	0 [0, 4]	0 [0, 8]	0 [0, 8]
	Travel	-	0 [0, 0]	0 [0, 4]	0 [0, 8]	0 [0, 8]
	Total	-	13 [9, 32]	20 [13, 34]	29 [13, 78]	29 [17, 42]
Total cost as % of monthly h/h expenditure		-	11 [6, 46]	5 [3, 13]	11 [4, 23]	13 [5, 30]
Households spending >10% of monthly expenditure on visit (%)		-	50	32	56	55
NG3						
Travel time (mins)		-	22 [10, 41]	10 [9, 20]	20 [14, 30]	30 [10, 40]
Waiting time (mins)		-	10 [5, 12]	20 [5, 50]	10 [5, 30]	46 [10, 120]
Costs (Int$)	Consultation	-	0 [0, 0]	1 [0, 2]	0 [0, 8]	0 [0, 4]
	Drugs	-	25 [12, 42]	13 [8, 22]	42 [21, 49]	17 [8, 30]
	Tests	-	0 [0, 19]	0 [0, 4]	0 [0, 13]	7 [0, 17]
	Travel	-	0 [0, 0]	0 [0, 2]	2 [0, 3]	2 [0, 4]
	Total	-	56 [29, 81]	20 [11, 34]	47 [33, 81]	34 [17, 60]
Total cost as % of monthly h/h expenditure		-	10 [3, 16]	4 [2, 9]	9 [4, 15]	7 [3, 12]
Households spending >10% of monthly expenditure on visit (%)		-	50	17	43	33
KE1						
Travel time (mins)		-	10 [5, 20]	15 [10, 30]	20 [10, 30]	28 [10, 45]
Waiting time (mins)		-	30 [5, 42]	60 [18, 94]	20 [5, 42]	30 [20, 120]
Costs (Int$)	Consultation	-	0 [0, 2]	0 [0, 0]	0 [0, 2]	0 [0, 1]
	Drugs	-	9 [2, 13]	5 [2, 9]	7 [2, 18]	6 [0, 9]
	Tests	-	2 [0, 6]	0 [0, 3]	0 [0, 6]	0 [0, 6]
	Travel	-	2 [0, 5]	0 [0, 2]	2 [0, 3]	1 [0, 4]
	Total	-	13 [8, 30]	6 [2, 12]	11 [4, 30]	8 [2, 24]
Total cost as % of monthly h/h expenditure		-	5 [1, 21]	3 [1, 11]	5 [1, 11]	3 [1, 11]
Households spending >10% of monthly expenditure on visit (%)		-	43	27	29	26
KE2						
Travel time (mins)		-	18 [10, 30]	20 [15, 30]	30 [15, 50]	35 [30, 58]
Waiting time (mins)		-	10 [5, 20]	30 [10, 60]	15 [5, 30]	40 [20, 88]
Costs (Int$)	Consultation	-	0 [0, 1]	0 [0, 1]	0 [0, 3]	1 [0, 3]

For peer review only - http://bmjopen.bmj.com/site/about/guidelines.xhtml

	Drugs	-	8 [4, 14]	6 [2, 11]	9 [5, 19]	11 [7, 28]
	Tests	-	3 [0, 6]	0 [0, 2]	9 [0, 19]	6 [2, 11]
	Travel	-	1 [0, 2]	1 [0, 1]	2 [1, 4]	2 [2, 4]
	Total	-	9 [4, 19]	6 [4, 13]	13 [6, 31]	15 [4, 48]
Total cost as % of monthly h/h expenditure		-	3 [1, 9]	3 [1, 8]	4 [1, 14]	5 [1, 15]
Households spending >10% of monthly expenditure on visit (%)		-	19	21	29	31
PK1						
Travel time (mins)		10 [5, 15]	5 [5, 10]	20 [14, 30]	15 [10, 30]	30 [15, 30]
Waiting time (mins)		10 [0, 25]	10 [10, 20]	52 [12, 98]	25 [10, 60]	30 [14, 60]
Costs (Int$)	Consultation	3 [3, 5]	3 [3, 6]	0 [0, 1]	6 [1, 29]	0 [0, 0]
	Drugs	12 [6, 16]	9 [6, 19]	12 [4, 16]	16 [5, 44]	16 [0, 31]
	Tests	0 [0, 0]	0 [0, 0]	0 [0, 0]	0 [0, 0]	0 [0, 0]
	Travel	0 [0, 0]	0 [0, 0]	1 [0, 2]	2 [0, 6]	2 [0, 6]
	Total	18 [11, 25]	16 [9, 24]	17 [11, 25]	42 [18, 104]	19 [7, 55]
Total cost as % of monthly h/h expenditure		1 [1, 4]	2 [1, 3]	1 [0, 3]	2 [4, 9]	2 [0, 4]
Households spending >10% of monthly expenditure on visit (%)		6	9	8	25	12
BD1						
Travel time (mins)		20 [15, 30]	30 [20, 60]	30 [20, 30]	35 [19, 60]	30 [24, 60]
Waiting time (mins)		45 [30, 90]	60 [30, 90]	85 [30, 128]	20 [19, 30]	60 [30, 120]
Costs (Int$)	Consultation	12 [6, 15]	12 [1, 15]	0 [0, 0]	0 [0, 4]	0 [1, 4]
	Drugs	26 [15, 47]	32 [14, 61]	15 [5, 24]	27 [20, 60]	21 [9, 41]
	Tests	21 [8, 46]	31 [0, 61]	11 [1, 20]	6 [6, 6]	31 [9, 48]
	Travel	2 [2, 3]	2 [1, 3]	3 [2, 5]	2 [2, 2]	3 [2, 6]
	Total	52 [30, 89]	82 [26, 128]	22 [15, 39]	36 [32, 62]	45 [18, 96]
Total cost as % of monthly h/h expenditure		13 [6, 23]	17 [5, 32]	6 [2, 10]	11 [10, 16]	9 [3, 17]
Households spending >10% of monthly expenditure on visit (%)		58	67	28	75	44

The most frequently cited reasons for choosing a particular healthcare facility and provider were proximity (54-78% of respondents), service quality (31-95%) and cordiality (21-65%) (Table C4 Supplementary Information). Generally, the majority of respondents were either “Very satisfied” or “satisfied” with their visit and rated most aspects of the visit either “Very good” or “good”, aside from waiting time where negative responses were more common. Respondents were more likely to be “Very satisfied” with care at public clinics in four of the seven sites, with comparable satisfaction with other providers in the remaining sites. Respondents were also least likely to report “very satisfied” for single-handed facilities and private clinics (see Figure C2, Supplementary Information). The most common medical reason for seeking care was “communicable” or “acute conditions” (35-69%) with only 2 to 6% of respondents reporting a chronic condition as the reason for the visit (Figure C5, Supplementary Information).

For peer review only

For peer review only - http://bmjopen.bmj.com/site/about/guidelines.xhtml

Table 5. Price and travel time elasticities of demand and willingness to pay for nearer services for households by total monthly per person household consumption expenditure, values are posterior mean (95% credible intervals)

	Price elasticity of demand		Time elasticity of demand		Willingness to pay for 15 minutes less travel time (Int$)	
	<Int$100 pppm	*≥Int$100 pppm*	*<Int$100 pppm*	*≥Int$100 pppm*	*<Int$100 pppm*	*≥Int$100 pppm*
NG1	-0.62 (-0.89, -0.36)	-0.27 (-0.46, -0.10)	-1.01 (-1.76, -0.26)	-0.47 (-1.04, -0.18)	8.45 (-0.95, 22.69)	7.20 (1.94, 14.51)
NG2	-0.96 (-1.43, -0.49)	-0.47 (-0.75, -0.17)	-0.29 (-0.96, 0.42)	-0.36 (-0.88, -0.21)	1.35 (-2.07, 5.03)	3.00 (-2.06, 9.49)
NG3	-0.45 (-1.23, 0.23)	-0.03 (-0.53, 0.43)	0.00 (-0.84, 1.02)	0.00 (-0.61, 0.78)	-2.39 (-39.37, 41.45)	-2.87 (-27.97, 33.16)
KE1	-1.92 (-2.46, -1.26)	-0.62 (-1.18, -0.06)	-0.34 (-0.94, 0.22)	-0.16 (-0.63, 0.31)	1.15 (-0.39, 3.25)	1.50 (-1.23, 5.44)
KE2	-1.94 (-2.44, -1.26)	-1.49 (-1.96, -0.89)	-0.27 (-0.87, 0.42)	-0.27 (-0.81, 0.40)	1.13 (-1.08, 3.60)	1.03 (-0.86, 3.10)
PK1	-0.30 (-1.72, 1.05)	-0.69 (-1.31, -0.04)	-1.52 (-2.70, -0.32)	-0.98 (-1.61, -0.30)	6.93 (2.03, 16.04)	6.19 (2.13, 12.65)
BD1	-0.79 (-2.38, 0.72)	-0.28 (-1.63, 0.76)	-1.09 (-1.88, -0.36)	-0.21 (-0.59, 0.17)	12.20 (-46.93, 72.03)	7.03 (-18.30, 34.48)

For peer review only - http://bmjopen.bmj.com/site/about/guidelines.xhtml

DISCUSSION

We found that people living in slums make use of a range of primary care providers, both public and private, and from individual clinicians to hospital outpatient departments. While there are considerable differences between slums and countries, we found across all sites that: traditional healers were seldom consulted for health needs, reflecting previous evidence;[21] doctors and nurses were frequently consulted, more so than pharmacies in all sites other than Bangladesh; formal clinics/hospital outpatient departments were more popular than “single-handed” settings; the costs of medication exceeded those of consultations; and, demand was relatively inelastic with respect to consultancy and travel cost.

The various providers of doctor and nurse consultations were all in relative proximity to the slum precinct; the majority of respondents reported requiring less than 30 minutes to reach their provider of choice. It is noteworthy that relatively frequent use was made of hospital outpatient departments, which accounted for as much as 50% of doctor or nurse outpatient consultations in some sites. We also found low use of individually owned and operated practices. This is in contrast to what we expected, perhaps because of the substantial literature on quality of care in “single-handed” practices, much of it emanating from India.[3,6,7,22]

There was evidence that many households spent a significant proportion of their money on healthcare. While we found evidence that the up-front cost of a visit, including consultancy fees and travel costs, were relatively low and had only a small effect on demand for services, the total cost of a visit was much higher and often differed little between public and private facilities. The WHO’s Global Health Observatory use the proportion of households spending more than 10% of their expenditure on healthcare as a marker of national health system performance and report figures of 15%, 5%, 13%, and 25% for Nigeria, Kenya, Pakistan, and Bangladesh, respectively (Table A1, Supplementary). The relative differences between countries reflect those found in this study. In both data sources, Kenya has the lowest rates of high spending relative to household

For peer review only - http://bmjopen.bmj.com/site/about/guidelines.xhtml

spending which may be attributed to the National Hospital Insurance Fund. Nevertheless, our results suggest the proportion of slum households meeting the 10% threshold is much higher (generally more than 50%) than the above national averages.

Most of the cost of a primary care visit was accounted for by the cost of drugs, which reflects findings from another LMIC-based study that showed high mark ups on medication.[23] Kenya is the only country in our study with any public funding for medication, and while respondents there did report the lowest cost to access care of any of our sites, drugs were still the biggest source of expenditure.[24] Much of the literature on healthcare access in LMICs has focused on the deleterious effect of user and consultation fees, and it has been shown that even very small costs can suppress demand and do so non-selectively and to the detriment of children (e.g.[25]). However, our evidence suggests that the costs of treatment may be a larger problem for access to care in urban areas.

Patient choice

The finding that people will frequently attend a formal facility such as a hospital outpatient department, bypassing other facilities/providers on the way, shows that people are likely willing to trade convenience and cost for perceptions of quality. We have quantified and explored these trade-offs formally by use of a 'choice model'. An immediate limitation is that we are not 'comparing like for like' since we must assume that demand for a provider type is heavily determined by the type of symptom a person is experiencing. That said, it must also be assumed that when the situation is perceived to be more serious, the more a person will eschew local in favor of more distant providers. In that sense our findings are all the more impressive, representing an underestimate of preference for formal providers when the complaint is more serious. Nevertheless, higher elasticities among the poorest people is a cause for concern.

Overall demand for health care

For peer review only - http://bmjopen.bmj.com/site/about/guidelines.xhtml

Despite the availability of a range of providers, and study participants reporting that they were able to access health care when they needed it, age adjusted use rates in our study sites were substantially lower than in high income countries despite a high burden of disease. In Nigeria, respondents consulted a doctor or nurse once every three years to once every 6 to 9 months in Bangladesh. Yet in the United States the crude consultation rate was 3.9 visits per person-year,[26] and the United Kingdom it was 5.5 visits per person-year.[27] Not only is use in our study population lower than in HIC, but there is evidence that health needs are not being met. For instance, very few visits were reported for chronic conditions and follow-up despite the prevalence of chronic illness, which has been reported to be high in other slum populations.[28] Lack of consultation for symptoms that do not cause immediate distress may be a reason that patients presenting with cancer in LMICs are much more likely than in HICs to be in stage 3 or 4.[29,30]

Our findings, interpreted in the context of the literature, provide the basis for an emerging policy to improve access to high-quality care in urban areas. First, while in rural areas the predominant consideration is often the existence of local services, in urban areas there are a wide variety of services in close proximity. The imperative in urban areas should therefore be to make better use of the services that already exist. For example, we have found a relatively high use of pharmacies rising to two thirds of all health care contacts in Bangladesh.[11] This suggests, for example, that they might provide a good focus for dissemination of preventive health advice.

Second, our findings on expressed preference show that, in the context of the city, distance to facilities does not significantly suppress demand. These observations suggests that it might be a mistake to pursue, in urban areas, a policy to ensure yet closer location of many services to where people live. Such services would inevitably be more dispersed into small and “single-handed” providers where care has been shown to be of low quality (even when the practitioners are medically qualified[6]), and that training has little effect on improving care quality.[7]

For peer review only - http://bmjopen.bmj.com/site/about/guidelines.xhtml

Third, overall use is low while people with serious disease such as cancer and TB present late. At the supply side this should be tackled by improving care quality, such as improved diagnosis, as suggested above and also by mitigating the main cost – namely medicines. On the demand side there is a need to continue research into barriers to appropriate health seeking for symptoms of serious disease.

Limitations

The response rates differed by study site. We made up to three callbacks for each sampled household to minimize selection bias, but non-responsive or non-consenting households may have differed from those that participated. The age standardized consultation rates were based on the INDPETH and WHO reference populations[17] enabling us to make age-adjusted comparisons between study sites. However, the reference population may be out of date and may not be the most appropriate population for broader international comparisons. However, estimated rates differed little between reference populations. Clinical officers were not included in this study as a category, and we do not how respondents might identify or categorise them (e.g. as a "doctor" or "other"); further research is required into the role of clinical officers in these locations. We cannot make a reliable comparison of individuals based on their health status as no reliable clinical or epidemiological indicators were captured. Differences between sites may well be attributable to large differences in population health, however we suggest that this would be unlikely. Any observed relationships, or lack thereof, may be driven by other underlying processes and confirmatory studies are needed to address some of the questions raised here. Finally, slum populations are often highly mobile, which could result in rapidly shifting population characteristics. The cross-sectional data here may therefore not provide a complete picture of slums around the world and we caution against making broad generalizations.

Conclusion

For peer review only - http://bmjopen.bmj.com/site/about/guidelines.xhtml

There have been big ‘pro-poor’ improvements in key indicators, such as infant and under-five mortality, in the last 20 years. But these have been largely achieved by preventive services such as immunization, improved nutrition and rehydration therapy. Further improvements, for example in cancer care, will require improved clinical care along the pathways from seeking care for the first symptom to definitive treatment. We hope that our findings contribute to the debate about how to improve primary care services by working with the grain of peoples’ preferences.

For peer review only

REFERENCES

1 WHO; UNICEF Declaration of Astanap.8-9. Declaration of Astana. *Glob Conf Prim Heal Care* 2018.

2 Kruk ME, Gage AD, Arsenault C, *et al.* High-quality health systems in the Sustainable Development Goals era: time for a revolution. *Lancet Glob Heal* 2018; **6**: e1196–252.

3 McPake B, Hanson K. Managing the public-private mix to achieve universal health coverage. *Lancet (London, England)* 2016; **388**: 622–30.

4 Ezeh A, Oyebode O, Satterthwaite D, *et al.* The history, geography, and sociology of slums and the health problems of people who live in slums. *Lancet* 2016; published online Oct. DOI:10.1016/S0140-6736(16)31650-6.

5 Lilford RJ, Oyebode O, Satterthwaite D, *et al.* Improving the health and welfare of people who live in slums. *Lancet* 2017; **389**: 559–70.

6 Das J. The Quality of Medical Care in Low-Income Countries: From Providers to Markets. *PLoS Med* 2011; **8**: e1000432.

7 Das J, Chowdhury A, Hussam R, Banerjee A V. The impact of training informal health care providers in India: A randomized controlled trial. *Science (80-)* 2016; **354**: aaf7384–aaf7384.

8 Kruk ME, Gage AD, Joseph NT, Danaei G, García-Saisó S, Salomon JA. Mortality due to low-quality health systems in the universal health coverage era: a systematic analysis of amenable deaths in 137 countries. *Lancet* 2018; **392**: 2203–12.

9 Chen Y-F, Park J-E, Harris B, Ajisola M, Griffiths F, Improving Health in Slums Collaborative. Scoping review protocol: Factors associated with accessing health care and provision of health services for residents of slums in low and middle income countries. 2021 https://osf.io/694t2.

10 Crocker-Buque T, Mindra G, Duncan R, Mounier-Jack S. Immunization, urbanization and slums – a systematic review of factors and interventions. *BMC Public Health* 2017; **17**: 556.

11 Khan MMH, Grübner O, Krämer A. Frequently used healthcare services in urban slums of

For peer review only - http://bmjopen.bmj.com/site/about/guidelines.xhtml

Dhaka and adjacent rural areas and their determinants. *J Public Health (Bangkok)* 2012; **34**: 261–71.

12 Owusu-Ansah FE, Tagbor H, Togbe MA. Access to health in city slum dwellers: The case of Sodom and Gomorrah in Accra, Ghana. *African J Prim Heal Care Fam Med* 2016; **8**. DOI:10.4102/phcfm.v8i1.822.

13 Improving Health in Slums Collaborative. A protocol for a multi-site, spatially-referenced household survey in slum settings: methods for access, sampling frame construction, sampling, and field data collection. *BMC Med Res Methodol* 2019; **19**: 109.

14 UN Habitat. The Challenge of the Slums: Global Report on Human Settlements 2003. London, UK, 2010.

15 Chipeta M, Terlouw D, Phiri K, Diggle P. Inhibitory geostatistical designs for spatial prediction taking account of uncertain covariance structure. *Environmetrics* 2017; **28**: e2425.

16 World Health Organization. WHO World Health Survey. 2007. https://www.who.int/healthinfo/survey/en/ (accessed March 6, 2020).

17 Ahmad OB, Boschi-pinto C, Lopez AD. Age standardization of rates: a new WHO standard. *GPE Discuss Pap Ser* 2001.

18 Sankoh O, Sharrow D, Herbst K, *et al.* The INDEPTH standard population for low- and middle-income countries, 2013. *Glob Health Action* 2014; **7**: 23286.

19 Fox JT, Kim K il, Ryan SP, Bajari P. The random coefficients logit model is identified. *J Econom* 2012; **166**: 204–12.

20 J. Borah B. A mixed logit model of health care provider choice: analysis of NSS data for rural India. *Health Econ* 2006; **15**: 915–32.

21 Oyebode O, Kandala N-B, Chilton PJ, Lilford RJ. Use of traditional medicine in middle-income countries: a WHO-SAGE study. *Health Policy Plan* 2016; **31**: 984–91.

22 Coarasa J, Das J, Gummerson E, Bitton A. A systematic tale of two differing reviews: Evaluating the evidence on public and private sector quality of primary care in low and middle income countries. *Global Health* 2017. DOI:10.1186/s12992-017-0246-4.

23 Cameron A, Ewen M, Ross-Degnan D, Ball D, Laing R. Medicine prices, availability, and affordability in 36 developing and middle-income countries: a secondary analysis. *Lancet* 2009; **373**: 240–9.

24 Bitton A, Fifield J, Ratcliffe H, *et al.* Primary healthcare system performance in low-income and middle-income countries: a scoping review of the evidence from 2010 to 2017. *BMJ Glob Heal* 2019; **4**: e001551.

25 Watson SI, Wroe EB, Dunbar EL, *et al.* The impact of user fees on health services utilization and infectious disease diagnoses in Neno District, Malawi: a longitudinal, quasi-experimental study. *BMC Health Serv Res* 2016; **16**: 595.

26 OECD. Doctor's Consultations. OECD Data. 2020. https://data.oecd.org/healthcare/doctors-consultations.htm (accessed March 6, 2020).

27 Hobbs FDR, Bankhead C, Mukhtar T, *et al.* Clinical workload in UK primary care: a retrospective analysis of 100 million consultations in England, 2007-14. *Lancet (London,*

For peer review only

For peer review only - http://bmjopen.bmj.com/site/about/guidelines.xhtml

England) 2016; **387**: 2323–30.

28 McNairy ML, Tymejczyk O, Rivera V, *et al.* High Burden of Non-communicable Diseases among a Young Slum Population in Haiti. *J Urban Heal* 2019; **96**: 797–812.

29 Rivera-Franco MM, Leon-Rodriguez E. Delays in Breast Cancer Detection and Treatment in Developing Countries. *Breast Cancer Basic Clin Res* 2018; **12**: 117822341775267.

30 Stewart TS, Moodley J, Walter FM. Population risk factors for late-stage presentation of cervical cancer in sub-Saharan Africa. *Cancer Epidemiol* 2018; **53**: 81–92.

Figure legends

Figure 1. Age-adjusted (to INDEPTH population) visit rates per person-year to different outpatient care providers for adults and children (under 12) for new and existing conditions.

Figure 2. Proportion of outpatient doctor and nurse consultations for a new condition by facility type

Figure 3. Proportion of outpatient consultations for a new condition by doctor or nurse

Figure 4. Predicted proportions of visits to different provider types for an outpatient doctor and nurse consultation if price and travel time were all equivalent versus actual proportions of visits.

For peer review only

For peer review only - http://bmjopen.bmj.com/site/about/guidelines.xhtml

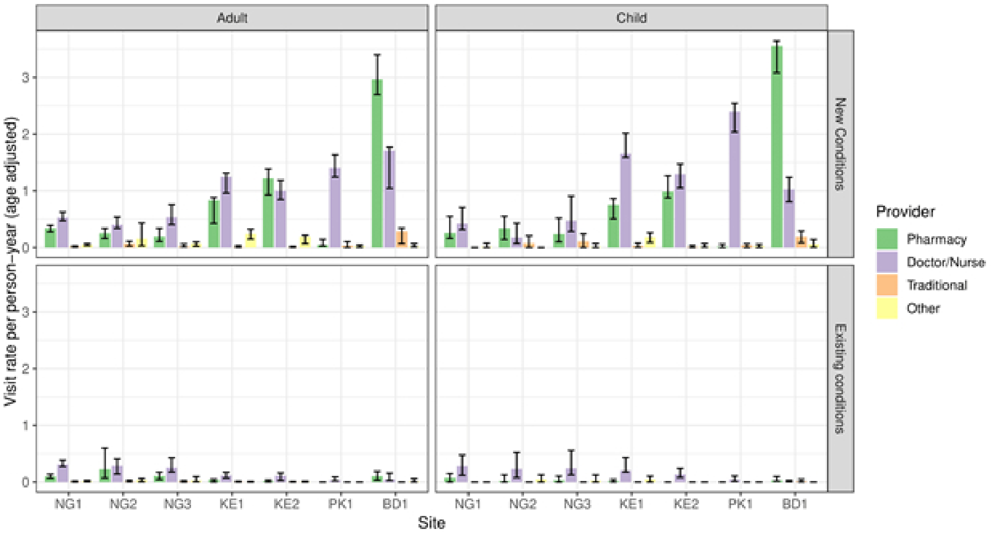

Age-adjusted (to INDEPTH population) visit rates per person-year to different outpatient care providers for adults and children (under 12) for new and existing conditions.

417x225mm (38 x 38 DPI)

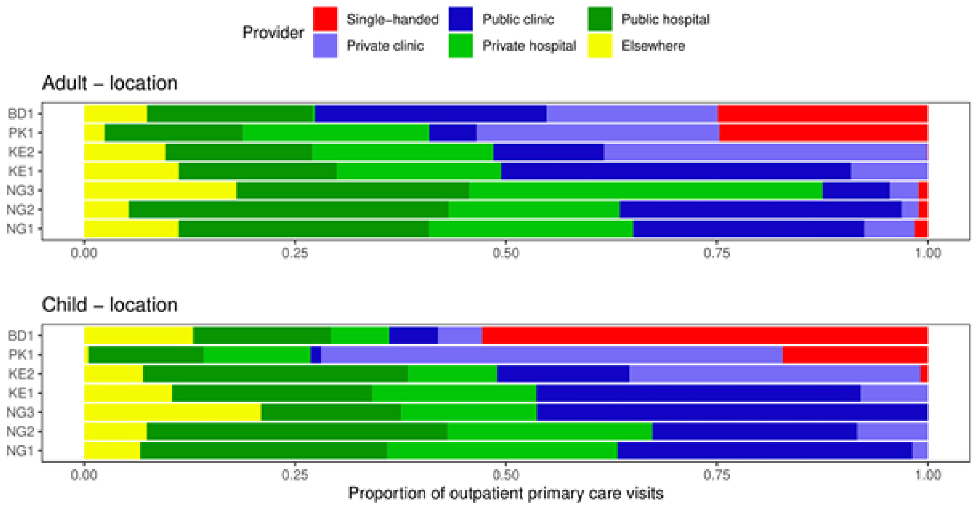

Proportion of outpatient doctor and nurse consultations for a new condition by facility type

417x214mm (38 x 38 DPI)

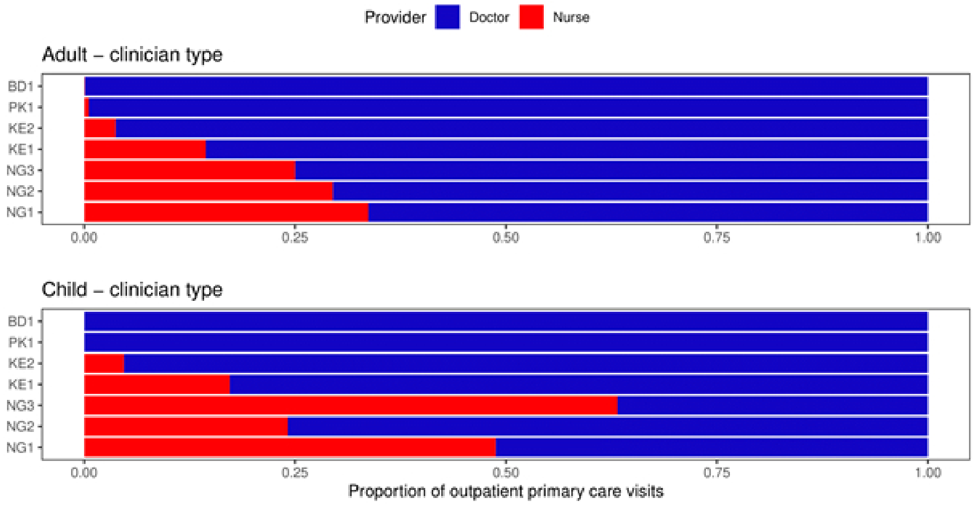

Proportion of outpatient consultations for a new condition by doctor or nurse

417x213mm (38 x 38 DPI)

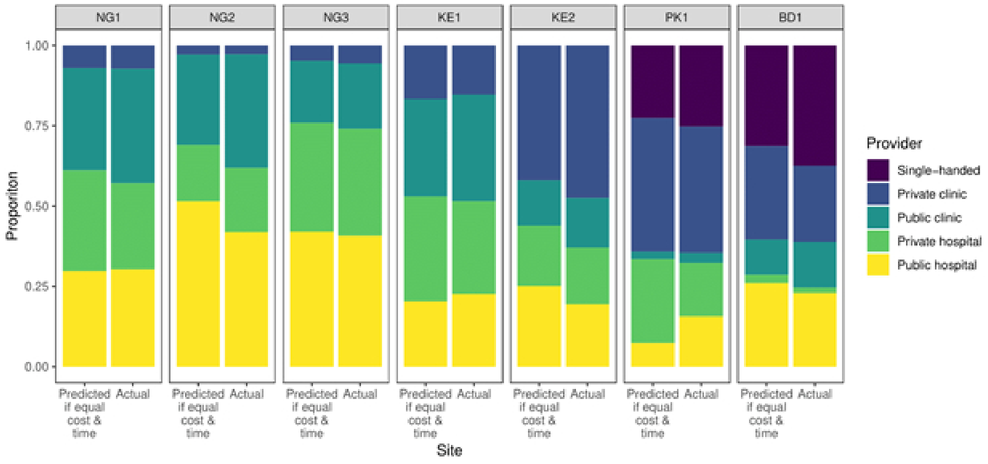

Predicted proportions of visits to different provider types for an outpatient doctor and nurse consultation if price and travel time were all equivalent versus actual proportions of visits.

417x195mm (38 x 38 DPI)

SUPPLEMENTARY INFORMATION

A Health system indicators

Table A1 reports major health system and population health indicators for the participating countries.

Table A1. Key indicators of health care systems in participating countries (Source: World Bank and WHO Global Health Observatory[1])

Key indicators	Nigeria	Kenya	Pakistan	Bangladesh
Life expectancy	54	66	67	72
GDP per capita (PPP; Int$)	5,991	3,468	5.572	4,372
Under five mortality (per 1,000 births)	120	41	69	30
Out of pocket expenditure as % of all health expenditures	75%	28%	65%	72%
Government health expenditure per capita (Int$)	27	52	40	16
Proportion with expenditures on healthcare greater than 10% of total household expenditure	15%	5%	13%	25%
Antenatal care coverage – at least four visits	49%	105%	51%	37%
ARV coverage among those with HIV	53%	68%	10%	22%
Hib (Hib3) coverage among one-year-olds	57%	92%	75%	98%
Tetanus coverage among neonates	60%	88%	85%	98%
Tuberculosis effective treatment coverage	20%	39%	64%	58%
Average of 13 International Heath Regulation core capacity scores	51	58	51	78

GDP: gross domestic product; Hib: Haemophilus influenzae type b vaccine; Int$: international dollar; PPP: purchasing power parity

For peer review only

B Supplementary information on methods

B1 Definitions of health care facilities

Table B1 Definitions of healthcare facilities used in the mapping and surveying of healthcare facilities at each site

Facility type	Definition
Clinic/centre	A facility used for the diagnosis and treatment of outpatients. Allopathic only. Subcategories include specialist clinics: vaccination; family planning; eye and vision; ear, nose, and throat; maternity and antenatal care; palliative care; and general primary or community care.
Dental clinic	A facility providing dentistry/dental care.
Hospital/secondary care facility	A health care organization that has a governing body, an organized medical staff and professional staff and inpatient facilities and provides medical nursing and related services for ill and injured patients 24 hrs per day, seven days per week
Residential or nursing care facility	Facilities that provide inpatient care specializing in nursing or long-term residential care but which are not hospital
Laboratory or sonologist clinic	A room or building equipped for testing, researching, or studying fluids, materials, or tissues from patients.
Pharmacy or medical store	A facility used by pharmacists for the compounding and dispensing of medicinal preparations and other associated professional and administrative services
Transportation services	Provider who moves a patient, specimen, or equipment from one location or another
General shop or kiosk selling healthcare products	Non-specialised vendor without healthcare professional selling health care products such as pain killers, first aid, or spectacles
Traditional practitioner	A practitioner of non-allopathic, traditional, alternative, or faith-based health services. Sub-categories include: traditional medicine, faith-based services, homeopathy, acupuncture, bone healing, and cupping.
Traditional products vendor	Vendor specialising in traditional (non-allopathic) medical products such as herbs.

For peer review only

B2 Supplementary Survey in Site PK1

An error with the electronic survey forms was identified following data collection in site PK1. The household survey form was completed successfully, including the household roster, however due to an error in the code to generate a randomly sampled adult the first recorded adult was instead sampled. This resulted in an almost exclusively male sample, which was not identified in the field at the time. After identification of this error we aimed to complete a set of individual surveys with randomly sampled women from the household rosters. However, the Covid-19 pandemic had started, so a telephone survey was deemed to be safer for the field workers. We sampled 400

For peer review only - http://bmjopen.bmj.com/site/about/guidelines.xhtml

households with at least one adult women and who had provided consent and contact details for follow-up from the original sample of households. Field workers then followed the same procedures as the main survey. Survey weights were modified for the sample from PK1 to reweight gender back to the population proportion.

B3 Statistical methods

Choice model

Choices

We define the choice set facing individual as: private clinic, public clinic, private hospital, and public hospital. In sites BD1 and PK1 we also include "private office" as a fifth option as a number of visits were recorded to these locations, whereas there were little to no visits to this type of provider in the other sites.

Choice-varying covariates

We consider two covariates that vary for each individual and choice: the price of the visit and the time to travel to the provider. The price includes consultation fees and transport cost. The price is reported in local currency (Naira, Kenyan Shillings, Pakistani Rupees, Taka). The time to provider is the travel time reported for the visit in minutes. We standardize both cost and time (dividing by the standard deviation) to ensure all variables are on the same scale to facilitate model fitting.

Individual-level covariates

From the survey data we extract the following individual level variables: age, sex, secondary education or not, if the visit was for an acute or communicable condition (excluding HIV), if the visit was for generalised or chronic pain, and if the monthly consumption expenditure was above Int$100 per person per month. For use in the models we standardize the age variable to facilitate model fitting.

Statistical Model

We take a Bayesian approach to model specification and fitting given the complex hierarchical structure of the model.

For peer review only

Each individual $i \in 1{:}N$ chooses from $k \in 1{:}K$ possible options (K is either 4 or 5). The healthcare provider options are described by a vector of choice attributes for each individual $X_{ik} = \left[C_{ik}, C_{ik}^2, T_{ik}\right]'$. This vector includes the cost, cost squared, and time. We include cost squared to allow for more flexibility between demand and price. Each individual has "part worth" preferences over the choice attributes β_i so that the utility of each choice for each individual is:

$$u_{ik} = \theta_k + X_{ik}\beta_i + u_{ik}$$

where u_{ik} is IID Gumbel distributed and θ_k are choice constant terms. Given the assumed distribution of the random error, the probability an individual chooses choice k is

$$\Pr(y_{ik} = k | X_{ik}, \beta_i, \theta_k) = \frac{\exp(\theta_k + X_{ik}\beta_i)}{\sum_{j=1}^{K} \exp\left(\theta_j + X_{ij}\beta_i\right)}$$

For more information see[2].

Hierarchical prior on model parameters

The model parameters β_i are allowed to vary by individual – they are so-called "random parameters". To model these parameters we use a hierarchical prior that "partially pools" information across individuals. We also allow these parameters to vary according to a vector of individual-level covariates W_i and further, we allow for correlation between parameters so that, for example, individuals more sensitive to price might be less sensitive to distance. In particular,

$$\beta_i \sim MVN(\beta + \Gamma W_i, \Sigma)$$

where β are mean values, Γ is a matrix that "loads" the individual covariates on to the parameters, and Σ is a covariance matrix. We can decompose the covariance matrix as $\Sigma = \mathrm{diag}(\tau)\Omega\,\mathrm{diag}(\tau)$, where τ is a scale vector and Ω is the correlation matrix of the variation across individuals. The correlation matrix can be further decomposed as $\Omega = L_\Omega L'_\Omega$ where L_Ω is the lower-triangular Cholesky decomposition. Therefore,

$$\beta_i = \beta + \Gamma W_i + \mathrm{diag}(\tau) L_\Omega z_i$$

$$z_i \sim N(0,1)$$

Missing data

We use an imputation-type procedure for the costs and times that we do not observe, i.e. for the choices not taken. The model below is part of the overall model and is estimated at the same time

For peer review only - http://bmjopen.bmj.com/site/about/guidelines.xhtml

so that the uncertainty in the imputed costs and times is carried through to the overall results. We explain the procedure for costs but use an identical model for times. Given that both variables are (often heavily) right-skewed we use log costs and time for the missing data models. We observe N values for log cost, one for each individual: $c_{ik}^{(obs)}$. We specify:

$$c_{ik}^{(obs)} = \mu_k + Z_i\gamma + u_i$$

where μ_k is a choice-specific intercept, Z_i is a vector of individual level covariates that may predict the cost of the visit (age, age squared, sex, reason for visit), γ are model parameters, and $u_i \sim N(0, \sigma_u^2)$ are IID error terms. We fit the above model using the observed data and then for the unobserved costs we sample from:

$$c_{ik}^{(mis)} \sim N(\mu_k + Z_i\gamma, \sigma_u^2)$$

so that the cost covariate is

$$C_{ik} = \begin{cases} \exp\left(c_{ik}^{(obs)}\right) & if\ y_{ik} = 1 \\ \exp\left(c_{ik}^{(mis)}\right) & if\ y_{ik} = 0 \end{cases}$$

Priors

We specify weakly informative priors on the model parameters (all N(0,1) except for the correlation matrix for which we use the lkj prior).

Estimation

We use Stan 2.19 to fit the model[3].

Elasticity of demand

To quantify the response to price or travel time we calculate the price and travel time elasticity of demand. We do this by calculating the arc elasticity:

$$\frac{(\mathrm{Pr1} - \mathrm{Pr0})/((\mathrm{Pr1} + \mathrm{Pr0})/2)}{(p_1 - p_0)/((p_1 + p_0)/2)}$$

where p_1 and p_0 are different prices or times and $\mathrm{Pr1}$ and $\mathrm{Pr0}$ are the probabilities of visiting a provider under each of the two prices or times, respectively.

For peer review only

C Additional Results

Table C1. Respondent care needs and outpatient primary care use, N(%)

Outcome	NG1	NG2	NG3	KE1	KE2	PK1	BD1
Adults							
Total	1,278 (100%)	840 (100%)	802 (100%)	1,008 (100%)	1,085 (100%)	1,112 (100%)	990 (100%)
Needed healthcare in the previous 12 months	772 (60%)	490 (58%)	418 (52%)	710 (70%)	906 (84%)	623 (56%)	959 (97%)
Received care when last needed	762 (60%)	481 (57%)	404 (50%)	674 (67%)	884 (81%)	596 (54%)	918 (93%)
Outpatient visit in last 12 months	626 (49%)	367 (44%)	309 (39%)	665 (66%)	867 (80%)	531 (48%)	900 (91%)
Outpatient visit to doctor or nurse	391 (31%)	215 (26%)	201 (25%)	405 (40%)	377 (35%)	498 (45%)	257 (26%)
Outpatient visit to doctor or nurse for new condition or routine visit	293 (23%)	132 (16%)	151 (19%)	310 (31%)	315 (29%)	291 (26%)	174 (18%)
Children (under 12)							
Total	128 (100%)	69 (100%)	79 (100%)	537 (100%)	421 (100%)	528 (100%)	658 (100%)
Needed healthcare in the previous 12 months	68 (53%)	29 (42%)	41 (52%)	482 (90%)	368 (87%)	409 (77%)	635 (97%)
Received care when last needed	68 (53%)	28 (41%)	41 (52%)	466 (87%)	363 (86%)	397 (75%)	627 (95%)
Outpatient visit in last 12 months	53 (41%)	24 (35%)	39 (49%)	462 (86%)	358 (85%)	374 (71%)	608 (92%)
Outpatient visit to doctor or nurse	36 (28%)	13 (19%)	24 (30%)	320 (60%)	205 (49%)	367 (70%)	136 (21%)
Outpatient visit to doctor or nurse for new condition	27 (21%)	11 (16%)	19 (24%)	283 (53%)	190 (45%)	329 (62%)	120 (18%)

For peer review only

Table C2. Summary statistics of study populations and national comparisons (DHS surveys – national results and urban results in parentheses where relevant)

Variable		Nigeria				Kenya			Pakistan		Bangladesh	
		NG1	NG2	NG3	DHS 2018	KE1	KE2	DHS 2014	PK1	DHS 2017/8	BD1	DHS 2014
Households												
N (completed)		1,286	845	812	-	1,018	1,089	-	988	-	1,035	-
Response rate (%)		83	69	68	99.3	69	57	99	73	96	94	99
Household size		3.7 (1.9)	3.4 (1.8)	4.2 (2.0)	4.3	3.2 (2.2)	3.4 (1.7)	3.9	5.7 (3.0)	6.6	3.8 (1.8)	4.5
Wealth quintile (%)	Bottom	0	0	0	20 (4.2)	0	0	20 (6)	0	20 (3)	0	20 (7)
	Lower	2	1	1	20 (8.1)	14	0	20 (8)	0	20 (7)	0	20 (6)
	Middle	50	59	23	20 (18.8)	59	53	20 (11)	84	20 (17)	21	20 (12)
	Upper	48	40	76	20 (30.6)	27	46	20 (26)	14	20 (31)	78	20 (26)
	Top	0	0	0	20 (38.4)	7	1	20 (49)	2	20 (42)	1	20 (49)
Monthly expenditure (Int$), median [IQR]	Total	312 [197, 487]	323 [210, 496]	502 [319, 815]	-	193 [131, 305]	187 [133, 295]	-	1,010 [672, 1,471]	--	490 [321, 770]	-
	Per person	97 [59, 162]	106 [69, 172]	144 [94, 231]	-	74 [45, 125]	96 [61, 156]	-	196 [146, 294]	-	110 [80, 162]	-
Individuals												
Age (%)	Under 5	11	9	10	15.4	13	12	14	11	13	11	10
	5 – 19	34	31	33	36.4	37	26	38	30	36	32	34
	20 – 44	36	35	39	32.0	37	51	33	41	34	45	36
	45 – 64	14	17	15	11.8	11	10	11	15	13	10	15
	65 and over	5	8	3	4.1	3	1	4	3	4	2	6
Male (%)		49	47	50	-	50	55	-	50	-	53	-
Education over 18s (%)	Completed primary	13	16	17	24.7	25	23	16	13	9	31	9
	Completed secondary	24	31	34	31.7	13	30	11	9	9	4	4
	Completed tertiary	11	6	20	12.1	5	6	7	0	12	4	10
Currently working over 18 (%)		72	76	74	75.5	65	77	81	57	96	75	98

For peer review only - http://bmjopen.bmj.com/site/about/guidelines.xhtml

Table C3. Outpatient primary care consultation rates and outpatient doctor and nurse consultation rates (visits per person-year (95% CI)).

Site	Outpatient consultation (new conditions)			Outpatient consultation (all conditions)		
	**Crude**	**WHO age adjusted**	**INDEPTH age adjusted**	**Crude**	**WHO age adjusted**	**INDEPTH age adjusted**
Adults						
NG1	0.31 (0.29, 0.33)	0.30 (0.28, 0.32)	0.30 (0.28, 0.32)	0.42 (0.40, 0.44)	0.42 (0.40, 0.44)	0.40 (0.38, 0.42)
NG2	0.25 (0.23, 0.27)	0.21 (0.19, 0.23)	0.20 (0.18, 0.22)	0.39 (0.35, 0.43)	0.32 (0.30, 0.34)	0.29 (0.27, 0.31)
NG3	0.21 (0.19, 0.23)	0.23 (0.21, 0.25)	0.22 (0.20, 0.24)	0.28 (0.26, 0.30)	0.31 (0.29, 0.33)	0.29 (0.27, 0.31)
KE1	0.77 (0.73, 0.81)	0.77 (0.73, 0.81)	0.72 (0.68, 0.76)	1.07 (1.03, 1.11)	1.17 (1.13, 1.21)	1.04 (1.00, 1.08)
KE2	0.76 (0.72, 0.80)	0.73 (0.69, 0.77)	0.70 (0.66, 0.74)	0.93 (0.89, 0.97)	1.06 (1.02, 1.10)	0.95 (0.91, 0.99)
PK1	0.60 (0.56, 0.64)	0.58 (0.54, 0.62)	0.56 (0.52, 0.60)	0.79 (0.75, 0.83)	0.85 (0.81, 0.89)	0.77 (0.73, 0.81)
BD1	1.08 (1.04, 1.12)	1.21 (1.15, 1.26)	1.16 (1.12, 1.20)	1.52 (1.46, 1.58)	1.73 (1.67, 1.79)	1.59 (1.53, 1.65)
Children (under 12)						
NG1	0.23 (0.15, 0.31)	-	-	0.29 (0.19, 0.39)	-	-
NG2	0.12 (0.04, 0.20)	-	-	0.15 (0.05, 0.25)	-	-
NG3	0.26 (0.14, 0.38)	-	-	0.34 (0.20, 0.48)	-	-
KE1	1.51 (1.41, 1.61)	-	-	1.74 (1.62, 1.86)	-	-
KE2	1.21 (1.11, 1.31)	-	-	1.30 (1.18, 1.42)	-	-
PK1	1.68 (1.56, 1.80)	-	-	1.85 (1.73, 1.97)	-	-
BD1	0.91 (0.83, 0.99)	-	-	1.04 (0.96, 1.12)	-	-
Children (under 5)						
NG1	0.24 (0.10, 0.38)	-	-	0.30 (0.14, 0.46)	-	-
NG2	0.13 (0.00, 0.27)	-	-	0.21 (0.03, 0.39)	-	-
NG3	0.38 (0.14, 0.62)	-	-	0.40 (0.16, 0.64)	-	-
KE1	2.17 (1.97, 2.37)	-	-	2.57 (2.35, 2.79)	-	-
KE2	1.62 (1.44, 1.80)	-	-	1.68 (1.50, 1.86)	-	-
PK1	2.33 (2.11, 2.55)	-	-	2.46 (2.24, 2.68)	-	-
BD1	1.35 (1.21, 1.49)	-	-	1.50 (1.36, 1.63)	-	-

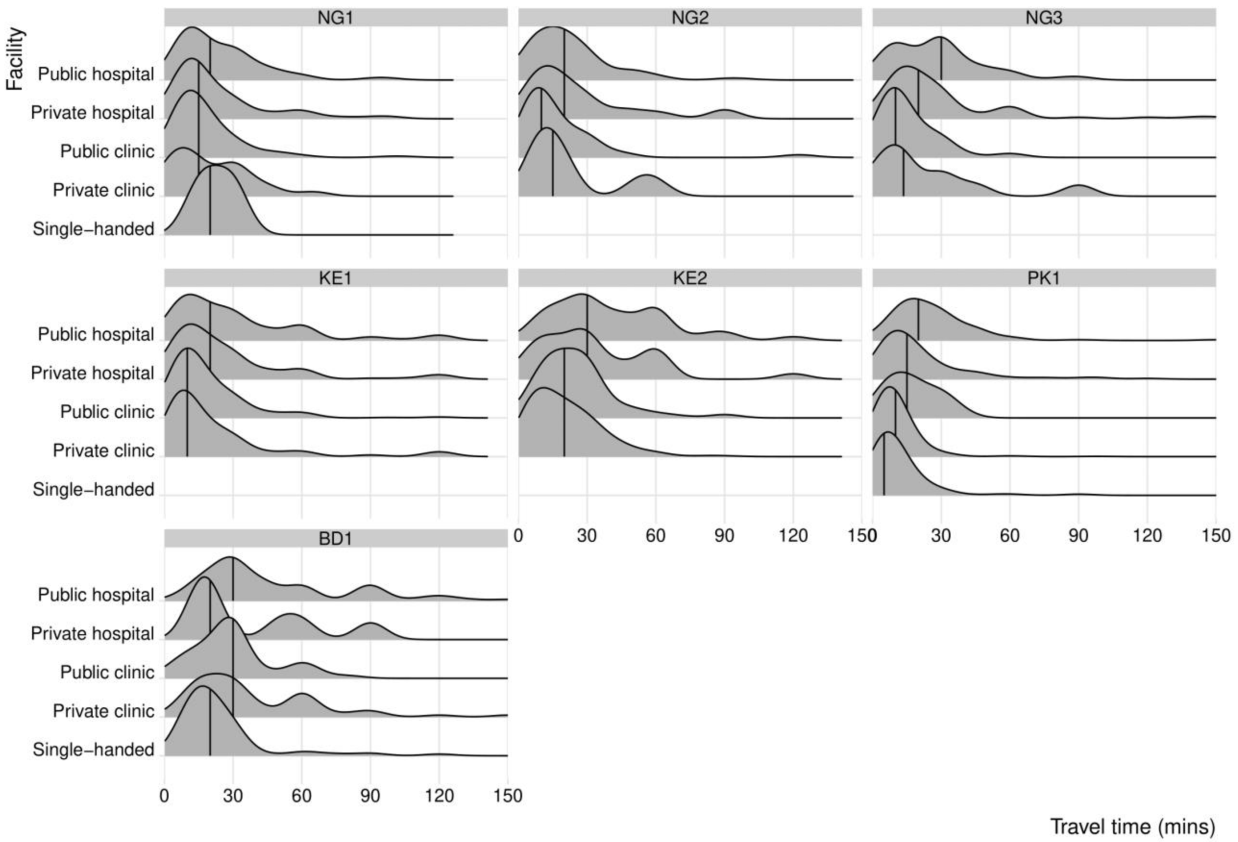

Figure C1 Distribution of reported travel times to different providers by study site with vertical lines indicating the median of the distribution.

Figure C2 Reported "primary reason" for visiting the doctor or nurse.

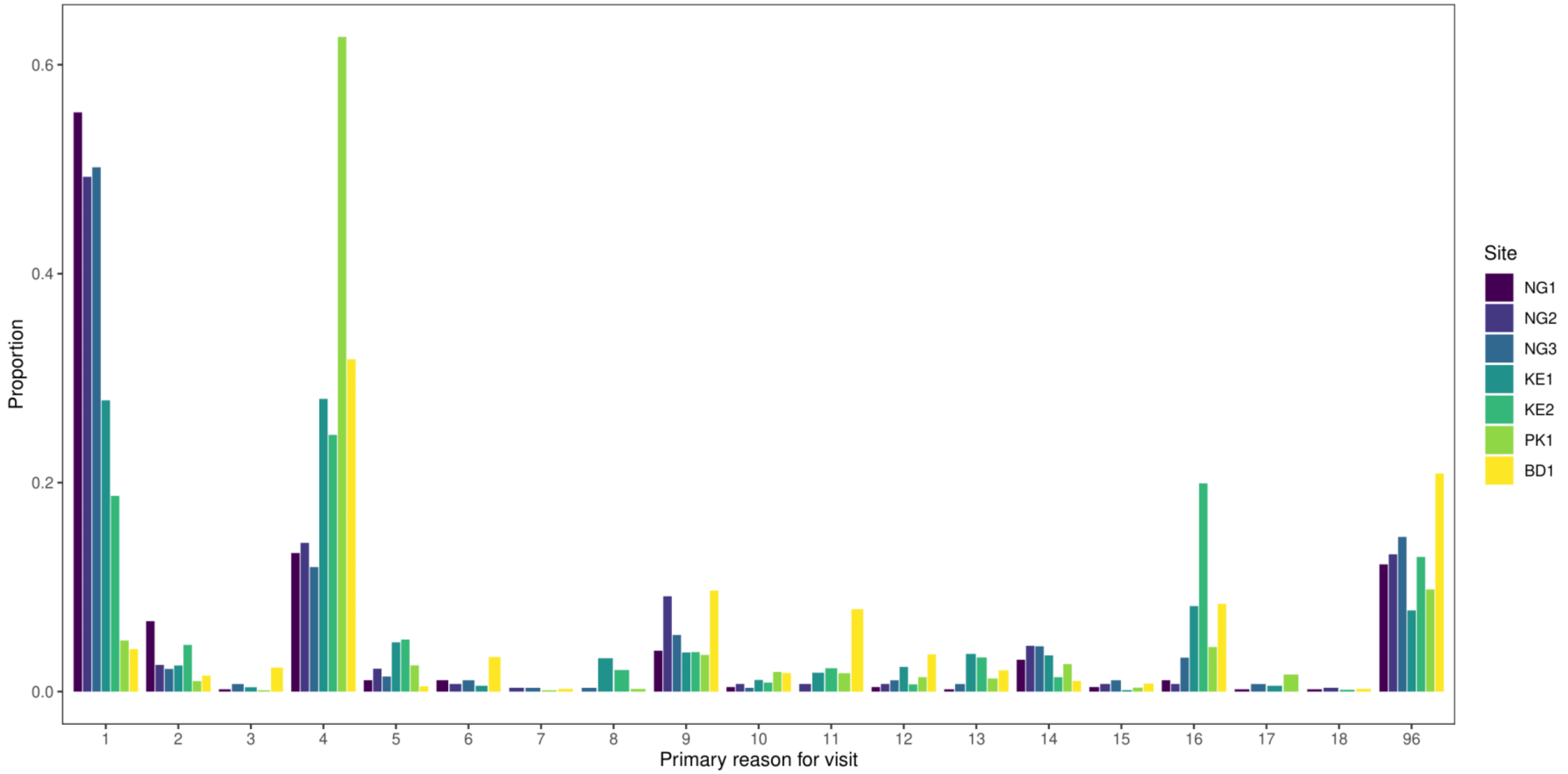

The reasons for visiting were: 1 Communicable disease (infections, malaria, tuberculosis, HIV), 2 Maternal and perinatal conditions (pregnancy), 3 Nutritional deficiencies, 4 Acute conditions (diarrhoea, fever, flu, headaches, cough, other), 5 Injury (not work related, see 8 below), 6 Surgery, 7 Sleep problems, 8 Occupation/work related condition/injury, 9 Chronic pain in your joints / arthritis (joints, back, neck), 10 Diabetes or related complications, 11 Problems with your heart, including unexplained pain in chest, 12 Problems with your mouth, teeth or swallowing, 13 Problems with your breathing, 14 High blood pressure / hypertension, 15 Stroke / sudden paralysis of one side of body, 16 Generalised pain (stomach, muscle or other non-specific pain), 17 Depression or anxiety, 18 Cancer, 96 Other (Specify)

Table C4 Percentage of respondents reporting reasons for choosing a healthcare providers by provider type

	Nigeria			Kenya		Pakistan	Bangladesh
	NG1	NG2	NG3	KE1	KE2	PK1	BD1
Nearness of the facility	63	69	57	78	72	61	54
Service providers are cordial	34	24	32	21	22	29	65
Good services available	38	32	44	31	37	37	95
Short waiting times	17	11	16	7	3	9	7
Qualified doctors are available	24	29	35	22	17	51	26
Low fees/treatment costs	28	33	43	36	24	36	39
Good waiting arrangement	12	9	15	3	2	7	5
Confidentiality is maintained	13	10	21	1	1	18	0
Don't know where else to go	7	15	7	12	6	5	3
Medicines are available	21	17	25	35	22	9	4
Diagnostics are available	11	12	11	13	4	1	11
Recommendation	11	6	11	16	8	9	20

For peer review only

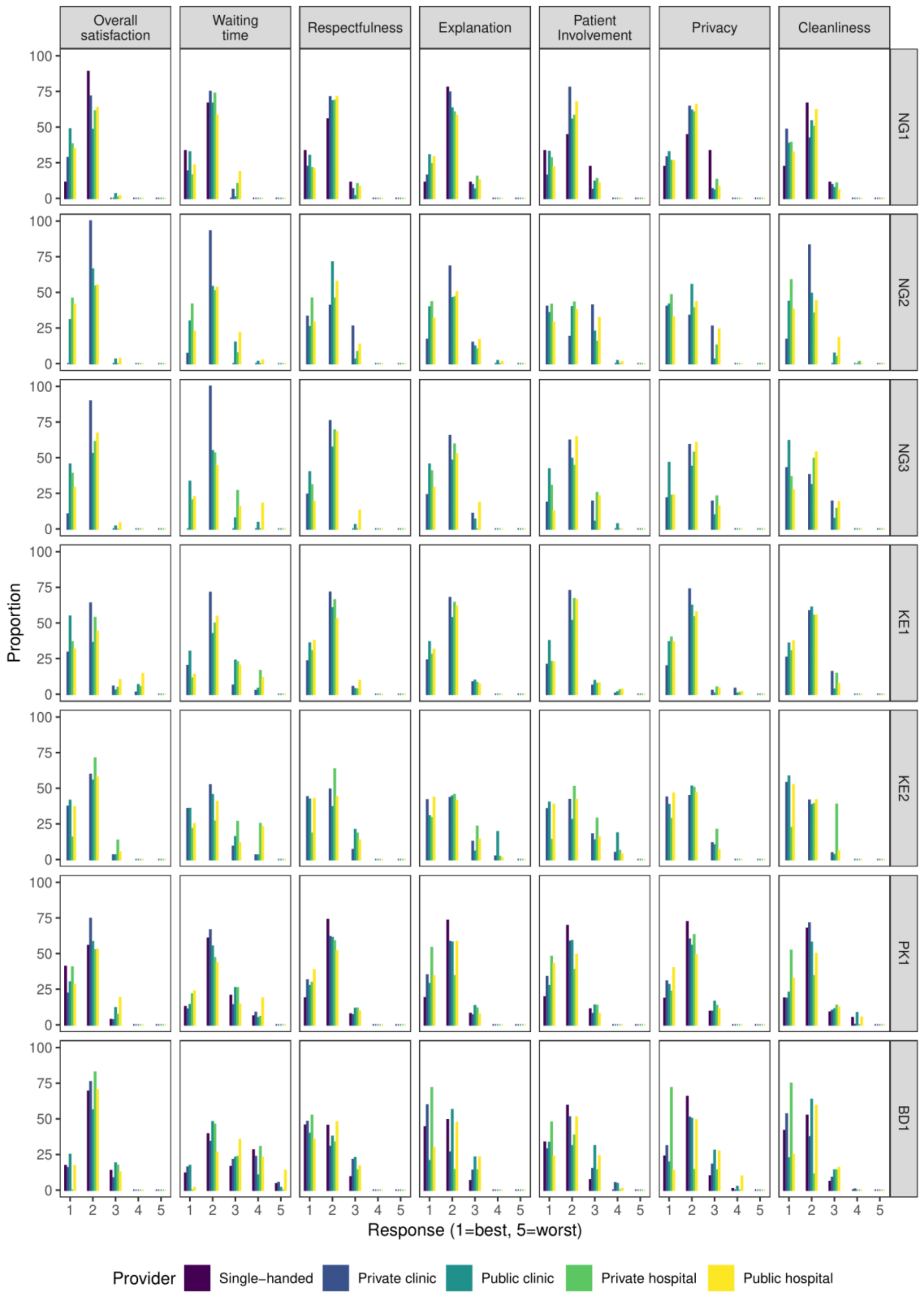

Figure C2. Satisfaction with outpatient consultations by type of facility and by site. Responses were on five-point Likert scales, either Very satisfied to very dissatisfied (Overall satisfaction) or Very good to very bad (Other questions).

REFERENCES

1 World Health Organization. Global Health Observatory. 2021.https://www.who.int/data/gho (accessed 6 Jun 2021).

2 J. Borah B. A mixed logit model of health care provider choice: analysis of NSS data for rural India. *Health Econ* 2006;**15**:915–32. doi:10.1002/hec.1166

3 Carpenter B, Gelman A, Hoffman M, *et al.* Stan: A Probabilistic Programming Language. *J Stat Softw* 2016;**VV**.

For peer review only

BMJ Open

Primary-care doctor and nurse consultations among people who live in slums: A retrospective, cross-sectional survey in four countries

Journal:	*BMJ Open*
Manuscript ID	bmjopen-2021-054142.R1
Article Type:	Original research
Date Submitted by the Author:	19-Oct-2021
Complete List of Authors:	Improving Health in Slums Collaborative, .; University of Birmingham<br>Watson, Samuel; University of Birmingham
**Primary Subject Heading**:	Health services research
Secondary Subject Heading:	Health policy
Keywords:	PRIMARY CARE, PUBLIC HEALTH, International health services < HEALTH SERVICES ADMINISTRATION & MANAGEMENT, Organisation of health services < HEALTH SERVICES ADMINISTRATION & MANAGEMENT

SCHOLARONE™
Manuscripts

I, the Submitting Author has the right to grant and does grant on behalf of all authors of the Work (as defined in the below author licence), an exclusive licence and/or a non-exclusive licence for contributions from authors who are: i) UK Crown employees; ii) where BMJ has agreed a CC-BY licence shall apply, and/or iii) in accordance with the terms applicable for US Federal Government officers or employees acting as part of their official duties; on a worldwide, perpetual, irrevocable, royalty-free basis to BMJ Publishing Group Ltd ("BMJ") its licensees and where the relevant Journal is co-owned by BMJ to the co-owners of the Journal, to publish the Work in this journal and any other BMJ products and to exploit all rights, as set out in our licence.

The Submitting Author accepts and understands that any supply made under these terms is made by BMJ to the Submitting Author unless you are acting as an employee on behalf of your employer or a postgraduate student of an affiliated institution which is paying any applicable article publishing charge ("APC") for Open Access articles. Where the Submitting Author wishes to make the Work available on an Open Access basis (and intends to pay the relevant APC), the terms of reuse of such Open Access shall be governed by a Creative Commons licence – details of these licences and which Creative Commons licence will apply to this Work are set out in our licence referred to above.

Other than as permitted in any relevant BMJ Author's Self Archiving Policies, I confirm this Work has not been accepted for publication elsewhere, is not being considered for publication elsewhere and does not duplicate material already published. I confirm all authors consent to publication of this Work and authorise the granting of this licence.

For peer review only

Primary-care doctor and nurse consultations among people who live in slums: A retrospective, cross-sectional survey in four countries

Improving Health in Slums Collaborative

Improving Health in Slums Collaborative members (in alphabetical order):

African Population and Health Research Centre (APHRC), Nairobi, Kenya
Pauline Bakibinga, Caroline Kabaria, Ziraba Kasiira, Peter Kibe, Catherine Kyobutungi, Nelson Mbaya, Blessing Mberu, Shukri Mohammed, Anne Njeri,

Aga Khan University, Karachi, Pakistan
Iqbal Azam, Romaina Iqbal, Ahsana Nazish, Narijis Rizvi,

Independent University, Bangladesh, Dhaka, Bangladesh
Syed A. K. Shifat Ahmed, Nazratun Choudhury, Ornob Alam, Afreen Zaman Khan, Omar Rahman, Rita Yusuf

Nigerian Academy of Sciences, Lagos, Nigeria
Doyin Odubanjo

University of Ibadan, Ibadan, Nigeria
Motunrayo Ayobola, Olufunke Fayehun, Akinyinka Omigbodun, Mary Osuh, Eme Owoaje, Olalekan Taiwo

University of Birmingham, Birmingham, UK
Richard J Lilford, Jo Sartori, Samuel I Watson

University of Lancaster, Lancaster, UK
Peter J Diggle

University of Warwick, Coventry, UK
Navneet Aujla, Yen-Fu Chen, Christopher Conlan, Paramjit Gill, Frances Griffiths, Bronwyn Harris, Jason Madan, Helen Muir, Oyinlola Oyebode, Vangelis Pitidis, João Porto de Albuquerque, Simon Smith, Celia Taylor, Grant Tregonning, Philip Ulbrich, Olalekan A Uthman, Ria Wilson, Godwin Yeboah, Ji-Eun Park

Corresponding author

Sam Watson

For peer review only - http://bmjopen.bmj.com/site/about/guidelines.xhtml

Institute for Applied Health Research, University of Birmingham, Birmingham, United Kingdom

s.i.watson@bham.ac.uk

Word count

3,861 words

Keywords

Healthcare access, health, LMICs, slums, welfare

Data sharing statement

The data are available from the authors on request

Author Contributions

Improving Health in Slums Collaborative drafted the manuscript and conducted the analyses. All members of the collaborative were involved in the design of the project, data collection, data analysis, and approved the final manuscript. SW submitted the paper.

Conflicts of Interest

None declared.

Patient and public involvement

Mapping of the study sites, identification of healthcare facilities, and enumeration of resident households was conducted using a participatory process involving local residents. Healthcare facility managers and owners were consulted about identification of their facilities. The public were not involved in the design of the survey questionnaires, however feedback was sought from residents in a pilot survey in all sites to assess the time burden of participating. Patient and public focus groups were established to present the findings, receive feedback, and provide contextualizing interpretation of the results.

For peer review only - http://bmjopen.bmj.com/site/about/guidelines.xhtml

For peer review only

For peer review only - http://bmjopen.bmj.com/site/about/guidelines.xhtml

ABSTRACT

Objectives

To survey on the availability and use of primary care services in slum populations..

Design

Retrospective, cross-sectional, household, individual, and healthcare provider surveys.

Setting

Seven slum sites in four countries (Nigeria, Kenya, Pakistan, and Bangladesh).

Participants

Residents of slums and informal settlements.

Primary and secondary outcome measures

Primary care consultation rates by type of provider and facility.

Results

We completed 7,692 household and 7,451 individual adult and 2,633 individual child surveys across seven sites. The majority of consultations were to doctors/nurses (in clinics or hospitals) and pharmacies rather than single handed providers or traditional healers. Consultation rates with a doctor or nurse varied from 0.2 to 1.5 visits per person-year, which was higher than visit rates to any other type of provider in all sites except Bangladesh, where pharmacies predominated. Approximately half the doctor/nurse visits were in hospital outpatient departments and most of the remainder were to clinics. Over 90% of visits across all sites were for acute symptoms rather than chronic disease. Median travel times were between 15 and 45 minutes and the median cost per visit was between 2 and 10% of a household's monthly total expenditure. Medicines comprised most of the cost. More respondents reported proximity (54-78%) and service quality

For peer review only - http://bmjopen.bmj.com/site/about/guidelines.xhtml

(31-95%) being a reason for choosing a provider than fees (23-43%). Demand was relatively inelastic with respect to both price of consultation and travel time.

Conclusions

People in slums tend to live sufficiently close to formal doctor/nurse facilities for their health seeking behavior to be influenced by preference for provider type over distance and cost. However, costs, especially for medicines are high in relation to income and use rates remain significantly below those of high-income countries.

Funding

This work was supported by National Institute for Health Research grant number 16/136/87.

Strength and limitations of this study

- This study provides the most comprehensive evidence across multiple nations to date on the facilities available to people who live in slums in general and on doctor and nurse consultations, in particular.
- We find that across the seven sites, patients most often consulted a doctor or nurse or a pharmacist/medicine seller. Moreover, around half of all doctor/nurse consultations without an overnight stay take place in a local hospital as opposed to a clinic or doctor's office and that traditional/faith healers are relatively infrequently consulted for health needs.
- The cost of outpatient care makes up more than 10% of income in all participating countries except Kenya. Drugs are the greatest cost for a visit, not consultation fees.

For peer review only - http://bmjopen.bmj.com/site/about/guidelines.xhtml

- Demand for doctor and nurse care is relatively inelastic with respect to consultation fees and travel time, so reducing travel distances in urban areas is unlikely to impact on service utilisation. Rather, service quality and patient education should be the main aim.

For peer review only

INTRODUCTION

Strengthening primary care is recognized as the most “inclusive, effective, and efficient approach” to improving population health and wellbeing and as being key to achieving Universal Health Care (UHC) globally as codified in recent international declarations.[1] However, recent high-profile analyses have shown that little systematic evidence has been collected on services that are available, how often they are used, how much they cost and what type of service patients will choose under various constraints.[2–5] It is widely believed though that people in LMIC have poor access to high quality primary care services.[2,3,6–8]

In this paper we focus on a large and vulnerable group of people; those who live in slums. A recent Lancet Series documented the many barriers that people is slums encounter in accessing services.[4,5] Following further review of the literature,[9] most evidence relates to specific programs, particularly those concerned with vaccination and childbirth (e.g.[10]). The little available evidence suggests that in many urban slums, pharmacies and drug sellers are frequently the first and only point of contact with a health system.[11,12] However, consultation with a doctor or nurse is an essential component of primary care providing diagnosis, treatment, advice and referral. Outpatient doctor and nurse consultations can be provided by public and private facilities that range from “single-handed”, sometimes informal, practitioners in solo practices to large hospital outpatient departments.

The purpose of our study is to start to fill the above gaps in the literature, examining outpatient care services in seven slum sites across four countries: Nigeria, Kenya, Pakistan, and Bangladesh. We compare visit rates to all providers that might offer primary care services including traditional/faith healers and pharmacies/medicine sellers. We then describe doctor and nurse services in more detail. Here we aim to find out use rates of different facilities that provide outpatient doctor/nurse consultations for people who live in slums, including solo providers, clinics, hospitals, and public verses private provision. We also aim to estimate how individuals

For peer review only - http://bmjopen.bmj.com/site/about/guidelines.xhtml

trade-off characteristics like cost and time when choosing between types of provider of outpatient care.

METHODS

Setting

Our aim was to examine the use of outpatient consultations with a doctor or nurse. The results reported in this article form part of a broader programme of work on health and healthcare in informal settlements described in detail elsewhere.[13]

We conducted a series of household, individual, and healthcare facility surveys across seven informal settlements in four countries: Nigeria, Kenya, Pakistan, and Bangladesh. We selected sites that (1) fulfilled the UN definition of a 'slum'[14] and (2) were named, geographically distinguishable, neighborhoods within city boundaries. This also provided a geographical basis on which to survey available health services.

Study design and population

Household and individual surveys

The design and methods of the survey components of the Improving Health in Slums project have been published elsewhere.[13] Briefly, we aimed to conduct a spatially-referenced, household-based, retrospective, cross-sectional survey in seven sites, which are described in Table 1. Key indicators for the health systems of the respective countries are reported in Supplementary Information Table 1.

All structures in each site were first mapped using satellite imagery. The resulting maps were then "ground-truthed" through participatory geospatial mapping and corrected as required. During this stage all households in each structure were identified, which formed the sampling frame for the household survey. We used an inhibitory sampling design with close pairs to generate a spatially-

For peer review only - http://bmjopen.bmj.com/site/about/guidelines.xhtml

regular and well-dispersed sample of 1,200 households for each study site,[15] assuming a response rate of 80%. Within each household, all residents were identified and an adult over 18 and a child under 12 (should one be resident) were selected at random for the individual surveys. Adult women were oversampled compared to men at a ratio of 2:1 to achieve reasonable precision in both groups as we expected greater healthcare use among women. Up to three attempts were made to complete each survey should the participant not be available at the initial attempt. For each survey instrument we sought consent from the respondent. They were provided with an oral and/or written explanation of the study and their involvement as required and then asked to provide written consent on both a paper and digital copy of the consent form. Due to an error with the sampling process, a follow-up telephone survey was conducted in PK1 to supplement the individual surveys with additional female respondents, which is described in Supplementary Information Section B2.

For peer review only

For peer review only - http://bmjopen.bmj.com/site/about/guidelines.xhtml

Table 1. Summary of study sites

Site	Location	Approx. pop. (000s)*	Approx. density (000s/km^2)	Description of population and area
NG1	Ibadan, Nigeria	5.8	5	Resettled, mixed Yoruba and Hausa community at the edge of the city including a large proportion of recent migrants from the North. Structures are mostly permanent and well-spaced. Variable access to energy with poor sanitation.
NG2	Ibadan, Nigeria	5.5	14	Mostly Yoruba population inhabiting a central, historical part of the city. Building mostly permanent but dilapidated with access to energy but little sanitation.
NG3	Lagos, Nigeria	8.1	11	Mixed Yoruba, Ilajes and other ethnic group population in low-paying or no employment. High crime area with little development and temporary structures. Little access to basic services.
KE1	Nairobi, Kenya	24.4	52	Mixed but segregated ethnicity community of generally long-term multi-generational residents. Structures are temporary with little to no access to basic services.
KE2	Nairobi, Kenya	44.9	83	Mixed community of predominantly economic migrants at the edge of an industrial area. Structures are temporary with little to no access to basic services.
PK1	Karachi, Pakistan	33.5	91	Mixed ethnicity and religion, mostly permanent, population working in blue collar jobs. Structures are permanent and multi-story with access to transient energy and sanitation services.
BD1	Dhaka, Bangladesh	60.0	171	Mostly Bengali, Muslim population working in manual services like rickshaw pulling and house work. Semi-permanent residential structures with variable access to water, sanitation, and other services.

*Estimated from data collected in this study.

The NIHR Global Health Research Unit on Improving Health in Slums was granted full ethical approval by the Ministry of Health, Lagos State Government (LSMH/2695/11/259) the Ministry of Health, Oyo State Government (ADB/479/657), Amref Health Africa (AMREF-ESRC P440/2018), the National Bioethics Committee Pakistan (4-87/NBC-298/18/RDC3530), the Bangladesh Medical Research Council and the University of Warwick Biomedical and Scientific Research Ethics Sub-Committee (REGO-2017-2043 AM01).

Procedures

Three instruments were used in the household surveys: (i) a household level survey of demographic and socioeconomic characteristics, including monthly household expenditures

For peer review only - http://bmjopen.bmj.com/site/about/guidelines.xhtml

across different categories; (ii) an individual adult survey enquiring about health care need, access, and use, and health and well-being; and (iii) a child (under 12 years of age) survey asking a caregiver about health care needs, access, and use. Questions were adapted from similar studies to facilitate comparability.[16] The adult and child surveys both asked the respondents to provide details of the last time they used healthcare (if they had done so in the previous 12 months), including facility type (public clinic, private hospital, etc.), who they saw (doctor, nurse, etc.), the reason for the visit, the cost and time taken for the visit, and questions regarding their satisfaction with the care provided. We included “doctor’s office” or “chamber” among the list of responses to facility-type, which generally refers to a solo clinician working alone in a private office – we refer to this category as “single-handed”. Survey instruments were translated using an iterative process involving forward and independent backward translations. (Survey forms are in the Supplementary Information adult.pdf, child.pdf, and household.pdf).

Outcomes and Statistical analysis

Summary statistics

From the individual survey data we identified all reported outpatient consultations to any type of provider and calculated visit rates per patient-year for each type of provider (doctor/nurse, pharmacy, traditional/faith, other) for new and existing conditions and for adults and children under 12 for comparison. We report crude rates as well as age standardized consultation rates calculated with respect to both the WHO reference population,[17] and INDEPTH population.[18] For doctor and nurse visits we further examined the proportion of visits by facility type (single-handed, public/private hospital, or public/private clinic) and reported provider type (doctor or nurse).

We estimated key characteristics of these visits including the median [Interquartile range (IQR)] time required for the visit, median [IQR] cost of the visit, proportion of households spending more

For peer review only - http://bmjopen.bmj.com/site/about/guidelines.xhtml

than 10% of their monthly expenditure on a visit (as used by the WHO's Global Health Observatory), reason for choosing the provider, and satisfaction by site and facility type.

Choice model

To estimate the role of price and travel time in the choice over a provider for a doctor or nurse for an outpatient consultation, we estimated a 'choice model'. We specified a random parameters logit model,[19] which we describe in detail in the Supplementary Information. In the model, each option from a set of choices has an observed component (price and time), the utility of which is determined by observed (e.g. age, sex) and unobserved (e.g. taste, quality) characteristics. There are several examples of choice modelling for healthcare providers in LMICs (for example, Borah (2006)[20]). The "choice set" we examined was: private clinic, public clinic, private hospital, or public hospital. We also included "single-handed" doctor's office for Bangladesh and Pakistan, since this type of consultation was rare or non-existent in our African sites.

We studied each site separately. There were four estimates of interest from each model: the predicted proportion of visits to each provider type "holding fixed" price and travel time, the price elasticity of demand, the travel time elasticity of demand, and the average change in price (willingness to pay, WTP) to make an individual indifferent between two options, one of which is 15 minutes further travel away than the other. The elasticities and WTP were calculated separately for households whose monthly consumption expenditure was above and below Int$ 100 per person per month. An elasticity is interpreted as the percentage change in demand you would expect for a 1% increase in price or travel time. We allowed preferences to vary by age, sex, secondary education, seeking care for an acute or communicable condition, or for chronic or generalized pain, and monthly consumption expenditure. The "price" of a visit included the consultation fee plus the travel cost, which would be known up front to the individual, and not drugs or tests, which would not be known in advance. The prices and times of travel for providers not visited were imputed based on the above listed individual covariates.

For peer review only - http://bmjopen.bmj.com/site/about/guidelines.xhtml

Patient and public involvement

Mapping of the study sites, identification of healthcare facilities, and enumeration of resident households was conducted using a participatory process involving local residents. Healthcare facility managers and owners were consulted about identification of their facilities. The public were not involved in the design of the survey questionnaires, however feedback was sought from residents in a pilot survey in all sites to assess the time burden of participating. Patient and public focus groups were established to present the findings, receive feedback, and provide contextualizing interpretation of the results.

For peer review only

For peer review only - http://bmjopen.bmj.com/site/about/guidelines.xhtml

RESULTS

Household and Individual Sample

Overall, 7,692 households participated in the surveys with 7,451 individual adults and 2,633 individual child surveys completed. The median response rate was 69%, varying by site from 57% in Site KE2 to 94% in Site BD1. Table 2 reports demographic and socioeconomic statistics of the population-weighted sample of individual respondents by site, and for those reporting an outpatient consultation (population summaries are reported in Table C2, Supplementary Information).

Outpatient consultation rates across providers of all types

Figure 1 shows the visit rates per patient-year to different types of provider. Between 29% (BD1) and 61% (KE1) of visits were to doctors and nurses, while the majority of other visits were to a pharmacy. In only two sites (BD1 and KE2) were pharmacies visited more frequently than doctors or nurses. The proportion of outpatient consultations that were for new conditions ranged from 61% (NG2) to 84% (KE2). Proportionately very few healthcare visits were made to traditional or faith healers.

Outpatient consultation rates to a doctor or nurse

Table 3 reports the outpatient consultation rates by study site for both adults and children (under 12 and under 5) – equivalent rates for new conditions only are reported in Table C3 Supplementary Information. Nigerian sites had consistently the lowest outpatient consultation rates, which were comparable for adults and children: approximately 0.2 to 0.4 outpatient visits to a doctor or nurse for a new condition per patient-year. Rates were higher in other sites, ranging, for adults, from approximately 0.8 (PK1) to 1.5 (BD1) visits per patient-year. Apart from Nigeria, outpatient consultation rates were higher for children than adults.

For peer review only - http://bmjopen.bmj.com/site/about/guidelines.xhtml

TABLE 2 Summary statistics of the overall study sample (All) and those reporting a doctor or nurse (D/N) consultation for a new condition visit by site. Values are mean (sd) unless otherwise stated.

Variable		Nigeria						Kenya				Pakistan		Bangladesh	
		NG1		NG2		NG3		KE1		KE2		PK1		BD1	
		All	D/N	All	D/N	All	D/N	All	D/N	All	D/N	All	D/N	All	D/N
Adults															
N		1,278	391	840	215	802	201	1,008	405	1,085	377	1,112	498	990	257
Household size		3.6 (1.8)	3.8 (1.8)	4.7 (1.4)	4.9 (1.3)	4.6 (1.8)	4.7 (1.8)	3.5 (2.1)	3.1 (2.0)	2.5 (1.4)	2.4 (1.3)	5.5 (2.6)	5.5 (2.5)	4.3 (1.9)	4.4 (2.0)
Wealth quintile (%)	Bottom	0	0	0	0	0	0	0	0	0	0	0	0	0	0
	Lower	2	3	3	0	1	1	15	13	0	0	0	0	0	0
	Middle	49	48	51	47	24	24	59	61	55	54	83	83	21	19
	Upper	48	49	46	53	75	75	25	26	44	45	15	15	78	77
	Top	0	0	0	0	0	0	0	0	1	1	3	2	1	3
Monthly household expenditure (Int$)	Total	300 (248)	362 (250)	342 (274)	349 (268)	560 (364)	580 (363)	232 (136)	213 (127)	243 (127)	274 (152)	1,122 (631)	1,179 (667)	463 (234)	505 (257)
	Per person	99 (86)	108 (79)	95 (78)	101 (87)	136 (101)	142 (104)	84 (62)	87 (63)	118 (81)	130 (92)	223 (125)	230 (138)	116 (58)	126 (61)
Age		39.0 (16.7)	39.9 (17.4)	47.8 (16.6)	51.3 (17.7)	42.0 (15.0)	43.8 (15.2)	37.6 (13.6)	40.1 (14.7)	34.3 (11.1)	34.4 (11.0)	37.6 (12.6)	39.2 (13.1)	34.6 (12.3)	36.6 (12.7)
Sex (% male)		45	35	49	50	47	45	46	34	46	42	50	42	53	41
Education (%)	Primary/middle	22	19	34	36	16	19	60	55	39	38	7	10	61	58
	Secondary	46	52	55	52	54	49	34	38	51	49	79	76	31	30
	Tertiary	24	22	9	9	32	31	6	7	8	12	14	14	8	12
Children (under 12)															
N		128	36	69	13	79	24	537	320	421	205	528	367	658	136
Household size		4.9 (1.5)	4.9 (1.4)	4.7 (1.4)	4.9 (1.3)	4.9 (1.4)	5.1 (1.4)	5.2 (1.8)	5.2 (1.9)	4.4 (1.4)	4.3 (1.4)	7.5 (3.0)	7.4 (2.9)	4.9 (1.6)	5.4 (1.8)
Wealth quintile (%)	Lowest	0	0	0	0	0	0	0	0	0	0	0	0	0	0
	Lower	2	1	3	0	1	0	13	13	0	0	0	0	0	0
	Middle	48	62	51	47	25	37	60	56	55	55	84	85	25	20
	Upper	50	37	46	53	74	63	27	30	44	45	15	14	74	78
	Top	0	0	0	0	0	0	0	1	0	0	1	1	1	2
Monthly household expenditure (Int$)	Total	365 (315)	359 (227)	437 (310)	417 (254)	579 (336)	626 (386)	284 (29)	274 (152)	276 (132)	286 (143)	1,304 (733)	1,324 (740)	472 (235)	525 (252)
	Per person	74 (58)	75 (49)	93 (60)	92 (59)	123 (68)	130 (75)	56 (29)	130 (92)	69 (37)	70 (36)	184 (112)	194 (117)	100 (46)	104 (50)
Age		6.9 (3.6)	6.0 (3.4)	6.7 (3.7)	4.9 (3.3)	6.5 (3.9)	6.2 (3.8)	5.9 (3.7)	5.5 (3.8)	5.6 (3.6)	5.1 (3.7)	6.0 (3.7)	5.4 (3.4)	5.5 (3.7)	4.7 (3.7)
Sex (% male)		44	33	57	51	43	40	55	52	48	46	53	53	52	62

For peer review only - http://bmjopen.bmj.com/site/about/guidelines.xhtml

Doctor and nurse consultation rates by provider type

Figure 2 shows the proportion of outpatient consultations for a new condition by facility type. There were differences between the sites and countries. Single-handed facilities accounted for approximately 25% of adult visits in Bangladesh and Pakistan, and 50% and 20% of child visits in these countries, respectively. However, almost no visits to single-handed facilities were recorded in Nigeria and Kenya. Hospital outpatient departments and clinics accounted for comparable shares of outpatient consultations; in particular, for adults, hospital visit shares were 51% (NG1), 66% (NG2), 69% (NG3), 38% (KE1), 39% (KE2), 37% (PK1), and 21% (BD1). These figures were similar for children. Figure 3 shows the proportion of visits by provider (doctor or nurse). For the Pakistani and Bangladeshi communities, almost all outpatient consultations were with a doctor, whereas in Kenya and Nigeria a significant minority of consultations were with a nurse for both adults and children.

For peer review only

Table 3. Outpatient primary care consultation rates and outpatient doctor and nurse consultation rates (visits per person-year (95% CI)).

Site	Outpatient consultation (all conditions)		
	**Crude**	**WHO age adjusted**	**INDEPTH age adjusted**
	Adults		
NG1	0.42 (0.40, 0.44)	0.42 (0.40, 0.44)	0.40 (0.38, 0.42)
NG2	0.39 (0.35, 0.43)	0.32 (0.30, 0.34)	0.29 (0.27, 0.31)
NG3	0.28 (0.26, 0.30)	0.31 (0.29, 0.33)	0.29 (0.27, 0.31)
KE1	1.07 (1.03, 1.11)	1.17 (1.13, 1.21)	1.04 (1.00, 1.08)
KE2	0.93 (0.89, 0.97)	1.06 (1.02, 1.10)	0.95 (0.91, 0.99)
PK1	0.79 (0.75, 0.83)	0.85 (0.81, 0.89)	0.77 (0.73, 0.81)
BD1	1.52 (1.46, 1.58)	1.73 (1.67, 1.79)	1.59 (1.53, 1.65)
	Children (under 12)		
NG1	0.29 (0.19, 0.39)	-	-
NG2	0.15 (0.05, 0.25)	-	-
NG3	0.34 (0.20, 0.48)	-	-
KE1	1.74 (1.62, 1.86)	-	-
KE2	1.30 (1.18, 1.42)	-	-
PK1	1.85 (1.73, 1.97)	-	-
BD1	1.04 (0.96, 1.12)	-	-
	Children (under 5)		
NG1	0.30 (0.14, 0.46)	-	-
NG2	0.21 (0.03, 0.39)	-	-
NG3	0.40 (0.16, 0.64)	-	-
KE1	2.57 (2.35, 2.79)	-	-
KE2	1.68 (1.50, 1.86)	-	-
PK1	2.46 (2.24, 2.68)	-	-
BD1	1.50 (1.36, 1.63)	-	-

For peer review only

Choice of providers of doctor and nurse consultations

Table 4 reports travel times, waiting times, and travel, drug, tests and other costs for the different types of facility for doctor and nurse consultations. Within each site the travel time to reach each type of facility was broadly similar with median travel times generally ranging from 15 to 30 minutes for all types of facility (see also Figure C1, Supplementary Information).

Bangladesh and Nigeria were the most expensive locations to seek treatment, both in relative and absolute terms, with median spending ranging from Int$ 21 to 82 depending on facility type. Median spending in Kenyan facilities ranged from Int$ 6 to 15, and in Pakistan Int$ 16 to 42. Medication costs accounted the bulk of the cost of an outpatient consultation in all sites: the median proportion of the total cost of a visit accounted for by drugs was 67% to 100%. Consultation fees in the Nigerian and Kenyan sites were generally under Int$5 and often zero, whereas in Pakistan and Bangladesh they were higher (approximately Int$5 to 15). The median expenditure for a visit was less than 10% of total monthly household expenditure for almost all types of consultation across all seven sites. However, the costs were highly skewed so that in all countries except Kenya over a third of consultations would constitute more than a third of a person's total monthly household expenditure.

Table 5 reports the results from the choice model. All elasticities were below zero, showing people prefer less costly and nearer services. However, the mean estimated elasticities were almost all between zero and minus one, with the exception of in Kenya, suggesting demand was relatively inelastic with respect to both price and time. Figure 4 compares predicted share of visits to different providers, net of costs and travel times, to actual proportions of visits: there was little qualitative difference between the two. This expressed choice is similar to survey responses; only 23-43% of respondents reported "low fees" being a reason they chose a provider (Table C4, Supplementary Information). Demand was generally more elastic in poorer households (Table 5).

For peer review only - http://bmjopen.bmj.com/site/about/guidelines.xhtml

Table 4. Summary of time and costs of outpatient doctor and nurse consultations by study site for adults and children combined. All values are median [IQR] if the number of recorded visits N was five or more.

Variable		Facility type				
		Single-handed	Clinics		Hospitals	
			Private	Public/NGO	Private	Public/NGO
NG1						
Travel time (mins)		-	15 [5, 30]	15 [10, 20]	15 [10, 30]	15 [10, 30]
Waiting time (mins)		-	5 [2, 10]	10 [5, 40]	5 [2, 10]	30 [10, 60]
Costs (Int$)	Consultation	-	0 [0, 3]	0 [0, 2]	0 [0, 8]	0 [0, 3]
	Drugs	-	17 [4, 73]	15 [8, 23]	21 [10, 42]	17 [10, 25]
	Tests	-	0 [0, 7]	1 [0, 4]	2 [0, 8]	2 [0, 8]
	Travel	-	0 [0, 4]	0 [0, 5]	2 [0, 8]	2 [0, 10]
	Total	-	42 [5, 88]	21 [13, 33]	42 [21, 61]	23 [14, 42]
Total cost as % of monthly h/h expenditure		-	9 [2, 21]	7 [3, 15]	11 [6, 19]	9 [4, 16]
Households spending >10% of monthly expenditure on visit (%)		-	43	32	56	43
NG2						
Travel time (mins)		-	20 [12, 44]	10 [5, 20]	15 [10, 30]	20 [10, 30]
Waiting time (mins)		-	30 [12, 55]	15 [5, 30]	10 [5, 30]	30 [13, 60]
Costs (Int$)	Consultation	-	0 [0, 1]	0 [0, 1]	0 [0, 0]	0 [0, 4]
	Drugs	-	5 [1, 11]	15 [8, 29]	20 [8, 42]	17 [11, 29]
	Tests	-	0 [0, 0]	0 [0, 4]	0 [0, 8]	0 [0, 8]
	Travel	-	0 [0, 0]	0 [0, 4]	0 [0, 8]	0 [0, 8]
	Total	-	13 [9, 32]	20 [13, 34]	29 [13, 78]	29 [17, 42]
Total cost as % of monthly h/h expenditure		-	11 [6, 46]	5 [3, 13]	11 [4, 23]	13 [5, 30]
Households spending >10% of monthly expenditure on visit (%)		-	50	32	56	55
NG3						
Travel time (mins)		-	22 [10, 41]	10 [9, 20]	20 [14, 30]	30 [10, 40]
Waiting time (mins)		-	10 [5, 12]	20 [5, 50]	10 [5, 30]	46 [10, 120]
Costs (Int$)	Consultation	-	0 [0, 0]	1 [0, 2]	0 [0, 8]	0 [0, 4]
	Drugs	-	25 [12, 42]	13 [8, 22]	42 [21, 49]	17 [8, 30]
	Tests	-	0 [0, 19]	0 [0, 4]	0 [0, 13]	7 [0, 17]
	Travel	-	0 [0, 0]	0 [0, 2]	2 [0, 3]	2 [0, 4]
	Total	-	56 [29, 81]	20 [11, 34]	47 [33, 81]	34 [17, 60]
Total cost as % of monthly h/h expenditure		-	10 [3, 16]	4 [2, 9]	9 [4, 15]	7 [3, 12]
Households spending >10% of monthly expenditure on visit (%)		-	50	17	43	33
KE1						
Travel time (mins)		-	10 [5, 20]	15 [10, 30]	20 [10, 30]	28 [10, 45]
Waiting time (mins)		-	30 [5, 42]	60 [18, 94]	20 [5, 42]	30 [20, 120]
Costs (Int$)	Consultation	-	0 [0, 2]	0 [0, 0]	0 [0, 2]	0 [0, 1]
	Drugs	-	9 [2, 13]	5 [2, 9]	7 [2, 18]	6 [0, 9]
	Tests	-	2 [0, 6]	0 [0, 3]	0 [0, 6]	0 [0, 6]
	Travel	-	2 [0, 5]	0 [0, 2]	2 [0, 3]	1 [0, 4]
	Total	-	13 [8, 30]	6 [2, 12]	11 [4, 30]	8 [2, 24]
Total cost as % of monthly h/h expenditure		-	5 [1, 21]	3 [1, 11]	5 [1, 11]	3 [1, 11]
Households spending >10% of monthly expenditure on visit (%)		-	43	27	29	26
KE2						
Travel time (mins)		-	18 [10, 30]	20 [15, 30]	30 [15, 50]	35 [30, 58]
Waiting time (mins)		-	10 [5, 20]	30 [10, 60]	15 [5, 30]	40 [20, 88]
Costs (Int$)	Consultation	-	0 [0, 1]	0 [0, 1]	0 [0, 3]	1 [0, 3]

For peer review only - http://bmjopen.bmj.com/site/about/guidelines.xhtml

	Drugs	-	8 [4, 14]	6 [2, 11]	9 [5, 19]	11 [7, 28]
	Tests	-	3 [0, 6]	0 [0, 2]	9 [0, 19]	6 [2, 11]
	Travel	-	1 [0, 2]	1 [0, 1]	2 [1, 4]	2 [2, 4]
	Total	-	9 [4, 19]	6 [4, 13]	13 [6, 31]	15 [4, 48]
Total cost as % of monthly h/h expenditure		-	3 [1, 9]	3 [1, 8]	4 [1, 14]	5 [1, 15]
Households spending >10% of monthly expenditure on visit (%)		-	19	21	29	31
PK1						
Travel time (mins)		10 [5, 15]	5 [5, 10]	20 [14, 30]	15 [10, 30]	30 [15, 30]
Waiting time (mins)		10 [0, 25]	10 [10, 20]	52 [12, 98]	25 [10, 60]	30 [14, 60]
Costs (Int$)	Consultation	3 [3, 5]	3 [3, 6]	0 [0, 1]	6 [1, 29]	0 [0, 0]
	Drugs	12 [6, 16]	9 [6, 19]	12 [4, 16]	16 [5, 44]	16 [0, 31]
	Tests	0 [0, 0]	0 [0, 0]	0 [0, 0]	0 [0, 0]	0 [0, 0]
	Travel	0 [0, 0]	0 [0, 0]	1 [0, 2]	2 [0, 6]	2 [0, 6]
	Total	18 [11, 25]	16 [9, 24]	17 [11, 25]	42 [18, 104]	19 [7, 55]
Total cost as % of monthly h/h expenditure		1 [1, 4]	2 [1, 3]	1 [0, 3]	2 [4, 9]	2 [0, 4]
Households spending >10% of monthly expenditure on visit (%)		6	9	8	25	12
BD1						
Travel time (mins)		20 [15, 30]	30 [20, 60]	30 [20, 30]	35 [19, 60]	30 [24, 60]
Waiting time (mins)		45 [30, 90]	60 [30, 90]	85 [30, 128]	20 [19, 30]	60 [30, 120]
Costs (Int$)	Consultation	12 [6, 15]	12 [1, 15]	0 [0, 0]	0 [0, 4]	0 [1, 4]
	Drugs	26 [15, 47]	32 [14, 61]	15 [5, 24]	27 [20, 60]	21 [9, 41]
	Tests	21 [8, 46]	31 [0, 61]	11 [1, 20]	6 [6, 6]	31 [9, 48]
	Travel	2 [2, 3]	2 [1, 3]	3 [2, 5]	2 [2, 2]	3 [2, 6]
	Total	52 [30, 89]	82 [26, 128]	22 [15, 39]	36 [32, 62]	45 [18, 96]
Total cost as % of monthly h/h expenditure		13 [6, 23]	17 [5, 32]	6 [2, 10]	11 [10, 16]	9 [3, 17]
Households spending >10% of monthly expenditure on visit (%)		58	67	28	75	44

The most frequently cited reasons for choosing a particular healthcare facility and provider were proximity (54-78% of respondents), service quality (31-95%) and cordiality (21-65%) (Table C4 Supplementary Information). Generally, the majority of respondents were either “Very satisfied” or “satisfied” with their visit and rated most aspects of the visit either “Very good” or “good”, aside from waiting time where negative responses were more common. Respondents were more likely to be “Very satisfied” with care at public clinics in four of the seven sites, with comparable satisfaction with other providers in the remaining sites. Respondents were also least likely to report “very satisfied” for single-handed facilities and private clinics (see Figure C2, Supplementary Information). The most common medical reason for seeking care was “communicable” or “acute conditions” (35-69%) with only 2 to 6% of respondents reporting a chronic condition as the reason for the visit (Figure C5, Supplementary Information).

For peer review only - http://bmjopen.bmj.com/site/about/guidelines.xhtml

Table 5. Price and travel time elasticities of demand and willingness to pay for nearer services for households by total monthly per person household consumption expenditure, values are posterior mean (95% credible intervals)

	**Price elasticity of demand**		**Time elasticity of demand**		**Willingness to pay for 15 minutes less travel time (Int$)**	
	<Int$100 pppm	***≥Int$100 pppm***	***<Int$100 pppm***	***≥Int$100 pppm***	***<Int$100 pppm***	***≥Int$100 pppm***
NG1	-0.62 (-0.89, -0.36)	-0.27 (-0.46, -0.10)	-1.01 (-1.76, -0.26)	-0.47 (-1.04, -0.18)	8.45 (-0.95, 22.69)	7.20 (1.94, 14.51)
NG2	-0.96 (-1.43, -0.49)	-0.47 (-0.75, -0.17)	-0.29 (-0.96, 0.42)	-0.36 (-0.88, -0.21)	1.35 (-2.07, 5.03)	3.00 (-2.06, 9.49)
NG3	-0.45 (-1.23, 0.23)	-0.03 (-0.53, 0.43)	0.00 (-0.84, 1.02)	0.00 (-0.61, 0.78)	-2.39 (-39.37, 41.45)	-2.87 (-27.97, 33.16)
KE1	-1.92 (-2.46, -1.26)	-0.62 (-1.18, -0.06)	-0.34 (-0.94, 0.22)	-0.16 (-0.63, 0.31)	1.15 (-0.39, 3.25)	1.50 (-1.23, 5.44)
KE2	-1.94 (-2.44, -1.26)	-1.49 (-1.96, -0.89)	-0.27 (-0.87, 0.42)	-0.27 (-0.81, 0.40)	1.13 (-1.08, 3.60)	1.03 (-0.86, 3.10)
PK1	-0.30 (-1.72, 1.05)	-0.69 (-1.31, -0.04)	-1.52 (-2.70, -0.32)	-0.98 (-1.61, -0.30)	6.93 (2.03, 16.04)	6.19 (2.13, 12.65)
BD1	-0.79 (-2.38, 0.72)	-0.28 (-1.63, 0.76)	-1.09 (-1.88, -0.36)	-0.21 (-0.59, 0.17)	12.20 (-46.93, 72.03)	7.03 (-18.30, 34.48)

For peer review only - http://bmjopen.bmj.com/site/about/guidelines.xhtml

DISCUSSION

We found that people living in slums make use of a range of primary care providers, both public and private, and from individual clinicians to hospital outpatient departments. While there are considerable differences between slums and countries, we found across all sites that: traditional healers were seldom consulted for health needs, reflecting previous evidence;[21] doctors and nurses were frequently consulted, more so than pharmacies in all sites other than Bangladesh; formal clinics/hospital outpatient departments were more popular than "single-handed" settings; the costs of medication exceeded those of consultations; and, demand was relatively inelastic with respect to consultancy and travel cost.

The various providers of doctor and nurse consultations were all in relative proximity to the slum precinct; the majority of respondents reported requiring less than 30 minutes to reach their provider of choice. It is noteworthy that relatively frequent use was made of hospital outpatient departments, which accounted for as much as 50% of doctor or nurse outpatient consultations in some sites. We also found low use of individually owned and operated practices. This is in contrast to what we expected, perhaps because of the substantial literature on quality of care in "single-handed" practices, much of it emanating from India.[3,6,7,22]

There was evidence that many households spent a significant proportion of their money on healthcare. While we found evidence that the up-front cost of a visit, including consultancy fees and travel costs, were relatively low and had only a small effect on demand for services, the total cost of a visit was much higher and often differed little between public and private facilities. Indeed, the consultancy fees even at private providers were often zero. The WHO's Global Health Observatory use the proportion of households spending more than 10% of their expenditure on healthcare as a marker of national health system performance and report figures of 15%, 5%, 13%, and 25% for Nigeria, Kenya, Pakistan, and Bangladesh, respectively (Table A1, Supplementary). The relative differences between countries reflect those found in this study. In

For peer review only - http://bmjopen.bmj.com/site/about/guidelines.xhtml

both data sources, Kenya has the lowest rates of high spending relative to household spending which may be attributed to the National Hospital Insurance Fund. Nevertheless, our results suggest the proportion of slum households meeting the 10% threshold is much higher (generally more than 50%) than the above national averages.

Most of the cost of a primary care visit was accounted for by the cost of drugs, which reflects findings from another LMIC-based study that showed high mark ups on medication.[23] Kenya is the only country in our study with any public funding for medication, and while respondents there did report the lowest cost to access care of any of our sites, drugs were still the biggest source of expenditure.[24] Much of the literature on healthcare access in LMICs has focused on the deleterious effect of user and consultation fees, and it has been shown that even very small costs can suppress demand and do so non-selectively and to the detriment of children (e.g.[25]). However, our evidence suggests that the costs of treatment may be a larger problem for access to care in urban areas. Few visits resulting in spending on medical tests.

Patient choice

The finding that people will frequently attend a formal facility such as a hospital outpatient department, bypassing other facilities/providers on the way, shows that people are likely willing to trade convenience and cost for perceptions of quality. We have quantified and explored these trade-offs formally by use of a 'choice model'. An immediate limitation is that we are not 'comparing like for like' since we must assume that demand for a provider type is heavily determined by the type of symptom a person is experiencing. That said, it must also be assumed that when the situation is perceived to be more serious, the more a person will eschew local in favor of more distant providers. In that sense our findings are all the more impressive, representing an underestimate of preference for formal providers when the complaint is more serious. Nevertheless, higher elasticities among the poorest people is a cause for concern.

For peer review only - http://bmjopen.bmj.com/site/about/guidelines.xhtml

Overall demand for health care

Despite the availability of a range of providers, and study participants reporting that they were able to access health care when they needed it, age adjusted use rates in our study sites were substantially lower than in high income countries despite a high burden of disease. In Nigeria, respondents consulted a doctor or nurse once every three years to once every 6 to 9 months in Bangladesh. Yet in the United States the crude consultation rate was 3.9 visits per person-year,[26] and the United Kingdom it was 5.5 visits per person-year.[27] Not only is use in our study population lower than in HIC, but there is evidence that health needs are not being met. For instance, very few visits were reported for chronic conditions and follow-up despite the prevalence of chronic illness, which has been reported to be high in other slum populations.[28] Lack of consultation for symptoms that do not cause immediate distress may be a reason that patients presenting with cancer in LMICs are much more likely than in HICs to be in stage 3 or 4.[29,30]

Policy recommendations

Our findings, interpreted in the context of the literature, provide the basis for an emerging policy to improve access to high-quality care in urban areas. First, while in rural areas the predominant consideration is often the existence of local services, in urban areas there are a wide variety of services in close proximity. The imperative in urban areas should therefore be to make better use of the services that already exist. For example, we have found a relatively high use of pharmacies rising to two thirds of all health care contacts in Bangladesh.[11] This suggests, for example, that they might provide a good focus for dissemination of preventive health advice.

Second, our findings on expressed preference show that, in the context of the city, distance to facilities does not significantly suppress demand. These observations suggests that it might be a mistake to pursue, in urban areas, a policy to ensure yet closer location of many services to where people live. Such services would inevitably be more dispersed into small and “single-handed”

For peer review only - http://bmjopen.bmj.com/site/about/guidelines.xhtml

providers where care has been shown to be of low quality (even when the practitioners are medically qualified[6]), and that training has little effect on improving care quality.[7]

Third, overall use is low while people with serious disease such as cancer and TB present late. At the supply side this should be tackled by improving care quality, such as improved diagnosis, as suggested above and also by mitigating the main cost – namely medicines. On the demand side there is a need to continue research into barriers to appropriate health seeking for symptoms of serious disease.

Strengths and Limitations

The response rates differed by study site. We made up to three callbacks for each sampled household to minimize selection bias, but non-responsive or non-consenting households may have differed from those that participated, particularly as slums can be highly dynamic places. There only exist one longitudinal study of slum populations,[31] while it does not capture healthcare use statistics, it does suggest the material circumstances and levels of health spending have not varied significantly year-to-year[32] (the Covid pandmemic notwithstanding). Nevertheless, the findings between slums in this article are qualitatively similar in terms of behaviour and agree with other studies in this area, thus we believe these results can be used to guide policy for these populations. The age standardized consultation rates were based on the INDPETH and WHO reference populations[17] enabling us to make age-adjusted comparisons between study sites. However, the reference population may be out of date and may not be the most appropriate population for broader international comparisons. However, estimated rates differed little between reference populations. Clinical officers were not included in this study as a category, and we do not how respondents might identify or categorise them (e.g. as a "doctor" or "other"); further research is required into the role of clinical officers in these locations. Indeed, a limitation of our study was that the type of provider (nurse or doctor) was self-reported, which means the provider could be misclassified. Further research is required to determine what effect this might have for

For peer review only - http://bmjopen.bmj.com/site/about/guidelines.xhtml

healthcare use surveys like this. We cannot make a reliable comparison of individuals based on their health status as no reliable clinical or epidemiological indicators were captured. Differences between sites may well be attributable to large differences in population health, however we suggest that this would be unlikely. Any observed relationships, or lack thereof, may be driven by other underlying processes and confirmatory studies are needed to address some of the questions raised here. Finally, slum populations are often highly mobile, which could result in rapidly shifting population characteristics. The cross-sectional data here may therefore not provide a complete picture of slums around the world and we caution against making broad generalizations.

Conclusion

There have been big 'pro-poor' improvements in key indicators, such as infant and under-five mortality, in the last 20 years. But these have been largely achieved by preventive services such as immunization, improved nutrition, and rehydration therapy. Further improvements, for example in cancer care, will require improved clinical care along the pathways from seeking care for the first symptom to definitive treatment. We hope that our findings contribute to the debate about how to improve primary care services by supporting understanding of when, where, and how the residents of poorer urban areas make contact with the health system when health needs arise.

REFERENCES

1 WHO; UNICEF Declaration of Astanap.8-9. Declaration of Astana. *Glob Conf Prim Heal Care* 2018.

2 Kruk ME, Gage AD, Arsenault C, *et al.* High-quality health systems in the Sustainable Development Goals era: time for a revolution. *Lancet Glob Heal* 2018; **6**: e1196–252.

3 McPake B, Hanson K. Managing the public-private mix to achieve universal health coverage. *Lancet (London, England)* 2016; **388**: 622–30.

4 Ezeh A, Oyebode O, Satterthwaite D, *et al.* The history, geography, and sociology of slums and the health problems of people who live in slums. *Lancet* 2016; published

For peer review only - http://bmjopen.bmj.com/site/about/guidelines.xhtml

online Oct. DOI:10.1016/S0140-6736(16)31650-6.

5 Lilford RJ, Oyebode O, Satterthwaite D, *et al.* Improving the health and welfare of people who live in slums. *Lancet* 2017; **389**: 559–70.

6 Das J. The Quality of Medical Care in Low-Income Countries: From Providers to Markets. *PLoS Med* 2011; **8**: e1000432.

7 Das J, Chowdhury A, Hussam R, Banerjee A V. The impact of training informal health care providers in India: A randomized controlled trial. *Science (80-)* 2016; **354**: aaf7384–aaf7384.

8 Kruk ME, Gage AD, Joseph NT, Danaei G, García-Saisó S, Salomon JA. Mortality due to low-quality health systems in the universal health coverage era: a systematic analysis of amenable deaths in 137 countries. *Lancet* 2018; **392**: 2203–12.

9 Chen Y-F, Park J-E, Harris B, Ajisola M, Griffiths F, Improving Health in Slums Collaborative. Scoping review protocol: Factors associated with accessing health care and provision of health services for residents of slums in low and middle income countries. 2021 https://osf.io/694t2.

10 Crocker-Buque T, Mindra G, Duncan R, Mounier-Jack S. Immunization, urbanization and slums – a systematic review of factors and interventions. *BMC Public Health* 2017; **17**: 556.

11 Khan MMH, Grübner O, Krämer A. Frequently used healthcare services in urban slums of Dhaka and adjacent rural areas and their determinants. *J Public Health (Bangkok)* 2012; **34**: 261–71.

12 Owusu-Ansah FE, Tagbor H, Togbe MA. Access to health in city slum dwellers: The case of Sodom and Gomorrah in Accra, Ghana. *African J Prim Heal Care Fam Med* 2016; **8**. DOI:10.4102/phcfm.v8i1.822.

13 Improving Health in Slums Collaborative. A protocol for a multi-site, spatially-referenced household survey in slum settings: methods for access, sampling frame construction, sampling, and field data collection. *BMC Med Res Methodol* 2019; **19**: 109.

14 UN Habitat. The Challenge of the Slums: Global Report on Human Settlements 2003. London, UK, 2010.

15 Chipeta M, Terlouw D, Phiri K, Diggle P. Inhibitory geostatistical designs for spatial prediction taking account of uncertain covariance structure. *Environmetrics* 2017; **28**: e2425.

16 World Health Organization. WHO World Health Survey. 2007. https://www.who.int/healthinfo/survey/en/ (accessed March 6, 2020).

17 Ahmad OB, Boschi-pinto C, Lopez AD. Age standardization of rates: a new WHO standard. *GPE Discuss Pap Ser* 2001.

18 Sankoh O, Sharrow D, Herbst K, *et al.* The INDEPTH standard population for low- and middle-income countries, 2013. *Glob Health Action* 2014; **7**: 23286.

19 Fox JT, Kim K il, Ryan SP, Bajari P. The random coefficients logit model is identified. *J Econom* 2012; **166**: 204–12.

20 J. Borah B. A mixed logit model of health care provider choice: analysis of NSS data for

For peer review only - http://bmjopen.bmj.com/site/about/guidelines.xhtml

rural India. *Health Econ* 2006; **15**: 915–32.

21 Oyebode O, Kandala N-B, Chilton PJ, Lilford RJ. Use of traditional medicine in middle-income countries: a WHO-SAGE study. *Health Policy Plan* 2016; **31**: 984–91.

22 Coarasa J, Das J, Gummerson E, Bitton A. A systematic tale of two differing reviews: Evaluating the evidence on public and private sector quality of primary care in low and middle income countries. *Global Health* 2017. DOI:10.1186/s12992-017-0246-4.

23 Cameron A, Ewen M, Ross-Degnan D, Ball D, Laing R. Medicine prices, availability, and affordability in 36 developing and middle-income countries: a secondary analysis. *Lancet* 2009; **373**: 240–9.

24 Bitton A, Fifield J, Ratcliffe H, *et al.* Primary healthcare system performance in low-income and middle-income countries: a scoping review of the evidence from 2010 to 2017. *BMJ Glob Heal* 2019; **4**: e001551.

25 Watson SI, Wroe EB, Dunbar EL, *et al.* The impact of user fees on health services utilization and infectious disease diagnoses in Neno District, Malawi: a longitudinal, quasi-experimental study. *BMC Health Serv Res* 2016; **16**: 595.

26 OECD. Doctor's Consultations. OECD Data. 2020. https://data.oecd.org/healthcare/doctors-consultations.htm (accessed March 6, 2020).

27 Hobbs FDR, Bankhead C, Mukhtar T, *et al.* Clinical workload in UK primary care: a retrospective analysis of 100 million consultations in England, 2007-14. *Lancet (London, England)* 2016; **387**: 2323–30.

28 McNairy ML, Tymejczyk O, Rivera V, *et al.* High Burden of Non-communicable Diseases among a Young Slum Population in Haiti. *J Urban Heal* 2019; **96**: 797–812.

29 Rivera-Franco MM, Leon-Rodriguez E. Delays in Breast Cancer Detection and Treatment in Developing Countries. *Breast Cancer Basic Clin Res* 2018; **12**: 117822341775267.

30 Stewart TS, Moodley J, Walter FM. Population risk factors for late-stage presentation of cervical cancer in sub-Saharan Africa. *Cancer Epidemiol* 2018; **53**: 81–92.

31 African Population and Health Research Center. Nairobi Urban Health Demographic Surveillance System. 2018. http://aphrc.org/post/projects/nairobi-urban-health-and-demographic-surveillance-system-nuhdss (accessed Aug 3, 2018).

32 Buigut S, Ettarh R, Amendah DD. Catastrophic health expenditure and its determinants in Kenya slum communities. *Int J Equity Health* 2015; **14**: 46.

Figure legends

Figure 1. Age-adjusted (to INDEPTH population) visit rates per person-year to different outpatient care providers for adults and children (under 12) for new and existing conditions.

Figure 2. Proportion of outpatient doctor and nurse consultations for a new condition by facility type

For peer review only - http://bmjopen.bmj.com/site/about/guidelines.xhtml

Figure 3. Proportion of outpatient consultations for a new condition by doctor or nurse

Figure 4. Predicted proportions of visits to different provider types for an outpatient doctor and nurse consultation if price and travel time were all equivalent versus actual proportions of visits.

For peer review only

For peer review only - http://bmjopen.bmj.com/site/about/guidelines.xhtml

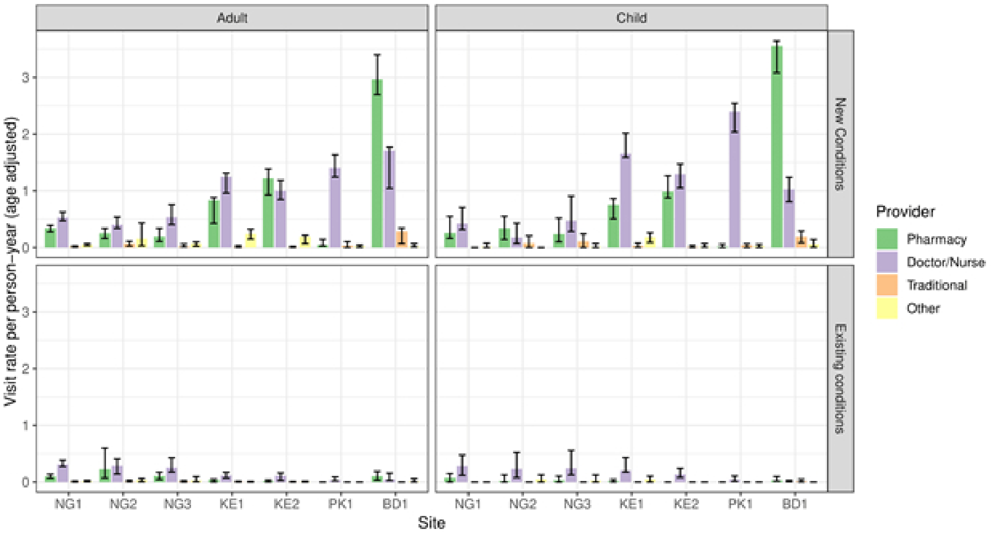

Age-adjusted (to INDEPTH population) visit rates per person-year to different outpatient care providers for adults and children (under 12) for new and existing conditions.

417x225mm (38 x 38 DPI)

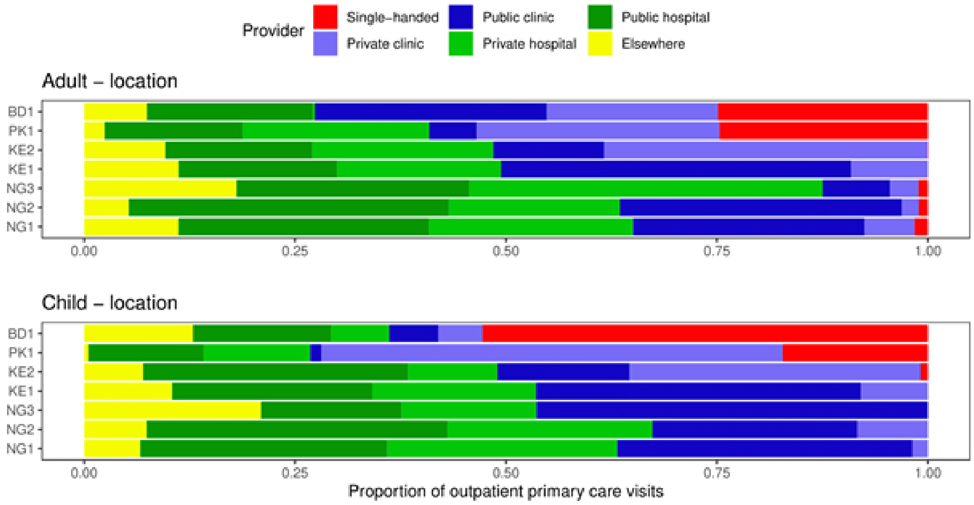

Proportion of outpatient doctor and nurse consultations for a new condition by facility type

417x214mm (38 x 38 DPI)

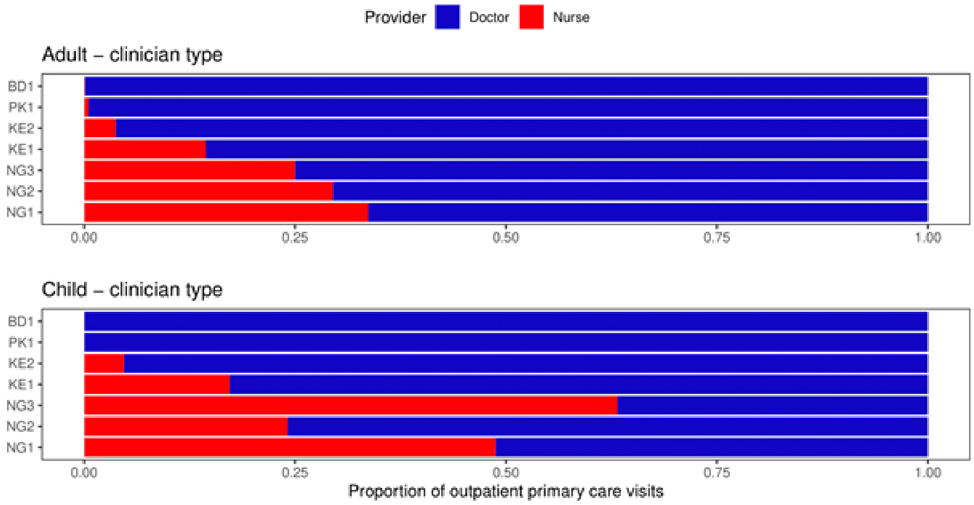

Proportion of outpatient consultations for a new condition by doctor or nurse

417x213mm (38 x 38 DPI)

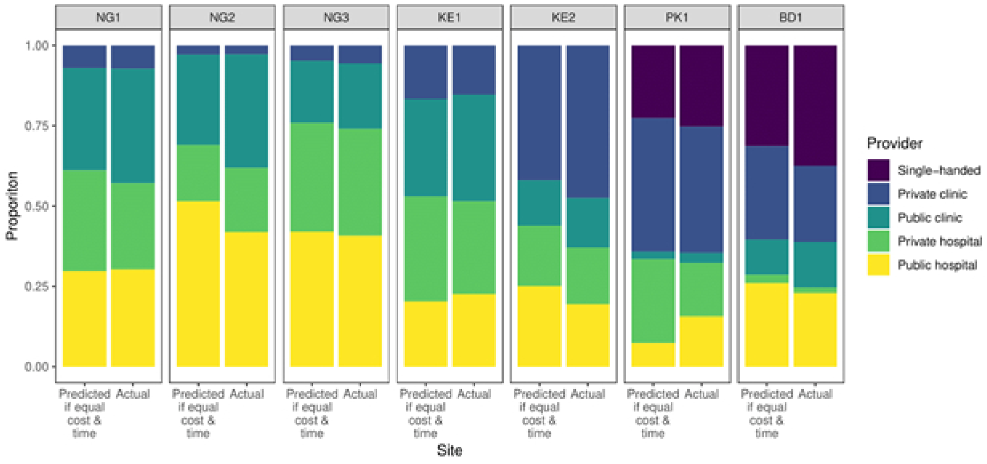

Predicted proportions of visits to different provider types for an outpatient doctor and nurse consultation if price and travel time were all equivalent versus actual proportions of visits.

417x195mm (38 x 38 DPI)

SUPPLEMENTARY INFORMATION

A Health system indicators

Table A1 reports major health system and population health indicators for the participating countries.

Table A1. Key indicators of health care systems in participating countries (Source: World Bank and WHO Global Health Observatory[1])

Key indicators	Nigeria	Kenya	Pakistan	Bangladesh
Life expectancy	54	66	67	72
GDP per capita (PPP; Int$)	5,991	3,468	5.572	4,372
Under five mortality (per 1,000 births)	120	41	69	30
Out of pocket expenditure as % of all health expenditures	75%	28%	65%	72%
Government health expenditure per capita (Int$)	27	52	40	16
Proportion with expenditures on healthcare greater than 10% of total household expenditure	15%	5%	13%	25%
Antenatal care coverage – at least four visits	49%	105%	51%	37%
ARV coverage among those with HIV	53%	68%	10%	22%
Hib (Hib3) coverage among one-year-olds	57%	92%	75%	98%
Tetanus coverage among neonates	60%	88%	85%	98%
Tuberculosis effective treatment coverage	20%	39%	64%	58%
Average of 13 International Heath Regulation core capacity scores	51	58	51	78

GDP: gross domestic product; Hib: Haemophilus influenzae type b vaccine; Int$: international dollar; PPP: purchasing power parity

For peer review only

B Supplementary information on methods

B1 Definitions of health care facilities

Table B1 Definitions of healthcare facilities used in the mapping and surveying of healthcare facilities at each site

Facility type	Definition
Clinic/centre	A facility used for the diagnosis and treatment of outpatients. Allopathic only. Subcategories include specialist clinics: vaccination; family planning; eye and vision; ear, nose, and throat; maternity and antenatal care; palliative care; and general primary or community care.
Dental clinic	A facility providing dentistry/dental care.
Hospital/secondary care facility	A health care organization that has a governing body, an organized medical staff and professional staff and inpatient facilities and provides medical nursing and related services for ill and injured patients 24 hrs per day, seven days per week
Residential or nursing care facility	Facilities that provide inpatient care specializing in nursing or long-term residential care but which are not hospital
Laboratory or sonologist clinic	A room or building equipped for testing, researching, or studying fluids, materials, or tissues from patients.
Pharmacy or medical store	A facility used by pharmacists for the compounding and dispensing of medicinal preparations and other associated professional and administrative services
Transportation services	Provider who moves a patient, specimen, or equipment from one location or another
General shop or kiosk selling healthcare products	Non-specialised vendor without healthcare professional selling health care products such as pain killers, first aid, or spectacles
Traditional practitioner	A practitioner of non-allopathic, traditional, alternative, or faith-based health services. Sub-categories include: traditional medicine, faith-based services, homeopathy, acupuncture, bone healing, and cupping.
Traditional products vendor	Vendor specialising in traditional (non-allopathic) medical products such as herbs.

B2 Supplementary Survey in Site PK1

An error with the electronic survey forms was identified following data collection in site PK1. The household survey form was completed successfully, including the household roster, however due to an error in the code to generate a randomly sampled adult the first recorded adult was instead sampled. This resulted in an almost exclusively male sample, which was not identified in the field at the time. After identification of this error we aimed to complete a set of individual surveys with randomly sampled women from the household rosters. However, the Covid-19 pandemic had started, so a telephone survey was deemed to be safer for the field workers. We sampled 400

For peer review only

households with at least one adult women and who had provided consent and contact details for follow-up from the original sample of households. Field workers then followed the same procedures as the main survey. Survey weights were modified for the sample from PK1 to reweight gender back to the population proportion.

B3 Statistical methods

Choice model

Choices

We define the choice set facing individual as: private clinic, public clinic, private hospital, and public hospital. In sites BD1 and PK1 we also include “private office” as a fifth option as a number of visits were recorded to these locations, whereas there were little to no visits to this type of provider in the other sites.

Choice-varying covariates

We consider two covariates that vary for each individual and choice: the price of the visit and the time to travel to the provider. The price includes consultation fees and transport cost. The price is reported in local currency (Naira, Kenyan Shillings, Pakistani Rupees, Taka). The time to provider is the travel time reported for the visit in minutes. We standardize both cost and time (dividing by the standard deviation) to ensure all variables are on the same scale to facilitate model fitting.

Individual-level covariates

From the survey data we extract the following individual level variables: age, sex, secondary education or not, if the visit was for an acute or communicable condition (excluding HIV), if the visit was for generalised or chronic pain, and if the monthly consumption expenditure was above Int$100 per person per month. For use in the models we standardize the age variable to facilitate model fitting.

Statistical Model

We take a Bayesian approach to model specification and fitting given the complex hierarchical structure of the model.

For peer review only

Each individual $i \in 1{:}N$ chooses from $k \in 1{:}K$ possible options (K is either 4 or 5). The healthcare provider options are described by a vector of choice attributes for each individual $X_{ik} = \left[C_{ik}, C_{ik}^2, T_{ik}\right]'$. This vector includes the cost, cost squared, and time. We include cost squared to allow for more flexibility between demand and price. Each individual has "part worth" preferences over the choice attributes β_i so that the utility of each choice for each individual is:

$$u_{ik} = \theta_k + X_{ik}\beta_i + u_{ik}$$

where u_{ik} is IID Gumbel distributed and θ_k are choice constant terms. Given the assumed distribution of the random error, the probability an individual chooses choice k is

$$\Pr(y_{ik} = k | X_{ik}, \beta_i, \theta_k) = \frac{\exp(\theta_k + X_{ik}\beta_i)}{\sum_{j=1}^{K} \exp\left(\theta_j + X_{ij}\beta_i\right)}$$

For more information see[2].

Hierarchical prior on model parameters

The model parameters β_i are allowed to vary by individual – they are so-called "random parameters". To model these parameters we use a hierarchical prior that "partially pools" information across individuals. We also allow these parameters to vary according to a vector of individual-level covariates W_i and further, we allow for correlation between parameters so that, for example, individuals more sensitive to price might be less sensitive to distance. In particular,

$$\beta_i \sim MVN(\beta + \Gamma W_i, \Sigma)$$

where β are mean values, Γ is a matrix that "loads" the individual covariates on to the parameters, and Σ is a covariance matrix. We can decompose the covariance matrix as $\Sigma = \text{diag}(\tau)\Omega\,\text{diag}(\tau)$, where τ is a scale vector and Ω is the correlation matrix of the variation across individuals. The correlation matrix can be further decomposed as $\Omega = L_\Omega L_\Omega'$ where L_Ω is the lower-triangular Cholesky decomposition. Therefore,

$$\beta_i = \beta + \Gamma W_i + \text{diag}(\tau) L_\Omega z_i$$

$$z_i \sim N(0,1)$$

Missing data

We use an imputation-type procedure for the costs and times that we do not observe, i.e. for the choices not taken. The model below is part of the overall model and is estimated at the same time

For peer review only

so that the uncertainty in the imputed costs and times is carried through to the overall results. We explain the procedure for costs but use an identical model for times. Given that both variables are (often heavily) right-skewed we use log costs and time for the missing data models. We observe N values for log cost, one for each individual: $c_{ik}^{(obs)}$. We specify:

$$c_{ik}^{(obs)} = \mu_k + Z_i\gamma + u_i$$

where μ_k is a choice-specific intercept, Z_i is a vector of individual level covariates that may predict the cost of the visit (age, age squared, sex, reason for visit), γ are model parameters, and $u_i \sim N(0, \sigma_u^2)$ are IID error terms. We fit the above model using the observed data and then for the unobserved costs we sample from:

$$c_{ik}^{(mis)} \sim N(\mu_k + Z_i\gamma, \sigma_u^2)$$

so that the cost covariate is

$$C_{ik} = \begin{cases} \exp\left(c_{ik}^{(obs)}\right) & if\ y_{ik} = 1 \\ \exp\left(c_{ik}^{(mis)}\right) & if\ y_{ik} = 0 \end{cases}$$

Priors

We specify weakly informative priors on the model parameters (all N(0,1) except for the correlation matrix for which we use the lkj prior).

Estimation

We use Stan 2.19 to fit the model[3].

Elasticity of demand

To quantify the response to price or travel time we calculate the price and travel time elasticity of demand. We do this by calculating the arc elasticity:

$$\frac{(\mathrm{Pr1} - \mathrm{Pr0})/((\mathrm{Pr1} + \mathrm{Pr0})/2)}{(p_1 - p_0)/((p_1 + p_0)/2)}$$

where p_1 and p_0 are different prices or times and $\mathrm{Pr1}$ and $\mathrm{Pr0}$ are the probabilities of visiting a provider under each of the two prices or times, respectively.

For peer review only

C Additional Results

Table C1. Respondent care needs and outpatient primary care use, N(%)

Outcome	NG1	NG2	NG3	KE1	KE2	PK1	BD1
Adults							
Total	1,278 (100%)	840 (100%)	802 (100%)	1,008 (100%)	1,085 (100%)	1,112 (100%)	990 (100%)
Needed healthcare in the previous 12 months	772 (60%)	490 (58%)	418 (52%)	710 (70%)	906 (84%)	623 (56%)	959 (97%)
Received care when last needed	762 (60%)	481 (57%)	404 (50%)	674 (67%)	884 (81%)	596 (54%)	918 (93%)
Outpatient visit in last 12 months	626 (49%)	367 (44%)	309 (39%)	665 (66%)	867 (80%)	531 (48%)	900 (91%)
Outpatient visit to doctor or nurse	391 (31%)	215 (26%)	201 (25%)	405 (40%)	377 (35%)	498 (45%)	257 (26%)
Outpatient visit to doctor or nurse for new condition or routine visit	293 (23%)	132 (16%)	151 (19%)	310 (31%)	315 (29%)	291 (26%)	174 (18%)
Children (under 12)							
Total	128 (100%)	69 (100%)	79 (100%)	537 (100%)	421 (100%)	528 (100%)	658 (100%)
Needed healthcare in the previous 12 months	68 (53%)	29 (42%)	41 (52%)	482 (90%)	368 (87%)	409 (77%)	635 (97%)
Received care when last needed	68 (53%)	28 (41%)	41 (52%)	466 (87%)	363 (86%)	397 (75%)	627 (95%)
Outpatient visit in last 12 months	53 (41%)	24 (35%)	39 (49%)	462 (86%)	358 (85%)	374 (71%)	608 (92%)
Outpatient visit to doctor or nurse	36 (28%)	13 (19%)	24 (30%)	320 (60%)	205 (49%)	367 (70%)	136 (21%)
Outpatient visit to doctor or nurse for new condition	27 (21%)	11 (16%)	19 (24%)	283 (53%)	190 (45%)	329 (62%)	120 (18%)

For peer review only

Table C2. Summary statistics of study populations and national comparisons (DHS surveys – national results and urban results in parentheses where relevant)

Variable		Nigeria				Kenya			Pakistan		Bangladesh	
		NG1	NG2	NG3	DHS 2018	KE1	KE2	DHS 2014	PK1	DHS 2017/8	BD1	DHS 2014
Households												
N (completed)		1,286	845	812	-	1,018	1,089	-	988	-	1,035	-
Response rate (%)		83	69	68	99.3	69	57	99	73	96	94	99
Household size		3.7 (1.9)	3.4 (1.8)	4.2 (2.0)	4.3	3.2 (2.2)	3.4 (1.7)	3.9	5.7 (3.0)	6.6	3.8 (1.8)	4.5
Wealth quintile (%)	Bottom	0	0	0	20 (4.2)	0	0	20 (6)	0	20 (3)	0	20 (7)
	Lower	2	1	1	20 (8.1)	14	0	20 (8)	0	20 (7)	0	20 (6)
	Middle	50	59	23	20 (18.8)	59	53	20 (11)	84	20 (17)	21	20 (12)
	Upper	48	40	76	20 (30.6)	27	46	20 (26)	14	20 (31)	78	20 (26)
	Top	0	0	0	20 (38.4)	7	1	20 (49)	2	20 (42)	1	20 (49)
Monthly expenditure (Int$), median [IQR]	Total	312 [197, 487]	323 [210, 496]	502 [319, 815]	-	193 [131, 305]	187 [133, 295]	-	1,010 [672, 1,471]	--	490 [321, 770]	-
	Per person	97 [59, 162]	106 [69, 172]	144 [94, 231]	-	74 [45, 125]	96 [61, 156]	-	196 [146, 294]	-	110 [80, 162]	-
Individuals												
Age (%)	Under 5	11	9	10	15.4	13	12	14	11	13	11	10
	5 – 19	34	31	33	36.4	37	26	38	30	36	32	34
	20 – 44	36	35	39	32.0	37	51	33	41	34	45	36
	45 – 64	14	17	15	11.8	11	10	11	15	13	10	15
	65 and over	5	8	3	4.1	3	1	4	3	4	2	6
Male (%)		49	47	50	-	50	55	-	50	-	53	-
Education over 18s (%)	Completed primary	13	16	17	24.7	25	23	16	13	9	31	9
	Completed secondary	24	31	34	31.7	13	30	11	9	9	4	4
	Completed tertiary	11	6	20	12.1	5	6	7	0	12	4	10
Currently working over 18 (%)		72	76	74	75.5	65	77	81	57	96	75	98

Table C3. Outpatient primary care consultation rates and outpatient doctor and nurse consultation rates (visits per person-year (95% CI)).

Site	Outpatient consultation (new conditions)			Outpatient consultation (all conditions)		
	**Crude**	**WHO age adjusted**	**INDEPTH age adjusted**	**Crude**	**WHO age adjusted**	**INDEPTH age adjusted**
Adults						
NG1	0.31 (0.29, 0.33)	0.30 (0.28, 0.32)	0.30 (0.28, 0.32)	0.42 (0.40, 0.44)	0.42 (0.40, 0.44)	0.40 (0.38, 0.42)
NG2	0.25 (0.23, 0.27)	0.21 (0.19, 0.23)	0.20 (0.18, 0.22)	0.39 (0.35, 0.43)	0.32 (0.30, 0.34)	0.29 (0.27, 0.31)
NG3	0.21 (0.19, 0.23)	0.23 (0.21, 0.25)	0.22 (0.20, 0.24)	0.28 (0.26, 0.30)	0.31 (0.29, 0.33)	0.29 (0.27, 0.31)
KE1	0.77 (0.73, 0.81)	0.77 (0.73, 0.81)	0.72 (0.68, 0.76)	1.07 (1.03, 1.11)	1.17 (1.13, 1.21)	1.04 (1.00, 1.08)
KE2	0.76 (0.72, 0.80)	0.73 (0.69, 0.77)	0.70 (0.66, 0.74)	0.93 (0.89, 0.97)	1.06 (1.02, 1.10)	0.95 (0.91, 0.99)
PK1	0.60 (0.56, 0.64)	0.58 (0.54, 0.62)	0.56 (0.52, 0.60)	0.79 (0.75, 0.83)	0.85 (0.81, 0.89)	0.77 (0.73, 0.81)
BD1	1.08 (1.04, 1.12)	1.21 (1.15, 1.26)	1.16 (1.12, 1.20)	1.52 (1.46, 1.58)	1.73 (1.67, 1.79)	1.59 (1.53, 1.65)
Children (under 12)						
NG1	0.23 (0.15, 0.31)	-	-	0.29 (0.19, 0.39)	-	-
NG2	0.12 (0.04, 0.20)	-	-	0.15 (0.05, 0.25)	-	-
NG3	0.26 (0.14, 0.38)	-	-	0.34 (0.20, 0.48)	-	-
KE1	1.51 (1.41, 1.61)	-	-	1.74 (1.62, 1.86)	-	-
KE2	1.21 (1.11, 1.31)	-	-	1.30 (1.18, 1.42)	-	-
PK1	1.68 (1.56, 1.80)	-	-	1.85 (1.73, 1.97)	-	-
BD1	0.91 (0.83, 0.99)	-	-	1.04 (0.96, 1.12)	-	-
Children (under 5)						
NG1	0.24 (0.10, 0.38)	-	-	0.30 (0.14, 0.46)	-	-
NG2	0.13 (0.00, 0.27)	-	-	0.21 (0.03, 0.39)	-	-
NG3	0.38 (0.14, 0.62)	-	-	0.40 (0.16, 0.64)	-	-
KE1	2.17 (1.97, 2.37)	-	-	2.57 (2.35, 2.79)	-	-
KE2	1.62 (1.44, 1.80)	-	-	1.68 (1.50, 1.86)	-	-
PK1	2.33 (2.11, 2.55)	-	-	2.46 (2.24, 2.68)	-	-
BD1	1.35 (1.21, 1.49)	-	-	1.50 (1.36, 1.63)	-	-

For peer review only

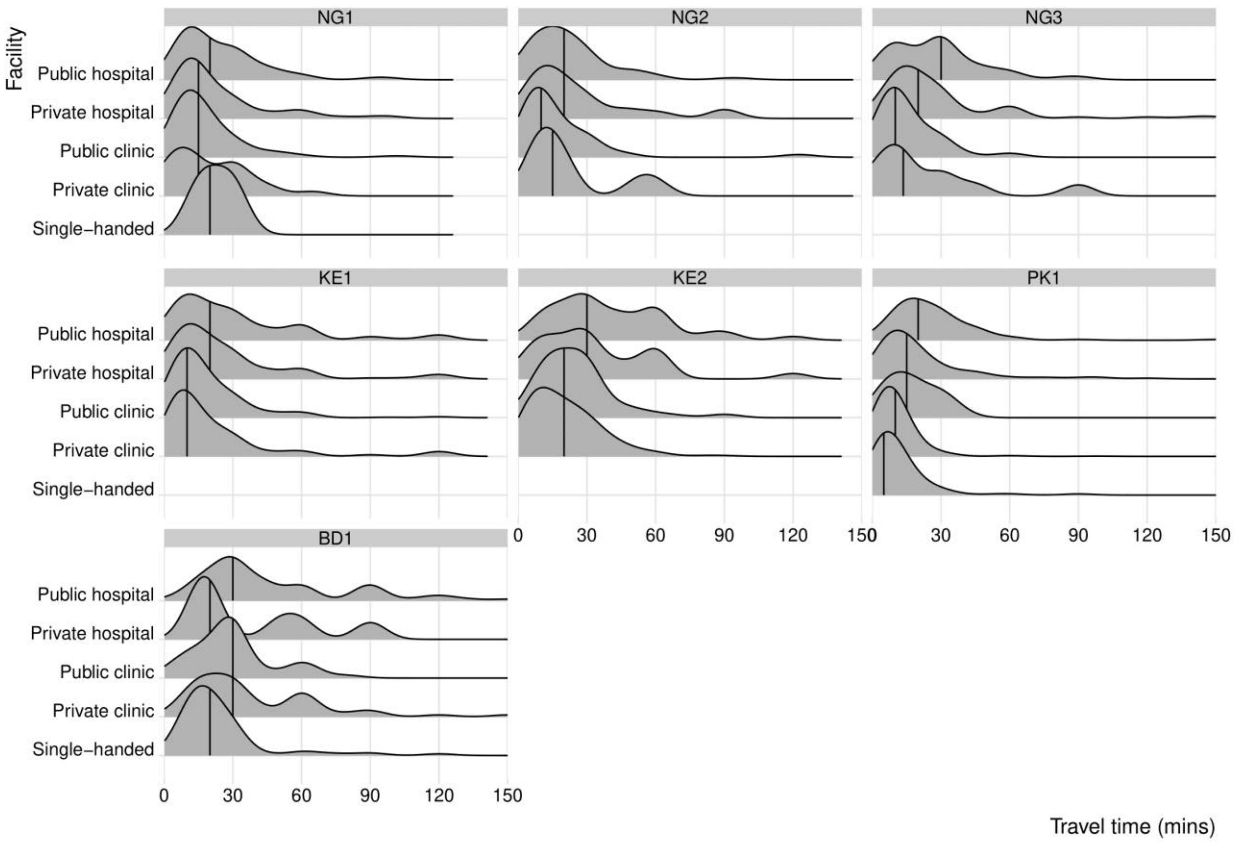

Figure C1 Distribution of reported travel times to different providers by study site with vertical lines indicating the median of the distribution.

For peer review only - http://bmjopen.bmj.com/site/about/guidelines.xhtml

Figure C2 Reported "primary reason" for visiting the doctor or nurse.

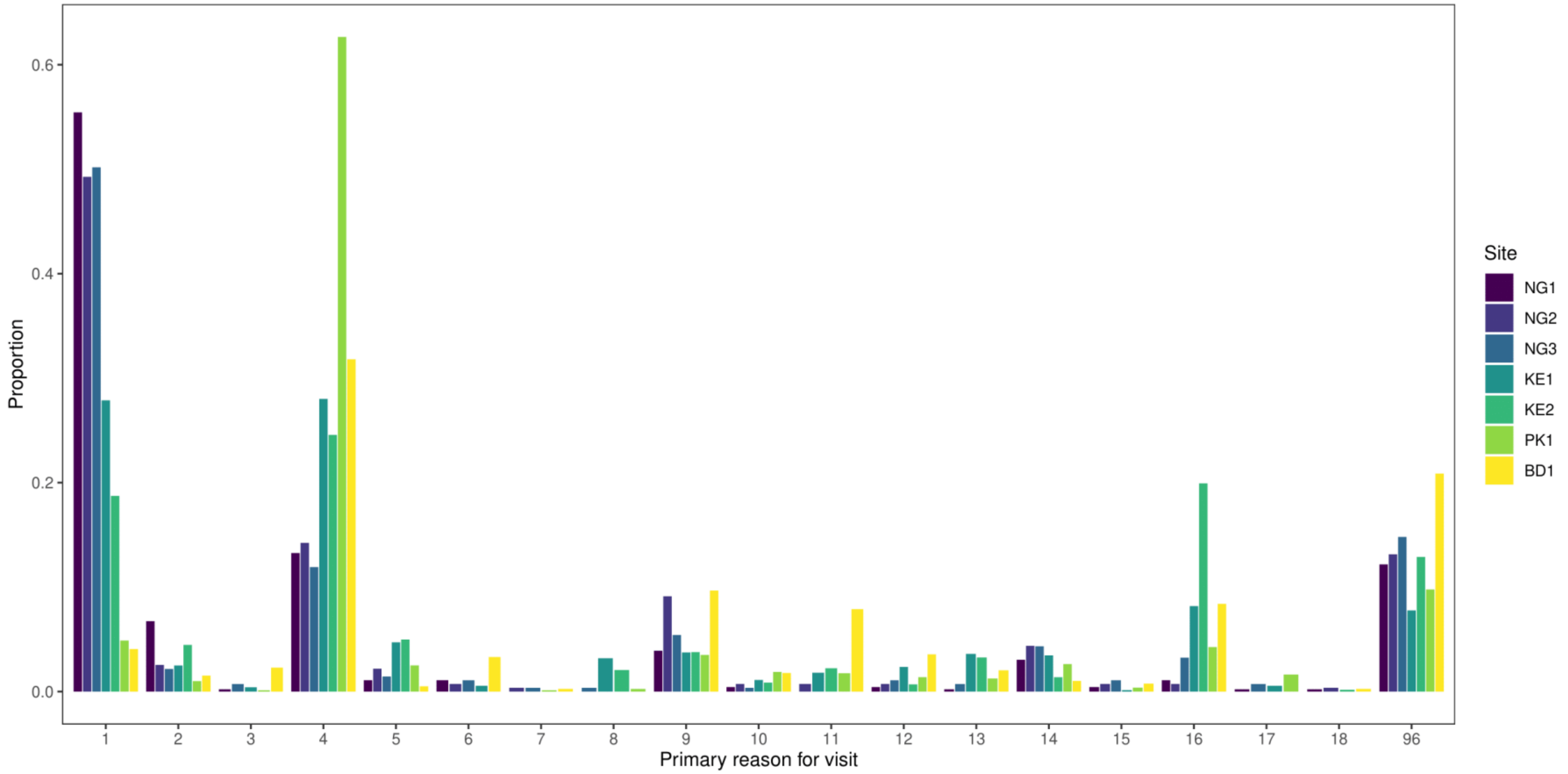

The reasons for visiting were: 1 Communicable disease (infections, malaria, tuberculosis, HIV), 2 Maternal and perinatal conditions (pregnancy), 3 Nutritional deficiencies, 4 Acute conditions (diarrhoea, fever, flu, headaches, cough, other), 5 Injury (not work related, see 8 below), 6 Surgery, 7 Sleep problems, 8 Occupation/work related condition/injury, 9 Chronic pain in your joints / arthritis (joints, back, neck), 10 Diabetes or related complications, 11 Problems with your heart, including unexplained pain in chest, 12 Problems with your mouth, teeth or swallowing, 13 Problems with your breathing, 14 High blood pressure / hypertension, 15 Stroke / sudden paralysis of one side of body, 16 Generalised pain (stomach, muscle or other non-specific pain), 17 Depression or anxiety, 18 Cancer, 96 Other (Specify)

Table C4 Percentage of respondents reporting reasons for choosing a healthcare providers by provider type

	Nigeria			Kenya		Pakistan	Bangladesh
	**NG1**	**NG2**	**NG3**	**KE1**	**KE2**	**PK1**	**BD1**
Nearness of the facility	63	69	57	78	72	61	54
Service providers are cordial	34	24	32	21	22	29	65
Good services available	38	32	44	31	37	37	95
Short waiting times	17	11	16	7	3	9	7
Qualified doctors are available	24	29	35	22	17	51	26
Low fees/treatment costs	28	33	43	36	24	36	39
Good waiting arrangement	12	9	15	3	2	7	5
Confidentiality is maintained	13	10	21	1	1	18	0
Don't know where else to go	7	15	7	12	6	5	3
Medicines are available	21	17	25	35	22	9	4
Diagnostics are available	11	12	11	13	4	1	11
Recommendation	11	6	11	16	8	9	20

For peer review only

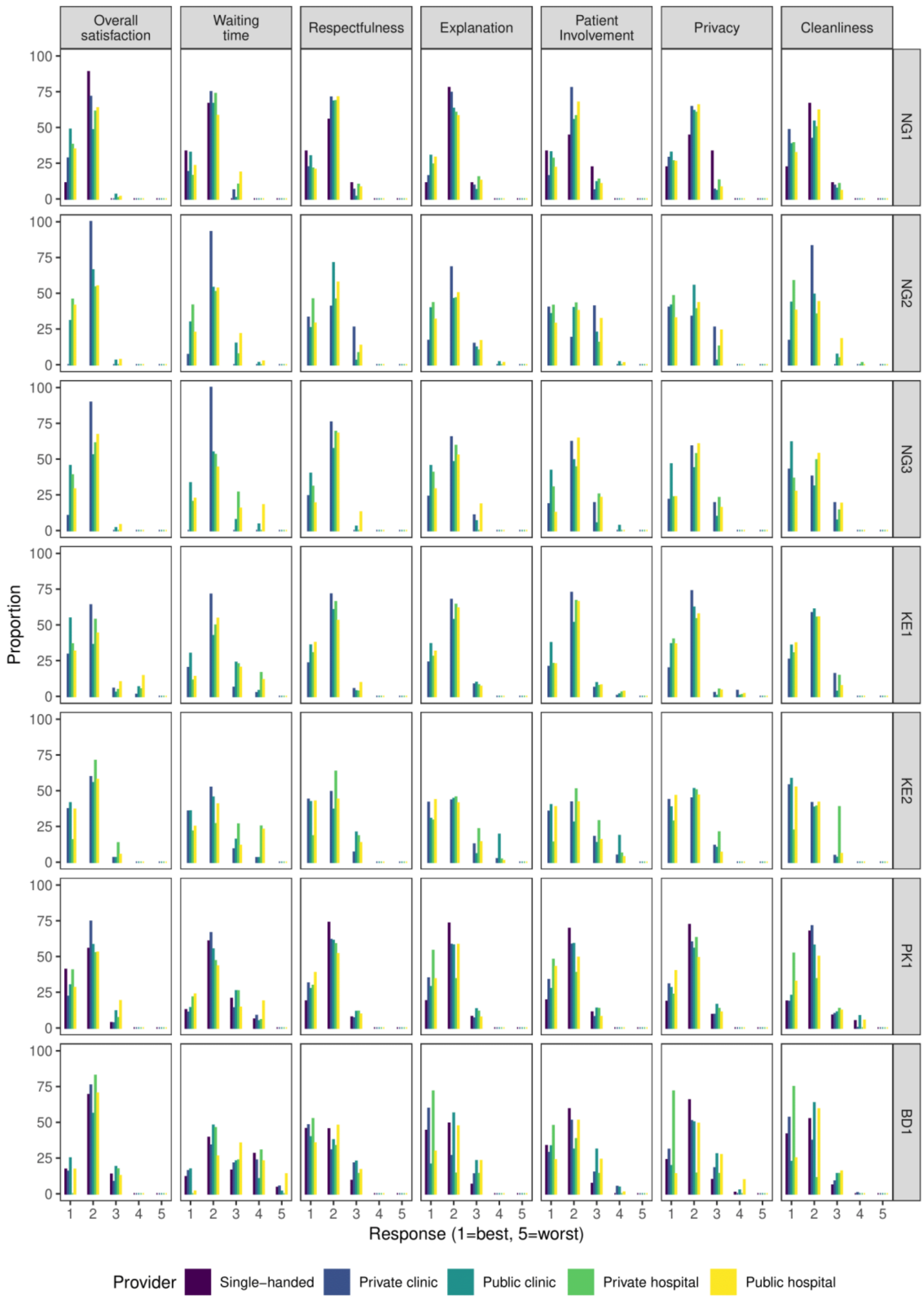

Figure C2. Satisfaction with outpatient consultations by type of facility and by site. Responses were on five-point Likert scales, either Very satisfied to very dissatisfied (Overall satisfaction) or Very good to very bad (Other questions).

REFERENCES

1 World Health Organization. Global Health Observatory. 2021.https://www.who.int/data/gho (accessed 6 Jun 2021).

2 J. Borah B. A mixed logit model of health care provider choice: analysis of NSS data for rural India. *Health Econ* 2006;**15**:915–32. doi:10.1002/hec.1166

3 Carpenter B, Gelman A, Hoffman M, *et al.* Stan: A Probabilistic Programming Language. *J Stat Softw* 2016;**VV**.

For peer review only

[Name of Country]
[Name of Slum]

Individual Questionnaire

[To be completed by the randomly selected adult (18+) from within the household. Note: first complete introduction and consent]

The next set of questions will ask you about the last time that you needed health care, whether or not you received that care. Please think back to the last time.

Module 4: Health Service Use

Q401	Have you needed health care in the last 12 months (whether or not you received health care)?<br><br>[INFORM THE RESPONDANT THAT THIS CAN BE INPATIENT CARE, OUTPATIENT CARE, MEDICATION, TRADITIONAL CARE, OR ANY OTHER INTERVENTION FOR THEIR HEALTH..]	1. Yes<br>2. No	Q402<br>Q443
Q402	The last time you needed health care, did you get health care?	1. Yes…………………………………………….-><br>2. No…………………………………………….->	Q403<br>Q402
Q402b<br><br>[ONLY IF 402 = "NO"]	Which reason(s) best explains why you did not get health care?<br><br>[CHOOSE ALL THAT THE RESPONDENT INDICATES]	1. Could not afford the cost of the visit<br>2. No transport available<br>3. Could not afford the cost of transport<br>4. You were previously badly treated<br>5. Could not take the time off work or had other commitments<br>6. The health care provider's drugs or equipment were inadequate<br>7. The health care provider's skills were inadequate<br>8. You did not know where to go<br>9. You tried but were denied health care<br>10. You thought you were not sick enough<br>97. Other, please specify	

Inpatient Hospital Care

The next two questions ask about any overnight stay in a hospital or other health care facility you had in the last year.

Q403	In the last 12 months, have you ever stayed overnight in a hospital or long-term health care facility?<br>[Select all that apply]	1. Yes, a hospital<br>2. Yes, long-term care facility<br>3. Yes, other provider (Please specify)<br><br>4. No ………………………………………………… →	 <br><br><br>Q423

For peer review only - http://bmjopen.bmj.com/site/about/guidelines.xhtml

[Name of Country]
[Name of Slum]

Q404	Over the last 12 months, how many different times were you a patient in a hospital / long-term care facility for at least one night?	☐☐ times<br>98. Don't know	
		IF "00" (NO OVERNIGHT STAYS) .... →	Q423

I would like you to ask about your last inpatient stay only.

Q405	What type of hospital or facility was it? Remember we are asking now about your last (most recent) overnight stay.<br><br>[ONE ANSWER ONLY]	1. Public hospital<br>2. Private hospital<br>3. Charity or church-run hospital<br>4. Old person's home or long-term care facility<br>97. Other, please specify<br><br>[BANGLADESH: remove option 3, replace with "NGO clinical or health care facility and community health clinic"]	
Q405a	Please name the facility	Free text	
Q405b	Which reason(s) best describes why you chose this health care provider?<br>[CHOOSE ALL THAT THE RESPONDENT INDICATES]	1. Nearness of the facility<br>2. Service providers are cordial<br>3. Good service available<br>4. Short waiting time<br>5. Qualified doctors available<br>6. Low fees/low treatment cost<br>7. Good waiting arrangement<br>8. Confidentiality is maintained<br>9. Do not know where else to go<br>10. Medicine is also available<br>11. Availability of diagnostic service<br>12. Recommendation from someone<br>97. Other (please specify)	
Q405c	Which reason best describes why you were last hospitalised?<br><br>[RESPONDENT CAN SELECT ONLY ONE MAIN REASON FOR VISIT.]<br>1. Communicable disease (infections, malaria, tuberculosis, HIV)<br>2. Maternal and perinatal conditions (pregnancy)<br>3. Nutritional deficiencies<br>4. Acute conditions (diarrhoea, fever, flu, headaches, cough, other)<br>5. Injury (not work related, see 8 below)<br>6. Surgery<br>7. Sleep problems<br>8. Occupation/work related condition/injury<br>9. Chronic pain in your joints / arthritis (joints, back, neck)<br>10. Diabetes or related complications	11. Problems with your heart, including unexplained pain in chest<br>12. Problems with your mouth, teeth or swallowing<br>13. Problems with your breathing<br>14. High blood pressure / hypertension<br>15. Stroke / sudden paralysis of one side of body<br>16. Generalised pain (stomach, muscle or other non-specific pain)<br>17. Depression or anxiety<br>18. Cancer<br>97. Other, please specify	

For peer review only - http://bmjopen.bmj.com/site/about/guidelines.xhtml

[Name of Country]
[Name of Slum]

Q406	How did you get there?<br><br>[SELECT ALL THAT THE RESPONDENT MENTIONS]	1. Private vehicle<br>2. Public transportation<br>3. Taxicab<br>4. Ambulance or emergency vehicle<br>5. Bicycle<br>6. Walked<br>7. Boda boda, rickshaw, other private transport other than a vehicle<br>8. Don't know<br><br>[BANGLADESH: add option Rickshaw]	
Q406a	About how long did it take you to get there?	☐☐:☐☐ hours: minutes<br><br>98. Don't know	
Q406b	About how long did you wait between arrival and first consultation with a health professional?	☐☐:☐☐ hours: minutes<br><br>98. Don't know	
Q407	Who paid for this inpatient stay?<br><br>Anyone else?<br><br>[SELECT ALL RESPONSES.<br>PROBE TO SEE IF ANYONE ELSE PAID OR CONTRIBUTED TO PAYING FOR THE CARE?]	1. Respondent<br>2. Spouse / Partner<br>3. Son / Daughter<br>4. Other family member<br>5. Non-family member<br>6. Mandatory insurance scheme<br>7. Voluntary insurance scheme	
		8. Hospitalisation was free .... →	Q413
Q408	Thinking about you last inpatient stay, how much did you or your family / household members pay out-of-pocket for:<br><br>[WRITE "0" IF THE SERVICE WAS FREE – IF A PERSON DID NOT HAVE MEDICINES OR TESTS, ENTER 99990 FOR "NOT APPLICABLE, DID NOT HAVE."]<br><br>[USE LOCAL CURRENCY]	a. [Health care provider's] fees: ☐☐☐☐☐☐<br><br>b. Medicines: ☐☐☐☐☐☐<br><br>c. Tests: ☐☐☐☐☐☐<br><br>d. Transport: ☐☐☐☐☐☐<br><br>e. Other, ☐☐☐☐☐☐<br><br>please specify:	
Q409	About how much in total was paid out-of-pocket for this inpatient visit?<br><br>[USE LOCAL CURRENCY]	☐☐☐☐☐☐☐☐☐	

For peer review only

[Name of Country]
[Name of Slum]

Q410	Overall, how satisfied were you with the care you received during your last overnight stay?	1. Very satisfied<br>2. Satisfied<br>3. Neither satisfied nor dissatisfied<br>4. Dissatisfied<br>5. Very dissatisfied	

For your last overnight visit to a hospital or long-term care facility, how would you rate the following:		Very good	Good	Moderate	Bad	Very bad
Q411	… the amount of time you waited before being attended to?	1	2	3	4	5
Q412	… your experience of being treated respectfully?	1	2	3	4	5
Q413	… how clearly health care providers explained things to you?	1	2	3	4	5
Q414	… your experience of being involved in making decisions for your treatment?	1	2	3	4	5
Q415	… the way the health services ensured that you could talk privately to providers?	1	2	3	4	5
Q416	… the ease with which you could see a health care provider you were happy with?	1	2	3	4	5
Q417	… the cleanliness in the health facility?	1	2	3	4	5

Outpatient Care

Now I will shift away from questions about overnight stays – to questions about health care you received that did not include an overnight hospital stay. The following questions are about care you received at a hospital, health centre, clinic, pharmacy, or private office including traditional health care but where you did not stay overnight.

Q418	Over the last 12 months did you receive any health care NOT including an overnight stay?	1. Yes<br>2. No …………………………………………… →	 Q443
Q419	In total, how many times did you receive health care or consultation in the last 12 months?	☐☐ times	

Q420	What was the last (most recent) health care facility you visited in the last 12 months?<br><br>[READ OUT RESPONSES, SELECT ONE OPTION ONLY.]	1. Private doctor's office<br>2. Private clinic or health care facility<br>3. Private hospital<br>4. Public clinic or health care facility<br>5. Public hospital<br>6. Charity or church run clinic<br>7. Charity or church run hospital<br>8. Traditional Healer [USE LOCAL TERMS]<br>9. Pharmacy<br>97. Other, please specify:<br><br>[BANGLADESH: remove options 6 and 7, replace with "NGO clinic or health care facility and community health clinic"]	
Q420a	Please name the provider	Free text	

For peer review only - http://bmjopen.bmj.com/site/about/guidelines.xhtml

[Name of Country]
[Name of Slum]

Q420b	Which reason(s) best describes why you chose this health care provider?<br>[CHOOSE ALL THAT THE RESPONDENT INDICATES]	1. Nearness of the facility<br>2. Service providers are cordial<br>3. Good service available<br>4. Short waiting time<br>5. Qualified doctors available<br>6. Low fees/low treatment cost<br>7. Good waiting arrangement<br>8. Confidentiality is maintained<br>9. Do not know where else to go<br>10. Medicine is also available<br>11. Availability of diagnostic service<br>12. Recommendation from someone<br>97. Other (please vspecify)	
Q421	Which was the last (most recent) health care provider you visited?<br><br>[THE PERSON WHO PROVIDED THE CARE FOR THE MAIN REASON FOR THE VISIT]<br><br>[AFTER Q426 SUBSTITUTE THE TYPE OF HEALTH CARE PROVIDER SELECTED BY THE PATIENT WHEN YOU SEE [HEALTH CARE PROVIDER] IN BRACKETS.]	1. Medical doctor (including surgeon, gynecologist, psychiatrist, ophthalmologist…)<br>2. Nurse<br>3. Midwife<br>4. Dentist<br>5. Physiotherapist or chiropractor<br>6. Traditional medicine practitioner [USE LOCAL NAME]<br>7. Pharmacist, druggist<br>8. Don't know	
Q421a	What was the sex of the [HEALTH CARE PROVIDER]?	1. Male<br>2. Female	
Q421b	Was this visit to [HEALTH CARE PROVIDER] for a chronic (ongoing) condition, new condition, both, or routine check-up?	1. Chronic<br>2. New<br>3. Both<br>4. Routine check-up	
Q421c	Which reason best describes why you needed this visit?<br><br>[RESPONDENT CAN SELECT ONLY ONE MAIN REASON FOR VISIT.		
	1. Communicable disease (infections, malaria, tuberculosis, HIV)<br>2. Maternal and perinatal conditions (pregnancy)<br>3. Nutritional deficiencies<br>4. Acute conditions (diarrhoea, fever, flu, headaches, cough, other)<br>5. Injury (not work related, see 8 below)<br>6. Surgery<br>7. Sleep problems<br>8. Occupation/work related condition/injury<br>9. Chronic pain in your joints / arthritis (joints, back, neck)<br>10. Diabetes or related complications	11. Problems with your heart, including unexplained pain in chest<br>12. Problems with your mouth, teeth or swallowing<br>13. Problems with your breathing<br>14. High blood pressure / hypertension<br>15. Stroke / sudden paralysis of one side of body<br>16. Generalised pain (stomach, muscle or other non-specific pain)<br>17. Depression or anxiety<br>18. Cancer<br>97. Other, please specify	

For peer review only - http://bmjopen.bmj.com/site/about/guidelines.xhtml

[Name of Country]
[Name of Slum]

Q422	Thinking about your <u>last visit</u>, how did you get there?<br><br>[CIRCLE ALL THAT THE RESPONDENT MENTIONS.]	1. Private vehicle<br>2. Public transportation<br>3. Taxicab<br>4. Ambulance or emergency vehicle<br>5. Bicycle<br>6. Walked<br>8. Don't know<br><br>[BANGLADESH: add option Rickshaw]	
Q423	About how long did it take you to get there?	☐☐:☐☐ hours: minutes<br><br>98. Don't know	
Q423a	About how long did you wait between arrival and first consultation with a health professional?	☐☐:☐☐ hours: minutes<br><br>98. Don't know	
Q424	Who paid for this most recent visit?<br><br>Anyone else?<br><br>[SELECT ALL RESPONSES.<br>PROBE TO SEE IF ANYONE ELSE PAID OR CONTRIBUTED TO PAYING FOR THE CARE?]	1. Respondent<br>2. Spouse / Partner<br>3. Son / Daughter<br>4. Other family member<br>5. Non-family member<br>6. Mandatory insurance scheme<br>7. Voluntary insurance scheme	
		8. It was free ……………………………… →	Q431
Q425	Thinking about your last <u>visit</u>, how much did you or your family / household members pay for:<br><br>[WRITE "0" IF THE SERVICE WAS FREE – IF A PERSON DID NOT HAVE MEDICINES OR TESTS, ENTER 99998 FOR "NOT APPLICABLE, DID NOT HAVE."]<br><br>[USE LOCAL CURRENCY]	a. [HEALTH CARE PROVIDER'S] fees:<br>☐☐☐☐☐☐<br><br>b. Medicines:<br>☐☐☐☐☐☐<br><br>c. Tests:<br>☐☐☐☐☐☐<br><br>d. Transport:<br>☐☐☐☐☐☐<br><br>e. Other,<br>☐☐☐☐☐☐<br><br>please specify:	
		f. Total costs:<br>☐☐☐☐☐☐☐☐☐	

For peer review only - http://bmjopen.bmj.com/site/about/guidelines.xhtml

[Name of Country]
[Name of Slum]

Q426	Overall, how satisfied were you with the care you received during your last visit?	1. Very satisfied<br>2. Satisfied<br>3. Neither satisfied nor dissatisfied<br>4. Dissatisfied<br>5. Very dissatisfied	

For your last visit to a health care provider, how would you rate the following:		Very good	Good	Moderate	Bad	Very bad
Q427	… the amount of time you waited before being attended to?	1	2	3	4	5
Q428	… your experience of being treated respectfully?	1	2	3	4	5

For your last visit to a health care provider, how would you rate the following:		*Very good*	*Good*	*Moderate*	*Bad*	*Very bad*
Q429	… how clearly health care providers explained things to you?	1	2	3	4	5
Q430	… your experience of being involved in making decisions for your treatment?	1	2	3	4	5
Q431	… the way the health services ensured that you could talk privately to providers?	1	2	3	4	5
Q432	… the ease with which you could see a health care provider you were happy with?	1	2	3	4	5
Q433	… the cleanliness in the health facility?	1	2	3	4	5

Care at home

Now I would like to ask you to think about care you received from a health care worker or provider while at home, place of work or at an education centre. For the following questions please think about health care accessed in the past year only.

Q434	Over the last 12 months, have you received a visit in your home or place of work or education from any health care worker or provider?	1. Yes<br>2. No……………………………………. →<br>8. Don't know……………………….→	 Q444<br>Q444
Q434a	What was discussed with the health care worker?<br><br>[SELECT ALL THAT APPLY]	1. Family planning<br>2. Healthy life-style<br>3. Advice for common illness<br>4. Vaccines for pregnant women and children<br>5. Diet and nutrition<br>6. Other (specify _____________)<br>8. Don't know	
Q434b	What, if any, materials were distributed by the health care worker?	1. Family planning materials<br>2. De-worming tablets<br>3. Medicine for common cold or fever<br>4. Vitamin(s)<br>5. Calcium<br>6. Iron tablet(s)<br>7. Other (specify _____________)	

For peer review only - http://bmjopen.bmj.com/site/about/guidelines.xhtml

[Name of Country]
[Name of Slum]

		8. Don't know	

M-health

Q435	In the last 12 months have you used or attempted to use your mobile phone or other digital communication device (e.g.: laptop, tablet) to access health information, advice or care for yourself, where information about your health was received or given?	1. Yes<br>2. No……………………………………… →	Q472
Q436	Which ways of accessing health advice or information have you used?<br><br>[SELECT ALL THAT APPLY]	1. Contacted someone (via text message, chat website, e-mail, video call etc.)…………………..-><br>2. Researched health information without speaking to someone (via Google, health website etc.)……………………………..->	
Q437	Have you had any problems when using your mobile phone or other device for this purpose?	1. Yes<br>2. No ……………………………………… →	Q448
Q437a	What were the problem(s):<br><br>[SELECT ALL THAT THE RESPONDENT INDICATES]	1. No airtime/data/wifi<br>2. Poor connectivity/signal<br>3. Device not sufficiently charged<br>4. No response from the person/organisation contacted<br>5. Unable to read the textual message received<br>6. Unable to find the information needed on web/social media<br>7. Unable to read the relevant text found<br>97. Other	

Now I would like to ask you to think about the last time you used or attempted to use your mobile phone or other digital communication device to access health information, advice or care for yourself, where information about your health was received or given. For the following questions please think about the most recent time only.

Q438	Which ways of accessing health or health care information did you use?<br><br>[SELECT ALL THAT APPLY.]	1. Contacted someone (via text message, chat website, e-mail, video call etc.)…………………..-><br>2. Researched health information without speaking to someone (via Google, health website etc.)……………………………..->	Q450<br><br>Q451
Q439	Who did you receive this information or what service did you use to get this information?<br><br>[REMEMBER WE ARE ASKING NOW ABOUT YOUR LAST (MOST RECENT) USE OF A DIGITAL CHANNEL FOR SEEKING HEALTH INFORMATION, ADVICE OR CARE.]	1. Medical Doctor<br>2. Nurse<br>3. Midwife<br>4. Dentist<br>5. Physiotherapist or chiropractor<br>6. Traditional medicine practitioner<br>7. Pharmacist, druggist<br>8. Users and/or provider of health related app.	
Q440	Please provide name of person/organisation/website/social media site	[Free text]	

For peer review only - http://bmjopen.bmj.com/site/about/guidelines.xhtml

[Name of Country]
[Name of Slum]

	and phone number or web address/e-mail address		
Q441	Why did you choose to get health information this way?<br><br>[QUESTION REFERS TO WHY THE RESPONDENT CHOSE TO USE M-HEALTH RATHER THAN SEEING A PROVIDER IN PERSON]<br><br>[CHOOSE ALL THAT THE RESPONDENT INDICATES]	1. I could seek the health information, advice or care at a time I chose<br>2. I could seek the health information, advice or care in a place I chose<br>3. The problem was urgent<br>4. The problem was not urgent<br>5. Service providers are cordial<br>6. I would not have to wait at a facility<br>7. I trust the provider<br>8. I needed more information than I usually receive from a health worker<br>9. I had no money to get to see a health worker<br>10. It was cheaper than going to see a health worker<br>11. I had more privacy during the communication than when seeing a health worker<br>12. I did not want other people to know I was seeking health, information advice or care<br>13. This was the only option that was available at that time	
Q442	Which reason best describes why you needed this contact?<br><br>[RESPONDENT CAN SELECT ONLY ONE MAIN REASON FOR VISIT.		
	1. Communicable disease (infections, malaria, tuberculosis, HIV)<br>2. Maternal and perinatal conditions (pregnancy)<br>3. Nutritional deficiencies<br>4. Acute conditions (diarrhoea, fever, flu, headaches, cough, other)<br>5. Injury (not work related, see 8 below)<br>6. Surgery<br>7. Sleep problems<br>8. Occupation/work related condition/injury<br>9. Chronic pain in your joints / arthritis (joints, back, neck)<br>10. Diabetes or related complications	11. Problems with your heart, including unexplained pain in chest<br>12. Problems with your mouth, teeth or swallowing<br>13. Problems with your breathing<br>14. High blood pressure / hypertension<br>15. Stroke / sudden paralysis of one side of body<br>16. Generalised pain (stomach, muscle or other non-specific pain)<br>17. Depression or anxiety<br>18. Cancer<br>97. Other, please specify	
Q443	Was there a cost associated with accessing this health advice or information?	1. Yes<br>2. No... →	Q458
Q444	Thinking about the last time you last accessed health advice or information, how much did you or your family / household members pay (Do not include cost of airtime/data):	[ ][ ][ ][ ]<br><br>8. Don't know	
Q445	Overall, how <u>satisfied</u> were you with the information, advice or care you received?	1. Very satisfied<br>2. Satisfied<br>3. Neither satisfied nor dissatisfied<br>4. Dissatisfied<br>5. Very dissatisfied	

9

For peer review only

[Name of Country]
[Name of Slum]

Q446	Did you seek information, advice or care about the same problem from a health worker face-to-face after this digital contact?	1. Yes<br>2. No	

Now I would like you to think about your most recent contact again. I want to know your impression of your most recent contact for health information, advice or care. I would like you to rate your experiences using the following questions:

For the last time you accessed health advice or information with a phone, computer, or other electronic device, , how would you rate the following:		Very good	Good	Moderate	Bad	Very bad
FOR RESPONDENTS WHO HAVE REPORTED A CONTACTING SOMEONE						
Q447	… your experience of being treated respectfully?	1	2	3	4	5
Q448	… how clearly health care providers explained things to you?	1	2	3	4	5
Q449	… the ease with which you could make contact with a health care provider you were happy with?	1	2	3	4	5
FOR RESPONDENTS WHO HAVE REPORTED RESEARCHING HEALTH INFO						
Q450	… how understandable was the information or advice?	1	2	3	4	5

Unpaid care

Now I would like to ask you to think about the last time you provided/receive care from someone for a long-term physical or mental illness or disability or for getting old and weak. For the following questions please think about care provided or received in the past year only.

Q451	During the past year, did you provide help to a relative or friend (adult or child), because this person has a long-term physical or mental illness or disability, or is getting old and weak?	1. Yes, for a person living in the same household<br>2. Yes, for a person living in a separate household<br>3. No…………………………………………. →	Q473
Q451a	Please tell me the kind of care provided:<br><br>[PLEASE SELECT ALL THAT APPLY]	1. You helped with personal care, such as going to the toilet, washing, getting dressed, or eating<br>2. You helped with medical care, like changing bandages, and giving medicines<br>3. You helped with household activities, such as meal preparation, shopping, cleaning, laundry<br>4. You watched over them since their behaviour can be upsetting or dangerous to themselves or others	
Q451b	Is/was this one-off or regular care?	1. One-off<br>2. Regular	
Q451c	Approximately how many hours a week do/did you spend providing this help?	☐☐☐☐ hours per week	

For peer review only - http://bmjopen.bmj.com/site/about/guidelines.xhtml

[Name of Country]
[Name of Slum]

Q452	During the past year, did you receive help from a relative or friend (adult or child) because you have a long-term physical or mental illness or disability or are getting old and weak?	1. Yes, from a person living in the same household<br>2. Yes, from a person living in a separate household<br>3. No………………………………………. →	Q474
Q452a	Please tell me the kind of care provided:<br><br>[PLEASE SELECT ALL THAT APPLY]	1. You were helped with personal care, such as going to the toilet, washing, getting dressed, or eating<br>2. You were helped with medical care, like changing bandages, and giving medicines<br>3. You were helped with household activities, such as meal preparation, shopping, cleaning, laundry<br>4. You were watched over since your behaviour can be upsetting or dangerous to yourself or others	
Q452b	Is/was this one-off or regular care?	1. One-off<br>2. Regular	
Q452c	Approximately how many hours a week do you receive this help?	☐☐☐☐ hours per week	

For peer review only

For peer review only - http://bmjopen.bmj.com/site/about/guidelines.xhtml

[Name of Country]
[Name of Slum]

Module 5: Health Status and Wellbeing

We will now as you a series of questions about your health and well-being. Please try to answer as honestly and accurately as possible. All responses will be kept in the strictest confidence.

Q501	In general would you say your health is:	1. Excellent<br>2. Very good<br>3. Good<br>4. Fair<br>5. Poor
Q502	Compared to one year ago, how would you rate your health in general now?	1. Much better than one year ago<br>2. Somewhat better now than one year ago<br>3. About the same<br>4. Somewhat worse now that one year ago<br>5. Much worse now than one year ago

Please select the response that best describes you

Q503	Physical functioning	1. Your health does not limit you in vigorous activities (such as running, lifting heavy objects)<br>2. Your health limits you a little in vigorous activities (such as running, lifting heavy objects)<br>3. Your health limits you a little in moderate activities (such as moving a table, playing with children)<br>4. Your health limits you a lot in moderate activities (such as moving a table, playing with children)<br>5. Your health limits you a little in bathing and dressing<br>6. Your health limits you a lot in bathing and dressing	
Q504	Role limitations	1. You have no problems with your work or other regular daily activities as a result of your physical health or emotional problems<br>2. You are limited in the kind of work or other activities as a result of your physical health<br>3. You accomplish less than you would like as a result of emotional problems<br>4. You are limited in the kind of work or other activities as a result of you physical health and accomplish less than you would like as a result of emotional problems	
Q505	Social functioning	Your health limits your social activities (like visiting friends, relatives, etc.):<br>1. none of the time<br>2. a little of the time<br>3. some of the time<br>4. most of the time<br>5. all of the time	
Q506	Pain	1. You have no pain<br>2. You have pain but it does not interfere with your normal work (both outside the home and housework)<br>3. You have pain that interferes with your normal work (both outside the home and housework) a little bit<br>4. You have pain that interferes with your normal work (both outside the home and housework) moderately<br>5. You have pain that interferes with your normal work (both outside the home and housework) quite a bit<br>6. You have pain that interferes with your normal work (both outside the home and housework) extremely	

For peer review only

For peer review only - http://bmjopen.bmj.com/site/about/guidelines.xhtml

[Name of Country]
[Name of Slum]

Q507	Mental health<br>You feel tense or downhearted and low:	1. None of the time<br>2. A little of the time<br>3. Some of the time<br>4. Most of the time<br>5. All of the time	
Q508	Vitality<br>You have a lot of energy:	1. None of the time<br>2. A little of the time<br>3. Some of the time<br>4. Most of the time<br>5. All of the time	

The following question asks how satisfied you feel, on a scale from 0 to 10. Zero means you feel 'not at all satisfied', and 10 means you feel 'completely satisfied'.

Q509	Overall, how satisfied are you with life as a whole these days? [0-10]	☐☐

The following question asks how worthwhile you feel the things you do in your life are, on a scale from 0 to 10. Zero means you feel the things you do in your life are 'not at all worthwhile', and 10 means 'completely worthwhile'.

Q510	Overall, to what extent do you feel the things you do in your life are worthwhile? [0-10]	☐☐

The following questions ask about how you felt yesterday on a scale from 0 to 10. Zero means you did not experience the feeling 'at all' yesterday, while 10 means you experienced the feeling 'all of the time' yesterday.

I will now read out a list of ways you might have felt yesterday.

Q511	How about happy? [0-10]	☐☐
Q512	How about worried? [0-10]	☐☐
Q513	How about depressed? [0-10]	☐☐

For peer review only - http://bmjopen.bmj.com/site/about/guidelines.xhtml

[Name of Country]
[Name of Slum]

Individual (child) Questionnaire

[THE PERSON COMPLETING THE QUESTIONNAIRE SHOULD BE THE RESPONSIBLE ADULT FOR THE RANDOMLY SELECTED CHILD]

[The child's name is referred to as {NAME}]

The next set of questions will ask you about the last time that you needed health care, whether or not you received that care. Please think back to the last time.

Module 4: Health Service Use

Q401	Has {NAME} needed health care in the last 12 months (whether or not {NAME} received health care)?<br><br>[INFORM THE RESPONDANT THAT THIS CAN BE INPATIENT CARE, OUTPATIENT CARE, MEDICATION, TRADITIONAL CARE, OR ANY OTHER INTERVENTION FOR THEIR HEALTH	1. Yes<br>2. No	Q402<br>Q443
Q402	The last time {NAME} needed health care, did {NAME} get health care?	1. Yes<br>2. No	
Q402b<br><br>[ONLY IF 402 = "NO"]	Which reason(s) best explains why {NAME} did not get health care?<br><br>[CHOOSE ALL THAT THE RESPONDENT INDICATES]	1. Could not afford the cost of the visit<br>2. No transport available<br>3. Could not afford the cost of transport<br>4. You or your child were previously badly treated<br>5. Could not take the time off work or had other commitments<br>6. The health care provider's drugs or equipment were inadequate<br>7. The health care provider's skills were inadequate<br>8. You did not know where to go<br>9. You tried but were denied health care<br>10. You thought you or your child were not sick enough<br>97. Other, please specify	

<u>Inpatient Hospital Care</u>

The next two questions ask about <u>any</u> overnight stay in a hospital or other health care facility {NAME} had in the last year.

For peer review only - http://bmjopen.bmj.com/site/about/guidelines.xhtml

[Name of Country]
[Name of Slum]

Q403	In the last 12 months, has {NAME} ever stayed overnight in a hospital, long-term care facility, or other provider?<br>[Select all that apply]	1. Yes, a hospital<br>2. Yes, long-term care facility<br>3. Yes, other provider (Please specify)	
		4. No …………………………………………… →	Q423

Q404	Over the last 12 months, how many different times was {NAME} a patient in a hospital / long-term care facility for at least one night?	☐☐ times<br>98. Don't know	
		IF "00" (NO OVERNIGHT STAYS) …………….. →	Q423

I would like you to ask about {NAME}'s last inpatient stay only.

Q405	What type of hospital or facility was it? Remember we are asking now about {NAME}'s last (most recent) overnight stay.<br><br>[ONE ANSWER ONLY]	1. Public hospital<br>2. Private hospital<br>3. Charity or church-run hospital<br>4. Old person's home or long-term care facility<br>97. Other, please specify<br><br>[BANGLADESH: remove option 3, replace with "NGO clinical or health care facility and community health clinic"]	
Q405a	Please name the facility	Free text	
Q405b	Which reason(s) best describes why you chose this health care provider?<br>[CHOOSE ALL THAT THE RESPONDENT INDICATES]	1. Nearness of the facility<br>2. Service providers are cordial<br>3. Good service available<br>4. Short waiting time<br>5. Qualified doctors available<br>6. Low fees/low treatment cost<br>7. Good waiting arrangement<br>8. Confidentiality is maintained<br>9. Do not know where else to go<br>10. Medicine is also available<br>11. Availability of diagnostic service<br>12. Recommendation from someone<br>97. Other (please specify)	
Q405c	Which reason best describes why {NAME} was last hospitalised?<br><br>[RESPONDENT CAN SELECT ONLY ONE MAIN REASON FOR VISIT. USE SHOWCARD IN APPENDIX.]		

For peer review only - http://bmjopen.bmj.com/site/about/guidelines.xhtml

[Name of Country]
[Name of Slum]

	1. Communicable disease (infections, malaria, tuberculosis, HIV)<br>2. Maternal and perinatal conditions (pregnancy)<br>3. Nutritional deficiencies<br>4. Acute conditions (diarrhoea, fever, flu, headaches, cough, other)<br>5. Injury (not work related, see 8 below)<br>6. Surgery<br>7. Sleep problems<br>8. Occupation/work related condition/injury<br>9. Chronic pain in your joints / arthritis (joints, back, neck)<br>10. Diabetes or related complications	11. Problems with your heart, including unexplained pain in chest<br>12. Problems with your mouth, teeth or swallowing<br>13. Problems with your breathing<br>14. High blood pressure / hypertension<br>15. Stroke / sudden paralysis of one side of body<br>16. Generalised pain (stomach, muscle or other non-specific pain)<br>17. Depression or anxiety<br>18. Cancer<br>97. Other, please specify	
Q406	How did you get there?<br><br>[CIRCLE ALL THAT THE RESPONDENT MENTIONS]	1. Private vehicle<br>2. Public transportation<br>3. Taxicab<br>4. Ambulance or emergency vehicle<br>5. Bicycle<br>6. Walked<br>7. Boda boda, rickshaw, other private transport other than a vehicle<br>8. Don't know<br><br>[BANGLADESH: add option Rickshaw]	
Q406a	About how long did it take you to get there?	☐☐:☐☐ hours: minutes<br><br>98. Don't know	
Q406b	About how long did you wait between arrival and first consultation with a health professional?	☐☐:☐☐ hours: minutes<br><br>98. Don't know	
Q407	Who paid for this inpatient stay?<br><br>Anyone else?<br><br>[CIRCLE ALL RESPONSES.<br>PROBE TO SEE IF ANYONE ELSE PAID OR CONTRIBUTED TO PAYING FOR THE CARE?]	1. Respondent<br>2. Spouse / Partner<br>3. Son / Daughter<br>4. Other family member<br>5. Non-family member<br>6. Mandatory insurance scheme<br>7. Voluntary insurance scheme	
		8. Hospitalisation was free .... →	Q413
Q408	Thinking about {NAME}'s last inpatient stay, how much did you or your family / household members <u>pay out-of-pocket</u> for:<br><br>[WRITE "0" IF THE SERVICE WAS FREE – IF A PERSON DID NOT HAVE MEDICINES OR TESTS, ENTER 99990 FOR "NOT APPLICABLE, DID NOT HAVE."]<br><br>[USE LOCAL CURRENCY]	a. [Health care provider's] fees:<br>☐☐☐☐☐☐<br><br>b. Medicines:<br>☐☐☐☐☐☐<br><br>c. Tests:<br>☐☐☐☐☐☐	

For peer review only - http://bmjopen.bmj.com/site/about/guidelines.xhtml

[Name of Country]
[Name of Slum]

		d. Transport: ☐☐☐☐☐☐<br><br>e. Other, ☐☐☐☐☐☐<br><br>please specify:	
Q409	About how much in total was paid out-of-pocket for this inpatient visit?<br><br>[USE LOCAL CURRENCY]	☐☐☐☐☐☐☐☐☐	
Q410	Overall, how satisfied were you with the care {NAME} received during his/her last [hospital] stay?	1. Very satisfied<br>2. Satisfied<br>3. Neither satisfied nor dissatisfied<br>4. Dissatisfied<br>5. Very dissatisfied	

For {NAME}'s last overnight visit to a hospital or long-term care facility, how would you rate the following:		Very good	Good	Moderate	Bad	Very bad
Q411	… the amount of time you waited before being attended to?	1	2	3	4	5
Q412	… your experience of being treated respectfully?	1	2	3	4	5
Q413	… how clearly health care providers explained things to you?	1	2	3	4	5
Q414	… your experience of being involved in making decisions for {NAME}'s treatment?	1	2	3	4	5
Q415	… the way the health services ensured that you could talk privately to providers?	1	2	3	4	5
Q416	… the ease with which you could see a health care provider you were happy with?	1	2	3	4	5
Q417	… the cleanliness in the health facility?	1	2	3	4	5

Outpatient Care

Now I will shift away from questions about overnight stays – to questions about health care {NAME} received that did not include an overnight hospital stay. The following questions are about care {NAME} received at a hospital, health centre, clinic, pharmacy, or private office including traditional health care but where {NAME} did not stay overnight.

Q418	Over the last 12 months did {NAME} receive any health care NOT including an overnight stay?	1. Yes<br>2. No …………………………………………… →	 Q443
Q419	In total, how many times did {NAME} receive health care or consultation in the last 12 months?	☐☐ times	

Now I would like you to think about the most recent visit – and ask you specifically about {NAME}'s last or most recent visit:

For peer review only - http://bmjopen.bmj.com/site/about/guidelines.xhtml

[Name of Country]
[Name of Slum]

Q420	What was the last (most recent) health care facility {NAME} visited in the last 12 months?<br><br>[READ OUT RESPONSES, CHOOSE ONE OPTION ONLY.]	1. Private doctor's office<br>2. Private clinic or health care facility<br>3. Private hospital<br>4. Public clinic or health care facility<br>5. Public hospital<br>6. Charity or church run clinic<br>7. Charity or church run hospital<br>8. Traditional Healer [USE LOCAL TERMS]<br>9. Pharmacy<br>97. Other, please specify:<br><br>[BANGLADESH: remove options 6 and 7, replace with "NGO clinic or health care facility and community health clinic"]	
Q420a	Please name the provider	Free text	
Q420b	Which reason(s) best describes why you chose this health care provider?<br>[CHOOSE ALL THAT THE RESPONDENT INDICATES]	1. Nearness of the facility<br>2. Service providers are cordial<br>3. Good service available<br>4. Short waiting time<br>5. Qualified doctors available<br>6. Low fees/low treatment cost<br>7. Good waiting arrangement<br>8. Confidentiality is maintained<br>9. Do not know where else to go<br>10. Medicine is also available<br>11. Availability of diagnostic service<br>12. Recommendation from someone<br>97. Other (please specify)	
Q421	Which was the last (most recent) health care provider {NAME} visited?<br><br>[THE PERSON WHO PROVIDED THE CARE FOR THE MAIN REASON FOR THE VISIT]<br><br>[AFTER Q426 SUBSTITUTE THE TYPE OF HEALTH CARE PROVIDER SELECTED BY THE PATIENT WHEN YOU SEE [HEALTH CARE PROVIDER] IN BRACKETS.]	1. Medical doctor (including surgeon, gynecologist, psychiatrist, ophthalmologist…)<br>2. Nurse / midwife<br>3. Dentist<br>4. Physiotherapist or chiropractor<br>5. Traditional medicine practitioner [USE LOCAL NAME]<br>6. Pharmacist, druggist<br>8. Don't know	
Q421a	What was the sex of the [HEALTH CARE PROVIDER]?	1. Male<br>2. Female	
Q421b	Was this visit to [HEALTH CARE PROVIDER] for a chronic (ongoing) condition, new condition, both, or routine check-up?	1. Chronic<br>2. New<br>3. Both<br>4. Routine check-up	
Q421c	Which reason best describes why {NAME} needed this visit?<br><br>[RESPONDENT CAN SELECT ONLY ONE MAIN REASON FOR VISIT.		

For peer review only - http://bmjopen.bmj.com/site/about/guidelines.xhtml

[Name of Country]
[Name of Slum]

	1. Communicable disease (infections, malaria, tuberculosis, HIV)<br>2. Maternal and perinatal conditions (pregnancy)<br>3. Nutritional deficiencies<br>4. Acute conditions (diarrhoea, fever, flu, headaches, cough, other)<br>5. Injury (not work related, see 8 below)<br>6. Surgery<br>7. Sleep problems<br>8. Occupation/work related condition/injury<br>9. Chronic pain in your joints / arthritis (joints, back, neck)<br>10. Diabetes or related complications	11. Problems with your heart, including unexplained pain in chest<br>12. Problems with your mouth, teeth or swallowing<br>13. Problems with your breathing<br>14. High blood pressure / hypertension<br>15. Stroke / sudden paralysis of one side of body<br>16. Generalised pain (stomach, muscle or other non-specific pain)<br>17. Depression or anxiety<br>18. Cancer<br>97. Other, please specify	
Q422	Thinking about {NAME}'s last visit, how did you get there?<br><br>[CIRCLE ALL THAT THE RESPONDENT MENTIONS.]	1. Private vehicle<br>2. Public transportation<br>3. Taxicab<br>4. Ambulance or emergency vehicle<br>5. Bicycle<br>6. Walked<br>8. Don't know<br><br>[BANGLADESH: add option Rickshaw]	
Q423	About how long did it take you to get there?	☐☐:☐☐ hours: minutes<br><br>98. Don't know	
Q423a	About how long did you wait between arrival and first consultation with a health professional?	☐☐:☐☐ hours: minutes<br><br>98. Don't know	
Q424	Who paid for this most recent visit?<br><br>Anyone else?<br><br>[SELECT ALL RESPONSES.<br>PROBE TO SEE IF ANYONE ELSE PAID OR CONTRIBUTED TO PAYING FOR THE CARE?]	1. Respondent<br>2. Spouse / Partner<br>3. Son / Daughter<br>4. Other family member<br>5. Non-family member<br>6. Mandatory insurance scheme<br>7. Voluntary insurance scheme	
		8. It was free .... →	Q431

6

[Name of Country]
[Name of Slum]

Q425	Thinking about {NAME}'s last <u>visit</u>, how much did you or your family / household members pay for:<br><br>[WRITE "0" IF THE SERVICE WAS FREE – IF A PERSON DID NOT HAVE MEDICINES OR TESTS, ENTER 99998 FOR "NOT APPLICABLE, DID NOT HAVE."]<br><br>[USE LOCAL CURRENCY]	a. [HEALTH CARE PROVIDER'S] fees:<br>b. Medicines:<br>c. Tests:<br>d. Transport:<br>e. Other,<br>please specify:<br>f. Total costs:	
Q426	Overall, how <u>satisfied</u> were you with the care {NAME} received during your last visit?	1. Very satisfied<br>2. Satisfied<br>3. Neither satisfied nor dissatisfied<br>4. Dissatisfied<br>5. Very dissatisfied	

For peer review only

I want to know your impression of your most recent visit for health care. I would like you to rate your experiences using the following questions:

For {NAME}'s <u>last visit</u> to a <u>health care provider</u>, how would you rate the following:		Very good	Good	Moderate	Bad	Very bad
Q427	... the amount of time you <u>waited</u> before being attended to?	1	2	3	4	5
Q428	... your experience of <u>being treated respectfully</u>?	1	2	3	4	5

For {NAME}'s <u>last visit</u> to a <u>health care provider</u>, how would you rate the following:		*Very good*	*Good*	*Moderate*	*Bad*	*Very bad*
Q429	... how <u>clearly</u> health care providers explained things to you?	1	2	3	4	5
Q430	... your experience of being <u>involved in making decisions</u> for {NAME}'s treatment?	1	2	3	4	5
Q431	... the way the health services ensured that you could <u>talk privately</u> to providers?	1	2	3	4	5
Q432	... the <u>ease</u> with which you could see a health care provider you were happy with?	1	2	3	4	5
Q433	... the <u>cleanliness</u> in the health facility?	1	2	3	4	5

For peer review only - http://bmjopen.bmj.com/site/about/guidelines.xhtml

[Name of Country]
[Name of Slum]

Q501	In general would you say {NAME}'s health is:	1. Excellent<br>2. Very good<br>3. Good<br>4. Fair<br>5. Poor
Q502	Compared to one year ago, how would you rate {NAME}'s health in general now?	1. Much better than one year ago<br>2. Somewhat better now than one year ago<br>3. About the same<br>4. Somewhat worse now that one year ago<br>5. Much worse now than one year ago

For peer review only

[Name of Country]
[Name of Slum]

Household Questionnaire

[THE PERSON COMPLETING THE HOUSEHOLD QUESTIONNAIRE SHOULD BE THE HEAD OF HOUSEHOLD IF AVAILABLE. WHERE UNAVAILABLE, ANY SPOUSE OF THE HEAD OF HOUSEHOLD SHOULD BE SELECTED TO COMPLETE THE HOUSEHOLD QUESTIONNAIRE. IN THE CASE THAT NEITHER THE HEAD OF HOUSEHOLD NOR ANY SPOUSE IS AVAILABLE, PLEASE COMPLETE THE HOUSEHOLD QUESTIONNAIRE WITH ANY OTHER AVAILABLE ADULT MEMBER OF THE HOUSEHOLD, AGED 18+]

Module 1: Household Schedule

Line no.	Usual residents and visitors	Respondent	Relationship to head of household	Sex	Residence		Age	Marital status
1	Q101A	Q101B	Q101C	Q101D	Q102A	Q102B	Q103	Q104
	Please give me the names of the persons who usually live in your household and guests of the household who stayed here last night, starting with the respondent.<br><br>[WHEN THE RESPONDENT RESPONDS FOR THEMSELF, REFER TO 'YOU' OR 'YOUR']<br><br>[AFTER LISTING THE NAMES AND RECORDING THE RELATIONSHIP AND SEX FOR EACH PERSON (Q101A-C), ASK QUESTIONS **102A-C** TO BE SURE THAT THE LISTING IS COMPLETE. THEN ASK APPROPRIATE QUESTIONS 103-111 FOR EACH PERSON.]	Is this the respondent?<br>1 Yes<br>2 No	What is the relationship of [NAME] to the head of the household?<br><br>01 head; 02 spouse; 03 son or daughter; 04 son-in-law or daughter-in-law; 05 grandchild; 06 parent; 07 parent-in-law; 08 sibling; 09 other relative; 10 adopted/ foster/ stepchild; 11 not related; 98 don't know	Is [NAME] male or female?<br><br>01 Male; 02 Female	Does [NAME] usually live here?<br><br>01 Yes; 02 No	Did [NAME] sleep here last night?<br><br>01 Yes; 02 No	How old is [NAME]?<br><br>[IN YEARS; ASK TO SEE ID IF RESPONDENT UNSURE OF OWN AGE]<br><br>999 = Don't know	[IF AGED 16 OR OLDER]<br><br>What is [NAME]'s current marital status:<br>01 married or living together; 02 divorced / separated; 03 widowed; 04 Never-married and never lived together
01								
02								
03								
04								
05								
06								
07								
08								
09								
10								

For peer review only
For peer review only - http://bmjopen.bmj.com/site/about/guidelines.xhtml

[Name of Country]
[Name of Slum]

Line no.	Ever attended school		Current work status	Migration status				Health status
1	Q105A	Q105B	Q106	Q107A	Q107B	Q107C	Q107D	Q108
	[IF AGED 6 OR OLDER]<br><br>Has [NAME] ever attended school?<br><br>01 Yes;<br>02 No<br>08 Don't know [IF "NO" OR "DON'T KNOW" GO TO Q107]	[IF AGED 6 OR OLDER]<br><br>What is the highest level of school [NAME] has attended?<br><br>What is the highest grade [NAME] completed at that level?	Is [NAME] currently working?<br><br>01 Yes; 02 No<br><br>[DEFINE 'CURRENTLY WORKING' AS HAVING WORKED IN THE LAST WEEK IF UNSURE]	Has [NAME] always lived in this area/neighbourhood?<br><br>01 Yes; 02 No; 08 Don't know<br><br>[DEFINE AS HAVING BEEN BORN HERE IF UNSURE]<br><br>[IF "YES" GO TO Q110, IF "NO" CONTINUE WITH Q109B-D]	How long has [NAME] been living (continuously) in this area?<br><br>[□□ YEARS □□ MONTHS]	Where was [NAME] living before?<br><br>01 in the same area/ neighbourhood; 02 In another "slum" area in this country; 03 In "non-slum" urban area in this country; 04 In a rural area in this country; 05 Outside the country.<br><br>SENSITIVE TRANSLATION REQUIRED	Where has [NAME] lived for most of their life<br><br>01 in the same area/ neighbourhood; 02 In another "slum" area in this country; 03 In "non-slum" urban area in this country; 04 In a rural area in this country; 05 Outside the country.<br><br>SENSITIVE TRANSLATION REQUIRED	Does [NAME] have any physical or mental health conditions or illness lasting or expected to last for 12 months or more?<br><br>01 Yes;<br>02 No
01					□□ □□			
02					□□ □□			
03					□□ □□			
04					□□ □□			
05					□□ □□			
06					□□ □□			
07					□□ □□			
08					□□ □□			
09					□□ □□			
10					□□ □□			

For peer review only - http://bmjopen.bmj.com/site/about/guidelines.xhtml

[Name of Country]
[Name of Slum]

Health insurance status					Digital communication			
Q109A	Q109B	Q109C	Q109D	Q109E	Q110A	Q110B	Q110C	Q110D
Is [NAME] covered by any kind of insurance plan?<br><br>[IF “NO” Q111A, IF “YES” continue with Q110B-E]	What type of health insurance is (NAME) covered by?<br><br>[SELECT ALL THAT RESPONDENT INDICATES]<br><br>Mutual health organisation/ community-based health insurance;<br>Health insurance through employer;<br>Social security;<br>Other privately purchased health insurance;<br>Other, please specify	How much does your household pay for [NAME]’s health insurance each year?	Is [NAME] covered by insurance only because of his/her relationship to someone else who has health insurance?	Who is enrolled in the insurance plan that gives [NAME] health insurance?	Does [NAME] carry a mobile phone day-to-day?<br><br>01 Yes; 02 No; 08 Don’t know	Is [NAME] able to use a computer, tablet or other form of digital communication other than a mobile phone, day-to-day?<br><br>01 Yes; 02 No; 08 Don’t know	How do they usually access use of a mobile phone?<br><br>1. They have no access<br>2. They ask another household member to communicate for them<br>3. They borrow a phone from another member of the household when they need to use one	How do they usually access the internet?<br><br>1. They have no access<br>2. They ask another member of the household to communicate for them<br>3. They borrow a device from another member of the household when they need to use one

Q112A. Just to make sure that I have a complete listing: are there any other people such as small children or infants that we have not listed?

Q112B. Are there any other people who may not be members of your family, such as domestic servants, lodgers or friends who usually live here?

Q112C. Are there any guests or temporary visitors staying here, or anyone else who stayed here last night, who have not been listed?

For peer review only

For peer review only - http://bmjopen.bmj.com/site/about/guidelines.xhtml

[Name of Country]
[Name of Slum]

Module 2: Household characteristics

IN-COUNTRY TEAMS TO DELETE AS APPROPRIATE

I would like to ask you some questions about your dwelling or home. [BANGLADESH VERSION]

Q201	What is the main source of drinking water for members of your household?	1. Piped into dwelling……………………………→<br>2. Piped to yard/plot……………………………→<br>3. Public tap/standpipe<br>4. Tube well or Borehole<br>5. Dugwell: Protected well<br>6. Dugwell: Unprotected well<br>7. Protected spring<br>8. Unprotected spring<br>9. Rainwater…………………………………………→<br>10. Tanker truck<br>11. Cart with small tank<br>12. Surface water (river/dam/lake/pond/stream/canal/irrigation channel)<br>13. Bottled water<br>97. Other	Q206<br>Q206<br><br><br><br><br><br><br>Q206
Q202	Where is that water source located?	1. In own dwelling………………………………………→<br>2. In own yard/plot……………………………………→<br>3. Elsewhere	Q206<br>Q206
Q203	How long does it take to go there, get water, and come back?	[ ][ ][ ] Minutes<br>998. Don't know	
Q203A	Who usually goes to this source to fetch the water for your household	1. Adult woman<br>2. Adult man<br>3. Female child<br>4. Male child<br>5. No one person usually fetches the water	
Q204	Do you share this source with other households?	1. Yes<br>2. No…………………………………………………………→	 Q206
Q205	How many households use this source of water?	No. of households if less than 10: [0][ ]<br><br>95. 10 of more households<br>98. Don't know	
Q206	Do you do anything to the water to make it safer to drink?	1. Yes<br>2. No…………………………………………………………→<br>8. Don't know…………………………………………→	 Q207<br>Q207
Q206A	What do you usually do to make the water safer to drink?<br><br>Anything else?<br><br>[RECORD ALL MENTIONED]	1. Boil<br>2. Add bleach/chlorine<br>3. Strain through a cloth<br>4. Use water filter (ceramic/sand/composite/etc.)<br>5. Solar disinfection<br>6. Let it stand and Settle<br>97. Other	

For peer review only - http://bmjopen.bmj.com/site/about/guidelines.xhtml

[Name of Country]
[Name of Slum]

Q207	What kind of toilet facility do members of your household usually use?<br><br>[IF RESPONDENT INDICATES 'FLUSH' OR 'POUR FLUSH', THEN PROBE WITH:<br><br>WHERE DOES IT FLUSH TO?]	1. Flush/pour flush to piped sewer system<br>2. Flush/pour flush to septic tank<br>3. Flush/pour flush to pit latrine<br>4. Flush/pour flush to somewhere else<br>5. Flush/pour flush don't know where<br>6. Ventilated improved pit latrine<br>7. Pit latrine with slab<br>8. Pit latrine without slab/Open pit<br>9. Composting toilet<br>10. Bucket toilet<br>11. Hanging toilet/hanging latrine<br>12. No facility/bush/field……………………..→<br>97. Other	Q210
Q208	Do you share this toilet facility with other households?	1. Yes<br>2. No……………………………………………….→	Q210
Q209	How many households use this toilet facility	No of household if less than 10 [0][ ]<br>95. 10 or more households<br>98. Don't know	
Q210	Does your household have:<br>[RECORD ALL THAT APPLY]	1. Electricity<br>2. Solar Electricity<br>3. Radio<br>4. Television<br>5. Mobile telephone<br>6. Non-mobile telephone<br>7. Refrigerator<br>8. Almirah/wardrobe<br>9. Electric Fan<br>10. Blu-ray/DVD/VCD Player<br>11. Water pump<br>12. IPS/Generator<br>13. Air conditioner<br>14. Computer Laptop	
Q210A	Does this household receive a cash transfer or any social assistance from the government, NGO's, CBO's, Churches or Other organizations?<br><br>[DEFINE AS HAVE RECEIVED IN THE LAST MONTH IF UNSURE]	1. Yes<br>1. No……………………………………………….→	Q211
Q210B	For what reason does the household receive a cash transfer or social assistance?<br><br>Any other reason?<br><br>[RECORD ALL MENTIONED]	1. Orphaned children 18 years or younger<br>2. Elderly person<br>3. Person with severe disability<br>4. Urban food subsidy<br>5. Food aid for person in arid and semi-arid lands<br>6. Health voucher<br>7. Food/cash for work<br>8. School feeding<br>9. Hunger safety net programme<br>2. Other	

For peer review only - http://bmjopen.bmj.com/site/about/guidelines.xhtml

[Name of Country]
[Name of Slum]

Q211	What type of fuel does your household mainly use for cooking?	3. Electricity<br>4. LPG<br>5. Natural Gas<br>6. Biogas<br>7. Kerosene<br>8. Coal, lignite<br>9. Charcoal<br>10. Wood<br>11. Straw/shrubs/grass<br>12. Agricultural crop<br>13. Animal dung<br>14. No food cooked in household……………….→<br>97. Other	 <br><br><br><br><br><br><br><br><br><br>Q215
Q212	Where is cooking usually done?	1. In a room used for living or sleeping<br>2. In a separate room used as kitchen<br>3. In a separate building used as kitchen<br>4. Outdoor<br>7. Other, please specify:	
Q213	[MAIN MATERIAL OF THE FLOOR- RECORD OBSERVATION]	1. Natural floor (earth/sand)<br>2. Rudimentary floor: wood planks<br>3. Rudimentary floor: palm/bamboo<br>4. Finished floor: Parquet or polished wood<br>5. Finished floor: ceramic tiles<br>6. Finished floor: Cement<br>7. Finished floor: Carpet<br>97. Other	
Q214	[MAIN MATERIAL OF THE ROOF- RECORD OBSERVATION]	1. Natural roof: no roof<br>2. Natural roof: thatch/palm leaf<br>3. Rudimentary roof: palm/bamboo<br>4. Rudimentary roof: wood planks<br>5. Rudimentary roof: cardboard<br>6. Finished roof: tin<br>7. Finished roof: wood<br>8. Finished roof: ceramic tiles<br>9. Finished roof: cement<br>10. Finished roof: roofing shingles<br>97. Other	
Q215	[MAIN MATERIAL OF THE EXTERIOR WALLS- RECORD OBSERVATION]	1. Natural walls: no walls<br>2. Natural walls: cane/palm/trunks<br>3. Natural walls: dirt<br>4. Rudimentary walls: bamboo with mud<br>5. Rudimentary walls: stone with mud<br>6. Rudimentary walls: plywood<br>7. Rudimentary walls: cardboard<br>8. Finished walls: tin<br>9. Finished walls: cement<br>10. Finished walls: stone with lime/cement<br>11. Finished walls: brick<br>12. Finished walls: woodplanks/shingles<br>97. Other	
Q216	How many rooms in this household are used for sleeping?	☐☐ Rooms	

[Name of Country]
[Name of Slum]

Q217	Does any member of this household own:	1. A car/truck/microbus<br>2. An autobike/tempo/CNG<br>3. A rickshaw/van<br>4. A bicycle<br>5. A motorcycle or motor scooter	
Q217A	Does your household own this structure (house, flat, shack)?	1. Yes<br>2. No	Q217B<br>Q217C
Q217B	Does your household own the land on which the structure (house, flat, shack) sits?	1. Yes<br>2. No	
Q217C	Do you pay rent?	1. Pays rent/lease<br>2. No rent with consent of owner<br>3. No rent: squatting	
Q218	Does your household own any homestead?	4. Yes<br>5. No	
Q218AA	Does your household own any land (other than the homestead land)?	1. Yes<br>2. No…………………………………………. →	Q222
Q219	Does this household own any livestock, herds, other farm animals or poultry?	1. Yes<br>2. No………………………………………….. →	Q224
Q220	How many of the following animals does this household own?<br><br>Buffaloes?<br>Cows?<br>Goats or sheep?<br>Chickens or ducks?<br>Other farm animals?		
Q221	Does any member of this household have a bank account?	1. Yes<br>2. No	
Q222	In the past 7 days were there days when your household did not have enough food or money to buy food?	1. Yes<br>2. No	
Q223	How many days in the month do you have air time (for calls and SMS) for at least one mobile phone in the household	1. Everyday<br>2. Almost every day (over 21 days a month but not every day)<br>3. More than half the days (over 14 but not as many as 21)<br>4. More than 7 days but less than 14<br>5. Less than 7 days<br>6. Never	
Q224	How many days in the month does someone in the household have data or access to wifi (for accessing internet for searching the web, using social media or using Email) for at least one of your digital communication devices in the household (smart phone, laptop, tablet)?	1. Everyday<br>2. Almost every day (over 21 days a month but not every day)<br>3. More than half the days (over 14 but not as many as 21)<br>4. More than 7 days but less than 14<br>Less than 7 days<br>5. Never	

[Name of Country]
[Name of Slum]

I would like to ask you some questions about your dwelling or home. KENYA VERSION

Q201	What is the main source of drinking water for members of your household?	1. Piped into dwelling……………………→<br>2. Piped to yard/plot……………………→<br>3. Public tap/standpipe<br>4. Tube well or Borehole<br>5. Dugwell: Protected well<br>6. Dugwell: Unprotected well<br>7. Protected spring<br>8. Unprotected spring<br>9. Rainwater……………………………→<br>10. Tanker truck<br>11. Cart with small tank<br>12. Surface water (river/dam/lake/pond/stream/ canal/irrigation channel)<br>13. Bottled water<br>97. Other	Q206<br>Q206<br><br><br><br><br><br><br>Q206
Q202	Where is that water source located?	1. In own dwelling……………………………→<br>2. In own yard/plot……………………………→<br>3. Elsewhere	Q206<br>Q206
Q203	How long does it take to go there, get water, and come back?	[ ][ ][ ] Minutes<br>998. Don't know	
Q203A	Who usually goes to this source to fetch the water for your household	1. Adult woman<br>2. Adult man<br>3. Female child<br>4. Male child<br>5. No one person usually fetches the water	
Q204	Do you share this source with other households?	1. Yes<br>2. No……………………………………………→	 Q206
Q205	How many households use this source of water?	No. of households if less than 10: [0][ ]<br><br>95. 10 of more households<br>98. Don't know	
Q206	Do you do anything to the water to make it safer to drink?	1. Yes<br>2. No…………………………………………………→<br>8. Don't know……………………………………………→	 Q207<br>Q207
Q206A	What do you usually do to make the water safer to drink?<br><br>Anything else?<br><br>[RECORD ALL MENTIONED]	1. Boil<br>2. Add bleach/chlorine<br>3. Strain through a cloth<br>4. Use water filter (ceramic/sand/composite/etc.)<br>5. Solar disinfection<br>6. Let it stand and Settle<br>7. Cover the water container<br>97. Other	
Q207	What kind of toilet facility do members of your household usually use?	1. Flush/pour flush to piped sewer system<br>2. Flush/pour flush to septic tank<br>3. Flush/pour flush to pit latrine	

[Name of Country]
[Name of Slum]

	[IF RESPONDENT INDICATES 'FLUSH' OR 'POUR FLUSH', THEN PROBE WITH: WHERE DOES IT FLUSH TO?]	4. Flush/pour flush to somewhere else<br>5. Flush/pour flush don't know where<br>6. Ventilated improved pit latrine<br>7. Pit latrine with slab<br>8. Pit latrine without slab/Open pit<br>9. Composting toilet<br>10. Bucket toilet<br>11. Hanging toilet/hanging latrine<br>12. No facility/bush/field……………………..→<br>97. Other	Q210
Q208	Do you share this toilet facility with other households?	1. Yes<br>2. No………………………………………………→	Q210
Q209	How many households use this toilet facility	No of household if less than 10 [0][ ]<br>95. 10 or more households<br>98. Don't know	
Q210	Does your household have:<br>[RECORD ALL THAT APPLY]	1. Electricity<br>2. Radio<br>3. Television<br>4. Mobile telephone<br>5. Non-mobile telephone<br>6. Refrigerator<br>7. Solar panel<br>8. Table<br>9. Chair<br>10. Sofa<br>11. Bed<br>12. Cupboard<br>13. Clock<br>14. Microwave oven<br>15. Blu-ray/VCD/DVD Player<br>16. Cassette/CD Player	
Q210A	Does this household receive a cash transfer or any social assistance from the government, NGOs, CBOs, Churches or other organisations?<br><br>[DEFINE AS HAVE RECEIVED IN THE LAST MONTH IF UNSURE]	1. Yes<br>2. No………………………………………………→	Q211
Q210B	For what reason does the household receive a cash transfer or social assistance?<br><br>Any other reason?<br><br>[RECORD ALL MENTIONED]	1. Orphaned children 18 years or younger<br>2. Elderly person<br>3. Person with severe disability<br>4. Urban food subsidy<br>5. Food aid for person in arid and semi-arid lands<br>6. Health voucher<br>7. Food/cash for work<br>8. School feeding<br>9. Hunger safety net programme<br>97. Other	

For peer review only - http://bmjopen.bmj.com/site/about/guidelines.xhtml

[Name of Country]
[Name of Slum]

Q211	What type of fuel does your household mainly use for cooking?	1. Electricity<br>2. LPG/Natural gas<br>3. Biogas<br>4. Parafin/Kerosene<br>5. Coal, lignite<br>6. Charcoal<br>7. Wood<br>8. Straw/shrubs/grass<br>9. Agricultural crop<br>10. Animal dung<br>11. No food cooked in household……………→<br>97. Other	 <br><br><br><br><br><br><br><br><br>Q215
Q212	Where is cooking usually done?	1. In a room used for living or sleeping<br>2. In a separate room used as kitchen<br>3. In a separate building used as kitchen<br>4. Outdoor<br>97. Other, please specify:	
Q213	[MAIN MATERIAL OF THE FLOOR- RECORD OBSERVATION]	1. Natural floor: earth/sand<br>2. Natural floor: dung<br>3. Rudimentary floor: wood planks<br>4. Rudimentary floor: palm/bamboo<br>5. Finished floor: Parquet or polished wood<br>6. Finished floor: vinyl or asphalt strips<br>7. Finished floor: ceramic tiles<br>8. Finished floor: Cement<br>9. Finished floor: Carpet<br>97. Other	
Q214	[MAIN MATERIAL OF THE ROOF- RECORD OBSERVATION]	1. Natural roof: no roof<br>2. Natural roof: thatch/grass/makuti<br>3. Natural roof: dung/mud/sod<br>4. Rudimentary roof: iron sheets<br>5. Rudimentary roof: tin cans<br>6. Finished roof: asbestos sheet<br>7. Finished roof: concrete<br>8. Finished roof: tiles<br>97. Other	
Q215	[MAIN MATERIAL OF THE EXTERIOR WALLS-RECORD OBSERVATION]	1. Natural walls: no walls<br>2. Natural walls: cane/palm/trunks<br>3. Natural walls: dung/mud/sod<br>4. Rudimentary walls: bamboo with mud<br>5. Rudimentary walls: stone with mud<br>6. Rudimentary walls: uncovered adobe<br>7. Rudimentary walls: plywood<br>8. Rudimentary walls: cardboard<br>9. Rudimentary walls: reused wood<br>10. Rudimentary walls: iron sheets<br>11. Finished walls: cement<br>12. Finished walls: stone with lime/cement<br>13. Finished walls: brick<br>14. Finished walls: cement blocks<br>15. Finished walls: covered adobe<br>16. Finished walls: wood planks/shingles	

[Name of Country]
[Name of Slum]

		97. Other	
Q216	How many rooms in this household are used for sleeping?	☐☐ Rooms	
Q217	Does any member of this household own:	1. A watch<br>2. A bicycle<br>3. A motorcycle/scooter<br>4. An animal-drawn cart<br>5. A car/truck<br>6. A boat with a motor	
Q217A	Does your household own this structure (house, flat, shack)?	1. Yes<br>2. No	Q217B<br>Q217C
Q217B	Does your household own the land on which the structure (house, flat, shack) sits?	1. Yes<br>2. No	Q218<br>Q218
Q217C	Does your household pay rent?	6. Pays rent/lease<br>7. No rent with consent of owner<br>1. No rent: squatting	
Q218	Does any member of this household own any agricultural land?	2. Yes<br>3. No…………………………………… →	Q222
Q219	Does this household own any livestock, herds, other farm animals or poultry?	1. Yes<br>2. No…………………………………… →	Q224
Q220	How many of the following animals does this household own?<br><br>Local cattle (indigenous)<br>Exotic/grade cattle<br>Horses, donkeys, or camels?<br>Goats or sheep?<br>Pigs?<br>Chickens?<br>Other?	☐☐<br>☐☐<br>☐☐<br>☐☐<br>☐☐<br>☐☐	
Q221	Does any member of this household have a bank account?	1. Yes<br>2. No	
Q222	In the past 7 days were there days when your household did not have enough food or money to buy food?	1. Yes<br>2. No	
Q223	How many days in the month do you have air time (for calls and SMS) for at least one mobile phone in the household	1. Everyday<br>2. Almost every day (over 21 days a month but not every day)<br>3. More than half the days (over 14 but not as many as 21)<br>4. More than 7 days but less than 14<br>5. Less than 7 days<br>6. Never	
Q224	How many days in the month does someone in the household have data or access to wifi (for accessing internet for searching the web, using social media or using Email) for at least one of your digital communication devices I the household (smart phone, laptop, tablet)?	1. Everyday<br>2. Almost every day (over 21 days a month but not every day)<br>3. More than half the days (over 14 but not as many as 21)<br>4. More than 7 days but less than 14	

For peer review only - http://bmjopen.bmj.com/site/about/guidelines.xhtml

[Name of Country]
[Name of Slum]

		5. Less than 7 days<br>6. Never	

I would like to ask you some questions about your dwelling or home. NIGERIA VERSION

Q201	What is the main source of drinking water for members of your household?	1. Piped into dwelling……………………→<br>2. Piped to yard/plot……………………→<br>3. Public tap/standpipe<br>4. Tube well or Borehole<br>5. Dugwell: Protected well<br>6. Dugwell: Unprotected well<br>7. Protected spring<br>8. Unprotected spring<br>9. Rainwater……………………………→<br>10. Tanker truck<br>11. Cart with small tank<br>12. Surface water (river/dam/lake/pond/stream/ canal/irrigation channel)<br>13. Bottled water<br>97. Other	Q206<br>Q206<br><br><br><br><br><br><br>Q206
Q202	Where is that water source located?	1. In own dwelling……………………………→<br>2. In own yard/plot……………………………→<br>3. Elsewhere	Q206<br>Q206
Q203	How long does it take to go there, get water, and come back?	☐☐☐ Minutes<br>998. Don't know	
Q203A	Who usually goes to this source to fetch the water for your household	1. Adult woman<br>2. Adult man<br>3. Female child<br>4. Male child<br>5. No one person usually fetches the water	
Q204	Do you share this source with other households?	1. Yes<br>2. No……………………………………→	 Q206
Q205	How many households use this source of water?	No. of households if less than 10: 0☐<br><br>95. 10 of more households<br>98. Don't know	
Q206	Do you do anything to the water to make it safer to drink?	1. Yes<br>2. No……………………………………………→<br>8. Don't know……………………………………→	 Q207<br>Q207
Q206A	What do you usually do to make the water safer to drink?<br><br>Anything else?<br><br>[RECORD ALL MENTIONED]	1. Boil<br>2. Add bleach/chlorine<br>3. Strain through a cloth<br>4. Use water filter (ceramic/sand/composite/etc.)<br>5. Solar disinfection<br>6. Let it stand and Settle<br>7. Alum<br>97. Other	

For peer review only - http://bmjopen.bmj.com/site/about/guidelines.xhtml

[Name of Country]
[Name of Slum]

Q207	What kind of toilet facility do members of your household usually use?<br><br>[IF RESPONDENT INDICATES 'FLUSH' OR 'POUR FLUSH', THEN PROBE WITH:<br><br>WHERE DOES IT FLUSH TO?]	1. Flush/pour flush to piped sewer system<br>2. Flush/pour flush to septic tank<br>3. Flush/pour flush to pit latrine<br>4. Flush/pour flush to somewhere else<br>5. Flush/pour flush don't know where<br>6. Ventilated improved pit latrine<br>7. Pit latrine with slab<br>8. Pit latrine without slab/Open pit<br>9. Composting toilet<br>10. Bucket toilet<br>11. Hanging toilet/hanging latrine<br>12. No facility/bush/field……………………..→<br>97. Other	Q210
Q208	Do you share this toilet facility with other households?	1. Yes<br>2. No……………………………………….→	Q210
Q209	How many households use this toilet facility	No of household if less than 10 [0][ ]<br>95. 10 or more households<br>98. Don't know	
Q210	Does your household have:<br>[RECORD ALL THAT APPLY]	1. Electricity<br>2. Radio<br>3. Television<br>4. Mobile telephone<br>5. Non-mobile telephone<br>6. Refrigerator<br>7. Cable TV<br>8. Generating Set<br>9. Air conditioner<br>10. Computer<br>11. Electric Iron<br>12. Fan	
Q210A	Does this household receive a cash transfer or any social assistance from the government, NGOs, CBOs, churches or other organizations?<br><br>[DEFINE AS HAVING RECEIVED IN THE LAST MONTH IF UNSURE]	1. Yes<br>2. No…………………………………………….→	Q211
Q210B	For what reason does the household receive a cash transfer or social assistance?<br><br>Any other reason?<br><br>[RECORD ALL MENTIONED]	1. Orphaned children 18 years or younger<br>2. Elderly person<br>3. Person with severe disability<br>4. Urban food subsidy<br>5. Food aid for person in arid and semi-arid lands<br>6. Health voucher<br>7. Food/cash for work<br>8. School feeding<br>9. Hunger safety net programme<br>97. Other	
Q211	What type of fuel does your household mainly use for cooking?	1. Electricity<br>2. LPG<br>3. Natural gas<br>4. Biogas	

For peer review only - http://bmjopen.bmj.com/site/about/guidelines.xhtml

[Name of Country]
[Name of Slum]

		5. Kerosene<br>6. Coal, lignite<br>7. Charcoal<br>8. Wood<br>9. Straw/shrubs/grass<br>10. Agricultural crop<br>11. Animal dung<br>12. No food cooked in household.........→<br>97. Other	Q215
Q212	Where is cooking usually done?	1. In a room used for living or sleeping<br>2. In a separate room used as kitchen<br>3. In a separate building used as kitchen<br>4. Outdoors<br>97. Other, please specify:	
Q213	[MAIN MATERIAL OF THE FLOOR- RECORD OBSERVATION]	1. Natural floor: earth/sand<br>2. Natural floor: dung<br>3. Rudimentary floor: wood planks<br>4. Rudimentary floor: palm/bamboo<br>5. Finished floor: parquet or polished wood<br>6. Finished floor: vinyl or asphalt strips<br>7. Finished floor: ceramic tiles<br>8. Finished floor: cement<br>9. Finished floor: carpet/rug<br>97. Other	
Q214	[MAIN MATERIAL OF THE ROOF- RECORD OBSERVATION]	1. Natural roof: no roof<br>2. Natural roof: thatch/palm leaf<br>3. Rudimentary roof: rustic mat<br>4. Rudimentary roof: palm/bamboo<br>5. Rudimentary roof: wood planks<br>6. Rudimentary roof: cardboard<br>7. Finished roof: metal/zinc<br>8. Finished roof: wood<br>9. Finished roof: ceramic tiles<br>10. Finished roof: cement<br>11. Finished roof: roofing shingles<br>97. Other	
Q215	[MAIN MATERIAL OF THE EXTERIOR WALLS- RECORD OBSERVATION]	1. Natural walls: no walls<br>2. Natural walls: cane/palm/trunks<br>3. Natural walls: dirt (mud)<br>4. Rudimentary walls: bamboo with mud<br>5. Rudimentary walls: stone with mud<br>6. Rudimentary walls: plywood<br>7. Rudimentary walls: cardboard<br>8. Rudimentary walls: reused wood<br>9. Finished walls: cement<br>10. Finished walls: stone with lime/cement<br>11. Finished walls: bricks<br>12. Finished walls: cement blocks<br>13. Finished walls: wood planks/shingles<br>97. Other	
Q216	How many rooms in this household are used for sleeping?	☐☐ Rooms (Sleeping)	

[Name of Country]
[Name of Slum]

Q216A	How many rooms in total are in your household, including rooms for sleeping and all other rooms?	☐☐ Rooms (Total)	
Q217	Does any member of this household own:<br><br>[RECORD ALL THAT APPLY]	1. A watch<br>2. A bicycle<br>3. A motorcycle or motor scooter<br>4. An animal-drawn cart<br>5. A car or truck<br>6. A boat with a motor<br>7. A canoe	
Q217A	Does your household own this structure (house, flat, shack)?	1. Yes<br>2. No	Q217B<br>Q217C
Q217B	Does your household own the land on which the structure (house, flat, shack) sits?	1. Yes<br>2. No	Q218<br>Q218
Q217C	Do you pay rent?	8. Pays rent/lease<br>9. No rent with consent of owner<br>1. No rent: squatting	
Q218	Does any member of this household own any agricultural land?	2. Yes<br>3. No………………………………… →	Q222
Q219	Does this household own any livestock, herds, other farm animals or poultry?	1. Yes<br>2. No………………………………… →	Q224
Q220	How many of the following animals does this household own?<br><br>Milk cows or bulls?<br>Horses, donkeys, or mules?<br>Goats?<br>Sheep?<br>Chickens/ducks?<br>Pigs?<br>Other farm animals?		
Q221	Does any member of this household have a bank account?	1. Yes<br>2. No	
Q222	In the past 7 days were there days when your household did not have enough food or money to buy food?	1. Yes<br>2. No	
Q223	How many days in the month do you have air time (for calls and SMS) for at least one mobile phone in the household	1. Everyday<br>2. Almost every day (over 21 days a month but not every day)<br>3. More than half the days (over 14 but not as many as 21)<br>4. More than 7 days but less than 14<br>5. Less than 7 days<br>6. Never	
Q224	How many days in the month does someone in the household have data or access to wifi (for accessing internet for searching the web, using social media or using Email) for at least	1. Everyday<br>2. Almost every day (over 21 days a month but not every day)	

For peer review only - http://bmjopen.bmj.com/site/about/guidelines.xhtml

[Name of Country]
[Name of Slum]

	one of your digital communication devices I the household (smart phone, laptop, tablet)?	3. More than half the days (over 14 but not as many as 21)<br>4. More than 7 days but less than 14<br>5. Less than 7 days<br>6. Never	

I would like to ask you some questions about your dwelling or home. PAKISTAN VERSION

Q201	What is the main source of drinking water for members of your household?	1. Piped into dwelling…………………..→<br>2. Piped to yard/plot…………………..→<br>3. Public tap/standpipe<br>4. Tube well or Borehole<br>5. Handpump<br>6. Dugwell: Protected well<br>7. Dugwell: Unprotected well<br>8. Protected spring<br>9. Unprotected spring<br>10. Rainwater…………………………….→<br>11. Tanker truck<br>12. Filtration plant<br>13. Cart with small tank<br>14. Surface water (river/dam/lake/pond/stream/ canal/irrigation channel)<br>15. Bottled water<br>97. Other	Q206<br>Q206<br><br><br><br><br><br><br><br>Q206
Q202	Where is that water source located?	1. In own dwelling…………………………….→<br>2. In own yard/plot…………………………..→<br>3. Elsewhere	Q206<br>Q206
Q203	How long does it take to go there, get water, and come back?	☐☐☐ Minutes<br>998. Don't know	
Q203A	Who usually goes to this source to fetch the water for your household	1. Adult woman<br>2. Adult man<br>3. Female child<br>4. Male child<br>5. No one person usually fetches the water	
Q204	Do you share this source with other households?	1. Yes<br>2. No……………………………………….→	 Q206
Q205	How many households use this source of water?	No. of households if less than 10: 0☐<br><br>95. 10 of more households<br>98. Don't know	
Q206	Do you do anything to the water to make it safer to drink?	1. Yes<br>2. No…………………………………………………→<br>8. Don't know……………………………………→	 Q207<br>Q207
Q206A	What do you usually do to make the water safer to drink?<br><br>Anything else?	1. Boil<br>2. Add bleach/chlorine<br>3. Strain through a cloth	

[Name of Country]
[Name of Slum]

	[RECORD ALL MENTIONED]	4. Use water filter (ceramic/sand/composite/etc.)<br>5. Solar disinfection<br>6. Let it stand and Settle<br>97. Other	
Q207	What kind of toilet facility do members of your household usually use?<br><br>[IF RESPONDENT INDICATES 'FLUSH' OR 'POUR FLUSH', THEN PROBE WITH:<br><br>WHERE DOES IT FLUSH TO?]	1. Flush/pour flush to piped sewer system<br>2. Flush/pour flush to septic tank<br>3. Flush/pour flush to pit latrine<br>4. Flush/pour flush to somewhere else<br>5. Flush/pour flush don't know where<br>6. Ventilated improved pit latrine<br>7. Pit latrine with slab<br>8. Pit latrine without slab/Open pit<br>9. Bucket toilet<br>10. Hanging toilet/hanging latrine<br>11. No facility/bush/field..........................→<br>97. Other	Q210
Q208	Do you share this toilet facility with other households?	1. Yes<br>2. No..→	Q210
Q209	How many households use this toilet facility	No of household if less than 10 [0][ ]<br>95. 10 or more households<br>98. Don't know	
Q210	Does your household have:<br>[RECORD ALL THAT APPLY]	1. Electricity<br>2. Radio<br>3. Television<br>4. Landline telephone<br>5. Refrigerator<br>6. Almirah/cabinet<br>7. Chair<br>8. Room cooler<br>9. Air conditioner<br>10. Washing machine<br>11. Water pump<br>12. Bed<br>13. Clock<br>14. Sofa<br>15. Camera<br>16. Sewing machine<br>17. Computer<br>18. Internet connection	
Q210A	Does this household receive a cash transfer or any social assistance from the government, NGOs, CBOs, churches or other organizations?<br><br>[DEFINE AS HAVING RECEIVED IN THE LAST MONTH IF UNSURE]	1. Yes<br>2. No...→	Q211
Q210B	For what reason does the household receive a cash transfer or social assistance?<br><br>Any other reason?<br><br>[RECORD ALL MENTIONED]	98. Orphaned children 18 years or younger<br>99. Elderly person<br>100. Person with severe disability<br>101. Urban food subsidy	

For peer review only - http://bmjopen.bmj.com/site/about/guidelines.xhtml

[Name of Country]
[Name of Slum]

		102. Food aid for person in arid and semi-arid lands<br>103. Health voucher<br>104. Food/cash for work<br>105. School feeding<br>106. Hunger safety net programme<br>3. Other	
Q211	What type of fuel does your household mainly use for cooking?	1. Electricity<br>2. LPG<br>3. Natural gas<br>4. Biogas<br>5. Kerosene<br>6. Coal, lignite<br>7. Charcoal<br>8. Wood<br>9. Straw/shrubs/grass<br>10. Animal dung<br>11. No food cooked in household……….→<br>97. Other	 <br><br><br><br><br><br><br><br><br>Q215
Q212	Where is cooking usually done?	1. In a room used for living or sleeping<br>2. In a separate room used as kitchen<br>3. In a separate building used as kitchen<br>4. Outdoors<br>97. Other, please specify:	
Q213	[MAIN MATERIAL OF THE FLOOR- RECORD OBSERVATION]	1. Natural floor: earth/sand/mud<br>2. Natural floor: dung<br>3. Rudimentary floor: wood planks<br>4. Rudimentary floor: palm/bamboo<br>5. Finished floor: parquet or polished wood<br>6. Finished floor: vinyl or asphalt strips<br>7. Finished floor: ceramic tiles<br>8. Finished floor: cement<br>9. Finished floor: carpet<br>10. Finished floor: chips/terrazzo<br>11. Finished floor: bricks<br>12. Finished floor: mats<br>13. Finished floor: marble<br>97. Other	
Q214	[MAIN MATERIAL OF THE ROOF- RECORD OBSERVATION]	1. Natural roof: no roof<br>2. Natural roof: thatch/palm leaf<br>3. Natural roof: sod/grass<br>4. Rudimentary roof: rustic mat<br>5. Rudimentary roof: palm/bamboo<br>6. Rudimentary roof: wood planks<br>7. Rudimentary roof: cardboard<br>8. Finished roof: iron sheets/asbestos<br>9. Finished roof: reinforced brick cement/rcc metal<br>10. Finished roof: wood/t iron/mud	

For peer review only - http://bmjopen.bmj.com/site/about/guidelines.xhtml

[Name of Country]
[Name of Slum]

		11. Finished roof: calamine/cement fiber<br>12. Finished roof: ceramic tiles<br>13. Finished roof: cement/rcc<br>14. Finished roof: roofing shingles<br>97. Other	
Q215	[MAIN MATERIAL OF THE EXTERIOR WALLS-RECORD OBSERVATION]	1. Natural walls: no walls<br>2. Natural walls: cane/palm/trunks<br>3. Natural walls: dirt<br>4. Natural walls: mud/stones<br>5. Natural walls: bamboo/sticks/mud<br>6. Rudimentary walls: unbaked bricks/mud<br>7. Rudimentary walls: carton/plastic<br>8. Rudimentary walls: bamboo with mud<br>9. Rudimentary walls: stone with mud<br>10. Rudimentary walls: uncovered adobe<br>11. Rudimentary walls: plywood<br>12. Rudimentary walls: cardboard<br>13. Rudimentary walls: reused wood<br>14. Finished walls: baked bricks<br>15. Finished walls: tent<br>16. Finished walls: cement<br>17. Finished walls: stone with lime/cement<br>18. Finished walls: bricks<br>19. Finished walls: cement blocks<br>20. Finished walls: covered adobe<br>21. Finished walls: wood planks/shingles<br>97. Other	
Q216	How many rooms in this household are used for sleeping?	☐☐ Rooms (Sleeping)	
Q217	Does any member of this household own:	1. Watch<br>2. Mobile telephone<br>3. Bicycle<br>4. Motorcycle or motor scooter<br>5. An animal-drawn cart<br>6. A car or truck or bus<br>7. A tractor<br>8. A boat with a motor<br>9. A boat without a motor	
Q217A	Does your household own this structure (house, flat, shack)?	1. Yes<br>2. No	
Q217B	Does your household own the land on which the structure (house, flat, shack) sits?	1. Yes<br>2. No	
Q217C	Do you pay rent?	10. Pays rent/lease<br>11. No rent with consent of owner<br>1. No rent: squatting	
Q218	Does any member of this household own any agricultural land?	2. Yes<br>3. No……………………………. →	Q222

For peer review only - http://bmjopen.bmj.com/site/about/guidelines.xhtml

[Name of Country]
[Name of Slum]

Q219	Does this household own any livestock, herds, other farm animals or poultry?	1. Yes<br>2. No………………………………. →	 Q224
Q220	How many of the following animals does this household own?<br><br>Milk cows or bulls?<br>Horses, donkeys or mules?<br>Goats?<br>Sheep?<br>Chickens?<br>Buffalo?<br>Camels?<br>Other?		
Q221	Does any member of this household have a bank account?	1. Yes<br>2. No	
Q222	In the past 7 days were there days when your household did not have enough food or money to buy food?	1. Yes<br>2. No	
Q223	How many days in the month do you have air time (for calls and SMS) for at least one mobile phone in the household	1. Everyday<br>2. Almost every day (over 21 days a month but not every day)<br>3. More than half the days (over 14 but not as many as 21)<br>4. More than 7 days but less than 14<br>5. Less than 7 days<br>6. Never	
Q224	How many days in the month does someone in the household have data or access to wifi (for accessing internet for searching the web, using social media or using Email) for at least one of your digital communication devices I the household (smart phone, laptop, tablet)?	1. Everyday<br>2. Almost every day (over 21 days a month but not every day)<br>3. More than half the days (over 14 but not as many as 21)<br>4. More than 7 days but less than 14<br>5. Less than 7 days<br>6. Never	

Household income and expenditure

In the last part of this section, I will ask you about the total income for the household in the last 30 days. I would like to know about all sources of income. I know it may be difficult to calculate that figure, but please do try to give as accurate an amount as possible. Remember that all information will be kept strictly confidential. This information is important to assess overall health and wellbeing of people in your household compared to other, similar households.

Q225	I would like you to tell me your best estimate of the TOTAL income that this household has had in the last 30 days.	1. Less than 1000 KSh<br>2. 1000 – 2499 KSh<br>3. 2500 – 4999 KSh<br>4. 5000 – 7499 KSh<br>5. 7500 – 9999 KSh<br>6. 10000 – 14999 KSh<br>7. 15000 – 20000 KSh<br>8. Above 20000 KSh	

For peer review only - http://bmjopen.bmj.com/site/about/guidelines.xhtml

[Name of Country]
[Name of Slum]

Q226	Did your household receive or earn any income from the following in the last 30 days?	1. Salaried/waged employment<br>2. Business<br>3. Savings<br>4. Agriculture<br>5. Financial gifts/ support from any source<br>6. Any other source of income	

How much in total did your household spend on the following items?<br><br>[IF RESPONDENT UNSURE, PLEASE PROMPT WITH “TO THE BEST OF YOUR RECOLLECTION” THEN “CAN YOU ESTIMATE APPROXIMATELY WHAT IT MIGHT HAVE BEEN?”]			
Q227a	FOOD in the last 7 days?	☐☐☐☐☐☐☐☐<br>8. Don’t know	
Q227b	ENERGY (PARAFFIN, CHARCOAL) in the last 7 days?	☐☐☐☐☐☐☐☐<br>8. Don’t know	
Q227c	WATER in the last 7 days?	☐☐☐☐☐☐☐☐<br>8. Don’t know	
Q227d	TRANSPORT in the last 7 days?	☐☐☐☐☐☐☐☐<br>8. Don’t know	
Q227e	FINANCIAL GIFT/SUPPORT TO OTHERS in the last 7 days?	☐☐☐☐☐☐☐☐<br>8. Don’t know	
Q227f	ELECTRICITY in the last 30 days?	☐☐☐☐☐☐☐☐<br>8. Don’t know	
Q227g	HEALTH CARE in the last 30 days?	☐☐☐☐☐☐☐☐<br>8. Don’t know	
Q227h	RELIGIOUS OBLIGATIONS in the last 30 days?	☐☐☐☐☐☐☐☐<br>8. Don’t know	
Q227i	RENT in the last month?	☐☐☐☐☐☐☐☐<br>8. Don’t know	
Q227i	SCHOOL RELATED EXPENSES (school fees, scholastic materials) in the last 30 days?	☐☐☐☐☐☐☐☐<br>8. Don’t know	
Q227k	OTHER in the last 7 days?	☐☐☐☐☐☐☐☐<br>8. Don’t know	

For peer review only - http://bmjopen.bmj.com/site/about/guidelines.xhtml

[Name of Country]
[Name of Slum]

For peer review only

For peer review only - http://bmjopen.bmj.com/site/about/guidelines.xhtml

[Name of Country]
[Name of Slum]

Module 3: Household Health Care Spending

I would like to ask you more specific questions about how much your household and all its members spent in cash or in-kind on all health care and services that did not require an overnight stay. We want expenses in the last 3 months. If payment was in-kind, please estimate a monetary value. Please exclude costs to be reimbursed by insurance.

In the last 3 months, how much did your household spend on:		
Q301	Registration and consultation fees by doctors, nurses, or trained midwives that did not require an overnight stay?	☐☐☐☐☐☐☐☐<br>8. Don't know
Q302	Health-care by traditional or alternative healers?<br>[USE COUNTRY-SPECIFIC TERMS]	☐☐☐☐☐☐☐☐<br>8. Don't know
Q303	Diagnostic and laboratory tests, such as x-rays or blood tests?	☐☐☐☐☐☐☐☐<br>8. Don't know
Q304	Medications or drugs (prescription, non-prescription, traditional, homeopathic…)?	☐☐☐☐☐☐☐☐<br>8. Don't know
Q305	Dentists or dental care?	☐☐☐☐☐☐☐☐<br>8. Don't know
Q306	Ambulance or other transport?	☐☐☐☐☐☐☐☐<br>8. Don't know
Q307	Costs associated with overnight stays in a hospital or health facility? Please exclude any reimbursements from insurance and transportation costs.	☐☐☐☐☐☐☐☐<br>8. Don't know
Q308	Any other health care products or services that were not included above? Please specify:<br><br>[PROMPT FOR FOOD COSTS IF PROVIDED TO HOUSEHOLD MEMBERS DURING HOSPITAL/CLINIC STAYS; PROMPT FOR HOME BASED CARE PROVIDED FOR HOUSEHOLD MEMBERS (ADULT OR CHILD) DUE TO LONG-TERM CONDITIONS OR GETTING OLD AND WEAK]	☐☐☐☐☐☐☐☐<br>8. Don't know

For peer review only

[Name of Country]
[Name of Slum]
Finally, I want you to think of how you paid for your health care expenditures over the last 12 months. This includes costs for all fees, services and goods, including overnight stays.

Q309	In the last 12 months , have you borrowed from financial institutions, agencies (microfinance schemes, banks…), or individuals to pay for any health expenditures?	1. Yes<br>2. No<br>8. Don't know	
Q310	If you borrowed any money in the last 12 months to pay for health expenditures, are you expected to pay this back?	1. Yes……………………………………… →<br>2. No…………………………………… →	Q311<br>END
Q311	Have you started paying back the loan?	1. Yes……………………………………… →<br>2. No…………………………………… →	Q312<br>END
Q311A	Are you repaying or have you repayed the loan as a one-off payment or in installments?	1. Lump sum<br>2. Installments	Q312A<br>Q312B
Q312A	How much did you pay back?	________	Q401
Q312B	What is the monthly repayment on the loan including interest?<br><br>[FOR INFORMAL PAYMENT, PLEASE TEASE OUT THE AVERAGE MONTHLY REPAYMENT, IF ANY]	☐☐☐☐☐☐☐☐<br><br>8. Don't know	

END: Thank you for your time in completing the household questionnaire. I will now move on to complete an individual questionnaire with a member of your household.

For peer review only - http://bmjopen.bmj.com/site/about/guidelines.xhtml

STROBE Statement—Checklist of items that should be included in reports of ***cross-sectional studies***

	Item No	Recommendation	Page #
**Title and abstract**	1	(*a*) Indicate the study's design with a commonly used term in the title or the abstract	1
		(*b*) Provide in the abstract an informative and balanced summary of what was done and what was found	4-5
**Introduction**			
Background/rationale	2	Explain the scientific background and rationale for the investigation being reported	7
Objectives	3	State specific objectives, including any prespecified hypotheses	7-8
**Methods**			
Study design	4	Present key elements of study design early in the paper	8-9
Setting	5	Describe the setting, locations, and relevant dates, including periods of recruitment, exposure, follow-up, and data collection	8-9
Participants	6	(*a*) Give the eligibility criteria, and the sources and methods of selection of participants	8-9
Variables	7	Clearly define all outcomes, exposures, predictors, potential confounders, and effect modifiers. Give diagnostic criteria, if applicable	8-11
Data sources/ measurement	8*	For each variable of interest, give sources of data and details of methods of assessment (measurement). Describe comparability of assessment methods if there is more than one group	*8-11*
Bias	9	Describe any efforts to address potential sources of bias	8-11, 25, 26
Study size	10	Explain how the study size was arrived at	9
Quantitative variables	11	Explain how quantitative variables were handled in the analyses. If applicable, describe which groupings were chosen and why	10-12
Statistical methods	12	(*a*) Describe all statistical methods, including those used to control for confounding	10-13
		(*b*) Describe any methods used to examine subgroups and interactions	N/A
		(*c*) Explain how missing data were addressed	N/A
		(*d*) If applicable, describe analytical methods taking account of sampling strategy	11-12
		(*e*) Describe any sensitivity analyses	N/A
**Results**			
Participants	13*	(a) Report numbers of individuals at each stage of study—eg numbers potentially eligible, examined for eligibility, confirmed eligible, included in the study, completing follow-up, and analysed	14
		(b) Give reasons for non-participation at each stage	N/A
		(c) Consider use of a flow diagram	N/A
Descriptive data	14*	(a) Give characteristics of study participants (eg demographic, clinical, social) and information on exposures and potential confounders	15
		(b) Indicate number of participants with missing data for each variable of interest	N/A
Outcome data	15*	Report numbers of outcome events or summary measures	14-20
Main results	16	(*a*) Give unadjusted estimates and, if applicable, confounder-adjusted estimates and their precision (eg, 95% confidence interval). Make clear	12-13, 20-21

For peer review only - http://bmjopen.bmj.com/site/about/guidelines.xhtml

		which confounders were adjusted for and why they were included	
		(*b*) Report category boundaries when continuous variables were categorized	N/A
		(*c*) If relevant, consider translating estimates of relative risk into absolute risk for a meaningful time period	N/A
Other analyses	17	Report other analyses done—eg analyses of subgroups and interactions, and sensitivity analyses	N/A
**Discussion**			
Key results	18	Summarise key results with reference to study objectives	22-24
Limitations	19	Discuss limitations of the study, taking into account sources of potential bias or imprecision. Discuss both direction and magnitude of any potential bias	25, 26
Interpretation	20	Give a cautious overall interpretation of results considering objectives, limitations, multiplicity of analyses, results from similar studies, and other relevant evidence	22-26
Generalisability	21	Discuss the generalisability (external validity) of the study results	22-26
**Other information**			
Funding	22	Give the source of funding and the role of the funders for the present study and, if applicable, for the original study on which the present article is based	5

*Give information separately for exposed and unexposed groups.

Note: An Explanation and Elaboration article discusses each checklist item and gives methodological background and published examples of transparent reporting. The STROBE checklist is best used in conjunction with this article (freely available on the Web sites of PLoS Medicine at http://www.plosmedicine.org/, Annals of Internal Medicine at http://www.annals.org/, and Epidemiology at http://www.epidem.com/). Information on the STROBE Initiative is available at www.strobe-statement.org.

For peer review only

For peer review only - http://bmjopen.bmj.com/site/about/guidelines.xhtml

BMJ Open

Primary-care doctor and nurse consultations among people who live in slums: A retrospective, cross-sectional survey in four countries

Journal:	*BMJ Open*
Manuscript ID	bmjopen-2021-054142.R2
Article Type:	Original research
Date Submitted by the Author:	28-Oct-2021
Complete List of Authors:	Watson, Samuel; University of Birmingham<br>Improving Health in Slums Collaborative, .; University of Birmingham
**Primary Subject Heading**:	Health services research
Secondary Subject Heading:	Health policy
Keywords:	PRIMARY CARE, PUBLIC HEALTH, International health services < HEALTH SERVICES ADMINISTRATION & MANAGEMENT, Organisation of health services < HEALTH SERVICES ADMINISTRATION & MANAGEMENT

SCHOLARONE™
Manuscripts

I, the Submitting Author has the right to grant and does grant on behalf of all authors of the Work (as defined in the below author licence), an exclusive licence and/or a non-exclusive licence for contributions from authors who are: i) UK Crown employees; ii) where BMJ has agreed a CC-BY licence shall apply, and/or iii) in accordance with the terms applicable for US Federal Government officers or employees acting as part of their official duties; on a worldwide, perpetual, irrevocable, royalty-free basis to BMJ Publishing Group Ltd ("BMJ") its licensees and where the relevant Journal is co-owned by BMJ to the co-owners of the Journal, to publish the Work in this journal and any other BMJ products and to exploit all rights, as set out in our licence.

The Submitting Author accepts and understands that any supply made under these terms is made by BMJ to the Submitting Author unless you are acting as an employee on behalf of your employer or a postgraduate student of an affiliated institution which is paying any applicable article publishing charge ("APC") for Open Access articles. Where the Submitting Author wishes to make the Work available on an Open Access basis (and intends to pay the relevant APC), the terms of reuse of such Open Access shall be governed by a Creative Commons licence – details of these licences and which Creative Commons licence will apply to this Work are set out in our licence referred to above.

Other than as permitted in any relevant BMJ Author's Self Archiving Policies, I confirm this Work has not been accepted for publication elsewhere, is not being considered for publication elsewhere and does not duplicate material already published. I confirm all authors consent to publication of this Work and authorise the granting of this licence.

For peer review only

Primary-care doctor and nurse consultations among people who live in slums: a retrospective, cross-sectional survey in four countries

Improving Health in Slums Collaborative

Improving Health in Slums Collaborative members (in alphabetical order):

African Population and Health Research Centre (APHRC), Nairobi, Kenya
Pauline Bakibinga, Caroline Kabaria, Ziraba Kasiira, Peter Kibe, Catherine Kyobutungi, Nelson Mbaya, Blessing Mberu, Shukri Mohammed, Anne Njeri,

Aga Khan University, Karachi, Pakistan
Iqbal Azam, Romaina Iqbal, Ahsana Nazish, Narijis Rizvi,

Independent University, Bangladesh, Dhaka, Bangladesh
Syed A. K. Shifat Ahmed, Nazratun Choudhury, Ornob Alam, Afreen Zaman Khan, Omar Rahman, Rita Yusuf

Nigerian Academy of Sciences, Lagos, Nigeria
Doyin Odubanjo

University of Ibadan, Ibadan, Nigeria
Motunrayo Ayobola, Olufunke Fayehun, Akinyinka Omigbodun, Mary Osuh, Eme Owoaje, Olalekan Taiwo

University of Birmingham, Birmingham, UK
Richard J Lilford, Jo Sartori, Samuel I Watson

University of Lancaster, Lancaster, UK
Peter J Diggle

University of Warwick, Coventry, UK
Navneet Aujla, Yen-Fu Chen, Christopher Conlan, Paramjit Gill, Frances Griffiths, Bronwyn Harris, Jason Madan, Helen Muir, Oyinlola Oyebode, Vangelis Pitidis, João Porto de Albuquerque, Simon Smith, Celia Taylor, Grant Tregonning, Philip Ulbrich, Olalekan A Uthman, Ria Wilson, Godwin Yeboah, Ji-Eun Park

Corresponding author

Sam Watson

For peer review only

Institute for Applied Health Research, University of Birmingham, Birmingham, United Kingdom

s.i.watson@bham.ac.uk

Word count

3,861 words

Keywords

Healthcare access, health, LMICs, slums, welfare

Data availability statement

The data are available from the authors on request.

Author Contributions

Improving Health in Slums Collaborative drafted the manuscript and conducted the analyses. All members of the collaborative were involved in the design of the project, data collection, data analysis, and approved the final manuscript. SW submitted the paper.

Conflicts of Interest

None declared.

For peer review only

ABSTRACT

Objectives

To survey on the availability and use of primary care services in slum populations.

Design

Retrospective, cross-sectional, household, individual, and healthcare provider surveys.

Setting

Seven slum sites in four countries (Nigeria, Kenya, Pakistan, and Bangladesh).

Participants

Residents of slums and informal settlements.

Primary and secondary outcome measures

Primary care consultation rates by type of provider and facility.

Results

We completed 7,692 household and 7,451 individual adult and 2,633 individual child surveys across seven sites. The majority of consultations were to doctors/nurses (in clinics or hospitals) and pharmacies rather than single handed providers or traditional healers. Consultation rates with a doctor or nurse varied from 0.2 to 1.5 visits per person-year, which was higher than visit rates to any other type of provider in all sites except Bangladesh, where pharmacies predominated. Approximately half the doctor/nurse visits were in hospital outpatient departments and most of the remainder were to clinics. Over 90% of visits across all sites were for acute symptoms rather than chronic disease. Median travel times were between 15 and 45 minutes and the median cost per visit was between 2 and 10% of a household's monthly total expenditure. Medicines comprised most of the cost. More respondents reported proximity (54-78%) and service quality

For peer review only - http://bmjopen.bmj.com/site/about/guidelines.xhtml

(31-95%) being a reason for choosing a provider than fees (23-43%). Demand was relatively inelastic with respect to both price of consultation and travel time.

Conclusions

People in slums tend to live sufficiently close to formal doctor/nurse facilities for their health seeking behaviour to be influenced by preference for provider type over distance and cost. However, costs, especially for medicines are high in relation to income and use rates remain significantly below those of high-income countries.

Funding

This research was funded by the National Institute for Health Research (NIHR) (16/136/87) using UK aid from the UK Government to support global health research. RJL is also funded from the NIHR Applied Research Collaboration (ARC) West Midlands and PG is an NIHR Senior Investigator. The views expressed in this publication are those of the author(s) and not necessarily those of the NIHR or the UK government.

Strength and limitations of this study

- This study provides the most comprehensive evidence across multiple nations to date on the facilities available to people who live in slums in general and on doctor and nurse consultations.
- We surveyed over 7,000 individuals across seven slum sites in four counties on their access to and use of healthcare.
- We estimated consultation rates and provider types for primary care visits as well as the elasticity of demand for provider type with respect to time and cost of a visit.
- While several attempts were made to contact participants, our response rates ranged from 68% to 94% across the sites, and so our sample may miss some of the most vulnerable residents of the study areas.

For peer review only - http://bmjopen.bmj.com/site/about/guidelines.xhtml

For peer review only

- We could not make a reliable assessment of patient need for healthcare so it is difficult to interpret these results in terms of the equity of access to care.

INTRODUCTION

Strengthening primary care is recognized as the most “inclusive, effective, and efficient approach” to improving population health and wellbeing and as being key to achieving Universal Health Care (UHC) globally as codified in recent international declarations.[1] However, recent high-profile analyses have shown that little systematic evidence has been collected on services that are available, how often they are used, how much they cost and what type of service patients will choose under various constraints.[2–5] It is widely believed though that people in LMIC have poor access to high quality primary care services.[2,3,6–8]

In this paper we focus on a large and vulnerable group of people; those who live in slums. A recent Lancet Series documented the many barriers that people is slums encounter in accessing services.[4,5] Following further review of the literature,[9] most evidence relates to specific programs, particularly those concerned with vaccination and childbirth (e.g.[10]). The little available evidence suggests that in many urban slums, pharmacies and drug sellers are frequently the first and only point of contact with a health system.[11,12] However, consultation with a doctor or nurse is an essential component of primary care providing diagnosis, treatment, advice and referral. Outpatient doctor and nurse consultations can be provided by public and private facilities that range from “single-handed”, sometimes informal, practitioners in solo practices to large hospital outpatient departments.

The purpose of our study is to start to fill the above gaps in the literature, examining outpatient care services in seven slum sites across four countries: Nigeria, Kenya, Pakistan, and Bangladesh. We compare visit rates to all providers that might offer primary care services

For peer review only - http://bmjopen.bmj.com/site/about/guidelines.xhtml

including traditional/faith healers and pharmacies/medicine sellers. We then describe doctor and nurse services in more detail. Here we aim to find out use rates of different facilities that provide outpatient doctor/nurse consultations for people who live in slums, including solo providers, clinics, hospitals, and public verses private provision. We also aim to estimate how individuals trade-off characteristics like cost and time when choosing between types of provider of outpatient care.

METHODS

Setting

Our aim was to examine the use of outpatient consultations with a doctor or nurse. The results reported in this article form part of a broader programme of work on health and healthcare in informal settlements described in detail elsewhere.[13]

We conducted a series of household, individual, and healthcare facility surveys across seven informal settlements in four countries: Nigeria, Kenya, Pakistan, and Bangladesh. We selected sites that (1) fulfilled the UN definition of a 'slum'[14] and (2) were named, geographically distinguishable, neighborhoods within city boundaries. This also provided a geographical basis on which to survey available health services.

Study design and population

Household and individual surveys

The design and methods of the survey components of the Improving Health in Slums project have been published elsewhere.[13] Briefly, we aimed to conduct a spatially-referenced, household-based, retrospective, cross-sectional survey in seven sites, which are described in Table 1. Key indicators for the health systems of the respective countries are reported in Supplementary Information Table C1.

For peer review only - http://bmjopen.bmj.com/site/about/guidelines.xhtml

All structures in each site were first mapped using satellite imagery. The resulting maps were then "ground-truthed" through participatory geospatial mapping and corrected as required. During this stage all households in each structure were identified, which formed the sampling frame for the household survey. We used an inhibitory sampling design with close pairs to generate a spatially-regular and well-dispersed sample of 1,200 households for each study site,[15] assuming a response rate of 80%. Within each household, all residents were identified and an adult over 18 and a child under 12 (should one be resident) were selected at random for the individual surveys. Adult women were oversampled compared to men at a ratio of 2:1 to achieve reasonable precision in both groups as we expected greater healthcare use among women. Up to three attempts were made to complete each survey should the participant not be available at the initial attempt. For each survey instrument we sought consent from the respondent. They were provided with an oral and/or written explanation of the study and their involvement as required and then asked to provide written consent on both a paper and digital copy of the consent form. Due to an error with the sampling process, a follow-up telephone survey was conducted in PK1 to supplement the individual surveys with additional female respondents, which is described in Supplementary Information Section B2.

For peer review only - http://bmjopen.bmj.com/site/about/guidelines.xhtml

Table 1. Summary of study sites

Site	Location	Approx. pop. (000s)*	Approx. density (000s/km^2)	Description of population and area
NG1	Ibadan, Nigeria	5.8	5	Resettled, mixed Yoruba and Hausa community at the edge of the city including a large proportion of recent migrants from the North. Structures are mostly permanent and well-spaced. Variable access to energy with poor sanitation.
NG2	Ibadan, Nigeria	5.5	14	Mostly Yoruba population inhabiting a central, historical part of the city. Building mostly permanent but dilapidated with access to energy but little sanitation.
NG3	Lagos, Nigeria	8.1	11	Mixed Yoruba, Ilajes and other ethnic group population in low-paying or no employment. High crime area with little development and temporary structures. Little access to basic services.
KE1	Nairobi, Kenya	24.4	52	Mixed but segregated ethnicity community of generally long-term multi-generational residents. Structures are temporary with little to no access to basic services.
KE2	Nairobi, Kenya	44.9	83	Mixed community of predominantly economic migrants at the edge of an industrial area. Structures are temporary with little to no access to basic services.
PK1	Karachi, Pakistan	33.5	91	Mixed ethnicity and religion, mostly permanent, population working in blue collar jobs. Structures are permanent and multi-story with access to transient energy and sanitation services.
BD1	Dhaka, Bangladesh	60.0	171	Mostly Bengali, Muslim population working in manual services like rickshaw pulling and house work. Semi-permanent residential structures with variable access to water, sanitation, and other services.

*Estimated from data collected in this study.

The NIHR Global Health Research Unit on Improving Health in Slums was granted full ethical approval by the Ministry of Health, Lagos State Government (LSMH/2695/11/259) the Ministry of Health, Oyo State Government (ADB/479/657), Amref Health Africa (AMREF-ESRC P440/2018), the National Bioethics Committee Pakistan (4-87/NBC-298/18/RDC3530), the Bangladesh Medical Research Council and the University of Warwick Biomedical and Scientific Research Ethics Sub-Committee (REGO-2017-2043 AM01).

Procedures

Three instruments were used in the household surveys: (i) a household level survey of demographic and socioeconomic characteristics, including monthly household expenditures

For peer review only - http://bmjopen.bmj.com/site/about/guidelines.xhtml

across different categories; (ii) an individual adult survey enquiring about health care need, access, and use, and health and well-being; and (iii) a child (under 12 years of age) survey asking a caregiver about health care needs, access, and use. Questions were adapted from similar studies to facilitate comparability.[16] The adult and child surveys both asked the respondents to provide details of the last time they used healthcare (if they had done so in the previous 12 months), including facility type (public clinic, private hospital, etc.), who they saw (doctor, nurse, etc.), the reason for the visit, the cost and time taken for the visit, and questions regarding their satisfaction with the care provided. We included "doctor's office" or "chamber" among the list of responses to facility-type, which generally refers to a solo clinician working alone in a private office – we refer to this category as "single-handed". Survey instruments were translated using an iterative process involving forward and independent backward translations. (Survey forms are in the Supplementary Information adult.pdf, child.pdf, and household.pdf).

Outcomes and Statistical analysis

Summary statistics

From the individual survey data we identified all reported outpatient consultations to any type of provider and calculated visit rates per patient-year for each type of provider (doctor/nurse, pharmacy, traditional/faith, other) for new and existing conditions and for adults and children under 12 for comparison. We report crude rates as well as age standardized consultation rates calculated with respect to both the WHO reference population,[17] and INDEPTH population.[18] For doctor and nurse visits we further examined the proportion of visits by facility type (single-handed, public/private hospital, or public/private clinic) and reported provider type (doctor or nurse).

We estimated key characteristics of these visits including the median [Interquartile range (IQR)] time required for the visit, median [IQR] cost of the visit, proportion of households spending more

For peer review only - http://bmjopen.bmj.com/site/about/guidelines.xhtml

than 10% of their monthly expenditure on a visit (as used by the WHO's Global Health Observatory), reason for choosing the provider, and satisfaction by site and facility type.

Choice model

To estimate the role of price and travel time in the choice over a provider for a doctor or nurse for an outpatient consultation, we estimated a 'choice model'. We specified a random parameters logit model,[19] which we describe in detail in the Supplementary Information. In the model, each option from a set of choices has an observed component (price and time), the utility of which is determined by observed (e.g. age, sex) and unobserved (e.g. taste, quality) characteristics. There are several examples of choice modelling for healthcare providers in LMICs (for example, Borah (2006)[20]). The "choice set" we examined was: private clinic, public clinic, private hospital, or public hospital. We also included "single-handed" doctor's office for Bangladesh and Pakistan, since this type of consultation was rare or non-existent in our African sites.

We studied each site separately. There were four estimates of interest from each model: the predicted proportion of visits to each provider type "holding fixed" price and travel time, the price elasticity of demand, the travel time elasticity of demand, and the average change in price (willingness to pay, WTP) to make an individual indifferent between two options, one of which is 15 minutes further travel away than the other. The elasticities and WTP were calculated separately for households whose monthly consumption expenditure was above and below Int$ 100 per person per month. An elasticity is interpreted as the percentage change in demand you would expect for a 1% increase in price or travel time. We allowed preferences to vary by age, sex, secondary education, seeking care for an acute or communicable condition, or for chronic or generalized pain, and monthly consumption expenditure. The "price" of a visit included the consultation fee plus the travel cost, which would be known up front to the individual, and not drugs or tests, which would not be known in advance. The prices and times of travel for providers not visited were imputed based on the above listed individual covariates.

For peer review only - http://bmjopen.bmj.com/site/about/guidelines.xhtml

Patient and public involvement

Mapping of the study sites, identification of healthcare facilities, and enumeration of resident households was conducted using a participatory process involving local residents. Healthcare facility managers and owners were consulted about identification of their facilities. The public were not involved in the design of the survey questionnaires, however feedback was sought from residents in a pilot survey in all sites to assess the time burden of participating. Patient and public focus groups were established to present the findings, receive feedback, and provide contextualizing interpretation of the results.

For peer review only

For peer review only - http://bmjopen.bmj.com/site/about/guidelines.xhtml

RESULTS

Household and Individual Sample

Overall, 7,692 households participated in the surveys with 7,451 individual adults and 2,633 individual child surveys completed. The median response rate was 69%, varying by site from 57% in Site KE2 to 94% in Site BD1. Table 2 reports demographic and socioeconomic statistics of the population-weighted sample of individual respondents by site, and for those reporting an outpatient consultation (population summaries are reported in Table C2, Supplementary Information).

Outpatient consultation rates across providers of all types

Figure 1 shows the visit rates per patient-year to different types of provider. Between 29% (BD1) and 61% (KE1) of visits were to doctors and nurses, while the majority of other visits were to a pharmacy. In only two sites (BD1 and KE2) were pharmacies visited more frequently than doctors or nurses. The proportion of outpatient consultations that were for new conditions ranged from 61% (NG2) to 84% (KE2). Proportionately very few healthcare visits were made to traditional or faith healers.

Outpatient consultation rates to a doctor or nurse

Table 3 reports the outpatient consultation rates by study site for both adults and children (under 12 and under 5) – equivalent rates for new conditions only are reported in Table C3 Supplementary Information. Nigerian sites had consistently the lowest outpatient consultation rates, which were comparable for adults and children: approximately 0.2 to 0.4 outpatient visits to a doctor or nurse for a new condition per patient-year. Rates were higher in other sites, ranging, for adults, from approximately 0.8 (PK1) to 1.5 (BD1) visits per patient-year. Apart from Nigeria, outpatient consultation rates were higher for children than adults.

For peer review only - http://bmjopen.bmj.com/site/about/guidelines.xhtml

TABLE 2 Summary statistics of the overall study sample (All) and those reporting a doctor or nurse (D/N) consultation for a new condition visit by site. Values are mean (sd) unless otherwise stated.

Variable		Nigeria						Kenya				Pakistan		Bangladesh	
		NG1		NG2		NG3		KE1		KE2		PK1		BD1	
		All	D/N	All	D/N	All	D/N	All	D/N	All	D/N	All	D/N	All	D/N
Adults															
N		1,278	391	840	215	802	201	1,008	405	1,085	377	1,112	498	990	257
Household size		3.6 (1.8)	3.8 (1.8)	4.7 (1.4)	4.9 (1.3)	4.6 (1.8)	4.7 (1.8)	3.5 (2.1)	3.1 (2.0)	2.5 (1.4)	2.4 (1.3)	5.5 (2.6)	5.5 (2.5)	4.3 (1.9)	4.4 (2.0)
Wealth quintile (%)	Bottom	0	0	0	0	0	0	0	0	0	0	0	0	0	0
	Lower	2	3	3	0	1	1	15	13	0	0	0	0	0	0
	Middle	49	48	51	47	24	24	59	61	55	54	83	83	21	19
	Upper	48	49	46	53	75	75	25	26	44	45	15	15	78	77
	Top	0	0	0	0	0	0	0	0	1	1	3	2	1	3
Monthly household expenditure (Int$)	Total	300 (248)	362 (250)	342 (274)	349 (268)	560 (364)	580 (363)	232 (136)	213 (127)	243 (127)	274 (152)	1,122 (631)	1,179 (667)	463 (234)	505 (257)
	Per person	99 (86)	108 (79)	95 (78)	101 (87)	136 (101)	142 (104)	84 (62)	87 (63)	118 (81)	130 (92)	223 (125)	230 (138)	116 (58)	126 (61)
Age		39.0 (16.7)	39.9 (17.4)	47.8 (16.6)	51.3 (17.7)	42.0 (15.0)	43.8 (15.2)	37.6 (13.6)	40.1 (14.7)	34.3 (11.1)	34.4 (11.0)	37.6 (12.6)	39.2 (13.1)	34.6 (12.3)	36.6 (12.7)
Sex (% male)		45	35	49	50	47	45	46	34	46	42	50	42	53	41
Education (%)	Primary/middle	22	19	34	36	16	19	60	55	39	38	7	10	61	58
	Secondary	46	52	55	52	54	49	34	38	51	49	79	76	31	30
	Tertiary	24	22	9	9	32	31	6	7	8	12	14	14	8	12
Children (under 12)															
N		128	36	69	13	79	24	537	320	421	205	528	367	658	136
Household size		4.9 (1.5)	4.9 (1.4)	4.7 (1.4)	4.9 (1.3)	4.9 (1.4)	5.1 (1.4)	5.2 (1.8)	5.2 (1.9)	4.4 (1.4)	4.3 (1.4)	7.5 (3.0)	7.4 (2.9)	4.9 (1.6)	5.4 (1.8)
Wealth quintile (%)	Lowest	0	0	0	0	0	0	0	0	0	0	0	0	0	0
	Lower	2	1	3	0	1	0	13	13	0	0	0	0	0	0
	Middle	48	62	51	47	25	37	60	56	55	55	84	85	25	20
	Upper	50	37	46	53	74	63	27	30	44	45	15	14	74	78
	Top	0	0	0	0	0	0	0	1	0	0	1	1	1	2
Monthly household expenditure (Int$)	Total	365 (315)	359 (227)	437 (310)	417 (254)	579 (336)	626 (386)	284 (29)	274 (152)	276 (132)	286 (143)	1,304 (733)	1,324 (740)	472 (235)	525 (252)
	Per person	74 (58)	75 (49)	93 (60)	92 (59)	123 (68)	130 (75)	56 (29)	130 (92)	69 (37)	70 (36)	184 (112)	194 (117)	100 (46)	104 (50)
Age		6.9 (3.6)	6.0 (3.4)	6.7 (3.7)	4.9 (3.3)	6.5 (3.9)	6.2 (3.8)	5.9 (3.7)	5.5 (3.8)	5.6 (3.6)	5.1 (3.7)	6.0 (3.7)	5.4 (3.4)	5.5 (3.7)	4.7 (3.7)
Sex (% male)		44	33	57	51	43	40	55	52	48	46	53	53	52	62

For peer review only - http://bmjopen.bmj.com/site/about/guidelines.xhtml

Doctor and nurse consultation rates by provider type

Figure 2 shows the proportion of outpatient consultations for a new condition by facility type. There were differences between the sites and countries. Single-handed facilities accounted for approximately 25% of adult visits in Bangladesh and Pakistan, and 50% and 20% of child visits in these countries, respectively. However, almost no visits to single-handed facilities were recorded in Nigeria and Kenya. Hospital outpatient departments and clinics accounted for comparable shares of outpatient consultations; in particular, for adults, hospital visit shares were 51% (NG1), 66% (NG2), 69% (NG3), 38% (KE1), 39% (KE2), 37% (PK1), and 21% (BD1). These figures were similar for children. Figure 3 shows the proportion of visits by provider (doctor or nurse). For the Pakistani and Bangladeshi communities, almost all outpatient consultations were with a doctor, whereas in Kenya and Nigeria a significant minority of consultations were with a nurse for both adults and children.

For peer review only

Table 3. Outpatient primary care consultation rates and outpatient doctor and nurse consultation rates (visits per person-year (95% CI)).

Site	Outpatient consultation (all conditions)		
	**Crude**	**WHO age adjusted**	**INDEPTH age adjusted**
	Adults		
NG1	0.42 (0.40, 0.44)	0.42 (0.40, 0.44)	0.40 (0.38, 0.42)
NG2	0.39 (0.35, 0.43)	0.32 (0.30, 0.34)	0.29 (0.27, 0.31)
NG3	0.28 (0.26, 0.30)	0.31 (0.29, 0.33)	0.29 (0.27, 0.31)
KE1	1.07 (1.03, 1.11)	1.17 (1.13, 1.21)	1.04 (1.00, 1.08)
KE2	0.93 (0.89, 0.97)	1.06 (1.02, 1.10)	0.95 (0.91, 0.99)
PK1	0.79 (0.75, 0.83)	0.85 (0.81, 0.89)	0.77 (0.73, 0.81)
BD1	1.52 (1.46, 1.58)	1.73 (1.67, 1.79)	1.59 (1.53, 1.65)
	Children (under 12)		
NG1	0.29 (0.19, 0.39)	-	-
NG2	0.15 (0.05, 0.25)	-	-
NG3	0.34 (0.20, 0.48)	-	-
KE1	1.74 (1.62, 1.86)	-	-
KE2	1.30 (1.18, 1.42)	-	-
PK1	1.85 (1.73, 1.97)	-	-
BD1	1.04 (0.96, 1.12)	-	-
	Children (under 5)		
NG1	0.30 (0.14, 0.46)	-	-
NG2	0.21 (0.03, 0.39)	-	-
NG3	0.40 (0.16, 0.64)	-	-
KE1	2.57 (2.35, 2.79)	-	-
KE2	1.68 (1.50, 1.86)	-	-
PK1	2.46 (2.24, 2.68)	-	-
BD1	1.50 (1.36, 1.63)	-	-

For peer review only

For peer review only - http://bmjopen.bmj.com/site/about/guidelines.xhtml

Choice of providers of doctor and nurse consultations

Table 4 reports travel times, waiting times, and travel, drug, tests and other costs for the different types of facility for doctor and nurse consultations. Within each site the travel time to reach each type of facility was broadly similar with median travel times generally ranging from 15 to 30 minutes for all types of facility (see also Figure C1, Supplementary Information).

Bangladesh and Nigeria were the most expensive locations to seek treatment, both in relative and absolute terms, with median spending ranging from Int$ 21 to 82 depending on facility type. Median spending in Kenyan facilities ranged from Int$ 6 to 15, and in Pakistan Int$ 16 to 42. Medication costs accounted the bulk of the cost of an outpatient consultation in all sites: the median proportion of the total cost of a visit accounted for by drugs was 67% to 100%. Consultation fees in the Nigerian and Kenyan sites were generally under Int$5 and often zero, whereas in Pakistan and Bangladesh they were higher (approximately Int$5 to 15). The median expenditure for a visit was less than 10% of total monthly household expenditure for almost all types of consultation across all seven sites. However, the costs were highly skewed so that in all countries except Kenya over a third of consultations would constitute more than a third of a person's total monthly household expenditure.

Table 5 reports the results from the choice model. All elasticities were below zero, showing people prefer less costly and nearer services. However, the mean estimated elasticities were almost all between zero and minus one, with the exception of in Kenya, suggesting demand was relatively inelastic with respect to both price and time. Figure 4 compares predicted share of visits to different providers, net of costs and travel times, to actual proportions of visits: there was little qualitative difference between the two. This expressed choice is similar to survey responses; only 23-43% of respondents reported "low fees" being a reason they chose a provider (Table C4, Supplementary Information). Demand was generally more elastic in poorer households (Table 5).

For peer review only - http://bmjopen.bmj.com/site/about/guidelines.xhtml

Table 4. Summary of time and costs of outpatient doctor and nurse consultations by study site for adults and children combined. All values are median [IQR] if the number of recorded visits N was five or more.

Variable		Facility type				
		Single-handed	Clinics		Hospitals	
			Private	Public/NGO	Private	Public/NGO
NG1						
Travel time (mins)		-	15 [5, 30]	15 [10, 20]	15 [10, 30]	15 [10, 30]
Waiting time (mins)		-	5 [2, 10]	10 [5, 40]	5 [2, 10]	30 [10, 60]
Costs (Int$)	Consultation	-	0 [0, 3]	0 [0, 2]	0 [0, 8]	0 [0, 3]
	Drugs	-	17 [4, 73]	15 [8, 23]	21 [10, 42]	17 [10, 25]
	Tests	-	0 [0, 7]	1 [0, 4]	2 [0, 8]	2 [0, 8]
	Travel	-	0 [0, 4]	0 [0, 5]	2 [0, 8]	2 [0, 10]
	Total	-	42 [5, 88]	21 [13, 33]	42 [21, 61]	23 [14, 42]
Total cost as % of monthly h/h expenditure		-	9 [2, 21]	7 [3, 15]	11 [6, 19]	9 [4, 16]
Households spending >10% of monthly expenditure on visit (%)		-	43	32	56	43
NG2						
Travel time (mins)		-	20 [12, 44]	10 [5, 20]	15 [10, 30]	20 [10, 30]
Waiting time (mins)		-	30 [12, 55]	15 [5, 30]	10 [5, 30]	30 [13, 60]
Costs (Int$)	Consultation	-	0 [0, 1]	0 [0, 1]	0 [0, 0]	0 [0, 4]
	Drugs	-	5 [1, 11]	15 [8, 29]	20 [8, 42]	17 [11, 29]
	Tests	-	0 [0, 0]	0 [0, 4]	0 [0, 8]	0 [0, 8]
	Travel	-	0 [0, 0]	0 [0, 4]	0 [0, 8]	0 [0, 8]
	Total	-	13 [9, 32]	20 [13, 34]	29 [13, 78]	29 [17, 42]
Total cost as % of monthly h/h expenditure		-	11 [6, 46]	5 [3, 13]	11 [4, 23]	13 [5, 30]
Households spending >10% of monthly expenditure on visit (%)		-	50	32	56	55
NG3						
Travel time (mins)		-	22 [10, 41]	10 [9, 20]	20 [14, 30]	30 [10, 40]
Waiting time (mins)		-	10 [5, 12]	20 [5, 50]	10 [5, 30]	46 [10, 120]
Costs (Int$)	Consultation	-	0 [0, 0]	1 [0, 2]	0 [0, 8]	0 [0, 4]
	Drugs	-	25 [12, 42]	13 [8, 22]	42 [21, 49]	17 [8, 30]
	Tests	-	0 [0, 19]	0 [0, 4]	0 [0, 13]	7 [0, 17]
	Travel	-	0 [0, 0]	0 [0, 2]	2 [0, 3]	2 [0, 4]
	Total	-	56 [29, 81]	20 [11, 34]	47 [33, 81]	34 [17, 60]
Total cost as % of monthly h/h expenditure		-	10 [3, 16]	4 [2, 9]	9 [4, 15]	7 [3, 12]
Households spending >10% of monthly expenditure on visit (%)		-	50	17	43	33
KE1						
Travel time (mins)		-	10 [5, 20]	15 [10, 30]	20 [10, 30]	28 [10, 45]
Waiting time (mins)		-	30 [5, 42]	60 [18, 94]	20 [5, 42]	30 [20, 120]
Costs (Int$)	Consultation	-	0 [0, 2]	0 [0, 0]	0 [0, 2]	0 [0, 1]
	Drugs	-	9 [2, 13]	5 [2, 9]	7 [2, 18]	6 [0, 9]
	Tests	-	2 [0, 6]	0 [0, 3]	0 [0, 6]	0 [0, 6]
	Travel	-	2 [0, 5]	0 [0, 2]	2 [0, 3]	1 [0, 4]
	Total	-	13 [8, 30]	6 [2, 12]	11 [4, 30]	8 [2, 24]
Total cost as % of monthly h/h expenditure		-	5 [1, 21]	3 [1, 11]	5 [1, 11]	3 [1, 11]
Households spending >10% of monthly expenditure on visit (%)		-	43	27	29	26
KE2						
Travel time (mins)		-	18 [10, 30]	20 [15, 30]	30 [15, 50]	35 [30, 58]
Waiting time (mins)		-	10 [5, 20]	30 [10, 60]	15 [5, 30]	40 [20, 88]
Costs (Int$)	Consultation	-	0 [0, 1]	0 [0, 1]	0 [0, 3]	1 [0, 3]

For peer review only - http://bmjopen.bmj.com/site/about/guidelines.xhtml

	Drugs	-	8 [4, 14]	6 [2, 11]	9 [5, 19]	11 [7, 28]
	Tests	-	3 [0, 6]	0 [0, 2]	9 [0, 19]	6 [2, 11]
	Travel	-	1 [0, 2]	1 [0, 1]	2 [1, 4]	2 [2, 4]
	Total	-	9 [4, 19]	6 [4, 13]	13 [6, 31]	15 [4, 48]
Total cost as % of monthly h/h expenditure		-	3 [1, 9]	3 [1, 8]	4 [1, 14]	5 [1, 15]
Households spending >10% of monthly expenditure on visit (%)		-	19	21	29	31
PK1						
Travel time (mins)		10 [5, 15]	5 [5, 10]	20 [14, 30]	15 [10, 30]	30 [15, 30]
Waiting time (mins)		10 [0, 25]	10 [10, 20]	52 [12, 98]	25 [10, 60]	30 [14, 60]
Costs (Int$)	Consultation	3 [3, 5]	3 [3, 6]	0 [0, 1]	6 [1, 29]	0 [0, 0]
	Drugs	12 [6, 16]	9 [6, 19]	12 [4, 16]	16 [5, 44]	16 [0, 31]
	Tests	0 [0, 0]	0 [0, 0]	0 [0, 0]	0 [0, 0]	0 [0, 0]
	Travel	0 [0, 0]	0 [0, 0]	1 [0, 2]	2 [0, 6]	2 [0, 6]
	Total	18 [11, 25]	16 [9, 24]	17 [11, 25]	42 [18, 104]	19 [7, 55]
Total cost as % of monthly h/h expenditure		1 [1, 4]	2 [1, 3]	1 [0, 3]	2 [4, 9]	2 [0, 4]
Households spending >10% of monthly expenditure on visit (%)		6	9	8	25	12
BD1						
Travel time (mins)		20 [15, 30]	30 [20, 60]	30 [20, 30]	35 [19, 60]	30 [24, 60]
Waiting time (mins)		45 [30, 90]	60 [30, 90]	85 [30, 128]	20 [19, 30]	60 [30, 120]
Costs (Int$)	Consultation	12 [6, 15]	12 [1, 15]	0 [0, 0]	0 [0, 4]	0 [1, 4]
	Drugs	26 [15, 47]	32 [14, 61]	15 [5, 24]	27 [20, 60]	21 [9, 41]
	Tests	21 [8, 46]	31 [0, 61]	11 [1, 20]	6 [6, 6]	31 [9, 48]
	Travel	2 [2, 3]	2 [1, 3]	3 [2, 5]	2 [2, 2]	3 [2, 6]
	Total	52 [30, 89]	82 [26, 128]	22 [15, 39]	36 [32, 62]	45 [18, 96]
Total cost as % of monthly h/h expenditure		13 [6, 23]	17 [5, 32]	6 [2, 10]	11 [10, 16]	9 [3, 17]
Households spending >10% of monthly expenditure on visit (%)		58	67	28	75	44

The most frequently cited reasons for choosing a particular healthcare facility and provider were proximity (54-78% of respondents), service quality (31-95%) and cordiality (21-65%) (Table C4 Supplementary Information). Generally, the majority of respondents were either “Very satisfied” or “satisfied” with their visit and rated most aspects of the visit either “Very good” or “good”, aside from waiting time where negative responses were more common. Respondents were more likely to be “Very satisfied” with care at public clinics in four of the seven sites, with comparable satisfaction with other providers in the remaining sites. Respondents were also least likely to report “very satisfied” for single-handed facilities and private clinics. The most common medical reason for seeking care was “communicable” or “acute conditions” (35-69%) with only 2 to 6% of respondents reporting a chronic condition as the reason for the visit (see Figures C2 and C3, Supplementary Information).

For peer review only - http://bmjopen.bmj.com/site/about/guidelines.xhtml

Table 5. Price and travel time elasticities of demand and willingness to pay for nearer services for households by total monthly per person household consumption expenditure, values are posterior mean (95% credible intervals)

	**Price elasticity of demand**		**Time elasticity of demand**		**Willingness to pay for 15 minutes less travel time (Int$)**	
	<Int$100 pppm	***≥Int$100 pppm***	***<Int$100 pppm***	***≥Int$100 pppm***	***<Int$100 pppm***	***≥Int$100 pppm***
NG1	-0.62 (-0.89, -0.36)	-0.27 (-0.46, -0.10)	-1.01 (-1.76, -0.26)	-0.47 (-1.04, -0.18)	8.45 (-0.95, 22.69)	7.20 (1.94, 14.51)
NG2	-0.96 (-1.43, -0.49)	-0.47 (-0.75, -0.17)	-0.29 (-0.96, 0.42)	-0.36 (-0.88, -0.21)	1.35 (-2.07, 5.03)	3.00 (-2.06, 9.49)
NG3	-0.45 (-1.23, 0.23)	-0.03 (-0.53, 0.43)	0.00 (-0.84, 1.02)	0.00 (-0.61, 0.78)	-2.39 (-39.37, 41.45)	-2.87 (-27.97, 33.16)
KE1	-1.92 (-2.46, -1.26)	-0.62 (-1.18, -0.06)	-0.34 (-0.94, 0.22)	-0.16 (-0.63, 0.31)	1.15 (-0.39, 3.25)	1.50 (-1.23, 5.44)
KE2	-1.94 (-2.44, -1.26)	-1.49 (-1.96, -0.89)	-0.27 (-0.87, 0.42)	-0.27 (-0.81, 0.40)	1.13 (-1.08, 3.60)	1.03 (-0.86, 3.10)
PK1	-0.30 (-1.72, 1.05)	-0.69 (-1.31, -0.04)	-1.52 (-2.70, -0.32)	-0.98 (-1.61, -0.30)	6.93 (2.03, 16.04)	6.19 (2.13, 12.65)
BD1	-0.79 (-2.38, 0.72)	-0.28 (-1.63, 0.76)	-1.09 (-1.88, -0.36)	-0.21 (-0.59, 0.17)	12.20 (-46.93, 72.03)	7.03 (-18.30, 34.48)

For peer review only - http://bmjopen.bmj.com/site/about/guidelines.xhtml

DISCUSSION

We found that people living in slums make use of a range of primary care providers, both public and private, and from individual clinicians to hospital outpatient departments. While there are considerable differences between slums and countries, we found across all sites that: traditional healers were seldom consulted for health needs, reflecting previous evidence;[21] doctors and nurses were frequently consulted, more so than pharmacies in all sites other than Bangladesh; formal clinics/hospital outpatient departments were more popular than “single-handed” settings; the costs of medication exceeded those of consultations; and, demand was relatively inelastic with respect to consultancy and travel cost.

The various providers of doctor and nurse consultations were all in relative proximity to the slum precinct; the majority of respondents reported requiring less than 30 minutes to reach their provider of choice. It is noteworthy that relatively frequent use was made of hospital outpatient departments, which accounted for as much as 50% of doctor or nurse outpatient consultations in some sites. We also found low use of individually owned and operated practices. This is in contrast to what we expected, perhaps because of the substantial literature on quality of care in “single-handed” practices, much of it emanating from India.[3,6,7,22]

There was evidence that many households spent a significant proportion of their money on healthcare. While we found evidence that the up-front cost of a visit, including consultancy fees and travel costs, were relatively low and had only a small effect on demand for services, the total cost of a visit was much higher and often differed little between public and private facilities. Indeed, the consultancy fees even at private providers were often zero. The WHO’s Global Health Observatory use the proportion of households spending more than 10% of their expenditure on healthcare as a marker of national health system performance and report figures of 15%, 5%, 13%, and 25% for Nigeria, Kenya, Pakistan, and Bangladesh, respectively (Table A1, Supplementary). The relative differences between countries reflect those found in this study. In

For peer review only - http://bmjopen.bmj.com/site/about/guidelines.xhtml

both data sources, Kenya has the lowest rates of high spending relative to household spending which may be attributed to the National Hospital Insurance Fund. Nevertheless, our results suggest the proportion of slum households meeting the 10% threshold is much higher (generally more than 50%) than the above national averages.

Most of the cost of a primary care visit was accounted for by the cost of drugs, which reflects findings from another LMIC-based study that showed high mark ups on medication.[23] Kenya is the only country in our study with any public funding for medication, and while respondents there did report the lowest cost to access care of any of our sites, drugs were still the biggest source of expenditure.[24] Much of the literature on healthcare access in LMICs has focused on the deleterious effect of user and consultation fees, and it has been shown that even very small costs can suppress demand and do so non-selectively and to the detriment of children (e.g.[25]). However, our evidence suggests that the costs of treatment may be a larger problem for access to care in urban areas. Few visits resulting in spending on medical tests.

Patient choice

The finding that people will frequently attend a formal facility such as a hospital outpatient department, bypassing other facilities/providers on the way, shows that people are likely willing to trade convenience and cost for perceptions of quality. We have quantified and explored these trade-offs formally by use of a 'choice model'. An immediate limitation is that we are not 'comparing like for like' since we must assume that demand for a provider type is heavily determined by the type of symptom a person is experiencing. That said, it must also be assumed that when the situation is perceived to be more serious, the more a person will eschew local in favor of more distant providers. In that sense our findings are all the more impressive, representing an underestimate of preference for formal providers when the complaint is more serious. Nevertheless, higher elasticities among the poorest people is a cause for concern.

For peer review only - http://bmjopen.bmj.com/site/about/guidelines.xhtml

Overall demand for health care

Despite the availability of a range of providers, and study participants reporting that they were able to access health care when they needed it, age adjusted use rates in our study sites were substantially lower than in high income countries despite a high burden of disease. In Nigeria, respondents consulted a doctor or nurse once every three years to once every 6 to 9 months in Bangladesh. Yet in the United States the crude consultation rate was 3.9 visits per person-year,[26] and the United Kingdom it was 5.5 visits per person-year.[27] Not only is use in our study population lower than in HIC, but there is evidence that health needs are not being met. For instance, very few visits were reported for chronic conditions and follow-up despite the prevalence of chronic illness, which has been reported to be high in other slum populations.[28] Lack of consultation for symptoms that do not cause immediate distress may be a reason that patients presenting with cancer in LMICs are much more likely than in HICs to be in stage 3 or 4.[29,30]

Policy recommendations

Our findings, interpreted in the context of the literature, provide the basis for an emerging policy to improve access to high-quality care in urban areas. First, while in rural areas the predominant consideration is often the existence of local services, in urban areas there are a wide variety of services in close proximity. The imperative in urban areas should therefore be to make better use of the services that already exist. For example, we have found a relatively high use of pharmacies rising to two thirds of all health care contacts in Bangladesh.[11] This suggests, for example, that they might provide a good focus for dissemination of preventive health advice.

Second, our findings on expressed preference show that, in the context of the city, distance to facilities does not significantly suppress demand. These observations suggests that it might be a mistake to pursue, in urban areas, a policy to ensure yet closer location of many services to where people live. Such services would inevitably be more dispersed into small and “single-handed”

For peer review only - http://bmjopen.bmj.com/site/about/guidelines.xhtml

providers where care has been shown to be of low quality (even when the practitioners are medically qualified[6]), and that training has little effect on improving care quality.[7]

Third, overall use is low while people with serious disease such as cancer and TB present late. At the supply side this should be tackled by improving care quality, such as improved diagnosis, as suggested above and also by mitigating the main cost – namely medicines. On the demand side there is a need to continue research into barriers to appropriate health seeking for symptoms of serious disease.

Strengths and Limitations

The response rates differed by study site. We made up to three callbacks for each sampled household to minimize selection bias, but non-responsive or non-consenting households may have differed from those that participated, particularly as slums can be highly dynamic places. There only exist one longitudinal study of slum populations,[31] while it does not capture healthcare use statistics, it does suggest the material circumstances and levels of health spending have not varied significantly year-to-year[32] (the Covid pandmemic notwithstanding). Nevertheless, the findings between slums in this article are qualitatively similar in terms of behaviour and agree with other studies in this area, thus we believe these results can be used to guide policy for these populations. The age standardized consultation rates were based on the INDPETH and WHO reference populations[17] enabling us to make age-adjusted comparisons between study sites. However, the reference population may be out of date and may not be the most appropriate population for broader international comparisons. However, estimated rates differed little between reference populations. Clinical officers were not included in this study as a category, and we do not how respondents might identify or categorise them (e.g. as a “doctor” or “other”); further research is required into the role of clinical officers in these locations. Indeed, a limitation of our study was that the type of provider (nurse or doctor) was self-reported, which means the provider could be misclassified. Further research is required to determine what effect this might have for

For peer review only - http://bmjopen.bmj.com/site/about/guidelines.xhtml

healthcare use surveys like this. We cannot make a reliable comparison of individuals based on their health status as no reliable clinical or epidemiological indicators were captured. Differences between sites may well be attributable to large differences in population health, however we suggest that this would be unlikely. Any observed relationships, or lack thereof, may be driven by other underlying processes and confirmatory studies are needed to address some of the questions raised here. Finally, slum populations are often highly mobile, which could result in rapidly shifting population characteristics. The cross-sectional data here may therefore not provide a complete picture of slums around the world and we caution against making broad generalizations.

Conclusion

There have been big 'pro-poor' improvements in key indicators, such as infant and under-five mortality, in the last 20 years. But these have been largely achieved by preventive services such as immunization, improved nutrition, and rehydration therapy. Further improvements, for example in cancer care, will require improved clinical care along the pathways from seeking care for the first symptom to definitive treatment. We hope that our findings contribute to the debate about how to improve primary care services by supporting understanding of when, where, and how the residents of poorer urban areas make contact with the health system when health needs arise.

REFERENCES

1 WHO; UNICEF Declaration of Astanap.8-9. Declaration of Astana. *Glob Conf Prim Heal Care* 2018.

2 Kruk ME, Gage AD, Arsenault C, *et al.* High-quality health systems in the Sustainable Development Goals era: time for a revolution. *Lancet Glob Heal* 2018; **6**: e1196–252.

3 McPake B, Hanson K. Managing the public-private mix to achieve universal health coverage. *Lancet (London, England)* 2016; **388**: 622–30.

4 Ezeh A, Oyebode O, Satterthwaite D, *et al.* The history, geography, and sociology of slums and the health problems of people who live in slums. *Lancet* 2016; published

For peer review only - http://bmjopen.bmj.com/site/about/guidelines.xhtml

online Oct. DOI:10.1016/S0140-6736(16)31650-6.

5 Lilford RJ, Oyebode O, Satterthwaite D, *et al.* Improving the health and welfare of people who live in slums. *Lancet* 2017; **389**: 559–70.

6 Das J. The Quality of Medical Care in Low-Income Countries: From Providers to Markets. *PLoS Med* 2011; **8**: e1000432.

7 Das J, Chowdhury A, Hussam R, Banerjee A V. The impact of training informal health care providers in India: A randomized controlled trial. *Science (80-)* 2016; **354**: aaf7384–aaf7384.

8 Kruk ME, Gage AD, Joseph NT, Danaei G, García-Saisó S, Salomon JA. Mortality due to low-quality health systems in the universal health coverage era: a systematic analysis of amenable deaths in 137 countries. *Lancet* 2018; **392**: 2203–12.

9 Chen Y-F, Park J-E, Harris B, Ajisola M, Griffiths F, Improving Health in Slums Collaborative. Scoping review protocol: Factors associated with accessing health care and provision of health services for residents of slums in low and middle income countries. 2021 https://osf.io/694t2.

10 Crocker-Buque T, Mindra G, Duncan R, Mounier-Jack S. Immunization, urbanization and slums – a systematic review of factors and interventions. *BMC Public Health* 2017; **17**: 556.

11 Khan MMH, Grübner O, Krämer A. Frequently used healthcare services in urban slums of Dhaka and adjacent rural areas and their determinants. *J Public Health (Bangkok)* 2012; **34**: 261–71.

12 Owusu-Ansah FE, Tagbor H, Togbe MA. Access to health in city slum dwellers: The case of Sodom and Gomorrah in Accra, Ghana. *African J Prim Heal Care Fam Med* 2016; **8**. DOI:10.4102/phcfm.v8i1.822.

13 Improving Health in Slums Collaborative. A protocol for a multi-site, spatially-referenced household survey in slum settings: methods for access, sampling frame construction, sampling, and field data collection. *BMC Med Res Methodol* 2019; **19**: 109.

14 UN Habitat. The Challenge of the Slums: Global Report on Human Settlements 2003. London, UK, 2010.

15 Chipeta M, Terlouw D, Phiri K, Diggle P. Inhibitory geostatistical designs for spatial prediction taking account of uncertain covariance structure. *Environmetrics* 2017; **28**: e2425.

16 World Health Organization. WHO World Health Survey. 2007. https://www.who.int/healthinfo/survey/en/ (accessed March 6, 2020).

17 Ahmad OB, Boschi-pinto C, Lopez AD. Age standardization of rates: a new WHO standard. *GPE Discuss Pap Ser* 2001.

18 Sankoh O, Sharrow D, Herbst K, *et al.* The INDEPTH standard population for low- and middle-income countries, 2013. *Glob Health Action* 2014; **7**: 23286.

19 Fox JT, Kim K il, Ryan SP, Bajari P. The random coefficients logit model is identified. *J Econom* 2012; **166**: 204–12.

20 J. Borah B. A mixed logit model of health care provider choice: analysis of NSS data for

For peer review only - http://bmjopen.bmj.com/site/about/guidelines.xhtml

rural India. *Health Econ* 2006; **15**: 915–32.

21 Oyebode O, Kandala N-B, Chilton PJ, Lilford RJ. Use of traditional medicine in middle-income countries: a WHO-SAGE study. *Health Policy Plan* 2016; **31**: 984–91.

22 Coarasa J, Das J, Gummerson E, Bitton A. A systematic tale of two differing reviews: Evaluating the evidence on public and private sector quality of primary care in low and middle income countries. *Global Health* 2017. DOI:10.1186/s12992-017-0246-4.

23 Cameron A, Ewen M, Ross-Degnan D, Ball D, Laing R. Medicine prices, availability, and affordability in 36 developing and middle-income countries: a secondary analysis. *Lancet* 2009; **373**: 240–9.

24 Bitton A, Fifield J, Ratcliffe H, *et al.* Primary healthcare system performance in low-income and middle-income countries: a scoping review of the evidence from 2010 to 2017. *BMJ Glob Heal* 2019; **4**: e001551.

25 Watson SI, Wroe EB, Dunbar EL, *et al.* The impact of user fees on health services utilization and infectious disease diagnoses in Neno District, Malawi: a longitudinal, quasi-experimental study. *BMC Health Serv Res* 2016; **16**: 595.

26 OECD. Doctor's Consultations. OECD Data. 2020. https://data.oecd.org/healthcare/doctors-consultations.htm (accessed March 6, 2020).

27 Hobbs FDR, Bankhead C, Mukhtar T, *et al.* Clinical workload in UK primary care: a retrospective analysis of 100 million consultations in England, 2007-14. *Lancet (London, England)* 2016; **387**: 2323–30.

28 McNairy ML, Tymejczyk O, Rivera V, *et al.* High Burden of Non-communicable Diseases among a Young Slum Population in Haiti. *J Urban Heal* 2019; **96**: 797–812.

29 Rivera-Franco MM, Leon-Rodriguez E. Delays in Breast Cancer Detection and Treatment in Developing Countries. *Breast Cancer Basic Clin Res* 2018; **12**: 117822341775267.

30 Stewart TS, Moodley J, Walter FM. Population risk factors for late-stage presentation of cervical cancer in sub-Saharan Africa. *Cancer Epidemiol* 2018; **53**: 81–92.

31 African Population and Health Research Center. Nairobi Urban Health Demographic Surveillance System. 2018. http://aphrc.org/post/projects/nairobi-urban-health-and-demographic-surveillance-system-nuhdss (accessed Aug 3, 2018).

32 Buigut S, Ettarh R, Amendah DD. Catastrophic health expenditure and its determinants in Kenya slum communities. *Int J Equity Health* 2015; **14**: 46.

Figure legends

Figure 1. Age-adjusted (to INDEPTH population) visit rates per person-year to different outpatient care providers for adults and children (under 12) for new and existing conditions.

Figure 2. Proportion of outpatient doctor and nurse consultations for a new condition by facility type

For peer review only - http://bmjopen.bmj.com/site/about/guidelines.xhtml

Figure 3. Proportion of outpatient consultations for a new condition by doctor or nurse

Figure 4. Predicted proportions of visits to different provider types for an outpatient doctor and nurse consultation if price and travel time were all equivalent versus actual proportions of visits.

For peer review only

For peer review only - http://bmjopen.bmj.com/site/about/guidelines.xhtml

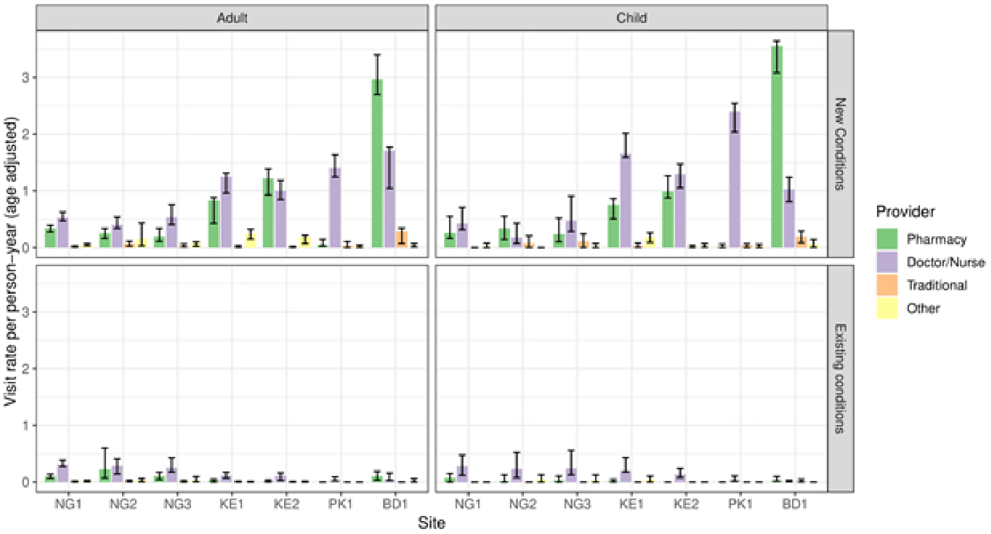

Age-adjusted (to INDEPTH population) visit rates per person-year to different outpatient care providers for adults and children (under 12) for new and existing conditions.

417x225mm (38 x 38 DPI)

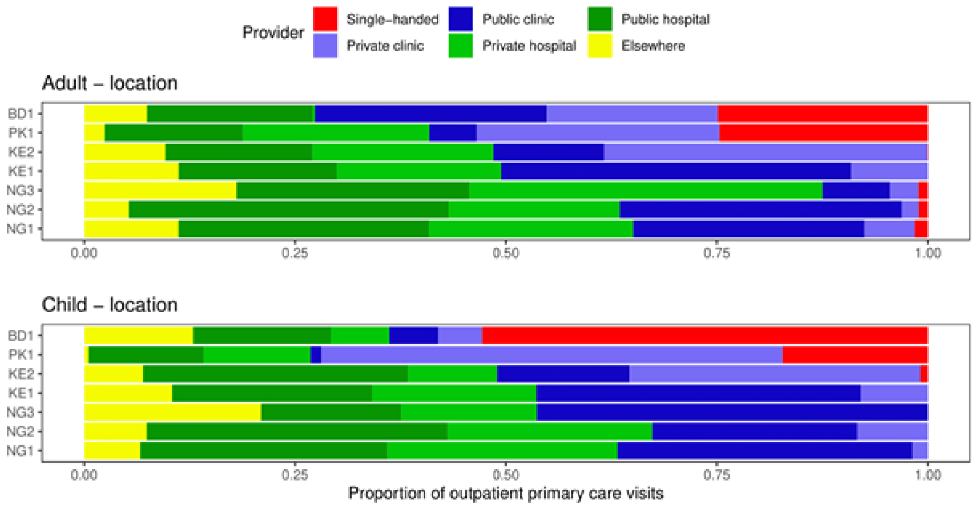

Proportion of outpatient doctor and nurse consultations for a new condition by facility type

417x214mm (38 x 38 DPI)

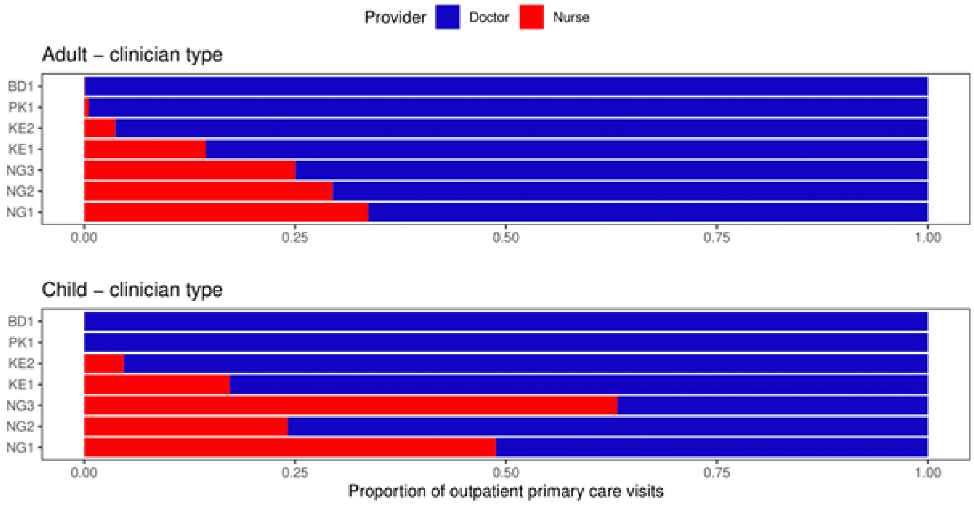

Proportion of outpatient consultations for a new condition by doctor or nurse

417x213mm (38 x 38 DPI)

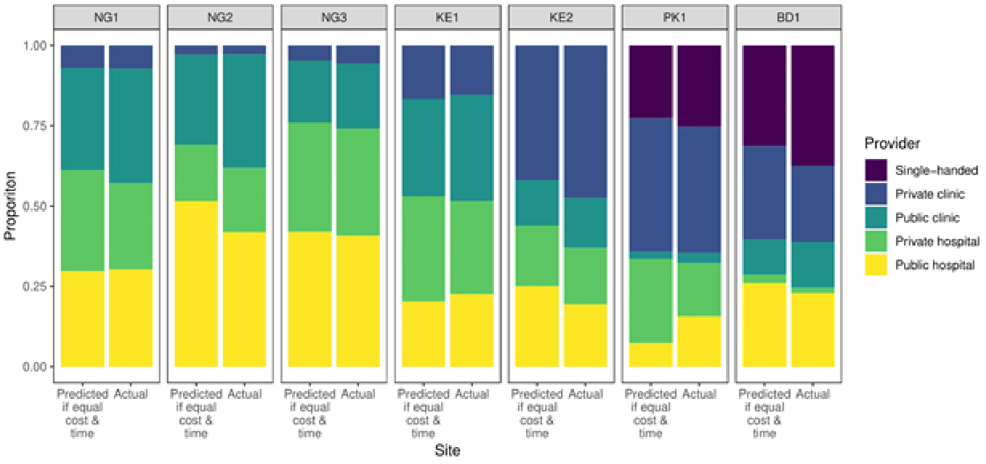

Predicted proportions of visits to different provider types for an outpatient doctor and nurse consultation if price and travel time were all equivalent versus actual proportions of visits.

417x195mm (38 x 38 DPI)

[Name of Country]
[Name of Slum]

Individual Questionnaire

[To be completed by the randomly selected adult (18+) from within the household. Note: first complete introduction and consent]

The next set of questions will ask you about the last time that you needed health care, whether or not you received that care. Please think back to the last time.

Module 4: Health Service Use

Q401	Have you needed health care in the last 12 months (whether or not you received health care)?<br><br>[INFORM THE RESPONDANT THAT THIS CAN BE INPATIENT CARE, OUTPATIENT CARE, MEDICATION, TRADITIONAL CARE, OR ANY OTHER INTERVENTION FOR THEIR HEALTH..]	1. Yes<br>2. No	 Q402<br>Q443
Q402	The last time you needed health care, did you get health care?	1. Yes……………………………………………..-><br>2. No……………………………………………..->	Q403<br>Q402
Q402b<br><br>[ONLY IF 402 = "NO"]	Which reason(s) best explains why you did not get health care?<br><br>[CHOOSE ALL THAT THE RESPONDENT INDICATES]	1. Could not afford the cost of the visit<br>2. No transport available<br>3. Could not afford the cost of transport<br>4. You were previously badly treated<br>5. Could not take the time off work or had other commitments<br>6. The health care provider's drugs or equipment were inadequate<br>7. The health care provider's skills were inadequate<br>8. You did not know where to go<br>9. You tried but were denied health care<br>10. You thought you were not sick enough<br>97. Other, please specify	

Inpatient Hospital Care

The next two questions ask about any overnight stay in a hospital or other health care facility you had in the last year.

Q403	In the last 12 months, have you ever stayed overnight in a hospital or long-term health care facility?<br>[Select all that apply]	1. Yes, a hospital<br>2. Yes, long-term care facility<br>3. Yes, other provider (Please specify)<br><br>4. No ………………………………………………… →	 <br><br><br>Q423

For peer review only - http://bmjopen.bmj.com/site/about/guidelines.xhtml

[Name of Country]
[Name of Slum]

Q404	Over the last 12 months, how many different times were you a patient in a hospital / long-term care facility for at least one night?	☐☐ times<br>98. Don't know	
		IF "00" (NO OVERNIGHT STAYS) .... →	Q423

I would like you to ask about your last inpatient stay only.

Q405	What type of hospital or facility was it? Remember we are asking now about your last (most recent) overnight stay.<br><br>[ONE ANSWER ONLY]	1. Public hospital<br>2. Private hospital<br>3. Charity or church-run hospital<br>4. Old person's home or long-term care facility<br>97. Other, please specify<br><br>[BANGLADESH: remove option 3, replace with "NGO clinical or health care facility and community health clinic"]	
Q405a	Please name the facility	Free text	
Q405b	Which reason(s) best describes why you chose this health care provider?<br>[CHOOSE ALL THAT THE RESPONDENT INDICATES]	1. Nearness of the facility<br>2. Service providers are cordial<br>3. Good service available<br>4. Short waiting time<br>5. Qualified doctors available<br>6. Low fees/low treatment cost<br>7. Good waiting arrangement<br>8. Confidentiality is maintained<br>9. Do not know where else to go<br>10. Medicine is also available<br>11. Availability of diagnostic service<br>12. Recommendation from someone<br>97. Other (please specify)	
Q405c	Which reason best describes why you were last hospitalised?<br><br>[RESPONDENT CAN SELECT ONLY ONE MAIN REASON FOR VISIT.]<br>1. Communicable disease (infections, malaria, tuberculosis, HIV)<br>2. Maternal and perinatal conditions (pregnancy)<br>3. Nutritional deficiencies<br>4. Acute conditions (diarrhoea, fever, flu, headaches, cough, other)<br>5. Injury (not work related, see 8 below)<br>6. Surgery<br>7. Sleep problems<br>8. Occupation/work related condition/injury<br>9. Chronic pain in your joints / arthritis (joints, back, neck)<br>10. Diabetes or related complications	11. Problems with your heart, including unexplained pain in chest<br>12. Problems with your mouth, teeth or swallowing<br>13. Problems with your breathing<br>14. High blood pressure / hypertension<br>15. Stroke / sudden paralysis of one side of body<br>16. Generalised pain (stomach, muscle or other non-specific pain)<br>17. Depression or anxiety<br>18. Cancer<br>97. Other, please specify	

For peer review only

For peer review only - http://bmjopen.bmj.com/site/about/guidelines.xhtml

[Name of Country]
[Name of Slum]

Q406	How did you get there?<br><br>[SELECT ALL THAT THE RESPONDENT MENTIONS]	1. Private vehicle<br>2. Public transportation<br>3. Taxicab<br>4. Ambulance or emergency vehicle<br>5. Bicycle<br>6. Walked<br>7. Boda boda, rickshaw, other private transport other than a vehicle<br>8. Don't know<br><br>[BANGLADESH: add option Rickshaw]	
Q406a	About how long did it take you to get there?	☐☐:☐☐ hours: minutes<br><br>98. Don't know	
Q406b	About how long did you wait between arrival and first consultation with a health professional?	☐☐:☐☐ hours: minutes<br><br>98. Don't know	
Q407	Who paid for this inpatient stay?<br><br>Anyone else?<br><br>[SELECT ALL RESPONSES.<br>PROBE TO SEE IF ANYONE ELSE PAID OR CONTRIBUTED TO PAYING FOR THE CARE?]	1. Respondent<br>2. Spouse / Partner<br>3. Son / Daughter<br>4. Other family member<br>5. Non-family member<br>6. Mandatory insurance scheme<br>7. Voluntary insurance scheme<br>8. Hospitalisation was free .... →	Q413
Q408	Thinking about you last inpatient stay, how much did you or your family / household members pay out-of-pocket for:<br><br>[WRITE "0" IF THE SERVICE WAS FREE – IF A PERSON DID NOT HAVE MEDICINES OR TESTS, ENTER 99990 FOR "NOT APPLICABLE, DID NOT HAVE."]<br><br>[USE LOCAL CURRENCY]	a. [Health care provider's] fees: ☐☐☐☐☐☐<br><br>b. Medicines: ☐☐☐☐☐☐<br><br>c. Tests: ☐☐☐☐☐☐<br><br>d. Transport: ☐☐☐☐☐☐<br><br>e. Other, ☐☐☐☐☐☐<br><br>please specify:	
Q409	About how much in total was paid out-of-pocket for this inpatient visit?<br><br>[USE LOCAL CURRENCY]	☐☐☐☐☐☐☐☐☐	

3

For peer review only

[Name of Country]
[Name of Slum]

Q410	Overall, how satisfied were you with the care you received during your last overnight stay?	1. Very satisfied<br>2. Satisfied<br>3. Neither satisfied nor dissatisfied<br>4. Dissatisfied<br>5. Very dissatisfied	

For your last overnight visit to a hospital or long-term care facility, how would you rate the following:		Very good	Good	Moderate	Bad	Very bad
Q411	… the amount of time you waited before being attended to?	1	2	3	4	5
Q412	… your experience of being treated respectfully?	1	2	3	4	5
Q413	… how clearly health care providers explained things to you?	1	2	3	4	5
Q414	… your experience of being involved in making decisions for your treatment?	1	2	3	4	5
Q415	… the way the health services ensured that you could talk privately to providers?	1	2	3	4	5
Q416	… the ease with which you could see a health care provider you were happy with?	1	2	3	4	5
Q417	… the cleanliness in the health facility?	1	2	3	4	5

Outpatient Care

Now I will shift away from questions about overnight stays – to questions about health care you received that did not include an overnight hospital stay. The following questions are about care you received at a hospital, health centre, clinic, pharmacy, or private office including traditional health care but where you did not stay overnight.

Q418	Over the last 12 months did you receive any health care NOT including an overnight stay?	1. Yes<br>2. No …………………………………………… →	 Q443
Q419	In total, how many times did you receive health care or consultation in the last 12 months?	☐☐ times	

Q420	What was the last (most recent) health care facility you visited in the last 12 months?<br><br>[READ OUT RESPONSES, SELECT ONE OPTION ONLY.]	1. Private doctor's office<br>2. Private clinic or health care facility<br>3. Private hospital<br>4. Public clinic or health care facility<br>5. Public hospital<br>6. Charity or church run clinic<br>7. Charity or church run hospital<br>8. Traditional Healer [USE LOCAL TERMS]<br>9. Pharmacy<br>97. Other, please specify:<br><br>[BANGLADESH: remove options 6 and 7, replace with "NGO clinic or health care facility and community health clinic"]	
Q420a	Please name the provider	Free text	

For peer review only - http://bmjopen.bmj.com/site/about/guidelines.xhtml

[Name of Country]
[Name of Slum]

Q420b	Which reason(s) best describes why you chose this health care provider?<br>[CHOOSE ALL THAT THE RESPONDENT INDICATES]	1. Nearness of the facility<br>2. Service providers are cordial<br>3. Good service available<br>4. Short waiting time<br>5. Qualified doctors available<br>6. Low fees/low treatment cost<br>7. Good waiting arrangement<br>8. Confidentiality is maintained<br>9. Do not know where else to go<br>10. Medicine is also available<br>11. Availability of diagnostic service<br>12. Recommendation from someone<br>97. Other (please vspecify)	
Q421	Which was the last (most recent) health care provider you visited?<br><br>[THE PERSON WHO PROVIDED THE CARE FOR THE MAIN REASON FOR THE VISIT]<br><br>[AFTER Q426 SUBSTITUTE THE TYPE OF HEALTH CARE PROVIDER SELECTED BY THE PATIENT WHEN YOU SEE [HEALTH CARE PROVIDER] IN BRACKETS.]	1. Medical doctor (including surgeon, gynecologist, psychiatrist, ophthalmologist…)<br>2. Nurse<br>3. Midwife<br>4. Dentist<br>5. Physiotherapist or chiropractor<br>6. Traditional medicine practitioner [USE LOCAL NAME]<br>7. Pharmacist, druggist<br>8. Don't know	
Q421a	What was the sex of the [HEALTH CARE PROVIDER]?	1. Male<br>2. Female	
Q421b	Was this visit to [HEALTH CARE PROVIDER] for a chronic (ongoing) condition, new condition, both, or routine check-up?	1. Chronic<br>2. New<br>3. Both<br>4. Routine check-up	
Q421c	Which reason best describes why you needed this visit?<br><br>[RESPONDENT CAN SELECT ONLY ONE MAIN REASON FOR VISIT.		
	1. Communicable disease (infections, malaria, tuberculosis, HIV)<br>2. Maternal and perinatal conditions (pregnancy)<br>3. Nutritional deficiencies<br>4. Acute conditions (diarrhoea, fever, flu, headaches, cough, other)<br>5. Injury (not work related, see 8 below)<br>6. Surgery<br>7. Sleep problems<br>8. Occupation/work related condition/injury<br>9. Chronic pain in your joints / arthritis (joints, back, neck)<br>10. Diabetes or related complications	11. Problems with your heart, including unexplained pain in chest<br>12. Problems with your mouth, teeth or swallowing<br>13. Problems with your breathing<br>14. High blood pressure / hypertension<br>15. Stroke / sudden paralysis of one side of body<br>16. Generalised pain (stomach, muscle or other non-specific pain)<br>17. Depression or anxiety<br>18. Cancer<br>97. Other, please specify	

For peer review only

For peer review only - http://bmjopen.bmj.com/site/about/guidelines.xhtml

[Name of Country]
[Name of Slum]

Q422	Thinking about your last visit, how did you get there?<br><br>[CIRCLE ALL THAT THE RESPONDENT MENTIONS.]	1. Private vehicle<br>2. Public transportation<br>3. Taxicab<br>4. Ambulance or emergency vehicle<br>5. Bicycle<br>6. Walked<br>8. Don't know<br><br>[BANGLADESH: add option Rickshaw]	
Q423	About how long did it take you to get there?	☐☐:☐☐ hours: minutes<br><br>98. Don't know	
Q423a	About how long did you wait between arrival and first consultation with a health professional?	☐☐:☐☐ hours: minutes<br><br>98. Don't know	
Q424	Who paid for this most recent visit?<br><br>Anyone else?<br><br>[SELECT ALL RESPONSES.<br>PROBE TO SEE IF ANYONE ELSE PAID OR CONTRIBUTED TO PAYING FOR THE CARE?]	1. Respondent<br>2. Spouse / Partner<br>3. Son / Daughter<br>4. Other family member<br>5. Non-family member<br>6. Mandatory insurance scheme<br>7. Voluntary insurance scheme	
		8. It was free ……………………………… →	Q431
Q425	Thinking about your last visit, how much did you or your family / household members pay for:<br><br>[WRITE "0" IF THE SERVICE WAS FREE – IF A PERSON DID NOT HAVE MEDICINES OR TESTS, ENTER 99998 FOR "NOT APPLICABLE, DID NOT HAVE."]<br><br>[USE LOCAL CURRENCY]	a. [HEALTH CARE PROVIDER'S] fees:<br>☐☐☐☐☐☐<br><br>b. Medicines:<br>☐☐☐☐☐☐<br><br>c. Tests:<br>☐☐☐☐☐☐<br><br>d. Transport:<br>☐☐☐☐☐☐<br><br>e. Other,<br>☐☐☐☐☐☐<br><br>please specify:	
		f. Total costs:<br>☐☐☐☐☐☐☐☐☐	

For peer review only - http://bmjopen.bmj.com/site/about/guidelines.xhtml

[Name of Country]
[Name of Slum]

Q426	Overall, how satisfied were you with the care you received during your last visit?	1. Very satisfied<br>2. Satisfied<br>3. Neither satisfied nor dissatisfied<br>4. Dissatisfied<br>5. Very dissatisfied	

For your last visit to a health care provider, how would you rate the following:		Very good	Good	Moderate	Bad	Very bad
Q427	... the amount of time you waited before being attended to?	1	2	3	4	5
Q428	... your experience of being treated respectfully?	1	2	3	4	5

For your last visit to a health care provider, how would you rate the following:		*Very good*	*Good*	*Moderate*	*Bad*	*Very bad*
Q429	... how clearly health care providers explained things to you?	1	2	3	4	5
Q430	... your experience of being involved in making decisions for your treatment?	1	2	3	4	5
Q431	... the way the health services ensured that you could talk privately to providers?	1	2	3	4	5
Q432	... the ease with which you could see a health care provider you were happy with?	1	2	3	4	5
Q433	... the cleanliness in the health facility?	1	2	3	4	5

Care at home

Now I would like to ask you to think about care you received from a health care worker or provider while at home, place of work or at an education centre. For the following questions please think about health care accessed in the past year only.

Q434	Over the last 12 months, have you received a visit in your home or place of work or education from any health care worker or provider?	1. Yes<br>2. No...................................... →<br>8. Don't know............................→	 Q444<br>Q444
Q434a	What was discussed with the health care worker?<br><br>[SELECT ALL THAT APPLY]	1. Family planning<br>2. Healthy life-style<br>3. Advice for common illness<br>4. Vaccines for pregnant women and children<br>5. Diet and nutrition<br>6. Other (specify ______________)<br>8. Don't know	
Q434b	What, if any, materials were distributed by the health care worker?	1. Family planning materials<br>2. De-worming tablets<br>3. Medicine for common cold or fever<br>4. Vitamin(s)<br>5. Calcium<br>6. Iron tablet(s)<br>7. Other (specify ______________)	

For peer review only - http://bmjopen.bmj.com/site/about/guidelines.xhtml

[Name of Country]
[Name of Slum]

		8. Don't know	

M-health

Q435	In the last 12 months have you used or attempted to use your mobile phone or other digital communication device (e.g.: laptop, tablet) to access health information, advice or care for yourself, where information about your health was received or given?	1. Yes<br>2. No.. →	Q472
Q436	Which ways of accessing health advice or information have you used?<br><br>[SELECT ALL THAT APPLY]	1. Contacted someone (via text message, chat website, e-mail, video call etc.)......................-><br>2. Researched health information without speaking to someone (via Google, health website etc.)..................................->	
Q437	Have you had any problems when using your mobile phone or other device for this purpose?	1. Yes<br>2. No .... →	Q448
Q437a	What were the problem(s):<br><br>[SELECT ALL THAT THE RESPONDENT INDICATES]	1. No airtime/data/wifi<br>2. Poor connectivity/signal<br>3. Device not sufficiently charged<br>4. No response from the person/organisation contacted<br>5. Unable to read the textual message received<br>6. Unable to find the information needed on web/social media<br>7. Unable to read the relevant text found<br>97. Other	

Now I would like to ask you to think about the last time you used or attempted to use your mobile phone or other digital communication device to access health information, advice or care for yourself, where information about your health was received or given. For the following questions please think about the most recent time only.

Q438	Which ways of accessing health or health care information did you use?<br><br>[SELECT ALL THAT APPLY.]	1. Contacted someone (via text message, chat website, e-mail, video call etc.)......................-><br>2. Researched health information without speaking to someone (via Google, health website etc.)..................................->	Q450<br><br>Q451
Q439	Who did you receive this information or what service did you use to get this information?<br><br>[REMEMBER WE ARE ASKING NOW ABOUT YOUR LAST (MOST RECENT) USE OF A DIGITAL CHANNEL FOR SEEKING HEALTH INFORMATION, ADVICE OR CARE.]	1. Medical Doctor<br>2. Nurse<br>3. Midwife<br>4. Dentist<br>5. Physiotherapist or chiropractor<br>6. Traditional medicine practitioner<br>7. Pharmacist, druggist<br>8. Users and/or provider of health related app.	
Q440	Please provide name of person/organisation/website/social media site	[Free text]	

For peer review only - http://bmjopen.bmj.com/site/about/guidelines.xhtml

[Name of Country]
[Name of Slum]

	and phone number or web address/e-mail address		
Q441	Why did you choose to get health information this way?<br><br>[QUESTION REFERS TO WHY THE RESPONDENT CHOSE TO USE M-HEALTH RATHER THAN SEEING A PROVIDER IN PERSON]<br><br>[CHOOSE ALL THAT THE RESPONDENT INDICATES]	1. I could seek the health information, advice or care at a time I chose<br>2. I could seek the health information, advice or care in a place I chose<br>3. The problem was urgent<br>4. The problem was not urgent<br>5. Service providers are cordial<br>6. I would not have to wait at a facility<br>7. I trust the provider<br>8. I needed more information than I usually receive from a health worker<br>9. I had no money to get to see a health worker<br>10. It was cheaper than going to see a health worker<br>11. I had more privacy during the communication than when seeing a health worker<br>12. I did not want other people to know I was seeking health, information advice or care<br>13. This was the only option that was available at that time	
Q442	Which reason best describes why you needed this contact?<br><br>[RESPONDENT CAN SELECT ONLY ONE MAIN REASON FOR VISIT.		
	1. Communicable disease (infections, malaria, tuberculosis, HIV)<br>2. Maternal and perinatal conditions (pregnancy)<br>3. Nutritional deficiencies<br>4. Acute conditions (diarrhoea, fever, flu, headaches, cough, other)<br>5. Injury (not work related, see 8 below)<br>6. Surgery<br>7. Sleep problems<br>8. Occupation/work related condition/injury<br>9. Chronic pain in your joints / arthritis (joints, back, neck)<br>10. Diabetes or related complications	11. Problems with your heart, including unexplained pain in chest<br>12. Problems with your mouth, teeth or swallowing<br>13. Problems with your breathing<br>14. High blood pressure / hypertension<br>15. Stroke / sudden paralysis of one side of body<br>16. Generalised pain (stomach, muscle or other non-specific pain)<br>17. Depression or anxiety<br>18. Cancer<br>97. Other, please specify	
Q443	Was there a cost associated with accessing this health advice or information?	1. Yes<br>2. No....................................... →	Q458
Q444	Thinking about the last time you last accessed health advice or information, how much did you or your family / household members pay (Do not include cost of airtime/data):	[ ][ ][ ][ ]<br><br>8. Don't know	
Q445	Overall, how <u>satisfied</u> were you with the information, advice or care you received?	1. Very satisfied<br>2. Satisfied<br>3. Neither satisfied nor dissatisfied<br>4. Dissatisfied<br>5. Very dissatisfied	

For peer review only - http://bmjopen.bmj.com/site/about/guidelines.xhtml

[Name of Country]
[Name of Slum]

Q446	Did you seek information, advice or care about the same problem from a health worker face-to-face after this digital contact?	1. Yes<br>2. No	

Now I would like you to think about your most recent contact again. I want to know your impression of your most recent contact for health information, advice or care. I would like you to rate your experiences using the following questions:

For the last time you accessed health advice or information with a phone, computer, or other electronic device, , how would you rate the following:		Very good	Good	Moderate	Bad	Very bad
FOR RESPONDENTS WHO HAVE REPORTED A CONTACTING SOMEONE						
Q447	… your experience of being treated respectfully?	1	2	3	4	5
Q448	… how clearly health care providers explained things to you?	1	2	3	4	5
Q449	… the ease with which you could make contact with a health care provider you were happy with?	1	2	3	4	5
FOR RESPONDENTS WHO HAVE REPORTED RESEARCHING HEALTH INFO						
Q450	… how understandable was the information or advice?	1	2	3	4	5

Unpaid care

Now I would like to ask you to think about the last time you provided/receive care from someone for a long-term physical or mental illness or disability or for getting old and weak. For the following questions please think about care provided or received in the past year only.

Q451	During the past year, did you provide help to a relative or friend (adult or child), because this person has a long-term physical or mental illness or disability, or is getting old and weak?	1. Yes, for a person living in the same household<br>2. Yes, for a person living in a separate household<br>3. No……………………………………… →	Q473
Q451a	Please tell me the kind of care provided:<br><br>[PLEASE SELECT ALL THAT APPLY]	1. You helped with personal care, such as going to the toilet, washing, getting dressed, or eating<br>2. You helped with medical care, like changing bandages, and giving medicines<br>3. You helped with household activities, such as meal preparation, shopping, cleaning, laundry<br>4. You watched over them since their behaviour can be upsetting or dangerous to themselves or others	
Q451b	Is/was this one-off or regular care?	1. One-off<br>2. Regular	
Q451c	Approximately how many hours a week do/did you spend providing this help?	☐☐☐☐ hours per week	

For peer review only - http://bmjopen.bmj.com/site/about/guidelines.xhtml

[Name of Country]
[Name of Slum]

Q452	During the past year, did you receive help from a relative or friend (adult or child) because you have a long-term physical or mental illness or disability or are getting old and weak?	1. Yes, from a person living in the same household<br>2. Yes, from a person living in a separate household<br>3. No………………………………………. →	Q474
Q452a	Please tell me the kind of care provided:<br><br>[PLEASE SELECT ALL THAT APPLY]	1. You were helped with personal care, such as going to the toilet, washing, getting dressed, or eating<br>2. You were helped with medical care, like changing bandages, and giving medicines<br>3. You were helped with household activities, such as meal preparation, shopping, cleaning, laundry<br>4. You were watched over since your behaviour can be upsetting or dangerous to yourself or others	
Q452b	Is/was this one-off or regular care?	1. One-off<br>2. Regular	
Q452c	Approximately how many hours a week do you receive this help?	☐☐☐☐ hours per week	

For peer review only

[Name of Country]
[Name of Slum]

Module 5: Health Status and Wellbeing

We will now as you a series of questions about your health and well-being. Please try to answer as honestly and accurately as possible. All responses will be kept in the strictest confidence.

Q501	In general would you say your health is:	1. Excellent<br>2. Very good<br>3. Good<br>4. Fair<br>5. Poor
Q502	Compared to one year ago, how would you rate your health in general now?	1. Much better than one year ago<br>2. Somewhat better now than one year ago<br>3. About the same<br>4. Somewhat worse now that one year ago<br>5. Much worse now than one year ago

Please select the response that best describes you

Q503	Physical functioning	1. Your health does not limit you in vigorous activities (such as running, lifting heavy objects)<br>2. Your health limits you a little in vigorous activities (such as running, lifting heavy objects)<br>3. Your health limits you a little in moderate activities (such as moving a table, playing with children)<br>4. Your health limits you a lot in moderate activities (such as moving a table, playing with children)<br>5. Your health limits you a little in bathing and dressing<br>6. Your health limits you a lot in bathing and dressing	
Q504	Role limitations	1. You have no problems with your work or other regular daily activities as a result of your physical health or emotional problems<br>2. You are limited in the kind of work or other activities as a result of your physical health<br>3. You accomplish less than you would like as a result of emotional problems<br>4. You are limited in the kind of work or other activities as a result of you physical health and accomplish less than you would like as a result of emotional problems	
Q505	Social functioning	Your health limits your social activities (like visiting friends, relatives, etc.):<br>1. none of the time<br>2. a little of the time<br>3. some of the time<br>4. most of the time<br>5. all of the time	
Q506	Pain	1. You have no pain<br>2. You have pain but it does not interfere with your normal work (both outside the home and housework)<br>3. You have pain that interferes with your normal work (both outside the home and housework) a little bit<br>4. You have pain that interferes with your normal work (both outside the home and housework) moderately<br>5. You have pain that interferes with your normal work (both outside the home and housework) quite a bit<br>6. You have pain that interferes with your normal work (both outside the home and housework) extremely	

For peer review only

For peer review only - http://bmjopen.bmj.com/site/about/guidelines.xhtml

[Name of Country]
[Name of Slum]

Q507	Mental health<br>You feel tense or downhearted and low:	1. None of the time<br>2. A little of the time<br>3. Some of the time<br>4. Most of the time<br>5. All of the time	
Q508	Vitality<br>You have a lot of energy:	1. None of the time<br>2. A little of the time<br>3. Some of the time<br>4. Most of the time<br>5. All of the time	

The following question asks how satisfied you feel, on a scale from 0 to 10. Zero means you feel 'not at all satisfied', and 10 means you feel 'completely satisfied'.

Q509	Overall, how satisfied are you with life as a whole these days? [0-10]	☐☐

The following question asks how worthwhile you feel the things you do in your life are, on a scale from 0 to 10. Zero means you feel the things you do in your life are 'not at all worthwhile', and 10 means 'completely worthwhile'.

Q510	Overall, to what extent do you feel the things you do in your life are worthwhile? [0-10]	☐☐

The following questions ask about how you felt yesterday on a scale from 0 to 10. Zero means you did not experience the feeling 'at all' yesterday, while 10 means you experienced the feeling 'all of the time' yesterday.

I will now read out a list of ways you might have felt yesterday.

Q511	How about happy? [0-10]	☐☐
Q512	How about worried? [0-10]	☐☐
Q513	How about depressed? [0-10]	☐☐

For peer review only - http://bmjopen.bmj.com/site/about/guidelines.xhtml

[Name of Country]
[Name of Slum]

Individual (child) Questionnaire

[THE PERSON COMPLETING THE QUESTIONNAIRE SHOULD BE THE RESPONSIBLE ADULT FOR THE RANDOMLY SELECTED CHILD]

[The child's name is referred to as {NAME}]

The next set of questions will ask you about the last time that you needed health care, whether or not you received that care. Please think back to the last time.

Module 4: Health Service Use

Q401	Has {NAME} needed health care in the last 12 months (whether or not {NAME} received health care)?<br><br>[INFORM THE RESPONDANT THAT THIS CAN BE INPATIENT CARE, OUTPATIENT CARE, MEDICATION, TRADITIONAL CARE, OR ANY OTHER INTERVENTION FOR THEIR HEALTH	1. Yes<br>2. No	Q402<br>Q443
Q402	The last time {NAME} needed health care, did {NAME} get health care?	1. Yes<br>2. No	
Q402b<br><br>[ONLY IF 402 = "NO"]	Which reason(s) best explains why {NAME} did not get health care?<br><br>[CHOOSE ALL THAT THE RESPONDENT INDICATES]	1. Could not afford the cost of the visit<br>2. No transport available<br>3. Could not afford the cost of transport<br>4. You or your child were previously badly treated<br>5. Could not take the time off work or had other commitments<br>6. The health care provider's drugs or equipment were inadequate<br>7. The health care provider's skills were inadequate<br>8. You did not know where to go<br>9. You tried but were denied health care<br>10. You thought you or your child were not sick enough<br>97. Other, please specify	

<u>Inpatient Hospital Care</u>

The next two questions ask about <u>any</u> overnight stay in a hospital or other health care facility {NAME} had in the last year.

For peer review only - http://bmjopen.bmj.com/site/about/guidelines.xhtml

[Name of Country]
[Name of Slum]

Q403	In the last 12 months, has {NAME} ever stayed overnight in a hospital, long-term care facility, or other provider?<br>[Select all that apply]	1. Yes, a hospital<br>2. Yes, long-term care facility<br>3. Yes, other provider (Please specify)	
		4. No …………………………………………… →	Q423

Q404	Over the last 12 months, how many different times was {NAME} a patient in a hospital / long-term care facility for at least one night?	☐☐ times<br>98. Don't know	
		IF "00" (NO OVERNIGHT STAYS) ……………. →	Q423

I would like you to ask about {NAME}'s last inpatient stay only.

Q405	What type of hospital or facility was it? Remember we are asking now about {NAME}'s last (most recent) overnight stay.<br><br>[ONE ANSWER ONLY]	1. Public hospital<br>2. Private hospital<br>3. Charity or church-run hospital<br>4. Old person's home or long-term care facility<br>97. Other, please specify<br><br>[BANGLADESH: remove option 3, replace with "NGO clinical or health care facility and community health clinic"]	
Q405a	Please name the facility	Free text	
Q405b	Which reason(s) best describes why you chose this health care provider?<br>[CHOOSE ALL THAT THE RESPONDENT INDICATES]	1. Nearness of the facility<br>2. Service providers are cordial<br>3. Good service available<br>4. Short waiting time<br>5. Qualified doctors available<br>6. Low fees/low treatment cost<br>7. Good waiting arrangement<br>8. Confidentiality is maintained<br>9. Do not know where else to go<br>10. Medicine is also available<br>11. Availability of diagnostic service<br>12. Recommendation from someone<br>97. Other (please specify)	
Q405c	Which reason best describes why {NAME} was last hospitalised?<br><br>[RESPONDENT CAN SELECT ONLY ONE MAIN REASON FOR VISIT. USE SHOWCARD IN APPENDIX.]		

For peer review only - http://bmjopen.bmj.com/site/about/guidelines.xhtml

[Name of Country]
[Name of Slum]

	1. Communicable disease (infections, malaria, tuberculosis, HIV)<br>2. Maternal and perinatal conditions (pregnancy)<br>3. Nutritional deficiencies<br>4. Acute conditions (diarrhoea, fever, flu, headaches, cough, other)<br>5. Injury (not work related, see 8 below)<br>6. Surgery<br>7. Sleep problems<br>8. Occupation/work related condition/injury<br>9. Chronic pain in your joints / arthritis (joints, back, neck)<br>10. Diabetes or related complications	11. Problems with your heart, including unexplained pain in chest<br>12. Problems with your mouth, teeth or swallowing<br>13. Problems with your breathing<br>14. High blood pressure / hypertension<br>15. Stroke / sudden paralysis of one side of body<br>16. Generalised pain (stomach, muscle or other non-specific pain)<br>17. Depression or anxiety<br>18. Cancer<br>97. Other, please specify	
Q406	How did you get there?<br><br>[CIRCLE ALL THAT THE RESPONDENT MENTIONS]	1. Private vehicle<br>2. Public transportation<br>3. Taxicab<br>4. Ambulance or emergency vehicle<br>5. Bicycle<br>6. Walked<br>7. Boda boda, rickshaw, other private transport other than a vehicle<br>8. Don't know<br><br>[BANGLADESH: add option Rickshaw]	
Q406a	About how long did it take you to get there?	☐☐:☐☐ hours: minutes<br><br>98. Don't know	
Q406b	About how long did you wait between arrival and first consultation with a health professional?	☐☐:☐☐ hours: minutes<br><br>98. Don't know	
Q407	Who paid for this inpatient stay?<br><br>Anyone else?<br><br>[CIRCLE ALL RESPONSES.<br>PROBE TO SEE IF ANYONE ELSE PAID OR CONTRIBUTED TO PAYING FOR THE CARE?]	1. Respondent<br>2. Spouse / Partner<br>3. Son / Daughter<br>4. Other family member<br>5. Non-family member<br>6. Mandatory insurance scheme<br>7. Voluntary insurance scheme<br>8. Hospitalisation was free .... →	Q413
Q408	Thinking about {NAME}'s last inpatient stay, how much did you or your family / household members pay out-of-pocket for:<br><br>[WRITE "0" IF THE SERVICE WAS FREE – IF A PERSON DID NOT HAVE MEDICINES OR TESTS, ENTER 99990 FOR "NOT APPLICABLE, DID NOT HAVE."]<br><br>[USE LOCAL CURRENCY]	a. [Health care provider's] fees: ☐☐☐☐☐☐<br><br>b. Medicines: ☐☐☐☐☐☐<br><br>c. Tests: ☐☐☐☐☐☐	

For peer review only - http://bmjopen.bmj.com/site/about/guidelines.xhtml

[Name of Country]
[Name of Slum]

		d. Transport: ☐☐☐☐☐☐<br>e. Other, ☐☐☐☐☐☐<br>please specify:	
Q409	About how much in total was paid out-of-pocket for this inpatient visit?<br><br>[USE LOCAL CURRENCY]	☐☐☐☐☐☐☐☐☐	
Q410	Overall, how satisfied were you with the care {NAME} received during his/her last [hospital] stay?	1. Very satisfied<br>2. Satisfied<br>3. Neither satisfied nor dissatisfied<br>4. Dissatisfied<br>5. Very dissatisfied	

For {NAME}'s last overnight visit to a hospital or long-term care facility, how would you rate the following:		Very good	Good	Moderate	Bad	Very bad
Q411	… the amount of time you waited before being attended to?	1	2	3	4	5
Q412	… your experience of being treated respectfully?	1	2	3	4	5
Q413	… how clearly health care providers explained things to you?	1	2	3	4	5
Q414	… your experience of being involved in making decisions for {NAME}'s treatment?	1	2	3	4	5
Q415	… the way the health services ensured that you could talk privately to providers?	1	2	3	4	5
Q416	… the ease with which you could see a health care provider you were happy with?	1	2	3	4	5
Q417	… the cleanliness in the health facility?	1	2	3	4	5

For peer review only

Outpatient Care

Now I will shift away from questions about overnight stays – to questions about health care {NAME} received that did not include an overnight hospital stay. The following questions are about care {NAME} received at a hospital, health centre, clinic, pharmacy, or private office including traditional health care but where {NAME} did not stay overnight.

Q418	Over the last 12 months did {NAME} receive any health care NOT including an overnight stay?	1. Yes	
		2. No ……………………………………………… →	Q443
Q419	In total, how many times did {NAME} receive health care or consultation in the last 12 months?	☐☐ times	

Now I would like you to think about the most recent visit – and ask you specifically about {NAME}'s last or most recent visit:

[Name of Country]
[Name of Slum]

Q420	What was the last (most recent) health care facility {NAME} visited in the last 12 months?<br><br>[READ OUT RESPONSES, CHOOSE ONE OPTION ONLY.]	1. Private doctor's office<br>2. Private clinic or health care facility<br>3. Private hospital<br>4. Public clinic or health care facility<br>5. Public hospital<br>6. Charity or church run clinic<br>7. Charity or church run hospital<br>8. Traditional Healer [USE LOCAL TERMS]<br>9. Pharmacy<br>97. Other, please specify:<br><br>[BANGLADESH: remove options 6 and 7, replace with "NGO clinic or health care facility and community health clinic"]	
Q420a	Please name the provider	Free text	
Q420b	Which reason(s) best describes why you chose this health care provider?<br>[CHOOSE ALL THAT THE RESPONDENT INDICATES]	1. Nearness of the facility<br>2. Service providers are cordial<br>3. Good service available<br>4. Short waiting time<br>5. Qualified doctors available<br>6. Low fees/low treatment cost<br>7. Good waiting arrangement<br>8. Confidentiality is maintained<br>9. Do not know where else to go<br>10. Medicine is also available<br>11. Availability of diagnostic service<br>12. Recommendation from someone<br>97. Other (please specify)	
Q421	Which was the last (most recent) health care provider {NAME} visited?<br><br>[THE PERSON WHO PROVIDED THE CARE FOR THE MAIN REASON FOR THE VISIT]<br><br>[AFTER Q426 SUBSTITUTE THE TYPE OF HEALTH CARE PROVIDER SELECTED BY THE PATIENT WHEN YOU SEE [HEALTH CARE PROVIDER] IN BRACKETS.]	1. Medical doctor (including surgeon, gynecologist, psychiatrist, ophthalmologist…)<br>2. Nurse / midwife<br>3. Dentist<br>4. Physiotherapist or chiropractor<br>5. Traditional medicine practitioner [USE LOCAL NAME]<br>6. Pharmacist, druggist<br>8. Don't know	
Q421a	What was the sex of the [HEALTH CARE PROVIDER]?	1. Male<br>2. Female	
Q421b	Was this visit to [HEALTH CARE PROVIDER] for a chronic (ongoing) condition, new condition, both, or routine check-up?	1. Chronic<br>2. New<br>3. Both<br>4. Routine check-up	
Q421c	Which reason best describes why {NAME} needed this visit?<br><br>[RESPONDENT CAN SELECT ONLY ONE MAIN REASON FOR VISIT.		

For peer review only - http://bmjopen.bmj.com/site/about/guidelines.xhtml

[Name of Country]
[Name of Slum]

	1. Communicable disease (infections, malaria, tuberculosis, HIV)<br>2. Maternal and perinatal conditions (pregnancy)<br>3. Nutritional deficiencies<br>4. Acute conditions (diarrhoea, fever, flu, headaches, cough, other)<br>5. Injury (not work related, see 8 below)<br>6. Surgery<br>7. Sleep problems<br>8. Occupation/work related condition/injury<br>9. Chronic pain in your joints / arthritis (joints, back, neck)<br>10. Diabetes or related complications	11. Problems with your heart, including unexplained pain in chest<br>12. Problems with your mouth, teeth or swallowing<br>13. Problems with your breathing<br>14. High blood pressure / hypertension<br>15. Stroke / sudden paralysis of one side of body<br>16. Generalised pain (stomach, muscle or other non-specific pain)<br>17. Depression or anxiety<br>18. Cancer<br>97. Other, please specify	
Q422	Thinking about {NAME}'s <u>last visit</u>, how did you get there?<br><br>[CIRCLE ALL THAT THE RESPONDENT MENTIONS.]	1. Private vehicle<br>2. Public transportation<br>3. Taxicab<br>4. Ambulance or emergency vehicle<br>5. Bicycle<br>6. Walked<br>8. Don't know<br><br>[BANGLADESH: add option Rickshaw]	
Q423	About how long did it take you to get there?	☐☐:☐☐ hours: minutes<br><br>98. Don't know	
Q423a	About how long did you wait between arrival and first consultation with a health professional?	☐☐:☐☐ hours: minutes<br><br>98. Don't know	
Q424	Who paid for this most recent visit?<br><br>Anyone else?<br><br>[SELECT ALL RESPONSES. PROBE TO SEE IF ANYONE ELSE PAID OR CONTRIBUTED TO PAYING FOR THE CARE?]	1. Respondent<br>2. Spouse / Partner<br>3. Son / Daughter<br>4. Other family member<br>5. Non-family member<br>6. Mandatory insurance scheme<br>7. Voluntary insurance scheme<br>8. It was free .... →	Q431

For peer review only - http://bmjopen.bmj.com/site/about/guidelines.xhtml

[Name of Country]
[Name of Slum]

Q425	Thinking about {NAME}'s last visit, how much did you or your family / household members pay for:<br><br>[WRITE "0" IF THE SERVICE WAS FREE – IF A PERSON DID NOT HAVE MEDICINES OR TESTS, ENTER 99998 FOR "NOT APPLICABLE, DID NOT HAVE."]<br><br>[USE LOCAL CURRENCY]	a. [HEALTH CARE PROVIDER'S] fees: ☐☐☐☐☐☐<br><br>b. Medicines: ☐☐☐☐☐☐<br><br>c. Tests: ☐☐☐☐☐☐<br><br>d. Transport: ☐☐☐☐☐☐<br><br>e. Other, ☐☐☐☐☐☐<br><br>please specify:	
		f. Total costs: ☐☐☐☐☐☐☐☐☐	
Q426	Overall, how satisfied were you with the care {NAME} received during your last visit?	1. Very satisfied<br>2. Satisfied<br>3. Neither satisfied nor dissatisfied<br>4. Dissatisfied<br>5. Very dissatisfied	

I want to know your impression of your most recent visit for health care. I would like you to rate your experiences using the following questions:

For {NAME}'s last visit to a health care provider, how would you rate the following:		Very good	Good	Moderate	Bad	Very bad
Q427	… the amount of time you waited before being attended to?	1	2	3	4	5
Q428	… your experience of being treated respectfully?	1	2	3	4	5

For {NAME}'s last visit to a health care provider, how would you rate the following:		*Very good*	*Good*	*Moderate*	*Bad*	*Very bad*
Q429	… how clearly health care providers explained things to you?	1	2	3	4	5
Q430	… your experience of being involved in making decisions for {NAME}'s treatment?	1	2	3	4	5
Q431	… the way the health services ensured that you could talk privately to providers?	1	2	3	4	5
Q432	… the ease with which you could see a health care provider you were happy with?	1	2	3	4	5
Q433	… the cleanliness in the health facility?	1	2	3	4	5

For peer review only

[Name of Country]
[Name of Slum]

Q501	In general would you say {NAME}'s health is:	1. Excellent<br>2. Very good<br>3. Good<br>4. Fair<br>5. Poor
Q502	Compared to one year ago, how would you rate {NAME}'s health in general now?	1. Much better than one year ago<br>2. Somewhat better now than one year ago<br>3. About the same<br>4. Somewhat worse now that one year ago<br>5. Much worse now than one year ago

For peer review only

For peer review only - http://bmjopen.bmj.com/site/about/guidelines.xhtml

[Name of Country]
[Name of Slum]

Household Questionnaire

[THE PERSON COMPLETING THE HOUSEHOLD QUESTIONNAIRE SHOULD BE THE HEAD OF HOUSEHOLD IF AVAILABLE. WHERE UNAVAILABLE, ANY SPOUSE OF THE HEAD OF HOUSEHOLD SHOULD BE SELECTED TO COMPLETE THE HOUSEHOLD QUESTIONNAIRE. IN THE CASE THAT NEITHER THE HEAD OF HOUSEHOLD NOR ANY SPOUSE IS AVAILABLE, PLEASE COMPLETE THE HOUSEHOLD QUESTIONNAIRE WITH ANY OTHER AVAILABLE ADULT MEMBER OF THE HOUSEHOLD, AGED 18+]

Module 1: Household Schedule

Line no.	Usual residents and visitors	Respondent	Relationship to head of household	Sex	Residence		Age	Marital status
1	Q101A	Q101B	Q101C	Q101D	Q102A	Q102B	Q103	Q104
	Please give me the names of the persons who usually live in your household and guests of the household who stayed here last night, starting with the respondent.<br><br>[WHEN THE RESPONDENT RESPONDS FOR THEMSELF, REFER TO 'YOU' OR 'YOUR']<br><br>[AFTER LISTING THE NAMES AND RECORDING THE RELATIONSHIP AND SEX FOR EACH PERSON (Q101A-C), ASK QUESTIONS **102A-C** TO BE SURE THAT THE LISTING IS COMPLETE. THEN ASK APPROPRIATE QUESTIONS 103-111 FOR EACH PERSON.]	Is this the respondent?<br>1 Yes<br>2 No	What is the relationship of [NAME] to the head of the household?<br><br>01 head; 02 spouse; 03 son or daughter; 04 son-in-law or daughter-in-law; 05 grandchild; 06 parent; 07 parent-in-law; 08 sibling; 09 other relative; 10 adopted/ foster/ stepchild; 11 not related; 98 don't know	Is [NAME] male or female?<br><br>01 Male; 02 Female	Does [NAME] usually live here?<br><br>01 Yes; 02 No	Did [NAME] sleep here last night?<br><br>01 Yes; 02 No	How old is [NAME]?<br><br>[IN YEARS; ASK TO SEE ID IF RESPONDENT UNSURE OF OWN AGE]<br><br>999 = Don't know	[IF AGED 16 OR OLDER]<br><br>What is [NAME]'s current marital status:<br>01 married or living together; 02 divorced / separated; 03 widowed; 04 Never-married and never lived together
01								
02								
03								
04								
05								
06								
07								
08								
09								
10								

For peer review only - http://bmjopen.bmj.com/site/about/guidelines.xhtml

[Name of Country]
[Name of Slum]

Line no.	Ever attended school		Current work status	Migration status				Health status
1	Q105A	Q105B	Q106	Q107A	Q107B	Q107C	Q107D	Q108
	[IF AGED 6 OR OLDER]<br><br>Has [NAME] ever attended school?<br><br>01 Yes;<br>02 No<br>08 Don't know [IF "NO" OR "DON'T KNOW" GO TO Q107]	[IF AGED 6 OR OLDER]<br><br>What is the highest level of school [NAME] has attended?<br><br>What is the highest grade [NAME] completed at that level?	Is [NAME] currently working?<br><br>01 Yes; 02 No<br><br>[DEFINE 'CURRENTLY WORKING' AS HAVING WORKED IN THE LAST WEEK IF UNSURE]	Has [NAME] always lived in this area/neighbourhood?<br><br>01 Yes; 02 No; 08 Don't know<br><br>[DEFINE AS HAVING BEEN BORN HERE IF UNSURE]<br><br>[IF "YES" GO TO Q110, IF "NO" CONTINUE WITH Q109B-D]	How long has [NAME] been living (continuously) in this area?<br><br>[□□ YEARS □□ MONTHS]	Where was [NAME] living before?<br><br>01 in the same area/ neighbourhood; 02 In another "slum" area in this country; 03 In "non-slum" urban area in this country; 04 In a rural area in this country; 05 Outside the country.<br><br>SENSITIVE TRANSLATION REQUIRED	Where has [NAME] lived for most of their life<br><br>01 in the same area/ neighbourhood; 02 In another "slum" area in this country; 03 In "non-slum" urban area in this country; 04 In a rural area in this country; 05 Outside the country.<br><br>SENSITIVE TRANSLATION REQUIRED	Does [NAME] have any physical or mental health conditions or illness lasting or expected to last for 12 months or more?<br><br>01 Yes;<br>02 No
01					□□ □□			
02					□□ □□			
03					□□ □□			
04					□□ □□			
05					□□ □□			
06					□□ □□			
07					□□ □□			
08					□□ □□			
09					□□ □□			
10					□□ □□			

For peer review only

[Name of Country]
[Name of Slum]

Health insurance status					Digital communication			
Q109A	Q109B	Q109C	Q109D	Q109E	Q110A	Q110B	Q110C	Q110D
Is [NAME] covered by any kind of insurance plan?<br><br>[IF "NO" Q111A, IF "YES" continue with Q110B-E]	What type of health insurance is (NAME) covered by?<br><br>[SELECT ALL THAT RESPONDENT INDICATES]<br><br>Mutual health organisation/ community-based health insurance;<br>Health insurance through employer;<br>Social security;<br>Other privately purchased health insurance;<br>Other, please specify	How much does your household pay for [NAME]'s health insurance each year?	Is [NAME] covered by insurance only because of his/her relationship to someone else who has health insurance?	Who is enrolled in the insurance plan that gives [NAME] health insurance?	Does [NAME] carry a mobile phone day-to-day?<br><br>01 Yes; 02 No; 08 Don't know	Is [NAME] able to use a computer, tablet or other form of digital communication other than a mobile phone, day-to-day?<br><br>01 Yes; 02 No; 08 Don't know	How do they usually access use of a mobile phone?<br><br>1. They have no access<br>2. They ask another household member to communicate for them<br>3. They borrow a phone from another member of the household when they need to use one	How do they usually access the internet?<br><br>1. They have no access<br>2. They ask another member of the household to communicate for them<br>3. They borrow a device from another member of the household when they need to use one

Q112A. Just to make sure that I have a complete listing: are there any other people such as small children or infants that we have not listed?

Q112B. Are there any other people who may not be members of your family, such as domestic servants, lodgers or friends who usually live here?

Q112C. Are there any guests or temporary visitors staying here, or anyone else who stayed here last night, who have not been listed?

For peer review only - http://bmjopen.bmj.com/site/about/guidelines.xhtml

[Name of Country]
[Name of Slum]

Module 2: Household characteristics

IN-COUNTRY TEAMS TO DELETE AS APPROPRIATE

I would like to ask you some questions about your dwelling or home. [BANGLADESH VERSION]

Q201	What is the main source of drinking water for members of your household?	1. Piped into dwelling…………………………→<br>2. Piped to yard/plot…………………………→<br>3. Public tap/standpipe<br>4. Tube well or Borehole<br>5. Dugwell: Protected well<br>6. Dugwell: Unprotected well<br>7. Protected spring<br>8. Unprotected spring<br>9. Rainwater……………………………………→<br>10. Tanker truck<br>11. Cart with small tank<br>12. Surface water (river/dam/lake/pond/stream/canal/irrigation channel)<br>13. Bottled water<br>97. Other	Q206<br>Q206<br><br><br><br><br><br><br>Q206
Q202	Where is that water source located?	1. In own dwelling……………………………………→<br>2. In own yard/plot…………………………………→<br>3. Elsewhere	Q206<br>Q206
Q203	How long does it take to go there, get water, and come back?	☐☐☐ Minutes<br>998. Don't know	
Q203A	Who usually goes to this source to fetch the water for your household	1. Adult woman<br>2. Adult man<br>3. Female child<br>4. Male child<br>5. No one person usually fetches the water	
Q204	Do you share this source with other households?	1. Yes<br>2. No…………………………………………………………→	 Q206
Q205	How many households use this source of water?	No. of households if less than 10: [0][ ]<br><br>95. 10 of more households<br>98. Don't know	
Q206	Do you do anything to the water to make it safer to drink?	1. Yes<br>2. No…………………………………………………………→<br>8. Don't know……………………………………………→	 Q207<br>Q207
Q206A	What do you usually do to make the water safer to drink?<br><br>Anything else?<br><br>[RECORD ALL MENTIONED]	1. Boil<br>2. Add bleach/chlorine<br>3. Strain through a cloth<br>4. Use water filter (ceramic/sand/composite/etc.)<br>5. Solar disinfection<br>6. Let it stand and Settle<br>97. Other	

[Name of Country]
[Name of Slum]

Q207	What kind of toilet facility do members of your household usually use?<br><br>[IF RESPONDENT INDICATES 'FLUSH' OR 'POUR FLUSH', THEN PROBE WITH:<br><br>WHERE DOES IT FLUSH TO?]	1. Flush/pour flush to piped sewer system<br>2. Flush/pour flush to septic tank<br>3. Flush/pour flush to pit latrine<br>4. Flush/pour flush to somewhere else<br>5. Flush/pour flush don't know where<br>6. Ventilated improved pit latrine<br>7. Pit latrine with slab<br>8. Pit latrine without slab/Open pit<br>9. Composting toilet<br>10. Bucket toilet<br>11. Hanging toilet/hanging latrine<br>12. No facility/bush/field……………………..→<br>97. Other	 <br><br><br><br><br><br><br><br><br><br>Q210
Q208	Do you share this toilet facility with other households?	1. Yes<br>2. No…………………………………………………→	 Q210
Q209	How many households use this toilet facility	No of household if less than 10 [0][ ]<br>95. 10 or more households<br>98. Don't know	
Q210	Does your household have:<br>[RECORD ALL THAT APPLY]	1. Electricity<br>2. Solar Electricity<br>3. Radio<br>4. Television<br>5. Mobile telephone<br>6. Non-mobile telephone<br>7. Refrigerator<br>8. Almirah/wardrobe<br>9. Electric Fan<br>10. Blu-ray/DVD/VCD Player<br>11. Water pump<br>12. IPS/Generator<br>13. Air conditioner<br>14. Computer Laptop	
Q210A	Does this household receive a cash transfer or any social assistance from the government, NGO's, CBO's, Churches or Other organizations?<br><br>[DEFINE AS HAVE RECEIVED IN THE LAST MONTH IF UNSURE]	1. Yes<br>1. No……………………………………………….→	 Q211
Q210B	For what reason does the household receive a cash transfer or social assistance?<br><br>Any other reason?<br><br>[RECORD ALL MENTIONED]	1. Orphaned children 18 years or younger<br>2. Elderly person<br>3. Person with severe disability<br>4. Urban food subsidy<br>5. Food aid for person in arid and semi-arid lands<br>6. Health voucher<br>7. Food/cash for work<br>8. School feeding<br>9. Hunger safety net programme<br>2. Other	

For peer review only - http://bmjopen.bmj.com/site/about/guidelines.xhtml

[Name of Country]
[Name of Slum]

Q211	What type of fuel does your household mainly use for cooking?	3. Electricity<br>4. LPG<br>5. Natural Gas<br>6. Biogas<br>7. Kerosene<br>8. Coal, lignite<br>9. Charcoal<br>10. Wood<br>11. Straw/shrubs/grass<br>12. Agricultural crop<br>13. Animal dung<br>14. No food cooked in household………………→<br>97. Other	 <br><br><br><br><br><br><br><br><br><br>Q215
Q212	Where is cooking usually done?	1. In a room used for living or sleeping<br>2. In a separate room used as kitchen<br>3. In a separate building used as kitchen<br>4. Outdoor<br>7. Other, please specify:	
Q213	[MAIN MATERIAL OF THE FLOOR- RECORD OBSERVATION]	1. Natural floor (earth/sand)<br>2. Rudimentary floor: wood planks<br>3. Rudimentary floor: palm/bamboo<br>4. Finished floor: Parquet or polished wood<br>5. Finished floor: ceramic tiles<br>6. Finished floor: Cement<br>7. Finished floor: Carpet<br>97. Other	
Q214	[MAIN MATERIAL OF THE ROOF- RECORD OBSERVATION]	1. Natural roof: no roof<br>2. Natural roof: thatch/palm leaf<br>3. Rudimentary roof: palm/bamboo<br>4. Rudimentary roof: wood planks<br>5. Rudimentary roof: cardboard<br>6. Finished roof: tin<br>7. Finished roof: wood<br>8. Finished roof: ceramic tiles<br>9. Finished roof: cement<br>10. Finished roof: roofing shingles<br>97. Other	
Q215	[MAIN MATERIAL OF THE EXTERIOR WALLS-RECORD OBSERVATION]	1. Natural walls: no walls<br>2. Natural walls: cane/palm/trunks<br>3. Natural walls: dirt<br>4. Rudimentary walls: bamboo with mud<br>5. Rudimentary walls: stone with mud<br>6. Rudimentary walls: plywood<br>7. Rudimentary walls: cardboard<br>8. Finished walls: tin<br>9. Finished walls: cement<br>10. Finished walls: stone with lime/cement<br>11. Finished walls: brick<br>12. Finished walls: woodplanks/shingles<br>97. Other	
Q216	How many rooms in this household are used for sleeping?	☐☐ Rooms	

[Name of Country]
[Name of Slum]

Q217	Does any member of this household own:	1. A car/truck/microbus<br>2. An autobike/tempo/CNG<br>3. A rickshaw/van<br>4. A bicycle<br>5. A motorcycle or motor scooter	
Q217A	Does your household own this structure (house, flat, shack)?	1. Yes<br>2. No	Q217B<br>Q217C
Q217B	Does your household own the land on which the structure (house, flat, shack) sits?	1. Yes<br>2. No	
Q217C	Do you pay rent?	1. Pays rent/lease<br>2. No rent with consent of owner<br>3. No rent: squatting	
Q218	Does your household own any homestead?	4. Yes<br>5. No	
Q218AA	Does your household own any land (other than the homestead land)?	1. Yes<br>2. No……………………………………… →	Q222
Q219	Does this household own any livestock, herds, other farm animals or poultry?	1. Yes<br>2. No……………………………………….→	Q224
Q220	How many of the following animals does this household own?<br><br>Buffaloes?<br>Cows?<br>Goats or sheep?<br>Chickens or ducks?<br>Other farm animals?		
Q221	Does any member of this household have a bank account?	1. Yes<br>2. No	
Q222	In the past 7 days were there days when your household did not have enough food or money to buy food?	1. Yes<br>2. No	
Q223	How many days in the month do you have air time (for calls and SMS) for at least one mobile phone in the household	1. Everyday<br>2. Almost every day (over 21 days a month but not every day)<br>3. More than half the days (over 14 but not as many as 21)<br>4. More than 7 days but less than 14<br>5. Less than 7 days<br>6. Never	
Q224	How many days in the month does someone in the household have data or access to wifi (for accessing internet for searching the web, using social media or using Email) for at least one of your digital communication devices in the household (smart phone, laptop, tablet)?	1. Everyday<br>2. Almost every day (over 21 days a month but not every day)<br>3. More than half the days (over 14 but not as many as 21)<br>4. More than 7 days but less than 14<br>Less than 7 days<br>5. Never	

For peer review only - http://bmjopen.bmj.com/site/about/guidelines.xhtml

[Name of Country]
[Name of Slum]

I would like to ask you some questions about your dwelling or home. KENYA VERSION

Q201	What is the main source of drinking water for members of your household?	1. Piped into dwelling……………………→<br>2. Piped to yard/plot……………………→<br>3. Public tap/standpipe<br>4. Tube well or Borehole<br>5. Dugwell: Protected well<br>6. Dugwell: Unprotected well<br>7. Protected spring<br>8. Unprotected spring<br>9. Rainwater………………………………→<br>10. Tanker truck<br>11. Cart with small tank<br>12. Surface water (river/dam/lake/pond/stream/ canal/irrigation channel)<br>13. Bottled water<br>97. Other	Q206<br>Q206<br><br><br><br><br><br><br>Q206
Q202	Where is that water source located?	1. In own dwelling………………………………→<br>2. In own yard/plot……………………………..→<br>3. Elsewhere	Q206<br>Q206
Q203	How long does it take to go there, get water, and come back?	☐☐☐ Minutes<br>998. Don't know	
Q203A	Who usually goes to this source to fetch the water for your household	1. Adult woman<br>2. Adult man<br>3. Female child<br>4. Male child<br>5. No one person usually fetches the water	
Q204	Do you share this source with other households?	1. Yes<br>2. No…………………………………………….→	 Q206
Q205	How many households use this source of water?	No. of households if less than 10: 0☐<br><br>95. 10 of more households<br>98. Don't know	
Q206	Do you do anything to the water to make it safer to drink?	1. Yes<br>2. No……………………………………………………→<br>8. Don't know…………………………………………→	 Q207<br>Q207
Q206A	What do you usually do to make the water safer to drink?<br><br>Anything else?<br><br>[RECORD ALL MENTIONED]	1. Boil<br>2. Add bleach/chlorine<br>3. Strain through a cloth<br>4. Use water filter (ceramic/sand/composite/etc.)<br>5. Solar disinfection<br>6. Let it stand and Settle<br>7. Cover the water container<br>97. Other	
Q207	What kind of toilet facility do members of your household usually use?	1. Flush/pour flush to piped sewer system<br>2. Flush/pour flush to septic tank<br>3. Flush/pour flush to pit latrine	

[Name of Country]
[Name of Slum]

	[IF RESPONDENT INDICATES 'FLUSH' OR 'POUR FLUSH', THEN PROBE WITH: WHERE DOES IT FLUSH TO?]	4. Flush/pour flush to somewhere else<br>5. Flush/pour flush don't know where<br>6. Ventilated improved pit latrine<br>7. Pit latrine with slab<br>8. Pit latrine without slab/Open pit<br>9. Composting toilet<br>10. Bucket toilet<br>11. Hanging toilet/hanging latrine<br>12. No facility/bush/field……………………..→<br>97. Other	Q210
Q208	Do you share this toilet facility with other households?	1. Yes<br>2. No……………………………………………→	Q210
Q209	How many households use this toilet facility	No of household if less than 10 [0][ ]<br>95. 10 or more households<br>98. Don't know	
Q210	Does your household have:<br>[RECORD ALL THAT APPLY]	1. Electricity<br>2. Radio<br>3. Television<br>4. Mobile telephone<br>5. Non-mobile telephone<br>6. Refrigerator<br>7. Solar panel<br>8. Table<br>9. Chair<br>10. Sofa<br>11. Bed<br>12. Cupboard<br>13. Clock<br>14. Microwave oven<br>15. Blu-ray/VCD/DVD Player<br>16. Cassette/CD Player	
Q210A	Does this household receive a cash transfer or any social assistance from the government, NGOs, CBOs, Churches or other organisations?<br><br>[DEFINE AS HAVE RECEIVED IN THE LAST MONTH IF UNSURE]	1. Yes<br>2. No……………………………………………→	Q211
Q210B	For what reason does the household receive a cash transfer or social assistance?<br><br>Any other reason?<br><br>[RECORD ALL MENTIONED]	1. Orphaned children 18 years or younger<br>2. Elderly person<br>3. Person with severe disability<br>4. Urban food subsidy<br>5. Food aid for person in arid and semi-arid lands<br>6. Health voucher<br>7. Food/cash for work<br>8. School feeding<br>9. Hunger safety net programme<br>97. Other	

For peer review only - http://bmjopen.bmj.com/site/about/guidelines.xhtml

[Name of Country]
[Name of Slum]

Q211	What type of fuel does your household mainly use for cooking?	1. Electricity<br>2. LPG/Natural gas<br>3. Biogas<br>4. Parafin/Kerosene<br>5. Coal, lignite<br>6. Charcoal<br>7. Wood<br>8. Straw/shrubs/grass<br>9. Agricultural crop<br>10. Animal dung<br>11. No food cooked in household……………→<br>97. Other	Q215
Q212	Where is cooking usually done?	1. In a room used for living or sleeping<br>2. In a separate room used as kitchen<br>3. In a separate building used as kitchen<br>4. Outdoor<br>97. Other, please specify:	
Q213	[MAIN MATERIAL OF THE FLOOR- RECORD OBSERVATION]	1. Natural floor: earth/sand<br>2. Natural floor: dung<br>3. Rudimentary floor: wood planks<br>4. Rudimentary floor: palm/bamboo<br>5. Finished floor: Parquet or polished wood<br>6. Finished floor: vinyl or asphalt strips<br>7. Finished floor: ceramic tiles<br>8. Finished floor: Cement<br>9. Finished floor: Carpet<br>97. Other	
Q214	[MAIN MATERIAL OF THE ROOF- RECORD OBSERVATION]	1. Natural roof: no roof<br>2. Natural roof: thatch/grass/makuti<br>3. Natural roof: dung/mud/sod<br>4. Rudimentary roof: iron sheets<br>5. Rudimentary roof: tin cans<br>6. Finished roof: asbestos sheet<br>7. Finished roof: concrete<br>8. Finished roof: tiles<br>97. Other	
Q215	[MAIN MATERIAL OF THE EXTERIOR WALLS- RECORD OBSERVATION]	1. Natural walls: no walls<br>2. Natural walls: cane/palm/trunks<br>3. Natural walls: dung/mud/sod<br>4. Rudimentary walls: bamboo with mud<br>5. Rudimentary walls: stone with mud<br>6. Rudimentary walls: uncovered adobe<br>7. Rudimentary walls: plywood<br>8. Rudimentary walls: cardboard<br>9. Rudimentary walls: reused wood<br>10. Rudimentary walls: iron sheets<br>11. Finished walls: cement<br>12. Finished walls: stone with lime/cement<br>13. Finished walls: brick<br>14. Finished walls: cement blocks<br>15. Finished walls: covered adobe<br>16. Finished walls: wood planks/shingles	

For peer review only - http://bmjopen.bmj.com/site/about/guidelines.xhtml

[Name of Country]
[Name of Slum]

		97. Other	
Q216	How many rooms in this household are used for sleeping?	☐☐ Rooms	
Q217	Does any member of this household own:	1. A watch<br>2. A bicycle<br>3. A motorcycle/scooter<br>4. An animal-drawn cart<br>5. A car/truck<br>6. A boat with a motor	
Q217A	Does your household own this structure (house, flat, shack)?	1. Yes<br>2. No	Q217B<br>Q217C
Q217B	Does your household own the land on which the structure (house, flat, shack) sits?	1. Yes<br>2. No	Q218<br>Q218
Q217C	Does your household pay rent?	6. Pays rent/lease<br>7. No rent with consent of owner<br>1. No rent: squatting	
Q218	Does any member of this household own any agricultural land?	2. Yes<br>3. No…………………………………… →	Q222
Q219	Does this household own any livestock, herds, other farm animals or poultry?	1. Yes<br>2. No…………………………………… →	Q224
Q220	How many of the following animals does this household own?<br><br>Local cattle (indigenous)<br>Exotic/grade cattle<br>Horses, donkeys, or camels?<br>Goats or sheep?<br>Pigs?<br>Chickens?<br>Other?	☐☐<br>☐☐<br>☐☐<br>☐☐<br>☐☐<br>☐☐	
Q221	Does any member of this household have a bank account?	1. Yes<br>2. No	
Q222	In the past 7 days were there days when your household did not have enough food or money to buy food?	1. Yes<br>2. No	
Q223	How many days in the month do you have air time (for calls and SMS) for at least one mobile phone in the household	1. Everyday<br>2. Almost every day (over 21 days a month but not every day)<br>3. More than half the days (over 14 but not as many as 21)<br>4. More than 7 days but less than 14<br>5. Less than 7 days<br>6. Never	
Q224	How many days in the month does someone in the household have data or access to wifi (for accessing internet for searching the web, using social media or using Email) for at least one of your digital communication devices I the household (smart phone, laptop, tablet)?	1. Everyday<br>2. Almost every day (over 21 days a month but not every day)<br>3. More than half the days (over 14 but not as many as 21)<br>4. More than 7 days but less than 14	

For peer review only - http://bmjopen.bmj.com/site/about/guidelines.xhtml

[Name of Country]
[Name of Slum]

		5. Less than 7 days<br>6. Never	

I would like to ask you some questions about your dwelling or home. NIGERIA VERSION

Q201	What is the main source of drinking water for members of your household?	1. Piped into dwelling……………………→<br>2. Piped to yard/plot……………………→<br>3. Public tap/standpipe<br>4. Tube well or Borehole<br>5. Dugwell: Protected well<br>6. Dugwell: Unprotected well<br>7. Protected spring<br>8. Unprotected spring<br>9. Rainwater……………………………→<br>10. Tanker truck<br>11. Cart with small tank<br>12. Surface water (river/dam/lake/pond/stream/ canal/irrigation channel)<br>13. Bottled water<br>97. Other	Q206<br>Q206<br><br><br><br><br><br><br>Q206
Q202	Where is that water source located?	1. In own dwelling……………………………→<br>2. In own yard/plot……………………………→<br>3. Elsewhere	Q206<br>Q206
Q203	How long does it take to go there, get water, and come back?	☐☐☐ Minutes<br>998. Don't know	
Q203A	Who usually goes to this source to fetch the water for your household	1. Adult woman<br>2. Adult man<br>3. Female child<br>4. Male child<br>5. No one person usually fetches the water	
Q204	Do you share this source with other households?	1. Yes<br>2. No……………………………………→	 Q206
Q205	How many households use this source of water?	No. of households if less than 10: 0☐<br><br>95. 10 of more households<br>98. Don't know	
Q206	Do you do anything to the water to make it safer to drink?	1. Yes<br>2. No……………………………………………→<br>8. Don't know……………………………………→	 Q207<br>Q207
Q206A	What do you usually do to make the water safer to drink?<br><br>Anything else?<br><br>[RECORD ALL MENTIONED]	1. Boil<br>2. Add bleach/chlorine<br>3. Strain through a cloth<br>4. Use water filter (ceramic/sand/composite/etc.)<br>5. Solar disinfection<br>6. Let it stand and Settle<br>7. Alum<br>97. Other	

[Name of Country]
[Name of Slum]

Q207	What kind of toilet facility do members of your household usually use?<br><br>[IF RESPONDENT INDICATES 'FLUSH' OR 'POUR FLUSH', THEN PROBE WITH:<br><br>WHERE DOES IT FLUSH TO?]	1. Flush/pour flush to piped sewer system<br>2. Flush/pour flush to septic tank<br>3. Flush/pour flush to pit latrine<br>4. Flush/pour flush to somewhere else<br>5. Flush/pour flush don't know where<br>6. Ventilated improved pit latrine<br>7. Pit latrine with slab<br>8. Pit latrine without slab/Open pit<br>9. Composting toilet<br>10. Bucket toilet<br>11. Hanging toilet/hanging latrine<br>12. No facility/bush/field……………………→<br>97. Other	 <br><br><br><br><br><br><br><br><br><br>Q210
Q208	Do you share this toilet facility with other households?	1. Yes<br>2. No……………………………………………→	 Q210
Q209	How many households use this toilet facility	No of household if less than 10 [0][ ]<br>95. 10 or more households<br>98. Don't know	
Q210	Does your household have:<br>[RECORD ALL THAT APPLY]	1. Electricity<br>2. Radio<br>3. Television<br>4. Mobile telephone<br>5. Non-mobile telephone<br>6. Refrigerator<br>7. Cable TV<br>8. Generating Set<br>9. Air conditioner<br>10. Computer<br>11. Electric Iron<br>12. Fan	
Q210A	Does this household receive a cash transfer or any social assistance from the government, NGOs, CBOs, churches or other organizations?<br><br>[DEFINE AS HAVING RECEIVED IN THE LAST MONTH IF UNSURE]	1. Yes<br>2. No…………………………………………….→	 Q211
Q210B	For what reason does the household receive a cash transfer or social assistance?<br><br>Any other reason?<br><br>[RECORD ALL MENTIONED]	1. Orphaned children 18 years or younger<br>2. Elderly person<br>3. Person with severe disability<br>4. Urban food subsidy<br>5. Food aid for person in arid and semi-arid lands<br>6. Health voucher<br>7. Food/cash for work<br>8. School feeding<br>9. Hunger safety net programme<br>97. Other	
Q211	What type of fuel does your household mainly use for cooking?	1. Electricity<br>2. LPG<br>3. Natural gas<br>4. Biogas	

[Name of Country]
[Name of Slum]

		5. Kerosene<br>6. Coal, lignite<br>7. Charcoal<br>8. Wood<br>9. Straw/shrubs/grass<br>10. Agricultural crop<br>11. Animal dung<br>12. No food cooked in household………→<br>97. Other	Q215
Q212	Where is cooking usually done?	1. In a room used for living or sleeping<br>2. In a separate room used as kitchen<br>3. In a separate building used as kitchen<br>4. Outdoors<br>97. Other, please specify:	
Q213	[MAIN MATERIAL OF THE FLOOR- RECORD OBSERVATION]	1. Natural floor: earth/sand<br>2. Natural floor: dung<br>3. Rudimentary floor: wood planks<br>4. Rudimentary floor: palm/bamboo<br>5. Finished floor: parquet or polished wood<br>6. Finished floor: vinyl or asphalt strips<br>7. Finished floor: ceramic tiles<br>8. Finished floor: cement<br>9. Finished floor: carpet/rug<br>97. Other	
Q214	[MAIN MATERIAL OF THE ROOF- RECORD OBSERVATION]	1. Natural roof: no roof<br>2. Natural roof: thatch/palm leaf<br>3. Rudimentary roof: rustic mat<br>4. Rudimentary roof: palm/bamboo<br>5. Rudimentary roof: wood planks<br>6. Rudimentary roof: cardboard<br>7. Finished roof: metal/zinc<br>8. Finished roof: wood<br>9. Finished roof: ceramic tiles<br>10. Finished roof: cement<br>11. Finished roof: roofing shingles<br>97. Other	
Q215	[MAIN MATERIAL OF THE EXTERIOR WALLS- RECORD OBSERVATION]	1. Natural walls: no walls<br>2. Natural walls: cane/palm/trunks<br>3. Natural walls: dirt (mud)<br>4. Rudimentary walls: bamboo with mud<br>5. Rudimentary walls: stone with mud<br>6. Rudimentary walls: plywood<br>7. Rudimentary walls: cardboard<br>8. Rudimentary walls: reused wood<br>9. Finished walls: cement<br>10. Finished walls: stone with lime/cement<br>11. Finished walls: bricks<br>12. Finished walls: cement blocks<br>13. Finished walls: wood planks/shingles<br>97. Other	
Q216	How many rooms in this household are used for sleeping?	☐☐ Rooms (Sleeping)	

For peer review only - http://bmjopen.bmj.com/site/about/guidelines.xhtml

[Name of Country]
[Name of Slum]

Q216A	How many rooms in total are in your household, including rooms for sleeping and all other rooms?	☐☐ Rooms (Total)	
Q217	Does any member of this household own:<br><br>[RECORD ALL THAT APPLY]	1. A watch<br>2. A bicycle<br>3. A motorcycle or motor scooter<br>4. An animal-drawn cart<br>5. A car or truck<br>6. A boat with a motor<br>7. A canoe	
Q217A	Does your household own this structure (house, flat, shack)?	1. Yes<br>2. No	Q217B<br>Q217C
Q217B	Does your household own the land on which the structure (house, flat, shack) sits?	1. Yes<br>2. No	Q218<br>Q218
Q217C	Do you pay rent?	8. Pays rent/lease<br>9. No rent with consent of owner<br>1. No rent: squatting	
Q218	Does any member of this household own any agricultural land?	2. Yes<br>3. No…………………………………. →	Q222
Q219	Does this household own any livestock, herds, other farm animals or poultry?	1. Yes<br>2. No…………………………………. →	Q224
Q220	How many of the following animals does this household own?<br><br>Milk cows or bulls?<br>Horses, donkeys, or mules?<br>Goats?<br>Sheep?<br>Chickens/ducks?<br>Pigs?<br>Other farm animals?		
Q221	Does any member of this household have a bank account?	1. Yes<br>2. No	
Q222	In the past 7 days were there days when your household did not have enough food or money to buy food?	1. Yes<br>2. No	
Q223	How many days in the month do you have air time (for calls and SMS) for at least one mobile phone in the household	1. Everyday<br>2. Almost every day (over 21 days a month but not every day)<br>3. More than half the days (over 14 but not as many as 21)<br>4. More than 7 days but less than 14<br>5. Less than 7 days<br>6. Never	
Q224	How many days in the month does someone in the household have data or access to wifi (for accessing internet for searching the web, using social media or using Email) for at least	1. Everyday<br>2. Almost every day (over 21 days a month but not every day)	

For peer review only - http://bmjopen.bmj.com/site/about/guidelines.xhtml

[Name of Country]
[Name of Slum]

	one of your digital communication devices I the household (smart phone, laptop, tablet)?	3. More than half the days (over 14 but not as many as 21)<br>4. More than 7 days but less than 14<br>5. Less than 7 days<br>6. Never	

I would like to ask you some questions about your dwelling or home. PAKISTAN VERSION

Q201	What is the main source of drinking water for members of your household?	1. Piped into dwelling……………………→<br>2. Piped to yard/plot……………………→<br>3. Public tap/standpipe<br>4. Tube well or Borehole<br>5. Handpump<br>6. Dugwell: Protected well<br>7. Dugwell: Unprotected well<br>8. Protected spring<br>9. Unprotected spring<br>10. Rainwater……………………………→<br>11. Tanker truck<br>12. Filtration plant<br>13. Cart with small tank<br>14. Surface water (river/dam/lake/pond/stream/ canal/irrigation channel)<br>15. Bottled water<br>97. Other	Q206<br>Q206<br><br><br><br><br><br><br><br>Q206
Q202	Where is that water source located?	1. In own dwelling……………………………→<br>2. In own yard/plot……………………………→<br>3. Elsewhere	Q206<br>Q206
Q203	How long does it take to go there, get water, and come back?	☐☐☐ Minutes<br>998. Don't know	
Q203A	Who usually goes to this source to fetch the water for your household	1. Adult woman<br>2. Adult man<br>3. Female child<br>4. Male child<br>5. No one person usually fetches the water	
Q204	Do you share this source with other households?	1. Yes<br>2. No……………………………………→	 Q206
Q205	How many households use this source of water?	No. of households if less than 10: 0 ☐<br><br>95. 10 of more households<br>98. Don't know	
Q206	Do you do anything to the water to make it safer to drink?	1. Yes<br>2. No……………………………………………→<br>8. Don't know…………………………………→	 Q207<br>Q207
Q206A	What do you usually do to make the water safer to drink?<br><br>Anything else?	1. Boil<br>2. Add bleach/chlorine<br>3. Strain through a cloth	

[Name of Country]
[Name of Slum]

	[RECORD ALL MENTIONED]	4. Use water filter (ceramic/sand/composite/etc.)<br>5. Solar disinfection<br>6. Let it stand and Settle<br>97. Other	
Q207	What kind of toilet facility do members of your household usually use?<br><br>[IF RESPONDENT INDICATES 'FLUSH' OR 'POUR FLUSH', THEN PROBE WITH:<br><br>WHERE DOES IT FLUSH TO?]	1. Flush/pour flush to piped sewer system<br>2. Flush/pour flush to septic tank<br>3. Flush/pour flush to pit latrine<br>4. Flush/pour flush to somewhere else<br>5. Flush/pour flush don't know where<br>6. Ventilated improved pit latrine<br>7. Pit latrine with slab<br>8. Pit latrine without slab/Open pit<br>9. Bucket toilet<br>10. Hanging toilet/hanging latrine<br>11. No facility/bush/field………………….….→<br>97. Other	Q210
Q208	Do you share this toilet facility with other households?	1. Yes<br>2. No…………………………………….→	Q210
Q209	How many households use this toilet facility	No of household if less than 10 [0][ ]<br>95. 10 or more households<br>98. Don't know	
Q210	Does your household have:<br>[RECORD ALL THAT APPLY]	1. Electricity<br>2. Radio<br>3. Television<br>4. Landline telephone<br>5. Refrigerator<br>6. Almirah/cabinet<br>7. Chair<br>8. Room cooler<br>9. Air conditioner<br>10. Washing machine<br>11. Water pump<br>12. Bed<br>13. Clock<br>14. Sofa<br>15. Camera<br>16. Sewing machine<br>17. Computer<br>18. Internet connection	
Q210A	Does this household receive a cash transfer or any social assistance from the government, NGOs, CBOs, churches or other organizations?<br><br>[DEFINE AS HAVING RECEIVED IN THE LAST MONTH IF UNSURE]	1. Yes<br>2. No……………………………………….→	Q211
Q210B	For what reason does the household receive a cash transfer or social assistance?<br><br>Any other reason?<br><br>[RECORD ALL MENTIONED]	98. Orphaned children 18 years or younger<br>99. Elderly person<br>100. Person with severe disability<br>101. Urban food subsidy	

[Name of Country]
[Name of Slum]

		102. Food aid for person in arid and semi-arid lands<br>103. Health voucher<br>104. Food/cash for work<br>105. School feeding<br>106. Hunger safety net programme<br>3. Other	
Q211	What type of fuel does your household mainly use for cooking?	1. Electricity<br>2. LPG<br>3. Natural gas<br>4. Biogas<br>5. Kerosene<br>6. Coal, lignite<br>7. Charcoal<br>8. Wood<br>9. Straw/shrubs/grass<br>10. Animal dung<br>11. No food cooked in household……….→<br>97. Other	Q215
Q212	Where is cooking usually done?	1. In a room used for living or sleeping<br>2. In a separate room used as kitchen<br>3. In a separate building used as kitchen<br>4. Outdoors<br>97. Other, please specify:	
Q213	[MAIN MATERIAL OF THE FLOOR- RECORD OBSERVATION]	1. Natural floor: earth/sand/mud<br>2. Natural floor: dung<br>3. Rudimentary floor: wood planks<br>4. Rudimentary floor: palm/bamboo<br>5. Finished floor: parquet or polished wood<br>6. Finished floor: vinyl or asphalt strips<br>7. Finished floor: ceramic tiles<br>8. Finished floor: cement<br>9. Finished floor: carpet<br>10. Finished floor: chips/terrazzo<br>11. Finished floor: bricks<br>12. Finished floor: mats<br>13. Finished floor: marble<br>97. Other	
Q214	[MAIN MATERIAL OF THE ROOF- RECORD OBSERVATION]	1. Natural roof: no roof<br>2. Natural roof: thatch/palm leaf<br>3. Natural roof: sod/grass<br>4. Rudimentary roof: rustic mat<br>5. Rudimentary roof: palm/bamboo<br>6. Rudimentary roof: wood planks<br>7. Rudimentary roof: cardboard<br>8. Finished roof: iron sheets/asbestos<br>9. Finished roof: reinforced brick cement/rcc metal<br>10. Finished roof: wood/t iron/mud	

For peer review only - http://bmjopen.bmj.com/site/about/guidelines.xhtml

[Name of Country]
[Name of Slum]

		11. Finished roof: calamine/cement fiber<br>12. Finished roof: ceramic tiles<br>13. Finished roof: cement/rcc<br>14. Finished roof: roofing shingles<br>97. Other	
Q215	[MAIN MATERIAL OF THE EXTERIOR WALLS-RECORD OBSERVATION]	1. Natural walls: no walls<br>2. Natural walls: cane/palm/trunks<br>3. Natural walls: dirt<br>4. Natural walls: mud/stones<br>5. Natural walls: bamboo/sticks/mud<br>6. Rudimentary walls: unbaked bricks/mud<br>7. Rudimentary walls: carton/plastic<br>8. Rudimentary walls: bamboo with mud<br>9. Rudimentary walls: stone with mud<br>10. Rudimentary walls: uncovered adobe<br>11. Rudimentary walls: plywood<br>12. Rudimentary walls: cardboard<br>13. Rudimentary walls: reused wood<br>14. Finished walls: baked bricks<br>15. Finished walls: tent<br>16. Finished walls: cement<br>17. Finished walls: stone with lime/cement<br>18. Finished walls: bricks<br>19. Finished walls: cement blocks<br>20. Finished walls: covered adobe<br>21. Finished walls: wood planks/shingles<br>97. Other	
Q216	How many rooms in this household are used for sleeping?	☐☐ Rooms (Sleeping)	
Q217	Does any member of this household own:	1. Watch<br>2. Mobile telephone<br>3. Bicycle<br>4. Motorcycle or motor scooter<br>5. An animal-drawn cart<br>6. A car or truck or bus<br>7. A tractor<br>8. A boat with a motor<br>9. A boat without a motor	
Q217A	Does your household own this structure (house, flat, shack)?	1. Yes<br>2. No	
Q217B	Does your household own the land on which the structure (house, flat, shack) sits?	1. Yes<br>2. No	
Q217C	Do you pay rent?	10. Pays rent/lease<br>11. No rent with consent of owner<br>1. No rent: squatting	
Q218	Does any member of this household own any agricultural land?	2. Yes<br>3. No……………………………. →	Q222

For peer review only - http://bmjopen.bmj.com/site/about/guidelines.xhtml

[Name of Country]
[Name of Slum]

Q219	Does this household own any livestock, herds, other farm animals or poultry?	1. Yes<br>2. No………………………………. →	Q224
Q220	How many of the following animals does this household own?<br><br>Milk cows or bulls?<br>Horses, donkeys or mules?<br>Goats?<br>Sheep?<br>Chickens?<br>Buffalo?<br>Camels?<br>Other?		
Q221	Does any member of this household have a bank account?	1. Yes<br>2. No	
Q222	In the past 7 days were there days when your household did not have enough food or money to buy food?	1. Yes<br>2. No	
Q223	How many days in the month do you have air time (for calls and SMS) for at least one mobile phone in the household	1. Everyday<br>2. Almost every day (over 21 days a month but not every day)<br>3. More than half the days (over 14 but not as many as 21)<br>4. More than 7 days but less than 14<br>5. Less than 7 days<br>6. Never	
Q224	How many days in the month does someone in the household have data or access to wifi (for accessing internet for searching the web, using social media or using Email) for at least one of your digital communication devices I the household (smart phone, laptop, tablet)?	1. Everyday<br>2. Almost every day (over 21 days a month but not every day)<br>3. More than half the days (over 14 but not as many as 21)<br>4. More than 7 days but less than 14<br>5. Less than 7 days<br>6. Never	

Household income and expenditure

In the last part of this section, I will ask you about the total income for the household in the last 30 days. I would like to know about all sources of income. I know it may be difficult to calculate that figure, but please do try to give as accurate an amount as possible. Remember that all information will be kept strictly confidential. This information is important to assess overall health and wellbeing of people in your household compared to other, similar households.

Q225	I would like you to tell me your best estimate of the TOTAL income that this household has had in the last 30 days.	1. Less than 1000 KSh<br>2. 1000 – 2499 KSh<br>3. 2500 – 4999 KSh<br>4. 5000 – 7499 KSh<br>5. 7500 – 9999 KSh<br>6. 10000 – 14999 KSh<br>7. 15000 – 20000 KSh<br>8. Above 20000 KSh	

For peer review only - http://bmjopen.bmj.com/site/about/guidelines.xhtml

[Name of Country]
[Name of Slum]

Q226	Did your household receive or earn any income from the following in the last 30 days?	1. Salaried/waged employment<br>2. Business<br>3. Savings<br>4. Agriculture<br>5. Financial gifts/ support from any source<br>6. Any other source of income	

How much in total did your household spend on the following items?<br><br>[IF RESPONDENT UNSURE, PLEASE PROMPT WITH "TO THE BEST OF YOUR RECOLLECTION" THEN "CAN YOU ESTIMATE APPROXIMATELY WHAT IT MIGHT HAVE BEEN?"]			
Q227a	FOOD in the last 7 days?	☐☐☐☐☐☐☐☐<br>8. Don't know	
Q227b	ENERGY (PARAFFIN, CHARCOAL) in the last 7 days?	☐☐☐☐☐☐☐☐<br>8. Don't know	
Q227c	WATER in the last 7 days?	☐☐☐☐☐☐☐☐<br>8. Don't know	
Q227d	TRANSPORT in the last 7 days?	☐☐☐☐☐☐☐☐<br>8. Don't know	
Q227e	FINANCIAL GIFT/SUPPORT TO OTHERS in the last 7 days?	☐☐☐☐☐☐☐☐<br>8. Don't know	
Q227f	ELECTRICITY in the last 30 days?	☐☐☐☐☐☐☐☐<br>8. Don't know	
Q227g	HEALTH CARE in the last 30 days?	☐☐☐☐☐☐☐☐<br>8. Don't know	
Q227h	RELIGIOUS OBLIGATIONS in the last 30 days?	☐☐☐☐☐☐☐☐<br>8. Don't know	
Q227i	RENT in the last month?	☐☐☐☐☐☐☐☐<br>8. Don't know	
Q227i	SCHOOL RELATED EXPENSES (school fees, scholastic materials) in the last 30 days?	☐☐☐☐☐☐☐☐<br>8. Don't know	
Q227k	OTHER in the last 7 days?	☐☐☐☐☐☐☐☐<br>8. Don't know	

For peer review only - http://bmjopen.bmj.com/site/about/guidelines.xhtml

[Name of Country]
[Name of Slum]

For peer review only

For peer review only - http://bmjopen.bmj.com/site/about/guidelines.xhtml

[Name of Country]
[Name of Slum]

Module 3: Household Health Care Spending

I would like to ask you more specific questions about how much your household and all its members spent in cash or in-kind on all health care and services that did not require an overnight stay. We want expenses in the last 3 months. If payment was in-kind, please estimate a monetary value. Please exclude costs to be reimbursed by insurance.

In the last 3 months, how much did your household spend on:		
Q301	Registration and consultation fees by doctors, nurses, or trained midwives that did not require an overnight stay?	☐☐☐☐☐☐☐☐<br>8. Don't know
Q302	Health-care by traditional or alternative healers? [USE COUNTRY-SPECIFIC TERMS]	☐☐☐☐☐☐☐☐<br>8. Don't know
Q303	Diagnostic and laboratory tests, such as x-rays or blood tests?	☐☐☐☐☐☐☐☐<br>8. Don't know
Q304	Medications or drugs (prescription, non-prescription, traditional, homeopathic…)?	☐☐☐☐☐☐☐☐<br>8. Don't know
Q305	Dentists or dental care?	☐☐☐☐☐☐☐☐<br>8. Don't know
Q306	Ambulance or other transport?	☐☐☐☐☐☐☐☐<br>8. Don't know
Q307	Costs associated with overnight stays in a hospital or health facility? Please exclude any reimbursements from insurance and transportation costs.	☐☐☐☐☐☐☐☐<br>8. Don't know
Q308	Any other health care products or services that were not included above? Please specify:<br><br>[PROMPT FOR FOOD COSTS IF PROVIDED TO HOUSEHOLD MEMBERS DURING HOSPITAL/CLINIC STAYS; PROMPT FOR HOME BASED CARE PROVIDED FOR HOUSEHOLD MEMBERS (ADULT OR CHILD) DUE TO LONG-TERM CONDITIONS OR GETTING OLD AND WEAK]	☐☐☐☐☐☐☐☐<br>8. Don't know

For peer review only - http://bmjopen.bmj.com/site/about/guidelines.xhtml

For peer review only

[Name of Country]
[Name of Slum]
Finally, I want you to think of how you paid for your health care expenditures over the last 12 months. This includes costs for all fees, services and goods, including overnight stays.

Q309	In the last 12 months , have you borrowed from financial institutions, agencies (microfinance schemes, banks…), or individuals to pay for any health expenditures?	1. Yes<br>2. No<br>8. Don't know	
Q310	If you borrowed any money in the last 12 months to pay for health expenditures, are you expected to pay this back?	1. Yes……………………………………… →<br>2. No…………………………………… →	Q311<br>END
Q311	Have you started paying back the loan?	1. Yes……………………………………… →<br>2. No…………………………………… →	Q312<br>END
Q311A	Are you repaying or have you repayed the loan as a one-off payment or in installments?	1. Lump sum<br>2. Installments	Q312A<br>Q312B
Q312A	How much did you pay back?	________	Q401
Q312B	What is the monthly repayment on the loan including interest?<br><br>[FOR INFORMAL PAYMENT, PLEASE TEASE OUT THE AVERAGE MONTHLY REPAYMENT, IF ANY]	☐☐☐☐☐☐☐☐<br><br>8. Don't know	

END: Thank you for your time in completing the household questionnaire. I will now move on to complete an individual questionnaire with a member of your household.

For peer review only

SUPPLEMENTARY INFORMATION

A Health system indicators

Table A1 reports major health system and population health indicators for the participating countries.

Table A1. Key indicators of health care systems in participating countries (Source: World Bank and WHO Global Health Observatory[1])

Key indicators	Nigeria	Kenya	Pakistan	Bangladesh
Life expectancy	54	66	67	72
GDP per capita (PPP; Int$)	5,991	3,468	5.572	4,372
Under five mortality (per 1,000 births)	120	41	69	30
Out of pocket expenditure as % of all health expenditures	75%	28%	65%	72%
Government health expenditure per capita (Int$)	27	52	40	16
Proportion with expenditures on healthcare greater than 10% of total household expenditure	15%	5%	13%	25%
Antenatal care coverage – at least four visits	49%	105%	51%	37%
ARV coverage among those with HIV	53%	68%	10%	22%
Hib (Hib3) coverage among one-year-olds	57%	92%	75%	98%
Tetanus coverage among neonates	60%	88%	85%	98%
Tuberculosis effective treatment coverage	20%	39%	64%	58%
Average of 13 International Heath Regulation core capacity scores	51	58	51	78

GDP: gross domestic product; Hib: Haemophilus influenzae type b vaccine; Int$: international dollar; PPP: purchasing power parity

For peer review only

B Supplementary information on methods

B1 Definitions of health care facilities

Table B1 Definitions of healthcare facilities used in the mapping and surveying of healthcare facilities at each site

Facility type	Definition
Clinic/centre	A facility used for the diagnosis and treatment of outpatients. Allopathic only. Subcategories include specialist clinics: vaccination; family planning; eye and vision; ear, nose, and throat; maternity and antenatal care; palliative care; and general primary or community care.
Dental clinic	A facility providing dentistry/dental care.
Hospital/secondary care facility	A health care organization that has a governing body, an organized medical staff and professional staff and inpatient facilities and provides medical nursing and related services for ill and injured patients 24 hrs per day, seven days per week
Residential or nursing care facility	Facilities that provide inpatient care specializing in nursing or long-term residential care but which are not hospital
Laboratory or sonologist clinic	A room or building equipped for testing, researching, or studying fluids, materials, or tissues from patients.
Pharmacy or medical store	A facility used by pharmacists for the compounding and dispensing of medicinal preparations and other associated professional and administrative services
Transportation services	Provider who moves a patient, specimen, or equipment from one location or another
General shop or kiosk selling healthcare products	Non-specialised vendor without healthcare professional selling health care products such as pain killers, first aid, or spectacles
Traditional practitioner	A practitioner of non-allopathic, traditional, alternative, or faith-based health services. Sub-categories include: traditional medicine, faith-based services, homeopathy, acupuncture, bone healing, and cupping.
Traditional products vendor	Vendor specialising in traditional (non-allopathic) medical products such as herbs.

B2 Supplementary Survey in Site PK1

An error with the electronic survey forms was identified following data collection in site PK1. The household survey form was completed successfully, including the household roster, however due to an error in the code to generate a randomly sampled adult the first recorded adult was instead sampled. This resulted in an almost exclusively male sample, which was not identified in the field at the time. After identification of this error we aimed to complete a set of individual surveys with randomly sampled women from the household rosters. However, the Covid-19 pandemic had started, so a telephone survey was deemed to be safer for the field workers. We sampled 400

For peer review only

households with at least one adult women and who had provided consent and contact details for follow-up from the original sample of households. Field workers then followed the same procedures as the main survey. Survey weights were modified for the sample from PK1 to reweight gender back to the population proportion.

B3 Statistical methods

Choice model

Choices

We define the choice set facing individual as: private clinic, public clinic, private hospital, and public hospital. In sites BD1 and PK1 we also include “private office” as a fifth option as a number of visits were recorded to these locations, whereas there were little to no visits to this type of provider in the other sites.

Choice-varying covariates

We consider two covariates that vary for each individual and choice: the price of the visit and the time to travel to the provider. The price includes consultation fees and transport cost. The price is reported in local currency (Naira, Kenyan Shillings, Pakistani Rupees, Taka). The time to provider is the travel time reported for the visit in minutes. We standardize both cost and time (dividing by the standard deviation) to ensure all variables are on the same scale to facilitate model fitting.

Individual-level covariates

From the survey data we extract the following individual level variables: age, sex, secondary education or not, if the visit was for an acute or communicable condition (excluding HIV), if the visit was for generalised or chronic pain, and if the monthly consumption expenditure was above Int$100 per person per month. For use in the models we standardize the age variable to facilitate model fitting.

Statistical Model

We take a Bayesian approach to model specification and fitting given the complex hierarchical structure of the model.

For peer review only

Each individual $i \in 1{:}N$ chooses from $k \in 1{:}K$ possible options (K is either 4 or 5). The healthcare provider options are described by a vector of choice attributes for each individual $X_{ik} = [C_{ik}, C_{ik}^2, T_{ik}]'$. This vector includes the cost, cost squared, and time. We include cost squared to allow for more flexibility between demand and price. Each individual has "part worth" preferences over the choice attributes β_i so that the utility of each choice for each individual is:

$$u_{ik} = \theta_k + X_{ik}\beta_i + u_{ik}$$

where u_{ik} is IID Gumbel distributed and θ_k are choice constant terms. Given the assumed distribution of the random error, the probability an individual chooses choice k is

$$\Pr(y_{ik} = k | X_{ik}, \beta_i, \theta_k) = \frac{\exp(\theta_k + X_{ik}\beta_i)}{\sum_{j=1}^{K} \exp\left(\theta_j + X_{ij}\beta_i\right)}$$

For more information see[2].

Hierarchical prior on model parameters

The model parameters β_i are allowed to vary by individual – they are so-called "random parameters". To model these parameters we use a hierarchical prior that "partially pools" information across individuals. We also allow these parameters to vary according to a vector of individual-level covariates W_i and further, we allow for correlation between parameters so that, for example, individuals more sensitive to price might be less sensitive to distance. In particular,

$$\beta_i \sim MVN(\beta + \Gamma W_i, \Sigma)$$

where β are mean values, Γ is a matrix that "loads" the individual covariates on to the parameters, and Σ is a covariance matrix. We can decompose the covariance matrix as $\Sigma = \mathrm{diag}(\tau)\Omega\,\mathrm{diag}(\tau)$, where τ is a scale vector and Ω is the correlation matrix of the variation across individuals. The correlation matrix can be further decomposed as $\Omega = L_\Omega L_\Omega'$ where L_Ω is the lower-triangular Cholesky decomposition. Therefore,

$$\beta_i = \beta + \Gamma W_i + \mathrm{diag}(\tau) L_\Omega z_i$$

$$z_i \sim N(0,1)$$

Missing data

We use an imputation-type procedure for the costs and times that we do not observe, i.e. for the choices not taken. The model below is part of the overall model and is estimated at the same time

For peer review only - http://bmjopen.bmj.com/site/about/guidelines.xhtml

so that the uncertainty in the imputed costs and times is carried through to the overall results. We explain the procedure for costs but use an identical model for times. Given that both variables are (often heavily) right-skewed we use log costs and time for the missing data models. We observe N values for log cost, one for each individual: $c_{ik}^{(obs)}$. We specify:

$$c_{ik}^{(obs)} = \mu_k + Z_i\gamma + u_i$$

where μ_k is a choice-specific intercept, Z_i is a vector of individual level covariates that may predict the cost of the visit (age, age squared, sex, reason for visit), γ are model parameters, and $u_i \sim N(0, \sigma_u^2)$ are IID error terms. We fit the above model using the observed data and then for the unobserved costs we sample from:

$$c_{ik}^{(mis)} \sim N(\mu_k + Z_i\gamma, \sigma_u^2)$$

so that the cost covariate is

$$C_{ik} = \begin{cases} \exp\left(c_{ik}^{(obs)}\right) & if\ y_{ik} = 1 \\ \exp\left(c_{ik}^{(mis)}\right) & if\ y_{ik} = 0 \end{cases}$$

Priors

We specify weakly informative priors on the model parameters (all N(0,1) except for the correlation matrix for which we use the lkj prior).

Estimation

We use Stan 2.19 to fit the model[3].

Elasticity of demand

To quantify the response to price or travel time we calculate the price and travel time elasticity of demand. We do this by calculating the arc elasticity:

$$\frac{(\text{Pr1} - \text{Pr0})/((\text{Pr1} + \text{Pr0})/2)}{(p_1 - p_0)/((p_1 + p_0)/2)}$$

where p_1 and p_0 are different prices or times and Pr1 and Pr0 are the probabilities of visiting a provider under each of the two prices or times, respectively.

For peer review only

C Additional Results

Table C1. Respondent care needs and outpatient primary care use, N(%)

Outcome	NG1	NG2	NG3	KE1	KE2	PK1	BD1
Adults							
Total	1,278 (100%)	840 (100%)	802 (100%)	1,008 (100%)	1,085 (100%)	1,112 (100%)	990 (100%)
Needed healthcare in the previous 12 months	772 (60%)	490 (58%)	418 (52%)	710 (70%)	906 (84%)	623 (56%)	959 (97%)
Received care when last needed	762 (60%)	481 (57%)	404 (50%)	674 (67%)	884 (81%)	596 (54%)	918 (93%)
Outpatient visit in last 12 months	626 (49%)	367 (44%)	309 (39%)	665 (66%)	867 (80%)	531 (48%)	900 (91%)
Outpatient visit to doctor or nurse	391 (31%)	215 (26%)	201 (25%)	405 (40%)	377 (35%)	498 (45%)	257 (26%)
Outpatient visit to doctor or nurse for new condition or routine visit	293 (23%)	132 (16%)	151 (19%)	310 (31%)	315 (29%)	291 (26%)	174 (18%)
Children (under 12)							
Total	128 (100%)	69 (100%)	79 (100%)	537 (100%)	421 (100%)	528 (100%)	658 (100%)
Needed healthcare in the previous 12 months	68 (53%)	29 (42%)	41 (52%)	482 (90%)	368 (87%)	409 (77%)	635 (97%)
Received care when last needed	68 (53%)	28 (41%)	41 (52%)	466 (87%)	363 (86%)	397 (75%)	627 (95%)
Outpatient visit in last 12 months	53 (41%)	24 (35%)	39 (49%)	462 (86%)	358 (85%)	374 (71%)	608 (92%)
Outpatient visit to doctor or nurse	36 (28%)	13 (19%)	24 (30%)	320 (60%)	205 (49%)	367 (70%)	136 (21%)
Outpatient visit to doctor or nurse for new condition	27 (21%)	11 (16%)	19 (24%)	283 (53%)	190 (45%)	329 (62%)	120 (18%)

For peer review only

Table C2. Summary statistics of study populations and national comparisons (DHS surveys – national results and urban results in parentheses where relevant)

Variable		Nigeria				Kenya			Pakistan		Bangladesh	
		NG1	NG2	NG3	DHS 2018	KE1	KE2	DHS 2014	PK1	DHS 2017/8	BD1	DHS 2014
Households												
N (completed)		1,286	845	812	-	1,018	1,089	-	988	-	1,035	-
Response rate (%)		83	69	68	99.3	69	57	99	73	96	94	99
Household size		3.7 (1.9)	3.4 (1.8)	4.2 (2.0)	4.3	3.2 (2.2)	3.4 (1.7)	3.9	5.7 (3.0)	6.6	3.8 (1.8)	4.5
Wealth quintile (%)	Bottom	0	0	0	20 (4.2)	0	0	20 (6)	0	20 (3)	0	20 (7)
	Lower	2	1	1	20 (8.1)	14	0	20 (8)	0	20 (7)	0	20 (6)
	Middle	50	59	23	20 (18.8)	59	53	20 (11)	84	20 (17)	21	20 (12)
	Upper	48	40	76	20 (30.6)	27	46	20 (26)	14	20 (31)	78	20 (26)
	Top	0	0	0	20 (38.4)	7	1	20 (49)	2	20 (42)	1	20 (49)
Monthly expenditure (Int$), median [IQR]	Total	312 [197, 487]	323 [210, 496]	502 [319, 815]	-	193 [131, 305]	187 [133, 295]	-	1,010 [672, 1,471]	--	490 [321, 770]	-
	Per person	97 [59, 162]	106 [69, 172]	144 [94, 231]	-	74 [45, 125]	96 [61, 156]	-	196 [146, 294]	-	110 [80, 162]	-
Individuals												
Age (%)	Under 5	11	9	10	15.4	13	12	14	11	13	11	10
	5 – 19	34	31	33	36.4	37	26	38	30	36	32	34
	20 – 44	36	35	39	32.0	37	51	33	41	34	45	36
	45 – 64	14	17	15	11.8	11	10	11	15	13	10	15
	65 and over	5	8	3	4.1	3	1	4	3	4	2	6
Male (%)		49	47	50	-	50	55	-	50	-	53	-
Education over 18s (%)	Completed primary	13	16	17	24.7	25	23	16	13	9	31	9
	Completed secondary	24	31	34	31.7	13	30	11	9	9	4	4
	Completed tertiary	11	6	20	12.1	5	6	7	0	12	4	10
Currently working over 18 (%)		72	76	74	75.5	65	77	81	57	96	75	98

For peer review only - http://bmjopen.bmj.com/site/about/guidelines.xhtml

Table C3. Outpatient primary care consultation rates and outpatient doctor and nurse consultation rates (visits per person-year (95% CI)).

Site	Outpatient consultation (new conditions)			Outpatient consultation (all conditions)		
	Crude	WHO age adjusted	INDEPTH age adjusted	Crude	WHO age adjusted	INDEPTH age adjusted
Adults						
NG1	0.31 (0.29, 0.33)	0.30 (0.28, 0.32)	0.30 (0.28, 0.32)	0.42 (0.40, 0.44)	0.42 (0.40, 0.44)	0.40 (0.38, 0.42)
NG2	0.25 (0.23, 0.27)	0.21 (0.19, 0.23)	0.20 (0.18, 0.22)	0.39 (0.35, 0.43)	0.32 (0.30, 0.34)	0.29 (0.27, 0.31)
NG3	0.21 (0.19, 0.23)	0.23 (0.21, 0.25)	0.22 (0.20, 0.24)	0.28 (0.26, 0.30)	0.31 (0.29, 0.33)	0.29 (0.27, 0.31)
KE1	0.77 (0.73, 0.81)	0.77 (0.73, 0.81)	0.72 (0.68, 0.76)	1.07 (1.03, 1.11)	1.17 (1.13, 1.21)	1.04 (1.00, 1.08)
KE2	0.76 (0.72, 0.80)	0.73 (0.69, 0.77)	0.70 (0.66, 0.74)	0.93 (0.89, 0.97)	1.06 (1.02, 1.10)	0.95 (0.91, 0.99)
PK1	0.60 (0.56, 0.64)	0.58 (0.54, 0.62)	0.56 (0.52, 0.60)	0.79 (0.75, 0.83)	0.85 (0.81, 0.89)	0.77 (0.73, 0.81)
BD1	1.08 (1.04, 1.12)	1.21 (1.15, 1.26)	1.16 (1.12, 1.20)	1.52 (1.46, 1.58)	1.73 (1.67, 1.79)	1.59 (1.53, 1.65)
Children (under 12)						
NG1	0.23 (0.15, 0.31)	-	-	0.29 (0.19, 0.39)	-	-
NG2	0.12 (0.04, 0.20)	-	-	0.15 (0.05, 0.25)	-	-
NG3	0.26 (0.14, 0.38)	-	-	0.34 (0.20, 0.48)	-	-
KE1	1.51 (1.41, 1.61)	-	-	1.74 (1.62, 1.86)	-	-
KE2	1.21 (1.11, 1.31)	-	-	1.30 (1.18, 1.42)	-	-
PK1	1.68 (1.56, 1.80)	-	-	1.85 (1.73, 1.97)	-	-
BD1	0.91 (0.83, 0.99)	-	-	1.04 (0.96, 1.12)	-	-
Children (under 5)						
NG1	0.24 (0.10, 0.38)	-	-	0.30 (0.14, 0.46)	-	-
NG2	0.13 (0.00, 0.27)	-	-	0.21 (0.03, 0.39)	-	-
NG3	0.38 (0.14, 0.62)	-	-	0.40 (0.16, 0.64)	-	-
KE1	2.17 (1.97, 2.37)	-	-	2.57 (2.35, 2.79)	-	-
KE2	1.62 (1.44, 1.80)	-	-	1.68 (1.50, 1.86)	-	-
PK1	2.33 (2.11, 2.55)	-	-	2.46 (2.24, 2.68)	-	-
BD1	1.35 (1.21, 1.49)	-	-	1.50 (1.36, 1.63)	-	-

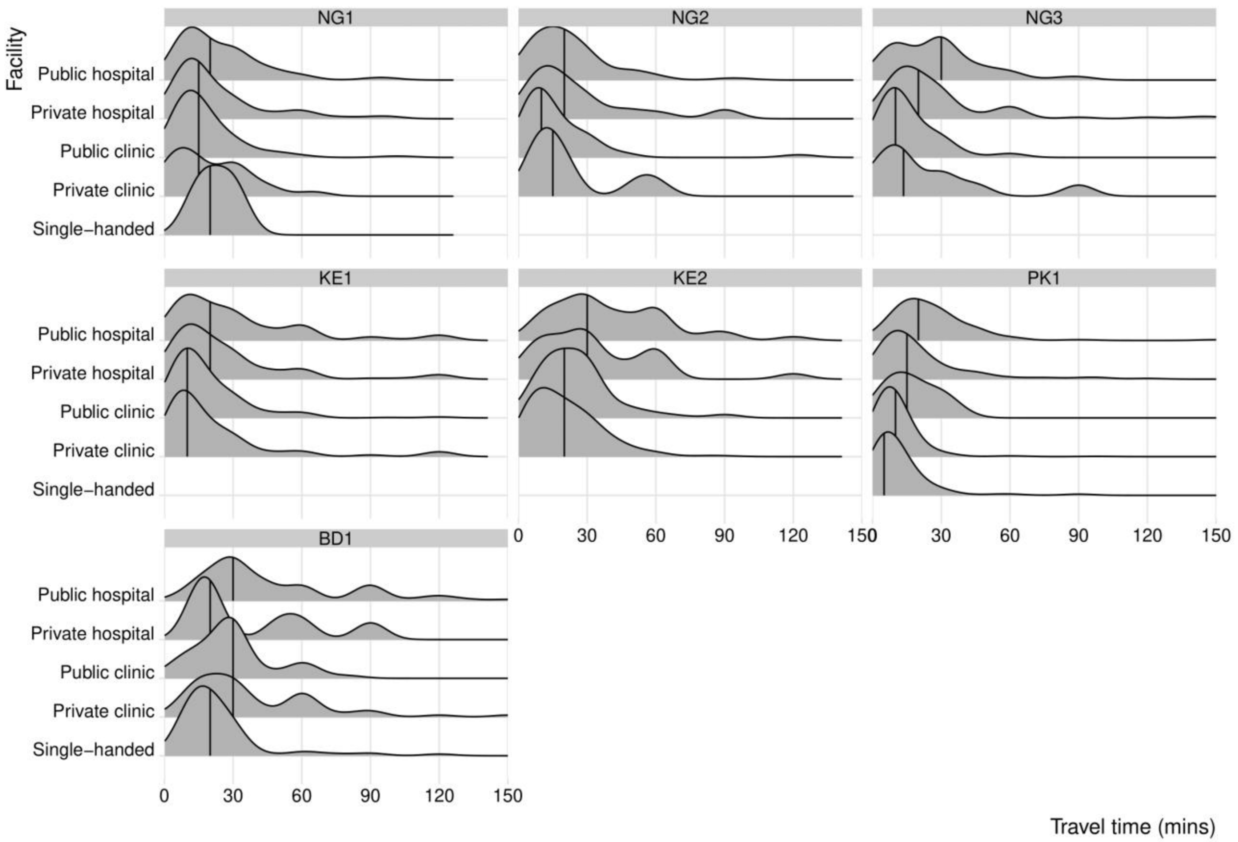

Figure C1 Distribution of reported travel times to different providers by study site with vertical lines indicating the median of the distribution.

Figure C2 Reported "primary reason" for visiting the doctor or nurse.

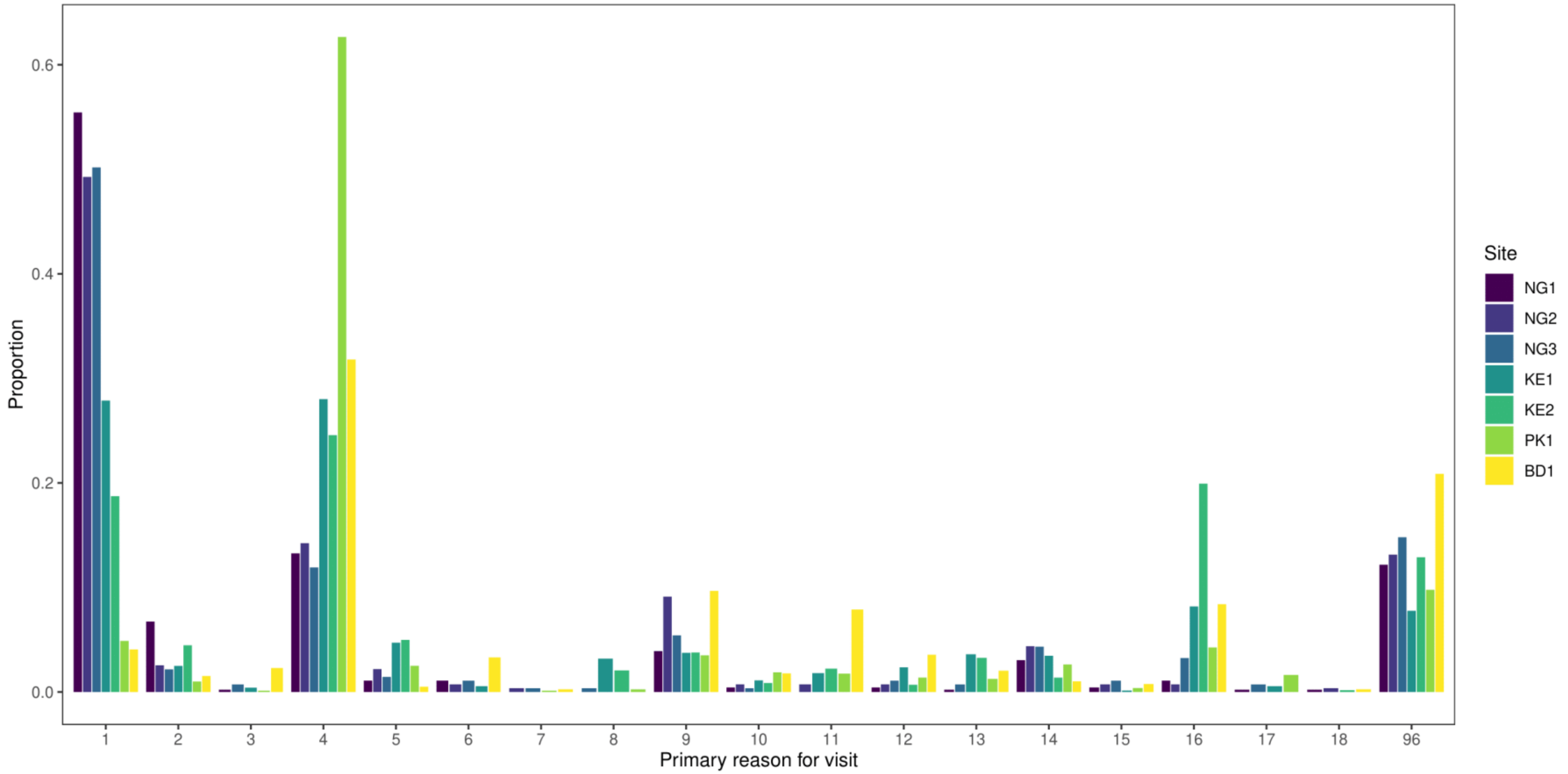

The reasons for visiting were: 1 Communicable disease (infections, malaria, tuberculosis, HIV), 2 Maternal and perinatal conditions (pregnancy), 3 Nutritional deficiencies, 4 Acute conditions (diarrhoea, fever, flu, headaches, cough, other), 5 Injury (not work related, see 8 below), 6 Surgery, 7 Sleep problems, 8 Occupation/work related condition/injury, 9 Chronic pain in your joints / arthritis (joints, back, neck), 10 Diabetes or related complications, 11 Problems with your heart, including unexplained pain in chest, 12 Problems with your mouth, teeth or swallowing, 13 Problems with your breathing, 14 High blood pressure / hypertension, 15 Stroke / sudden paralysis of one side of body, 16 Generalised pain (stomach, muscle or other non-specific pain), 17 Depression or anxiety, 18 Cancer, 96 Other (Specify)

Table C4 Percentage of respondents reporting reasons for choosing a healthcare providers by provider type

	Nigeria			Kenya		Pakistan	Bangladesh
	NG1	NG2	NG3	KE1	KE2	PK1	BD1
Nearness of the facility	63	69	57	78	72	61	54
Service providers are cordial	34	24	32	21	22	29	65
Good services available	38	32	44	31	37	37	95
Short waiting times	17	11	16	7	3	9	7
Qualified doctors are available	24	29	35	22	17	51	26
Low fees/treatment costs	28	33	43	36	24	36	39
Good waiting arrangement	12	9	15	3	2	7	5
Confidentiality is maintained	13	10	21	1	1	18	0
Don’t know where else to go	7	15	7	12	6	5	3
Medicines are available	21	17	25	35	22	9	4
Diagnostics are available	11	12	11	13	4	1	11
Recommendation	11	6	11	16	8	9	20

For peer review only

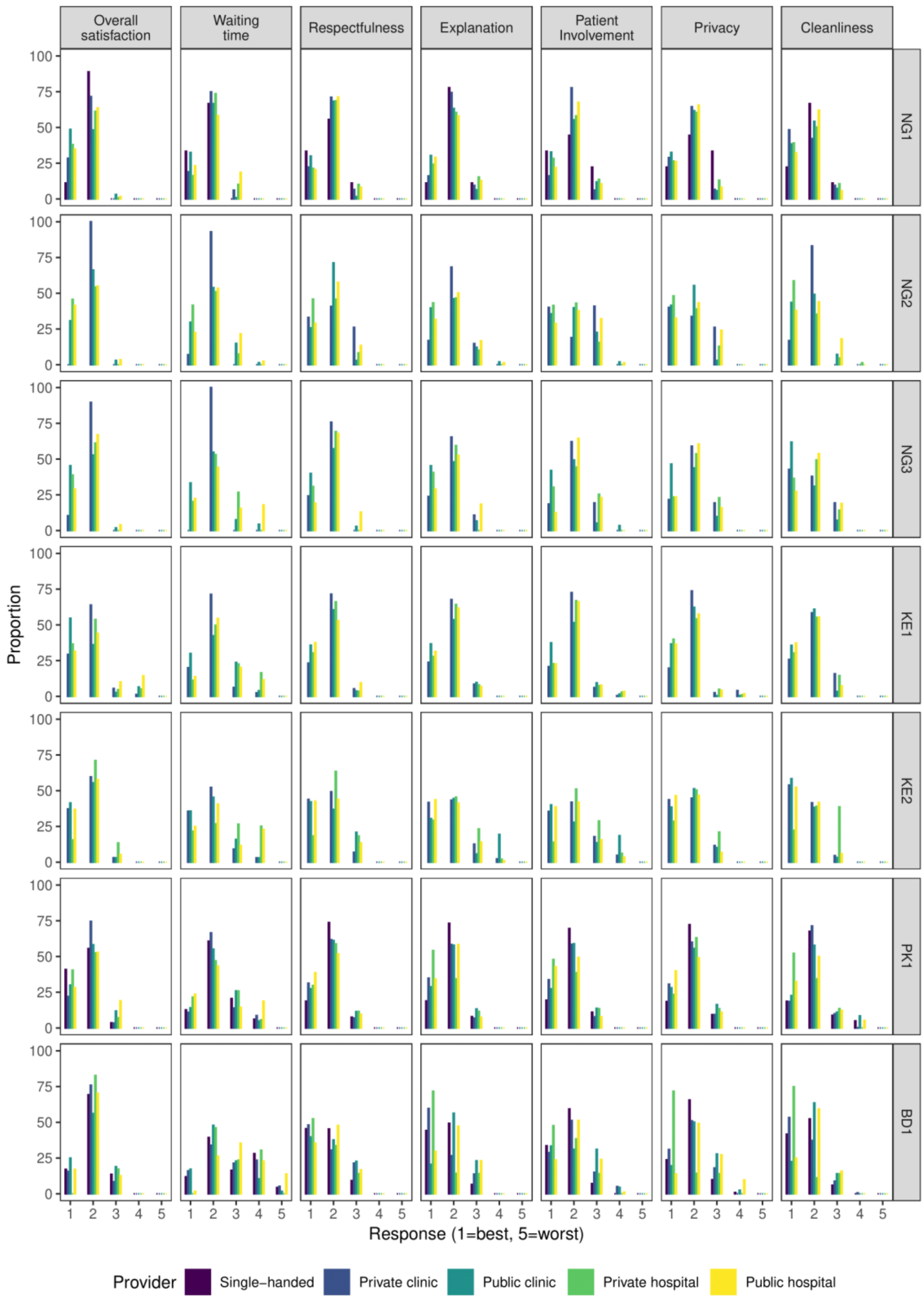

Figure C3. Satisfaction with outpatient consultations by type of facility and by site. Responses were on five-point Likert scales, either Very satisfied to very dissatisfied (Overall satisfaction) or Very good to very bad (Other questions).

REFERENCES

1 World Health Organization. Global Health Observatory. 2021.https://www.who.int/data/gho (accessed 6 Jun 2021).

2 J. Borah B. A mixed logit model of health care provider choice: analysis of NSS data for rural India. *Health Econ* 2006;**15**:915–32. doi:10.1002/hec.1166

3 Carpenter B, Gelman A, Hoffman M, *et al.* Stan: A Probabilistic Programming Language. *J Stat Softw* 2016;**VV**.

For peer review only

STROBE Statement—Checklist of items that should be included in reports of ***cross-sectional studies***

	Item No	Recommendation	Page #
**Title and abstract**	1	(*a*) Indicate the study's design with a commonly used term in the title or the abstract	1
		(*b*) Provide in the abstract an informative and balanced summary of what was done and what was found	4-5
**Introduction**			
Background/rationale	2	Explain the scientific background and rationale for the investigation being reported	7
Objectives	3	State specific objectives, including any prespecified hypotheses	7-8
**Methods**			
Study design	4	Present key elements of study design early in the paper	8-9
Setting	5	Describe the setting, locations, and relevant dates, including periods of recruitment, exposure, follow-up, and data collection	8-9
Participants	6	(*a*) Give the eligibility criteria, and the sources and methods of selection of participants	8-9
Variables	7	Clearly define all outcomes, exposures, predictors, potential confounders, and effect modifiers. Give diagnostic criteria, if applicable	8-11
Data sources/ measurement	8*	For each variable of interest, give sources of data and details of methods of assessment (measurement). Describe comparability of assessment methods if there is more than one group	*8-11*
Bias	9	Describe any efforts to address potential sources of bias	8-11, 25, 26
Study size	10	Explain how the study size was arrived at	9
Quantitative variables	11	Explain how quantitative variables were handled in the analyses. If applicable, describe which groupings were chosen and why	10-12
Statistical methods	12	(*a*) Describe all statistical methods, including those used to control for confounding	10-13
		(*b*) Describe any methods used to examine subgroups and interactions	N/A
		(*c*) Explain how missing data were addressed	N/A
		(*d*) If applicable, describe analytical methods taking account of sampling strategy	11-12
		(*e*) Describe any sensitivity analyses	N/A
**Results**			
Participants	13*	(a) Report numbers of individuals at each stage of study—eg numbers potentially eligible, examined for eligibility, confirmed eligible, included in the study, completing follow-up, and analysed	14
		(b) Give reasons for non-participation at each stage	N/A
		(c) Consider use of a flow diagram	N/A
Descriptive data	14*	(a) Give characteristics of study participants (eg demographic, clinical, social) and information on exposures and potential confounders	15
		(b) Indicate number of participants with missing data for each variable of interest	N/A
Outcome data	15*	Report numbers of outcome events or summary measures	14-20
Main results	16	(*a*) Give unadjusted estimates and, if applicable, confounder-adjusted estimates and their precision (eg, 95% confidence interval). Make clear	12-13, 20-21

For peer review only

		which confounders were adjusted for and why they were included	
		(*b*) Report category boundaries when continuous variables were categorized	N/A
		(*c*) If relevant, consider translating estimates of relative risk into absolute risk for a meaningful time period	N/A
Other analyses	17	Report other analyses done—eg analyses of subgroups and interactions, and sensitivity analyses	N/A
**Discussion**			
Key results	18	Summarise key results with reference to study objectives	22-24
Limitations	19	Discuss limitations of the study, taking into account sources of potential bias or imprecision. Discuss both direction and magnitude of any potential bias	25, 26
Interpretation	20	Give a cautious overall interpretation of results considering objectives, limitations, multiplicity of analyses, results from similar studies, and other relevant evidence	22-26
Generalisability	21	Discuss the generalisability (external validity) of the study results	22-26
**Other information**			
Funding	22	Give the source of funding and the role of the funders for the present study and, if applicable, for the original study on which the present article is based	5

*Give information separately for exposed and unexposed groups.

Note: An Explanation and Elaboration article discusses each checklist item and gives methodological background and published examples of transparent reporting. The STROBE checklist is best used in conjunction with this article (freely available on the Web sites of PLoS Medicine at http://www.plosmedicine.org/, Annals of Internal Medicine at http://www.annals.org/, and Epidemiology at http://www.epidem.com/). Information on the STROBE Initiative is available at www.strobe-statement.org.

For peer review only
